# *MiR-144-3p* is associated with pathological inflammation in patients infected with *Mycobacteroides abscessus*

**DOI:** 10.1038/s12276-020-00552-0

**Published:** 2021-01-20

**Authors:** Hyeon Ji Kim, In Soo Kim, Sung-Gwon Lee, Young Jae Kim, Prashanta Silwal, Ji Young Kim, Jin Kyung Kim, Wonhyoung Seo, Chaeuk Chung, Hyun Kyu Cho, Hee Jae Huh, Seung Cheol Shim, Chungoo Park, Byung Woo Jhun, Eun-Kyeong Jo

**Affiliations:** 1 Department of Microbiology, Chungnam National University School of Medicine, Daejeon, 35015 Korea; 2 Department of Medical Science, Chungnam National University School of Medicine, Daejeon, 35015 Korea; 3 Infection Control Convergence Research Center, Chungnam National University School of Medicine, Daejeon, 35015 Korea; 4 School of Biological Sciences and Technology, Chonnam National University, Gwangju, 61186 Korea; 5 Division of Rheumatology, Regional Rheumatoid & Degenerative Arthritis Center, Chungnam National University School of Medicine, Daejeon, 35015 Korea; 6 Division of Pulmonary and Critical Care, Department of Internal Medicine, Chungnam National University School of Medicine, Daejeon, 35015 Korea; 7 Division of Pulmonary and Critical Care Medicine, Department of Medicine, Samsung Medical Center, Sungkyunkwan University School of Medicine, Seoul, 06351 Korea; 8 Department of Laboratory Medicine and Genetics, Samsung Medical Center, Sungkyunkwan University School of Medicine, Seoul, 06351 Korea

**Keywords:** Bacterial infection, Bacterial infection, Chemokines, Mechanisms of disease

## Abstract

Infection with rapidly growing nontuberculous mycobacteria is emerging as a global health issue; however, key host factors remain elusive. Here, we investigated the characteristic immune profiles of peripheral blood mononuclear cells (PBMCs) from patients infected with *Mycobacteroides abscessus* subsp*. abscessus* (Mabc) and *M. abscessus* subsp. *massiliense* (Mmass). Using an integrated analysis of global mRNA and microRNA expression profiles, we found that several inflammatory cytokines/chemokines [interleukin (IL)-1β, *IL-6*, C-X-C motif chemokine ligand 2, and C-C motif chemokine ligand 2] and *miR-144-3p* were significantly upregulated in PBMCs from patients compared with those from healthy controls (HCs). Notably, there was a strong correlation between the expression levels of *miR-144-3p* and proinflammatory cytokines/chemokines. Similarly, upregulated expression of *miR-144-3p* and proinflammatory cytokines/chemokines was found in macrophages and lungs from mice after infection with Mabc and Mmass. We showed that the expression of negative regulators of inflammation (*SARM1* and *TNIP3*) was significantly downregulated in PBMCs from the patients, although they were not putative targets of *miR-144-3p*. Furthermore, overexpression of *miR-144-3p* led to a marked increase in proinflammatory cytokines/chemokines and promoted bacterial growth in macrophages. Together, our results highlight the importance of *miR-144-3p* linking to pathological inflammation during *M. abscessus* infection.

## Nontuberculous mycobacteria: Regulatory and signalling molecules that promote inflammation

A small RNA molecule which controls the expression of genes, and cell signaling molecules called cytokines are associated with the damaging inflammation caused by nontuberculous mycobacteria (NTM). NTM infections, which can affect most organs of the body, and are often resistant to most available drugs, are emerging as a serious global health concern. Patients with suppressed immunity are at particular risk. Increased production of a specific microRNA and of a variety of cytokines during NTM infection has been demonstrated by Hyeon Ji Kim and colleagues at Chungnam National University, Daejeon, and co-workers from other institutions in South Korea. Their findings come from comparing peripheral blood mononuclear cells in patients with *Mycobacteroides abscessus* lung infections with those from healthy controls. The researchers found similar increased microRNA and cytokine production in the lungs of infected mice.

## Introduction

There is growing concern over the increasing incidence and prevalence of nontuberculous mycobacteria (NTM) infection worldwide^1^. Among NTM, *Mycobacteroides abscessus*—which includes *M*. *abscessus* subsp. abscessus (Mabc), *M*. *abscessus* subsp. massiliense (Mmass), and *M*. *abscessus* subsp. *bolletii*—exhibits rapid growth. *M*. *abscessus* is clinically significant because of its multidrug resistance and the limited efficacy of the available treatments^2–5^. The risk of *M*. *abscessus* infection is associated with immune deficiency, immunosuppressive therapy, old age, and underlying lung and systemic diseases^6,7^. However, the host immune factors by which protection and pathological responses are modulated during *M*. *abscessus* infection are unclear.

The host immune response is a determinant of the outcome of NTM infection because most NTM strains are ubiquitous and not contagious. While several cytokines, including tumor necrosis factor (TNF) and interleukin (IL)-32, have potential as biomarkers for certain NTM diseases^6–8^, our understanding of the nature and regulation of the immune response to NTM infection is incomplete. In addition, innate immune signaling is tightly regulated to ensure effective immunity and prevent damage by excessive inflammation^9,10^. Thus, it is important for us to identify and quantify key host factors that influence the balance between pathological inflammation and protective immune responses during NTM infection.

MicroRNAs (miRNAs), small noncoding RNA molecules, are critical post-transcriptional regulators of immune and inflammatory responses^11^. MiRNAs also have potential as biomarkers of various infectious diseases^12,13^. Evaluation of the profile and function of differentially expressed miRNAs in NTM patients will enable the development of biomarkers and enhance our understanding of immunopathology during NTM infection.

Here, we sought to investigate the immune mRNA and miRNA signature of peripheral blood mononuclear cells (PBMCs) from untreated pulmonary disease patients infected with Mabc or Mmass. Several proinflammatory cytokines/chemokines were significantly upregulated in PBMCs from the NTM patients compared with those from the healthy controls (HCs). In addition, *miR-144-3p* levels were significantly upregulated in PBMCs from the patients and in murine bone marrow-derived macrophages (BMDMs) after infection with Mabc or Mmass or in infected lung tissues from mice. Further study showed that the levels of *SARM1* (sterile α and TIR motif-containing 1 protein)^14^ and *TNIP3* (TNFAIP3 interacting protein 3)^15^, negative regulators of inflammation, were depressed in PBMCs from the patients compared with those from the HCs, although they were not putative targets of *miR-144-3p*. Furthermore, overexpression of *miR-144-3p* led to a marked increase in inflammatory cytokines/chemokines and increased bacterial growth in BMDMs. Together, these data suggest that pulmonary disease patients with *M. abscessus* infection had increased pathological inflammation through upregulation of *miR-144-3p*.

## Materials and methods

### Ethics statement

Clinical and laboratory data of study patients were obtained from an ongoing Institutional Review Board–approved prospective observational cohort study to investigate NTM pulmonary disease (clinicaltrials.gov identifier NCT00970801). This study was approved by the Institutional Research and Ethics Committee at Chungnam National University Hospital (CNUH 2019-04-046; Daejeon, Korea).

### NTM identification

Sputum was obtained for microbiological evaluation. Acid-fast bacilli smears and cultures were conducted using standard methods. NTM species were identified using polymerase chain reaction (PCR)—reverse blot hybridization assay (REBA) targeting the *rpoB* gene or via nested multiplex PCR—reverse line blot (RLB) hybridization assays of the internal transcribed spacer (ITS) region.

For PCR-REBA targeting of the *rpoB* gene, a REBA Myco-ID kit (YD Diagnostics, Seoul, South Korea) was used. For this assay, 3 μL of template deoxyribonucleic acid (DNA) from clinical isolates was used. The *rpoB* gene region was amplified with biotin-labeled primers. The PCR conditions were as follows: one cycle at 94 °C for 5 min; 45 cycles at 94 °C for 30 s and 65 °C for 30 s; and one cycle at 72 °C for 7 min. The PCR products were hybridized to a membrane with immobilized probes. The data were interpreted using the REBA Myco-ID data sheet provided by the manufacturer.

For the multiplex PCR-RLB hybridization assay of the ITS region, AdvanSure Mycobacteria GenoBlot Assay (LG Life Sciences, Seoul, South Korea) was used. The AdvanSure assay was performed according to the manufacturer’s instructions. Briefly, PCR was performed using 25 μL of reaction mixture containing 2× master mix, 5 μL of primer mixture, and 7.5 μL of sample DNA. The PCR conditions were as follows: one cycle at 50 °C for 2 min and 95 °C for 10 min; 15 cycles at 94 °C for 30 s, 65 °C for 1 min, and 72 °C for 30 s; 38 cycles at 94 °C for 30 s, 55 °C for 1 min, and 72 °C for 30 s; and one cycle at 72 °C for 10 min. Hybridization was performed with 20 μL of the amplified sample and confirmed by observing blue coloration after adding the streptavidin-alkaline phosphatase conjugate. The color intensity of the band was measured using AdvanSure GenoScan (LG Life Science).

### Cell cultures

Human PBMCs from healthy volunteers were isolated from heparinized venous blood using Ficoll-Hypaque (Lymphoprep; Axis-shield, 1114544, Dundee, Scotland) as described previously^16^. For monocyte-derived macrophage (MDM) differentiation, adherent monocytes were incubated in Roswell Park Memorial Institute 1640 medium (Lonza, 12-702 F, Basel, Switzerland) containing 5% pooled human serum (Sigma-Aldrich, H4522, MO, USA) and 1% L-glutamine for 1 h at 37 °C, after which the nonadherent cells were removed. Human MDMs were prepared by culturing peripheral blood monocytes for 4 days in the presence of 4 ng/mL human macrophage colony-stimulating factor (M-CSF) (R&D, 216-MC, MN, USA).

For preparation of the mouse BMDMs, bone marrow was collected and cultured for 3–5 days in Dulbecco’s modified Eagle’s medium (Lonza, 12-604F) containing 10% fetal bovine serum (Serana, S-FBS-US-015, Pessin, Germany) and antibiotics (Lonza, 17-745E) in the presence of M-CSF (R&D, 416-ML). Cells were incubated in a 37 °C humidified atmosphere with 5% carbon dioxide.

### Mice

In this study, 8- to 12-week-old (age-matched) male C57BL/6 (Samtako Bio, Gyeonggi-do, Korea) mice were used. Mice were maintained on a 12 h light/dark cycle under specific pathogen-free conditions. All animal experimental methods and procedures were performed following the relevant ethical guidelines and regulations approved by the Institutional Research and Ethics Committee at Chungnam National University, School of Medicine (201912A-CNU-203; Daejeon, Korea) and the guidelines of the Korean Food and Drug Administration.

### Nanostring nCounter assay and data analysis

Nanostring nCounter Human Immunology gene expression assays and Human miRNA expression assays were performed at PhileKorea Technology (Daejeon, South Korea) using the NanoString nCounter GX Human Immunology Kit V2 (NanoString Technologies, Inc., Seattle, WA, USA) and nCounter Human miRNA Assay Kit V3 (NanoString Technologies), respectively. nSolver, version 4.0 (NanoString Technologies), was utilized for differential gene expression analyses from nCounter assay data. Based on the nSolver 4.0 Analysis Software User Manual, all reporter code count files from nCounter instruments passed the default QC settings. All raw gene expression data were normalized in positive control normalization and housekeeping normalization steps by default. Fold changes were computed by taking the mean of the normalized lanes from healthy controls, and false discovery rate (FDR)-adjusted *p*-values were calculated using the Benjamini-Yekutieli procedure.

### RNA extraction and quantitative real-time (qRT)-PCR

Human PBMCs were isolated from heparinized venous blood using Ficoll-Hypaque (Lymphoprep; Axis-shield, 1114544, Dundee, Scotland) following the manufacturer’s manual. Total RNA from PBMCs was extracted using QIAzol lysis reagent (Qiagen, 1023537, Hilden, Germany) and miRNeasy Mini Kits (Qiagen, 217004) according to the manufacturer’s instructions, followed by RNA quantitation and assessment using QIAxpert (Qiagen).

After RNA quantitation, complementary DNA (cDNA) from total RNA was synthesized using reverse transcription master premix (Elpis Biotech, EBT-1515, Daejeon, South Korea). For miRNA expression analysis, cDNA from total RNA was synthesized using miScript II RT Kits (Qiagen, 218161), as described previously^17^. qRT-PCR was performed in the Rotor-Gene Q 2plex system (Qiagen, 9001620) using SYBR Green master mix (Qiagen, 204076) or miScript SYBR Green PCR Kit (Qiagen, 218073) and primers for the indicated genes. The primer sequences are shown in Supplementary Table 1. Relative expression levels of mRNA and miRNA were calculated with the 2−ΔΔCt method.

### Preparation of mycobacterial strains, infection, and intracellular colony-forming unit (CFU) assays

Mabc (ATCC 19977) and Mmass (KMRC-00136-13018) were incubated at 37 °C using an orbital shaker in Middlebrook 7H9 medium containing 10% oleic albumin dextrose catalase (BD, Franklin Lakes, NJ, USA) until the mid-log phase (OD_600 nm_ = 0.4), as described previously^18^.

Cells were inoculated for 2 h with either Mabc or Mmass, washed three times with phosphate-buffered saline (PBS) to remove extracellular bacteria, and incubated in fresh media in a 37 °C humidified atmosphere with 5% carbon dioxide. For mouse infection in vivo, mice were administered Mabc or Mmass intranasally (i.n.; 1 × 10^7^ CFU/mouse). For determination of the bacterial burden, mice were euthanized at the indicated times after NTM infection. The lungs were removed and homogenized using a tissue homogenizer (OMNI TH; Omni International, GA, USA) in PBS with 0.1% Tween® 20, as described previously^18^.

For analysis of bacterial viability in host cells by CFU assays, infected cells were lysed in distilled water to release intracellular bacteria. Thereafter, the serially diluted homogenates of the infected tissues or cells were inoculated onto 7H10 agar plates, and colonies were counted 3–4 days later.

### Bioinformatics analysis

For each gene trait, the expression levels from the 24 samples were first transformed on a log2 scale before principal component analysis (PCA). PCA was performed using the “prcomp” function in the “stats” package in R (version 3.2.4)^19^ with the first two principal components. We performed in silico analysis to identify putative miRNA target genes using TargetScan Human Release 7.1 (http://www.targetscan.org/)^20^. Functional and pathway enrichment analyses were performed using the DAVID (version 6.8, https://david.ncifcrf.gov)^21^ and PANTHER (version 15.0, http://www.pantherdb.org)^22^ classification systems with a human reference gene set. To visualize volcano plots and heatmaps, we utilized the “plot” function in the “graphics” package in R.

### Cell transfection

The *hsa-miR-144-3p* and *mmu-miR-144-3p* mimics (sense 5′-UACAGUAUAGAUGAUGUACU-3′, antisense 5′-AGUACAUCAUCUAUACUGUA-3′) were the same and purchased from Genolution (Seoul, South Korea). The mimic negative control (4464058) was purchased from Invitrogen (CA, USA). Oligonucleotides were transfected into BMDMs or human primary MDMs using Lipofectamine 2000 (Invitrogen, 11668019) according to the manufacturer’s instructions as described previously^16^. For real-time PCR, an hsa-miR-144-3p primer (MS00020328) and mmu-miR-144-3p primer (MS00032326) were purchased from Qiagen.

### Statistical analysis

Statistical analyses were performed with Analyse-it, version 5.1 (Analyse-it Software, Ltd., Leeds, UK), SPSS Statistics for Windows, version 24.0 (SPSS, Inc., Chicago, IL, USA), and GraphPad Prism, version 5.0 (GraphPad Software, San Diego, CA, USA). The data were processed by principal component analysis, Spearman’s correlation, Student’s *t*-test, Mann–Whitney U test, ANOVA, and Kruskal–Wallis test, as appropriate, and are detailed in each figure and figure legend above. The results are presented as the mean ± SEM (standard error of the mean) and significance (*p < 0.05, **p < 0.01, ***p < 0.001, ns not significant).

## Results

### Clinical characteristics of NTM patients

The characteristics of the 31 Mabc-infected and 22 Mmass-infected patients are presented in Table 1. The mean ages of the Mabc- and Mmass-infected patients were 59 and 55 years, respectively; approximately three-quarters were female and had never smoked. The most common underlying condition was bronchiectasis, followed by previous pulmonary tuberculosis and chronic obstructive pulmonary disease in the Mabc and Mmass groups. None of the patients tested positive for the human immunodeficiency virus. All of the patients had respiratory symptoms, such as cough, sputum, or hemoptysis. On chest computed tomography, 93% of the Mabc-infected and 86% of the Mmass-infected patients showed a nodular bronchiectatic form (Supplementary Fig. 1). The mean erythrocyte sedimentation rates were 33 and 31 mm/h in the Mabc-infected and Mmass-infected patients, respectively. Sputum acid-fast bacilli smears were positive in 48% of the Mabc-infected and 36% of the Mmass-infected patients. There were no significant differences in characteristics between the two patient groups.

**Table 1:** Baseline characteristics of study patients at diagnosis.

Characteristics	Mabc (*n* = 31)	Mmass (*n* = 22)	*P*-value
Age, year	59 (± 2)	55 (± 2)	0.187
Female	22 (71)	16 (73)	0.889
Body mass index, kg/m^2^	20.3 (± 0.4)	20.9 (± 0.4)	0.367
Never smoker	25 (81)	19 (86)	0.585
Underlying conditions			
Bronchiectasis	28 (90)	20 (91)	0.943
Previous pulmonary tuberculosis	11 (36)	8 (36)	0.948
Chronic obstructive pulmonary disease	4 (13)	1 (5)	0.389
Diabetes mellitus	2 (7)	0 (0)	0.505
Sinusitis	0 (0)	1 (5)	0.415
Symptoms			
Cough	20 (65)	15 (68)	0.781
Sputum	25 (81)	17 (77)	0.765
Hemoptysis	7 (23)	10 (46)	0.079
Type of disease			0.229
Noncavitary nodular bronchiectatic	25 (80)	13 (58)	
Cavitary nodular bronchiectatic	4 (13)	6 (28)	
Fibrocavitary	2 (7)	3 (14)	
Erythrocyte sedimentation rate, mm/h	33 (±4)	31 (±6)	0.417
C-reactive protein, mg/dl	0.47 (±0.16)	0.57 (±0.25)	0.657
Albumin, g/dl	4.3 (±0.1)	4.2 (±0.10)	0.355
Sputum smear positivity	15 (48)	8 (36)	0.415

Data are presented as the mean (± standard error of the mean) or numbers (%). Mabc = *M. abscessus* subsp. *abscessus*, Mmass = *M. abscessus* subsp. *massiliense*.

### Immune transcriptome analysis of the NTM patients and the HCs

**Fig. 1:**
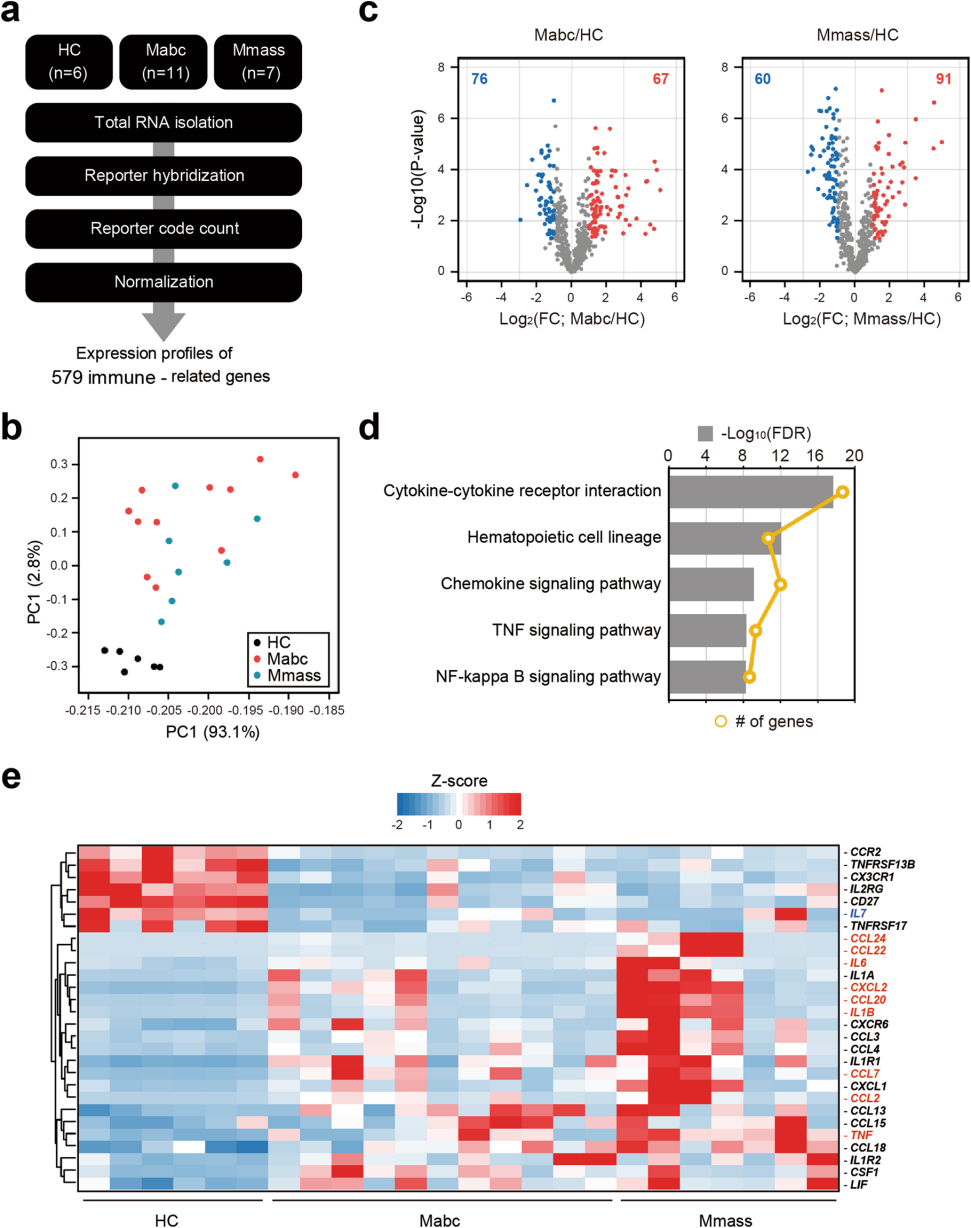
Immune transcriptome analysis reveals differential expression of proinflammatory cytokines/chemokines between PBMCs from the NTM patients and the HCs. **a** Schematic diagram of the immune transcriptome analysis in this study. **b** Results of principal component analysis showing the first two principal components (PC1 and PC2) for all tested samples (HC, *n* = 6; Mabc, *n* = 11; Mmass, *n* = 7). **c** Volcano plots representing 579 immune-related genes with the log2-fold change plotted against the negative log10 adjusted *p*-value for the Mabc and Mmass groups compared to the HC groups. Red and blue dots indicate upregulated and downregulated genes compared to the HC group. **d** Results of the top 5 KEGG pathways enriched with the 109 common DEGs of the Mabc and Mmass groups. **e** Heatmap for 28 DEGs that are constituents of the cytokine–cytokine interaction pathway (hsa04060). Nine genes labeled in red indicate that they are upregulated over 10-fold in the Mabc or Mmass group or members of the TNF signaling pathway. An *IL-7* gene labeled in blue indicates the most depressed gene in the Mabc and Mmass groups. The hierarchical clustering of genes was performed with Euclidean distance matrices of Z-scores. HC healthy controls, Mabc *M. abscessus* subsp. *abscessus*, Mmass *M. abscessus* subsp. *massiliense*.

We performed nCounter Human Immunology gene expression assays on PBMCs from the Mabc-infected patients (*n* = 11), the Mmass-infected patients (*n* = 7), and the HCs (*n* = 6) (Fig. 1a, Supplementary Table 2). All subjects were female and of similar ages (*p* = 0.31 by Kruskal–Wallis test). The mean age was 61.1 years for the Mabc-infected patients, 59.4 years for the Mmass-infected patients, and 55.2 years for the HCs.

To assess transcriptome similarity among 23 datasets, we performed PCA. The HCs were well separated and clustered, and the other two types of patients were intermingled in the PCA, suggesting that there are few or no significant differences in transcriptome-wide gene expression profiles between the Mabc-infected and Mmass-infected patients, but these profiles are clearly distinct from the transcriptome of the HCs (Fig. 1b). Next, we identified 143 and 151 differentially expressed genes (DEGs) in the Mabc and Mmass samples compared to the HC samples, respectively, using both *t*-tests and fold change (FC) analysis (FDR < 0.05 and FC > 2) (Fig. 1c). Of the 109 commonly identified DEGs, 28 DEGs were mainly involved in a cytokine–cytokine receptor interaction, which is the most significantly enriched pathway (FDR < 2.06 × 10^−16^) using KEGG (Kyoto Encyclopedia of Genes and Genomes) (Fig. 1d), and almost three-quarters (21 out of 28) of the genes were upregulated in the NTM-infected patients. Notably, nine genes [C-C motif chemokine ligand (CCL) 2, *CCL7, CCL20, CCL22, CCL24*, C-X-C motif chemokine ligand (CXCL) 2, *CCL24, IL1B, TNF*, and *IL-6*] related to immune responses and inflammation were persistently upregulated (over 10-fold) under NTM-infected conditions (Fig. 1e). In addition, *IL-7*, a crucial cytokine for T-helper 1 responses^23–26^, was the most downregulated gene, with an ~6-fold decrease in both types of patients.

### Upregulation of proinflammatory cytokines/chemokines in PBMCs from the patients compared with those from the HCs

**Fig. 2:**
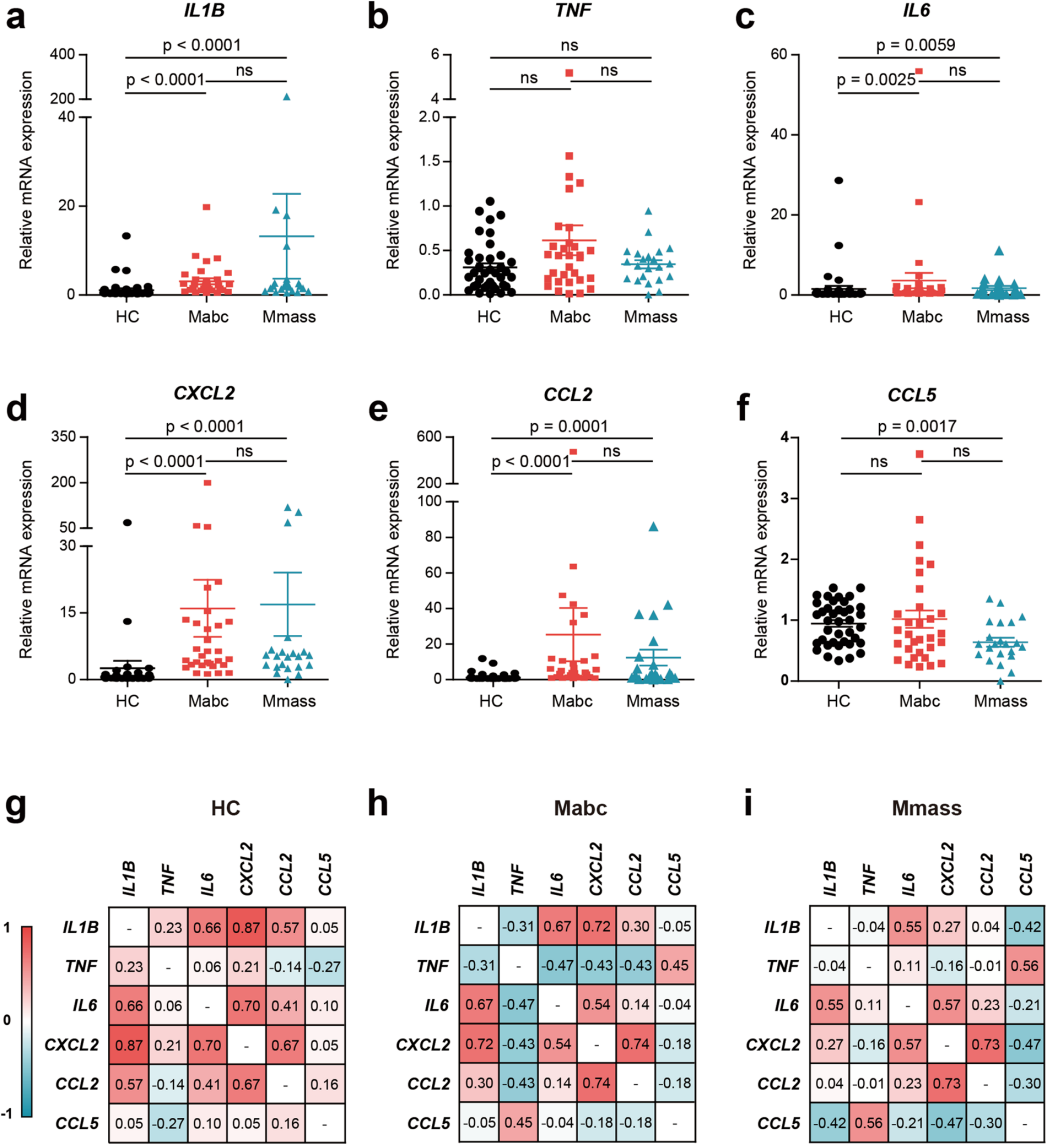
qRT-PCR analysis of cytokine/chemokine profiles in PBMCs between the NTM patients and the HCs. **a**–**f** Dot-plot graph depicting cytokine/chemokine genes that are differentially expressed between the NTM patients and the HCs. Human PBMCs were isolated from the HCs (*n* = 39) and the NTM patients (Mabc, *n* = 31; Mmass, *n* = 22), and cytokine/chemokine mRNA levels were determined using quantitative real-time PCR analysis (**a** *IL1B*; **b** *TNF*; **c** *IL6*; **d** *CXCL2*; **e** *CCL2*; **f** *CCL5*). **g**–**i** Correlation matrix between cytokine/chemokine variables from the HCs (**g**), Mabc-infected patients (**h**), and Mmass-infected patients (**i**). A correlation matrix showed correlation coefficients for a combination of 6 cytokine/chemokine variables for all subjects in this study. ns, not significant. Mann–Whitney U test. Data are the combined results (mean ± SEM) from three or four independent experiments performed in duplicate. HC healthy controls, Mabc *M. abscessus* subsp. *abscessus*, Mmass *M. abscessus* subsp. *massiliense*.

To validate the increased expression of cytokines/chemokines, we performed qRT-PCR of PBMCs from the expanded population (Mabc, *n* = 31; Mmass, *n* = 22; HC, *n* = 39). The mRNA levels of *IL-1β*, *IL-6*, *CXCL2*, and *CCL2* were significantly increased in PBMCs from the Mabc-infected and Mmass-infected patients compared to those from the HCs (Fig. 2a, c–e). The *TNF* mRNA level was not significantly increased in PBMCs from the Mabc-infected and Mmass-infected patients compared to those from the HCs (Fig. 2b). There were no significant differences in the levels of cytokines/chemokines (*IL-1β*, *TNF*, *IL-6*, *CXCL2*, and *CCL2*) between the Mabc-infected and Mmass-infected patients. Interestingly, the *CCL5* mRNA levels were significantly downregulated in PBMCs from the Mmass-infected patients compared with those from the HCs (Fig. 2f). In addition, the mRNA levels of *CCL7*, *CCL20*, and *CCL24*, but not *CCL22*, were significantly increased in the patients infected with Mabc or Mmass compared with the HCs (Supplementary Fig. 2).

We then investigated the interrelationships among different cytokines/chemokines in each group (Fig. 2g–i). In the HCs, there was a significant positive correlation among *IL-1β*, *IL-6*, *CXCL2*, and *CCL2*, whereas there was a negative correlation between *TNF* and *CCL5* (Fig. 2g). In the Mabc-infected patients, there was a strong positive correlation between *IL-6* and *CXCL2* (*n* = 31, *r* = 0.543, *p* = 0.002), *CXCL2* and *IL-1β* (*n* = 31, *r* = 0.723, *p* < 0.001), *CXCL2* and *CCL2* (*n* = 31, *r* = 0.736, *p* < 0.001), and *IL-1β* and *IL-6* (*n* = 31, *r* = 0.670, *p* < 0.001). However, the *TNF* levels were negatively correlated with *IL-1β*, *IL-6*, *CXCL2*, and *CCL2* but not *CCL5* (positive correlation; *n* = 31, *r* = 0.446, *p* = 0.012) (Fig. 2h). In the Mmass-infected patients, there was a strong correlation between *IL-6* and *IL-1β* (*n* = 22, *r* = 0.554, *p* = 0.007), *IL-6* and *CXCL2* (*n* = 22, *r* = 0.570, *p* = 0.006), and *CXCL2* and *CCL2* (*n* = 22, *r* = 0.733, *p* < 0.001). Similar to the findings observed in the Mabc-infected patients, the *TNF* levels showed a strong positive correlation with *CCL5* in the Mmass-infected patients (*n* = 22, *r* = 0.560, *p* = 0.007) (Fig. 2i). These data suggest an altered relationship of *TNF* with other proinflammatory cytokines/chemokines in the patients.

We further investigated the relationship between cytokine/chemokine levels and various clinical parameter(s). Each cytokine/chemokine had a strong positive/negative correlation with certain clinical parameter(s) (Supplementary Fig. 3). Notably, in the Mmass-infected patients, the *CCL5* level had a strong positive correlation with serum albumin levels (*n* = 22, *r* = 0.450, *p* = 0.036), whereas *CXCL2* had a negative correlation with serum albumin levels (*n* = 22, *r* = 0.418, *p* = 0.053). These results suggested that the Mabc-infected and Mmass-infected patients’ peripheral immune cells have a distinct set of upregulated proinflammatory cytokines/chemokines, which might be associated with the altered regulation of specific clinical parameter(s).

### The *miR-144-3p* level is upregulated in PBMCs from the patients

**Fig. 3:**
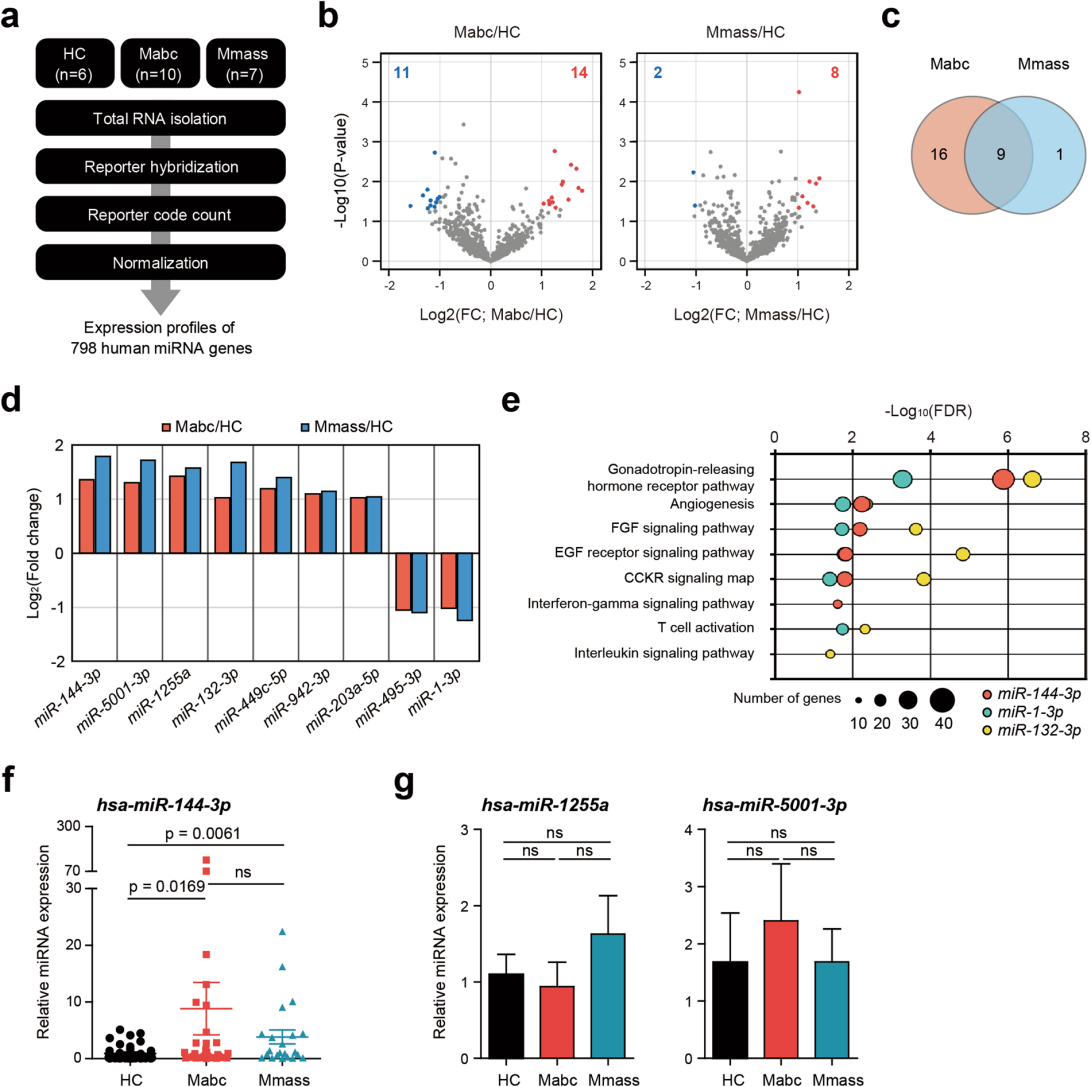
Expression of *miR-144-3p*, identified by miRNA transcriptome analysis, is upregulated in PBMCs from the NTM patients. **a** Schematic diagram of the miRNA transcriptome analysis. **b** Volcano plots representing 798 human miRNA genes with the log2-fold change (FC) plotted against the negative log10 adjusted *p*-value for the Mabc and Mmass groups compared to the HC group. Red and blue dots indicate upregulated and downregulated miRNA genes compared to the HCs. **c** Venn diagram showing that 9 miRNAs were changed in both patient groups. **d** Log2-fold changes of 9 common miRNAs of the Mabc and Mmass groups compared to the HC groups. **e** Results of the Panther pathway enrichment test for the miRNA target genes predicted by TargetScan7.1. **f** Dot-plot graph depicting *miR-144-3p*, which is differentially expressed between the NTM patients and the HCs. Human PBMCs were isolated from the HCs (*n* = 39) and NTM patients (Mabc, *n* = 31; Mmass, *n* = 22). Quantitative real-time PCR analysis of *miR-144-3p* expression. **g** Human PBMCs were isolated from the HCs (*n* = 17) and the NTM patients (Mabc, *n* = 14; Mmass, *n* = 14), and the expression of *miR-1255a* or *miR-5001-3p* was measured by qRT-PCR. ns, not significant; HC, healthy controls; Mabc, patients infected with Mabc; Mmass, patients infected with Mmass. Mann–Whitney U test. Values represent the mean (±SEM) from three independent experiments performed in triplicate (**f**, **g**). HC healthy controls, Mabc *M. abscessus* subsp. *abscessus*, Mmass *M. abscessus* subsp. *massiliense*.

To evaluate the mechanisms underlying the altered proinflammatory cytokine/chemokine expression in the patients, we performed an nCounter Human miRNA Expression Assay of PBMCs and evaluated the miRNA expression levels in the same 23 nCounter samples (Fig. 3a, Supplementary Table 3). We identified 25 (14 upregulated and 11 downregulated) and 10 (8 upregulated and 2 downregulated) miRNAs that were differentially expressed (FDR < 0.05 and fold change >2) in the Mabc-infected and Mmass-infected patients, respectively, compared to the HCs (Fig. 3b). Among them, nine (7 upregulated and 2 downregulated) miRNAs had the same expression pattern (Fig. 3c, d). Next, to examine whether these differentially expressed miRNAs are capable of regulating proinflammatory response genes, we screened for potential miRNA target genes using the TargetScan database and then identified significantly (FDR < 5%) enriched pathways for the predicted target genes. We found 11, 9, and 23 significant PANTHER pathways in the target genes of *miR-144-3p*, *miR-1-3p*, and *miR-132-3p*, respectively (Supplementary Table 4). There were five shared pathways (gonadotropin-releasing hormone receptor pathway; P06664, angiogenesis; P00005, FGF signaling pathway; P00021, EGF receptor signaling pathway; P00018, and CCKR signaling map; P06959), with three immune response-related pathways (interferon-gamma signaling pathway; P00035, T-cell activation; P00053, and interleukin signaling pathway; P00036) (Fig. 3e).

We selected three miRNAs that showed the greatest difference in expression between the patients and the HCs and confirmed that the *miR-144-3p* level was significantly higher in the Mabc-infected and Mmass-infected patients than in the HCs (Fig. 3f; *p* < 0.001). The levels of the other two miRNAs (*hsa-miR-1255a* and *hsa-miR-5001-3p*) were not significantly different (Fig. 3g). We also examined whether the severity of the condition and the initiation of antibiotic treatment affected the expression of *miR-144-3p*. When the severity was divided according to the BACES score^27^ (Supplementary Fig. 4a) and the corresponding expression levels of *miR-144-3p* were compared, no significant difference was found (Supplementary Fig. 4b; *p* = 0.905). The level of *miR-144-3p* also did not show any significant difference depending on whether antibiotic treatment was started (Supplementary Fig. 4c; *p* = 0.215). Altogether, these data suggested that *miR-144-3p* is a signature miRNA of PBMCs from patients with Mabc or Mmass infection.

### *MiR-144-3p* overexpression increases proinflammatory cytokine/chemokine expression in human macrophages

**Fig. 4:**
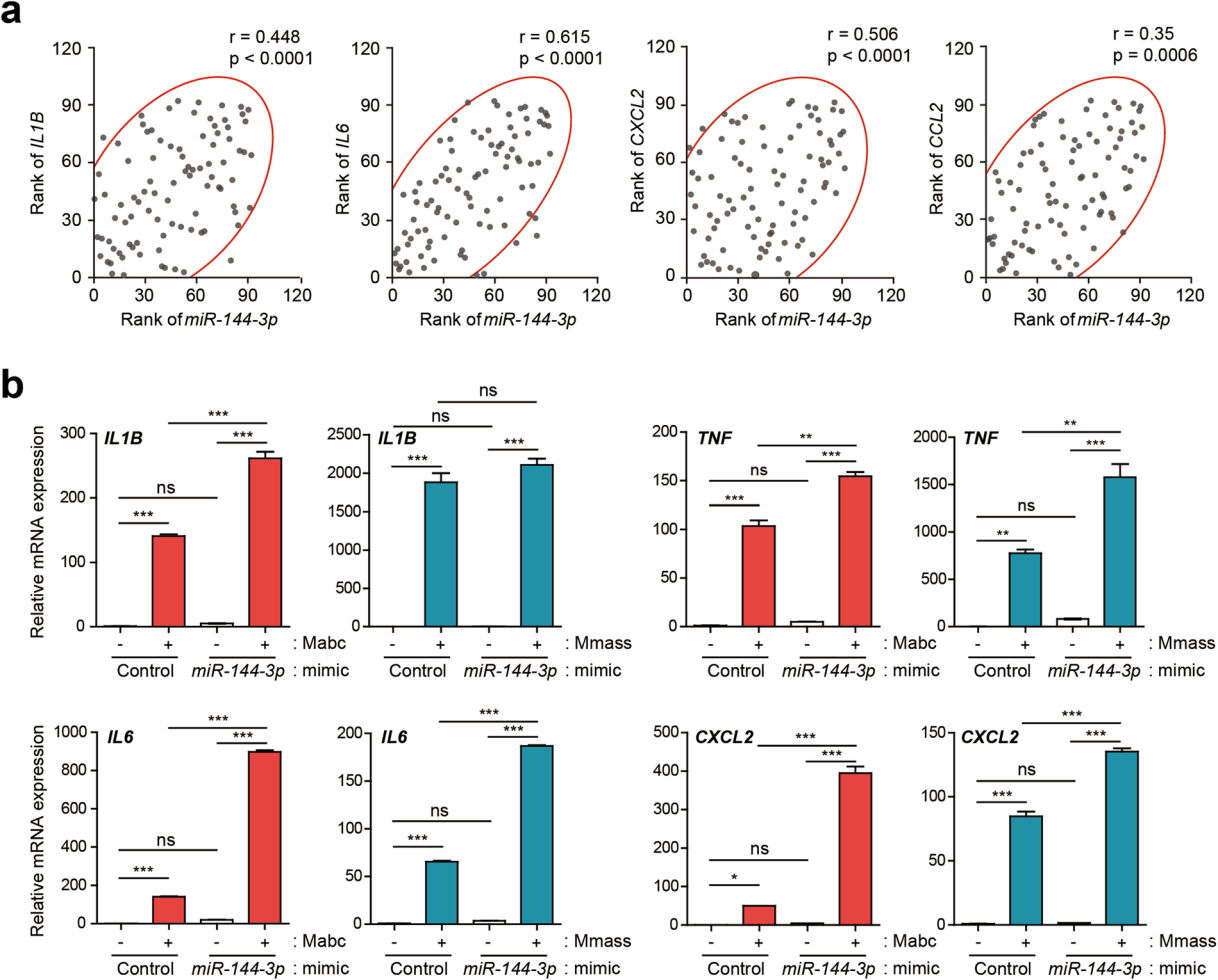
Overexpression of *miR-144-3p* upregulates proinflammatory cytokine/chemokine expression in human MDMs. **a** Scatter plots with 90% density ellipses showing the rank of *miR-144-3p* plotted against *IL1B*, *IL6*, *CXCL2*, and *CCL2* for all subjects (HC, *n* = 39; Mabc, *n* = 31; Mmass, *n* = 22). Spearman’s rank correlation determined the coefficient (*r*) and significance. **b** Human primary MDMs were transfected with mimic negative control (Control) or *miR-144-3p* mimic (10 nM) for 24 h and then infected with or without Mabc or Mmass (MOI = 2, for each; for 3 h). Quantitative real-time PCR analysis of *IL1B*, *IL6*, *TNF*, and *CXCL2* expression. *p < 0.05, **p < 0.01, and ***p < 0.001. Spearman correlation (**a**); one-way ANOVA (**b**). Values represent the mean (±SD) from three independent experiments performed in duplicate (**b**). HC healthy controls, Mabc *M. abscessus* subsp. *abscessus*, Mmass *M. abscessus* subsp. *massiliense*.

*Hsa-mir-144-3p* reportedly plays a proinflammatory role in atopic dermatitis^28^. We thus assessed the relationship between the *miR-144-3p* level and inflammatory cytokines/chemokines. The *miR-144-3p* level was significantly positively correlated with those of *IL-1β* (*n* = 92, *r* = 0.448, *p* < 0.0001), *IL-6* (*n* = 92, *r* = 0.615, *p* < 0.0001), *CXCL2* (*n* = 92, *r* = 0.506, *p* < 0.0001), and *CCL2* (*n* = 92, *r* = 0.35, *p* = 0.0006) (Fig. 4a).

We next transfected human primary MDMs with a *miR-144-3p* mimic and examined the mRNA levels of *IL-1β*, *IL-6*, *TNF*, and *CXCL2*. The overexpression of *miR-144-3p* resulted in significant upregulation of the levels of the four proinflammatory cytokines/chemokines in MDMs infected with Mabc (Fig. 4b). In Mmass infection, *miR-144-3p* overexpression led to increased *IL-6*, *TNF*, and *CXCL2* levels but did not affect the level of *IL-1β* (Fig. 4b). The transfection efficiency of *miR-144-3p* is shown in Supplementary Figure 5. Therefore, *miR-144-3p* induces the mRNA synthesis of proinflammatory cytokines/chemokines in human MDMs following Mabc or Mmass infection.

### The levels of the TLR negative regulators *SARM1* and *TNIP3* are downregulated in PBMCs from patients

**Fig. 5:**
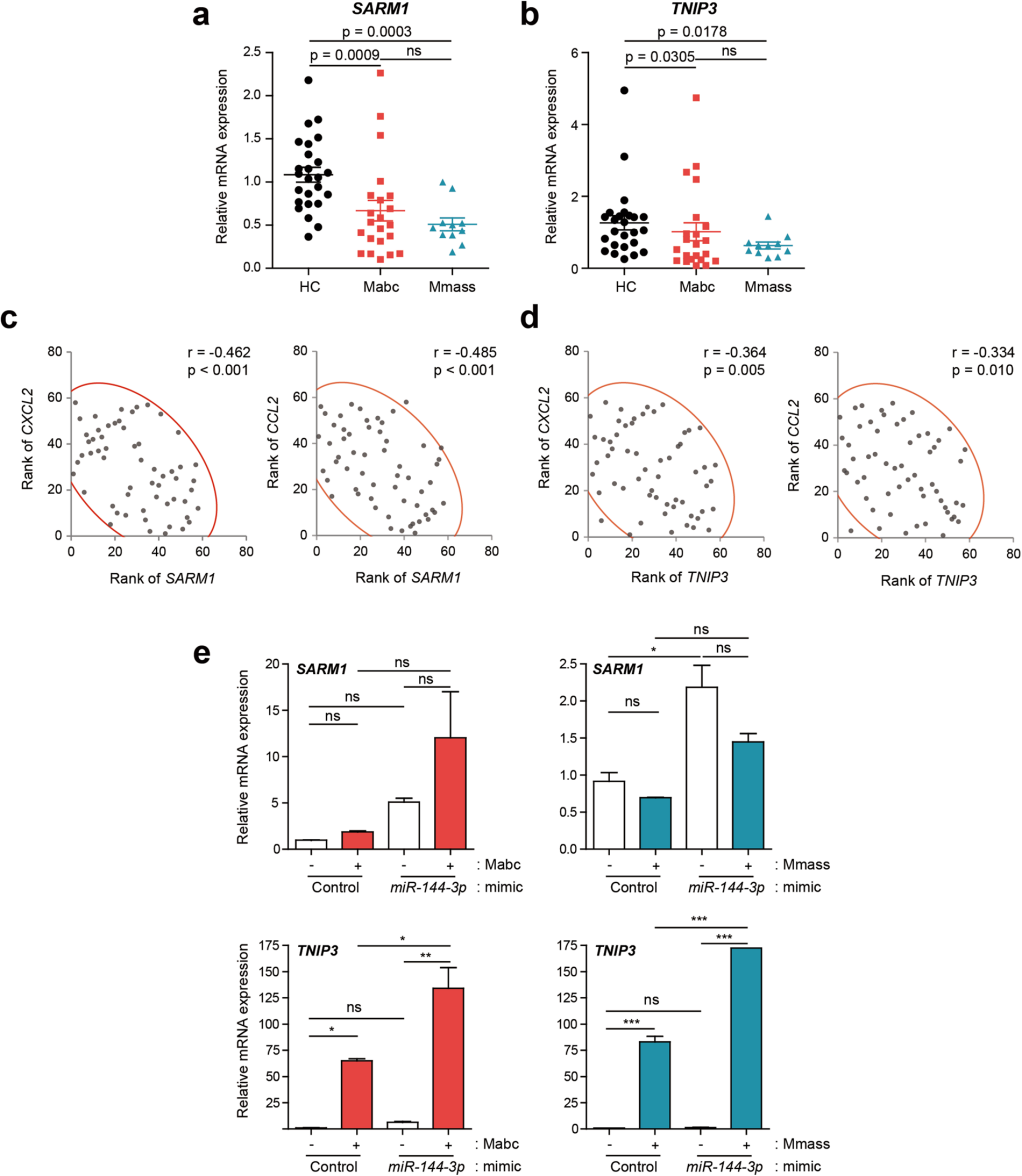
Transcriptional analysis of *SARM1* and *TNIP3* in PBMCs between the NTM patients and the HCs. **a**, **b** Dot-plot graph depicting cytokine/chemokine genes that are differentially expressed between NTM patients and HCs. Human PBMCs were isolated from HC (*n* = 25) and NTM patients (Mabc, *n* = 22; Mmass, *n* = 11) and *SARM1* (**a**) and *TNIP3* (**b**) mRNAs were determined using quantitative real-time PCR analysis. Mann–Whitney U test. **c, d** Correlation analysis between *SARM1* and *CXCL2*/*CCL2* (**c**) and *TNIP3* and *CXCL2*/*CCL2* (**d**). **e** Human primary MDMs were transfected with mimic negative control (Control) or *miR-144-3p* mimic (10 nM) for 24 h and then infected with or without Mabc or Mmass (MOI = 2, for each; for 3 h). Quantitative real-time PCR analysis of *TNIP3* and *SARM1* expression. *p < 0.05, **p < 0.01, and ***p < 0.001. Spearman correlation (**c**, **d**); one-way ANOVA (**e**). Data are representative of three or four independent experiments performed in duplicate. HC healthy controls, Mabc *M. abscessus* subsp. *abscessus*, Mmass *M. abscessus* subsp. *massiliense*.

To identify the targets of *miR-144-3p* in modulating host inflammatory responses, we used the computational target prediction tools miRanda (http://www.microrna.org/microrna/home.do), TargetScan, and DIANA-microT (http://diana.cslab.ece.ntua.gr/microT/). Bioinformatic analysis identified two putative TLR negative regulator genes encoding SARM1^14^ and TNIP3^15^ as *miR-144-3p* targets. We then examined the mRNA expression levels in PBMCs by qRT-PCR analysis and found that both *SARM1* and *TNIP3* levels were significantly downregulated in both the Mabc (*n* = 22) and Mmass (*n* = 11) groups (Fig. 5a, b). Furthermore, the *SARM1* levels were negatively correlated with the *CXCL2* and *CCL2* levels in all tested subjects. Similar to these results, the *TNIP3* mRNA levels showed an inverse correlation with the *CXCL2* and *CCL2* levels in all tested subjects (Fig. 5c, d).

To further determine whether *miR-144-3p* is involved in the downregulation of both *SARM1* and *TNIP3*, we overexpressed human MDMs with a *miR-144-3p* mimic and assessed the mRNA expression of *SARM1* and *TNIP3* after Mabc and Mmass infection. The data showed that the overexpression of *miR-144-3p* failed to downregulate and significantly upregulated the mRNA expression of *TNIP3* and *SARM1* in human MDMs before and after infection with Mabc or Mmass (Fig. 5e). These data revealed that both *SARM1* and *TNIP3* are not putative targets for *miR-144-3p*. Nevertheless, these data suggest that the depressed levels of the TLR negative regulators *SARM1* and *TNIP3* play a role in the upregulated inflammatory responses in NTM patients.

### *MiR-144-3p* is upregulated in a mouse model in vitro and in vivo

**Fig. 6:**
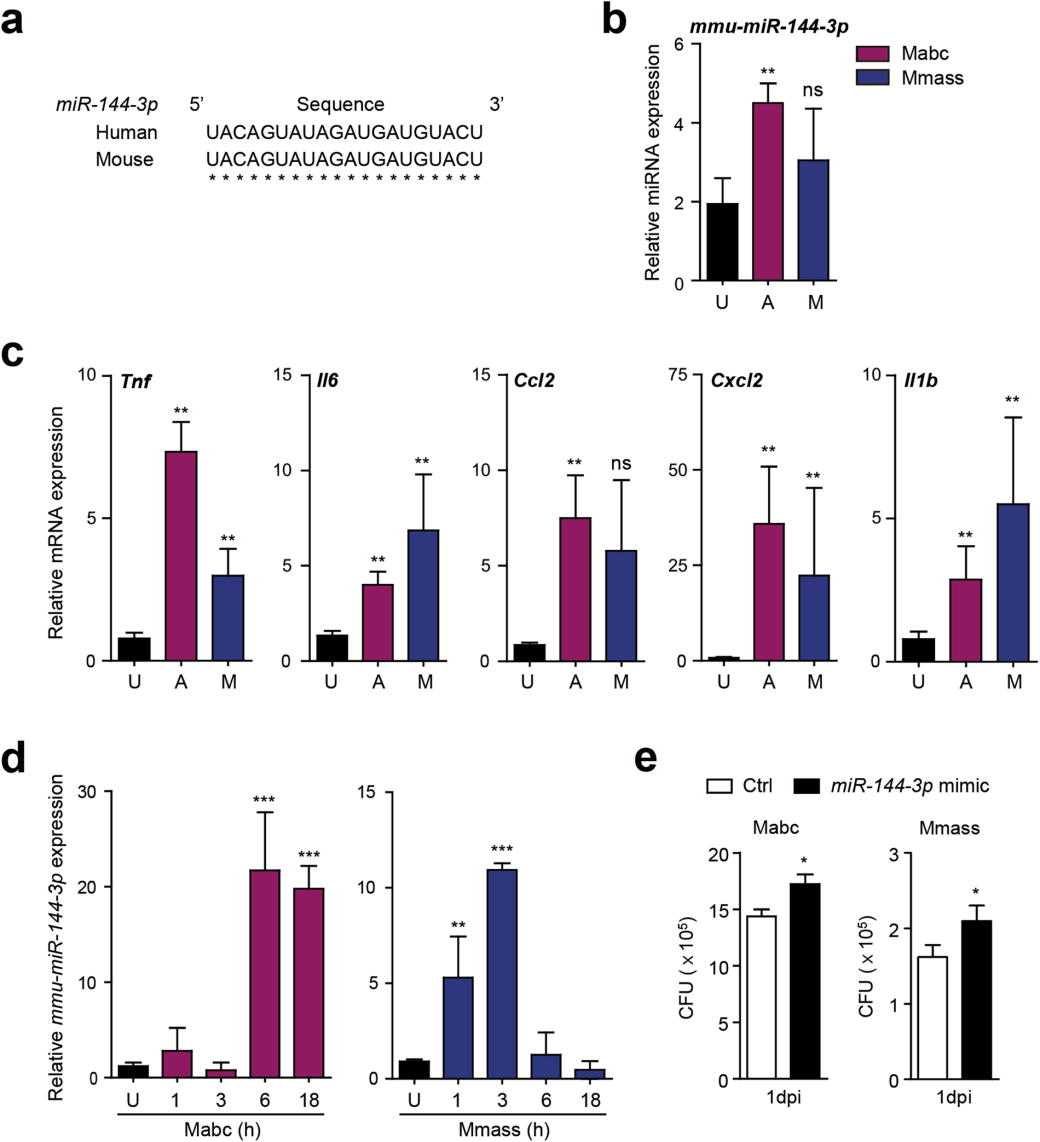
*MiR-144-3p* levels are upregulated in vitro and in vivo and associated with intracellular growth of Mabc and Mmass. **a** Alignment of *miR-144-3p* sequences from human and mouse genomes (hsa, *Homo sapiens*; mmu, *Mus musculus*). **b**, **c** C57BL/6 mice were infected intranasally with Mabc (1 × 10^7^ CFU) or Mmass (1 × 10^7^ CFU) and monitored at 7 days post-infection (dpi). Quantitative real-time PCR analysis of *miR-144-3p* (**b**) and *Tnf*, *Il6*, *Ccl2*, *Cxcl2*, and *Il1b* (**c**) in the lung tissues from infected mice (*n* = 4, for each group). **d** Murine BMDMs were infected with Mabc or Mmass (MOI = 3, 2 h) and cultured at the indicated times. Quantitative RT-PCR analysis of *mmu-miR-144-3p*. **e** Murine BMDMs were transfected with mimic negative control (Ctrl) or *mmu-miR-144-3p* mimic (10 nM) for 24 h. Cells were infected with Mabc or Mmass (MOI = 1, for each), followed by CFU assays for determination of intracellular survival of bacteria after 24 h. *p < 0.05, **p < 0.01, ***p < 0.001. ns not significant, U uninfected, A Mabc, MMmass. Mann–Whitney U test (**b**, **c**, and **e**); one-way ANOVA (**d**). Values represent the mean (±SD) from at least four independent experiments performed in triplicate (**b**–**d**). Data are the combined results (mean ± SEM) from three independent experiments (**e**). Mabc *M. abscessus* subsp. *abscessus*, Mmass *M. abscessus* subsp. *massiliense*.

Because the *miR-144-3p* sequence is identical in humans and mice (Fig. 6a), we next evaluated the effects of Mabc and Mmass infection on the *miR-144-3p* level in vivo and in vitro. To this end, C57BL/6 mice were infected with Mabc or Mmass for 3 days, and the lungs were harvested for determination of the miRNA and mRNA levels. The *mmu-miR-144-3p* level was significantly upregulated in the mouse lung after Mabc or Mmass infection (Fig. 6b), as were the levels of the five proinflammatory cytokines and chemokines (Fig. 6c).

We also determined the miRNA and mRNA levels in murine bone marrow-derived macrophages (BMDMs) after Mabc or Mmass infection. Although the peak time point differed between the Mabc and Mmass groups, either strain significantly upregulated the *mmu-miR-144-3p* level in BMDMs (Fig. 6d). In addition, Mabc infection robustly increased the mRNA levels of *TNF*, *IL-1β*, and *CXCL2* in BMDMs in a time-dependent manner (Supplementary Fig. 6a). Interestingly, Mmass infection significantly increased the *TNF*, *IL-1β*, and *CXCL2* levels in BMDMs after 18 h of infection (Supplementary Fig. 6b). Furthermore, *mmu-miR-144-3p* overexpression led to a slight but significant upregulation of intracellular Mabc and Mmass growth in BMDMs (Fig. 6e). Therefore, the *mmu-miR-144-3p* level is upregulated by Mabc and Mmass infection in vitro and in vivo and contributes to favoring the intracellular growth of Mabc and Mmass.

## Discussion

Compared with *Mycobacterium tuberculosis* (TB), the causal pathogen of tuberculosis, less is known about the host immunologic factors important during NTM infection, particularly with rapidly growing *M. abscessus*, such as Mabc, Mmass, and *M. abscessus* subsp. *bolletii*^29,30^*. M. abscessus* causes opportunistic pulmonary infections in high-risk patients, including those with cystic fibrosis and bronchiectasis^1,29,31,32^, and can cause pulmonary, skin, and soft tissue infections even in immunocompetent subjects^1,29,31,32^. Importantly, it is emerging as a clinically important strain with an increasing prevalence of antibiotic resistance^33^, although the response rates to antibiotic therapy are somewhat different among subspecies^34^. A mechanistic understanding of the immune profile of NTM disease is needed to develop host-directed therapeutics; however, the key immune biomarkers for patients with Mabc and Mmass infection remain ill-defined.

A bioinformatics analysis yielded a large set of proinflammatory cytokines/chemokines elevated in patients and identified several candidate genes for validation in the expanded patient group. The *IL-1β*, *IL-6*, *CXCL2*, and *CCL2* mRNA levels were upregulated in the Mabc-infected and Mmass-infected patients compared with the HCs. Our data partly correlate with recent findings that both *M. abscessus* and *Mycolicibacterium smegmatis* induce high chemokine levels in human monocytic THP-1 cells^35^. Notably, there was a strong positive correlation among proinflammatory cytokines/chemokines, except *TNF*, in *M. abscessus*-infected patients. Previous studies using animal models showed that *M. abscessus*-induced *TNF* is crucial for protective immunity^36,37^ or disease pathology depending on the strain^38^. Despite this, little is known about the expression of *TNF* in PBMCs between patients infected with Mabc and Mmass. Our data showed that the *TNF* levels did not differ among PBMCs from all groups. Previous data showed that *TNF* expression was lower in cultured human monocytes from NTM patients infected with *Mycobacterium avium* or *M. abscessus*^39^. In addition, we found that the chemokine *CCL5* was depressed in the Mmass-infected patients. *CCL5*, also known as RANTES (regulated on activation, normal T cell expressed and secreted), is important for T-cell migration^40^. It was shown that depressed *CCL5* in a pleural site of tuberculous pleurisy patients is associated with poor antigenic responses at the disease sites^41^. Together, *CCL5* levels may show potential immunobiologic relevance in anti-NTM immunity, particularly against Mmass infection. Interestingly, *CCL5* levels showed a strong positive correlation with serum albumin levels in the Mmass-infected patients. Human NTM diseases are extremely diverse, particularly in clinical and microbiological spectrum, geographic distribution, etc^31^. Therefore, the characterization of an organized pattern between immune and clinical phenotypes will be necessary for effective therapeutic interventions against human NTM infections.

MiRNAs are short noncoding RNAs and post-transcriptional regulators of a variety of biological responses, including immune responses and autophagy during *M*. *tuberculosis* infection^42,43^. Although a recent study has shown that several miRNAs can be used as potential biomarkers in NTM diseases^44^, the profile and role of miRNAs in human *M. abscessus* infection are unclear. Importantly, transcriptome analysis and qRT-PCR validation showed that the *miR-144-3p* level was markedly increased in the Mabc and Mmass patients compared with the HCs. In addition, *miR-144-3p* was strongly correlated with the expression of several proinflammatory cytokines and chemokines, suggesting its relevance to inflammatory responses during Mabc and Mmass infection. Importantly, overexpression of *miR-144-3p* led to a substantial increase in the expression of proinflammatory cytokines/chemokines and the intracellular growth of Mabc and Mmass. The data partly corroborate previous studies showing that BCG infection reportedly increases *miR-144-3p* to inhibit autophagy and favor intracellular growth in macrophages^45^. Future studies are warranted to elucidate the mechanisms by which *miR-144-3p* contributes to promoting intracellular mycobacterial growth in the context of NTM infection.

To date, the reported levels of *miR-144-3p* in TB patients are heterogeneous^46–49^, and there have not been reports of *miR-144-3p* in NTM infection. Given the finding that the *miR-144-3p* level is increased by NTM infection in PBMCs and mouse models in vitro and in vivo, we propose *miR-144-3p* as a potential biomarker of Mabc and Mmass infection. Importantly, overexpression of *miR-144-3p* in human macrophages increased proinflammatory cytokine/chemokine generation in response to Mabc or Mmass infection, indicating that *miR-144-3p* promotes pathological inflammation. *Hsa-mir-144-3p* is reportedly involved in proinflammatory responses in atopic dermatitis^28^. However, previous reports^28^ and our studies did not identify the target(s) of *miR-144-3p* in the context of modulation of inflammatory cytokines/chemokines. By bioinformatics analysis, we investigated two TLR negative regulators, *TNIP3*^15^ and *SARM1*^14^, and their relationships with *miR-144-3p*, although they were not putative targets of *miR-144-3p*. Notably, the *TNIP3* and *SARM1* levels were significantly decreased in PBMCs from the Mabc-infected and Mmass-infected patients and showed a negative correlation with *CXCL2* and *CCL2*. These data strongly indicate that depressed *TNIP3* and *SARM1* levels may be linked to excessive production of inflammatory chemokines in the context of *M. abscessus* infection. A deeper understanding of the key protective/pathological factors in human NTM diseases will facilitate the development of novel host-directed therapeutics for *M. abscessus* infection, which is often refractory to currently used antibiotics.

## Supplementary information

Supplemental Material File

## Data Availability

The data sets used or analyzed during the current study are available from the corresponding author on reasonable request.

## References

[1] Cowman S, van Ingen J, Griffith DE, Loebinger MR (2019). Non-tuberculous mycobacterial pulmonary disease. Eur. Respir J..

[2] Koh WJ, Stout JE, Yew WW (2014). Advances in the management of pulmonary disease due to *Mycobacterium abscessus* complex. Int. J. Tuberc. Lung Dis..

[3] Diel R (2017). Microbiological and clinical outcomes of treating non-mycobacterium avium complex nontuberculous mycobacterial pulmonary disease: a systematic review and meta-analysis. Chest.

[4] Lee MR (2015). *Mycobacterium abscessus* complex infections in humans. Emerg. Infect. Dis..

[5] Mougari F (2016). Infections caused by *Mycobacterium abscessus*: epidemiology, diagnostic tools and treatment. Expert Rev. Anti Infect. Ther..

[6] Nowag A, Platten M, Plum G, Hartmann P (2017). Nontuberculous mycobacterial infections. Z. Rheumatol..

[7] Brode SK (2015). Increased risk of mycobacterial infections associated with anti-rheumatic medications. Thorax.

[8] Matsuyama M (2018). Transcriptional response of respiratory epithelium to nontuberculous mycobacteria. Am. J. Respir. Cell Mol. Biol..

[9] Qian C, Liu J, Cao X (2014). Innate signaling in the inflammatory immune disorders. Cytokine Growth Factor Rev..

[10] Muralidharan S, Mandrekar P (2013). Cellular stress response and innate immune signaling: integrating pathways in host defense and inflammation. J. Leukoc. Biol..

[11] Aguilar C, Mano M, Eulalio A (2019). MicroRNAs at the host-bacteria interface: host defense or bacterial offense. Trends Microbiol..

[12] Lyu L (2019). Small RNA profiles of serum exosomes derived from individuals with latent and active tuberculosis. Front. Microbiol..

[13] Sabir N (2018). miRNAs in tuberculosis: new avenues for diagnosis and host-directed therapy. Front. Microbiol..

[14] Carlsson E, Ding JL, Byrne B (2016). SARM modulates MyD88-mediated TLR activation through BB-loop dependent TIR-TIR interactions. Biochim. Biophys. Acta.

[15] Wullaert A (2007). LIND/ABIN-3 is a novel lipopolysaccharide-inducible inhibitor of NF-kappaB activation. J. Biol. Chem..

[16] Kim JK (2017). MIR144* inhibits antimicrobial responses against *Mycobacterium tuberculosis* in human monocytes and macrophages by targeting the autophagy protein DRAM2. Autophagy.

[17] Kim JK (2018). GABAergic signaling linked to autophagy enhances host protection against intracellular bacterial infections. Nat. Commun..

[18] Kim YS (2020). The peroxisome proliferator-activated receptor alpha-agonist gemfibrozil promotes defense against *Mycobacterium abscessu*s Infections. Cells.

[19] R Core Team. R: A language and environment for statistical computing. R Foundation for Statistical Computing, Vienna, Austria. (2013). http://www.R-project.org/.

[20] Shin C (2010). Expanding the microRNA targeting code: functional sites with centered pairing. Mol. Cell.

[21] Kanehisa M, Goto S (2000). KEGG: kyoto encyclopedia of genes and genomes. Nucleic Acids Res..

[22] Mi H, Muruganujan A, Thomas PD (2013). PANTHER in 2013: modeling the evolution of gene function, and other gene attributes, in the context of phylogenetic trees. Nucleic Acids Res..

[23] Borger P, Kauffman HF, Postma DS, Vellenga E (1996). IL-7 differentially modulates the expression of IFN-gamma and IL-4 in activated human T lymphocytes by transcriptional and post-transcriptional mechanisms. J. Immunol..

[24] Arbelaez CA (2015). IL-7/IL-7 receptor signaling differentially affects effector CD4+ T cell subsets involved in experimental autoimmune encephalomyelitis. J. Immunol..

[25] Natale MA (2018). Trypanosoma cruzi-specific IFN-gamma-producing cells in chronic Chagas disease associate with a functional IL-7/IL-7R axis. PLoS Negl. Trop. Dis..

[26] Francois, B.et al. Interleukin-7 restores lymphocytes in septic shock: the IRIS-7 randomized clinical trial. JCI Insight **3**, e98960 (2018). 10.1172/jci.insight.98960. 10.1172/jci.insight.98960 PMC5922293 29515037

[27] Kim, H. J. et al. BACES score for predicting mortality in nontuberculous mycobacterial pulmonary disease. *Am. J. Respir. Crit. Care Med*. 2020. 10.1164/rccm.202004-1418OC. 10.1164/rccm.202004-1418OC 32721164

[28] Dissanayake E (2019). Hsa-mir-144-3p expression is increased in umbilical cord serum of infants with atopic dermatitis. J. Allergy Clin. Immunol..

[29] Lopeman RC, Harrison J, Desai M, Cox JAG (2019). Mycobacterium abscessus: environmental bacterium turned clinical nightmare. Microorganisms.

[30] Bryant JM (2013). Whole-genome sequencing to identify transmission of *Mycobacterium abscessus* between patients with cystic fibrosis: a retrospective cohort study. Lancet.

[31] Swenson C, Zerbe CS, Fennelly K (2018). Host variability in NTM disease: implications for research needs. Front Microbiol..

[32] Koh, W. J. Nontuberculous mycobacteria—overview. *Microbiol. Spectr.* **5**, (2017). 10.1128/microbiolspec.TNMI7-0024-2016. 10.1128/microbiolspec.tnmi7-0024-2016 PMC11687458 28128073

[33] Kwon YS, Daley CL, Koh WJ (2019). Managing antibiotic resistance in nontuberculous mycobacterial pulmonary disease: challenges and new approaches. Expert Rev. Respir. Med..

[34] Koh WJ (2011). Clinical significance of differentiation of *Mycobacterium massiliense* from *Mycobacterium abscessus*. Am. J. Respir. Crit. Care Med..

[35] Feng Z (2020). Differential responses by human macrophages to infection with *Mycobacterium tuberculosis* and non-tuberculous mycobacteria. Front Microbiol..

[36] Bernut A (2016). *Mycobacterium abscessus*-induced granuloma formation is strictly dependent on TNF signaling and neutrophil trafficking. PLoS Pathog..

[37] Kim JS (2015). Essential engagement of Toll-like receptor 2 in initiation of early protective Th1 response against rough variants of *Mycobacterium abscessus*. Infect. Immun..

[38] Catherinot E (2007). Hypervirulence of a rough variant of the *Mycobacterium abscessus* type strain. Infect. Immun..

[39] Ryu YJ (2007). Impaired expression of Toll-like receptor 2 in nontuberculous mycobacterial lung disease. Eur. Respir. J..

[40] Luther SA, Cyster JG (2001). Chemokines as regulators of T cell differentiation. Nat. Immunol..

[41] Pydi SS (2019). Down regulation of *RANTES* in pleural site is associated with inhibition of antigen specific response in tuberculosis. Tuberculosis.

[42] Rajaram MV, Ni B, Dodd CE, Schlesinger LS (2014). Macrophage immunoregulatory pathways in tuberculosis. Semin. Immunol..

[43] Silwal P, Kim YS, Basu J, Jo EK (2020). The roles of microRNAs in regulation of autophagy during bacterial infection. Semin. Cell Dev. Biol..

[44] Han SA (2020). miRNA expression profiles and potential as biomarkers in nontuberculous mycobacterial pulmonary disease. Sci. Rep..

[45] Guo L (2017). *MicroRNA-144-3p* inhibits autophagy activation and enhances *Bacillus Calmette-Guerin* infection by targeting ATG4a in RAW264.7 macrophage cells. PLoS ONE.

[46] Lv Y (2016). Sputum and serum *microRNA-144* levels in patients with tuberculosis before and after treatment. Int. J. Infect. Dis..

[47] Zhou M (2016). Circulating microRNAs as biomarkers for the early diagnosis of childhood tuberculosis infection. Mol. Med. Rep..

[48] Wu J (2012). Analysis of microRNA expression profiling identifies *miR-155* and *miR-155** as potential diagnostic markers for active tuberculosis: a preliminary study. Hum. Immunol..

[49] Wang C (2011). Comparative miRNA expression profiles in individuals with latent and active tuberculosis. PLoS ONE.

