# Supplementary material for: MiR-144-3p is associated with pathological inflammation in patients infected with Mycobacteroides abscessus
Source: Exp Mol Med. 2021 Jan 20;53(1):136–49. doi: 10.1038/s12276-020-00552-0 (PMC8080579; doi:10.1038/s12276-020-00552-0)
Supplement: Supplementary file 1 — Supplemental Material File [file 12276_2020_552_MOESM1_ESM.pdf]

Supplementary Table 1. Primers used in this study.

Genes	Primer	Sequences
Human		
IL1B	Forward<br>Reverse	5′-CCACAGACCTTCCAGGAGAATG-3′<br>5′-GTGCAGTTCAGTGATCGTACAGG-3′
TNF	Forward<br>Reverse	5′-GCTGCACTTTGGAGTGATCG-3′<br>5′-ACAACATGGGCTACAGGCTT-3′
IL6	Forward<br>Reverse	5′-TGTGAAAGCAGCAAAGAGGCACTG-3′<br>5′-ACAGCTCTGGCTTGTTCCTCACTA-3′
CXCL2	Forward<br>Reverse	5′-CCGAAGTCATAGCCACACTC-3′<br>5′-GGATTTGCCATTTTTCAGCA-3′
CCL2	Forward<br>Reverse	5′-CCCCAGTCACCTGCTGTTAT-3′<br>5′-TGGAATCCTGAACCCACTTC-3′
CCL5	Forward<br>Reverse	5′-CCTGCTGCTTTGCCTACATTGC-3′<br>5′-ACACACTTGGCGGTTCTTTCGG-3′
SARM1	Forward<br>Reverse	5′-AGGTAGCTCTCCTGAACCTCC-3′<br>5′-GTCCCTGGATATTGTCCTCCAG-3′
TNIP3	Forward<br>Reverse	5′-AAACTTCCCAATCCCAGTTGAAC-3′<br>5′-GGGTGGGCAATACATCTGTTTT-3′
CCL7	Forward<br>Reverse	5′-CCCTCACCCTCCAACATGAAA-3′<br>5′-TAGCTCTCCAGCCTCTGCTTA-3′
CCL20	Forward<br>Reverse	5′-GCTCCTGGCTGCTTTGATGT-3′<br>5′-CAGTCAAAGTTGCTTGCTTCTG-3′
CCL22	Forward<br>Reverse	5′-GCGTGGTGTTGCTAACCTTCA-3′<br>5′-GGGGAGCAGCTATAATGGCA-3′
CCL24	Forward<br>Reverse	5′-ACATCATCCCTACGGGCTCT-3′<br>5′-GGTAGCTGACCACTCGGTTC-3′
GAPDH	Forward<br>Reverse	5′-CAAGATCATCAGCAATGCCTCC-3′<br>5′-GGTCATGAGTCCTTCCACGA-3′
Mouse		
Cxcl2	Forward<br>Reverse	5′-CCCTGCCAAGGGTTGACTTC-3′<br>5′-GCAAACTTTTTGACCGCCCT-3′
Ccl2	Forward<br>Reverse	5′-TGACCCCAAGAAGGAATGGG-3′<br>5′-ACCTTAGGGCAGATGCAGTT-3′
Il6	Forward<br>Reverse	5′-TACCACTTCACAAGTCGGAGGC-3′<br>5′-CTGCAAGTGCATCATCGTTGTTC-3′
Il1b	Forward<br>Reverse	5′-TACGGACCCCAAAAGATGA-3′<br>5′-TGCTGCTGCGAGATTTGAAG-3′
Tnf	Forward<br>Reverse	5′-ACGGCATGGATCTCAAAGAC-3′<br>5′-AGATAGCAAATCGGCTGACG-3′
Gapdh	Forward<br>Reverse	5′-AAGATGGTGATGGGCTTCCCG-3′<br>5′-TGGCAAAGTGGAGATTGTTGCC-3′

Supplementary Figure Legends

Supplementary Figure 1. Chest radiographic findings of the patients infected with Mabc and Mmass. The nodular bronchiectatic form of Mabc pulmonary disease in a 71-year old female patient (**a**). The fibrocavitary form of Mmass pulmonary disease in a 43-year old female patient (**b**).

Supplementary Figure 2. Dot-plot graph depicting chemokine genes that are differentially expressed between NTM patients and HC. Human PBMCs were isolated from (HC; n = 12) and NTM patients (Mabc, n = 9; Mmass, n = 11). Quantitative real-time PCR analysis of *Ccl7*, *Ccl20*, *Ccl22*, and *Ccl24* expression. **P < 0.01, ***P < 0.001. ns, not significant. HC, healthy controls; Mabc, patients infected with *M. abscessus*; Mmass, patients infected with *M. massiliense*. Mann-Whitney U test. Values represent means (± SEM) from three independent experiments performed in duplicate.

Supplementary Figure 3. Correlation matrix between immune/clinical variables of NTM patients. A correlation matrix showing correlation coefficients for combination of 4 cytokine/chemokine variables and clinical parameters for all subjects in this study (n = 53; Mabc, n = 31; Mmass, n=22). BMI = body mass index, ESR = erythrocyte sedimentation rate, CRP = C-reactive protein, WBC = white blood cells, Plt = platelets, lymph% = percentage of lymphocytes to total white blood cells counts, mono% = percentage of monocytes to total white blood cells counts, AST = aspartate aminotransferase, ALT = alanine aminotransferase, Alb = albumin.

Supplementary Figure 4. Transfection efficiency of miR144-3p in human MDM. Human MDMs were transfected with vehicle control or *miR144-3p* mimic (10 nM) for 24 h. Expression levels of *miR-144-3p* were determined by quantitative real-time PCR analysis. **P < 0.01, ***P < 0.001. One-way ANOVA. Values represent means (± SD) from three independent experiments performed in duplicate. Mabc, *M. abscessus*.

Supplementary Figure 5. Kinetics of cytokine/chemokine mRNA expression in BMDMs following infection with Mabc or Mmass. Murine BMDMs were infected with Mabc (**a**) or Mmass (**b**; MOI = 3, for each) at the indicated times. Quantitative real-time PCR analysis of *Tnfa*, *Il1b*, and *Cxcl2*. *P < 0.05, **P < 0.01, ***P < 0.001. One-way ANOVA. Values represent means (± SD) from two independent experiments performed in triplicate. Mabc, *M. abscessus*; Mmass, *M. massiliense*.

Supplementary Figure 1

a b

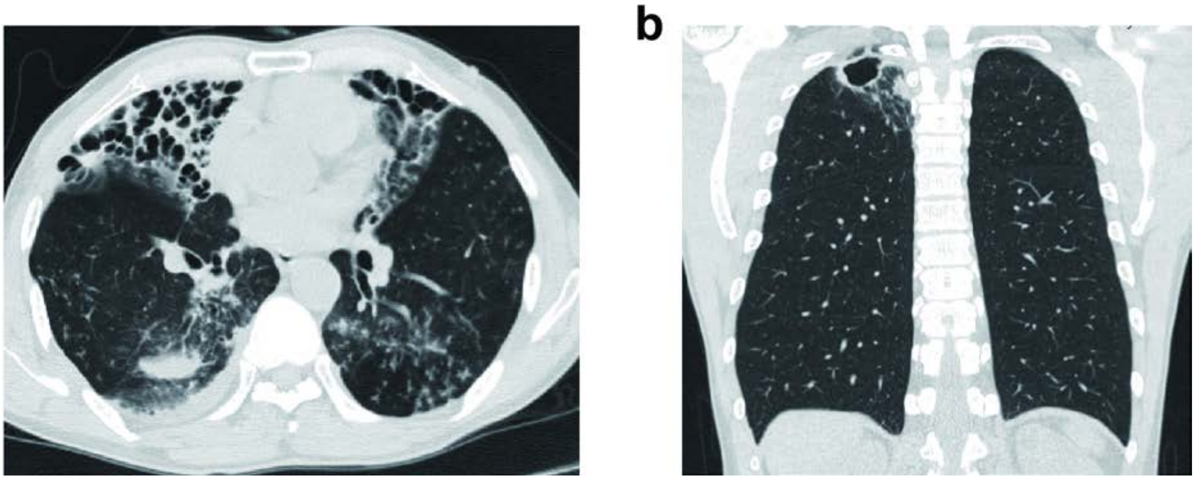

Supplementary Figure 2

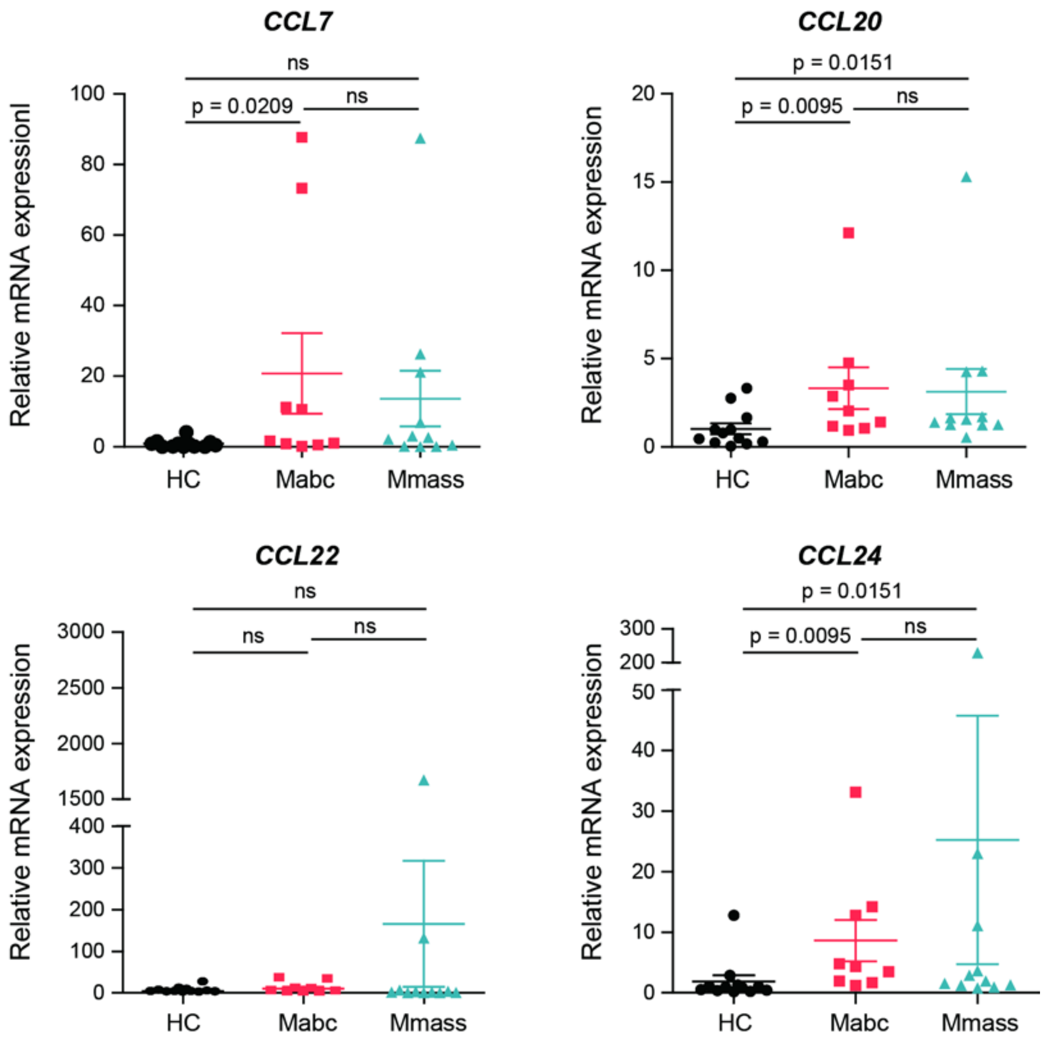

Supplementary Figure 3

Gene	Subject	BMI	ESR	CRP	WBC	Platelet	lymph%	mono%	AST	ALT	Albumin
CCL2	All	0.04	-0.05	-0.15	0.19	-0.09	-0.03	0.13	-0.06	-0.26	-0.20
	Mabc	-0.01	0.09	0.09	***0.33***	-0.06	-0.29	0.07	-0.08	-0.28	-0.17
	Mmass	0.10	-0.23	***-0.42***	-0.04	-0.16	0.11	0.20	-0.04	-0.23	-0.21
CCL5	All	-0.03	0.20	0.11	0.08	0.16	0.30	0.00	0.19	0.04	0.11
	Mabc	-0.20	0.22	0.09	-0.01	0.21	0.27	-0.12	0.29	-0.02	-0.18
	Mmass	0.27	0.20	0.13	0.13	0.15	0.22	0.22	0.04	0.16	***0.45***
CXCL2	All	0.01	-0.09	-0.02	0.01	-0.26	-0.04	0.17	-0.05	-0.05	-0.25
	Mabc	0.05	0.00	0.00	-0.01	***-0.34***	0.00	0.23	0.03	0.04	-0.12
	Mmass	0.00	-0.22	-0.06	-0.06	***-0.30***	-0.25	0.18	-0.11	-0.18	***-0.42***
IL1B	All	0.03	-0.06	-0.09	-0.13	-0.21	-0.04	0.05	0.07	0.21	-0.10
	Mabc	0.12	-0.20	-0.21	-0.29	***-0.40***	0.24	0.21	0.11	0.25	0.07
	Mmass	-0.20	0.20	0.10	0.16	0.01	***-0.47***	-0.16	0.03	0.12	-0.30
IL6	All	0.10	-0.09	-0.02	-0.11	-0.22	-0.07	-0.07	-0.02	0.20	0.00
	Mabc	0.16	-0.24	-0.27	***-0.35***	***-0.34***	***0.40***	0.06	0.25	***0.40***	***0.34***
	Mmass	-0.02	0.10	0.20	0.17	-0.12	***-0.65***	-0.24	***-0.31***	-0.10	***-0.38***
TNFA	All	0.07	0.01	0.04	-0.01	0.01	0.12	0.06	0.16	-0.07	0.07
	Mabc	0.02	-0.02	0.05	-0.12	0.13	0.03	-0.03	-0.02	-0.19	-0.13
	Mmass	0.19	0.12	0.07	0.17	-0.05	0.10	0.23	***0.39***	0.10	0.27

Supplementary Figure 4

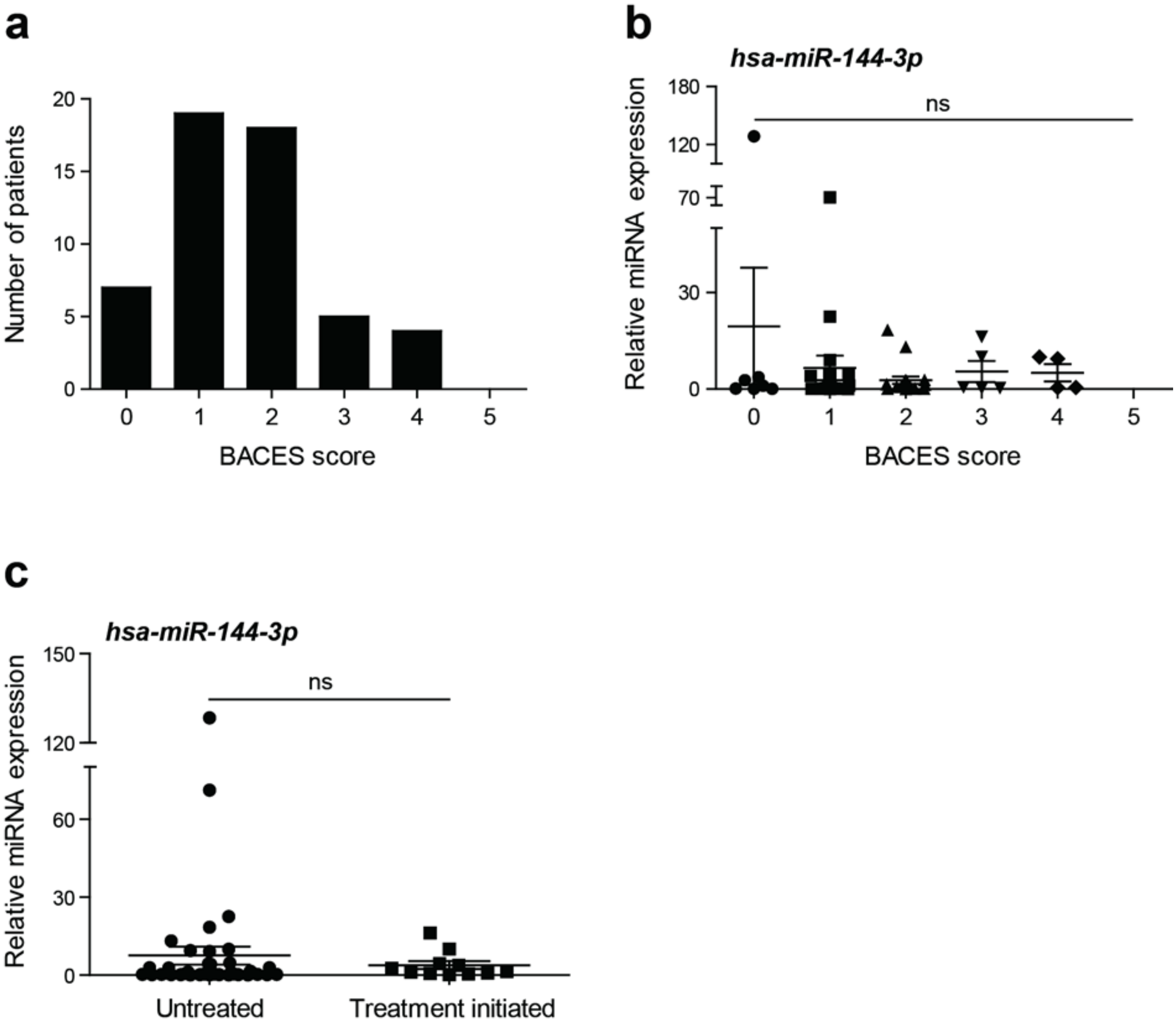

Supplementary Figure 5

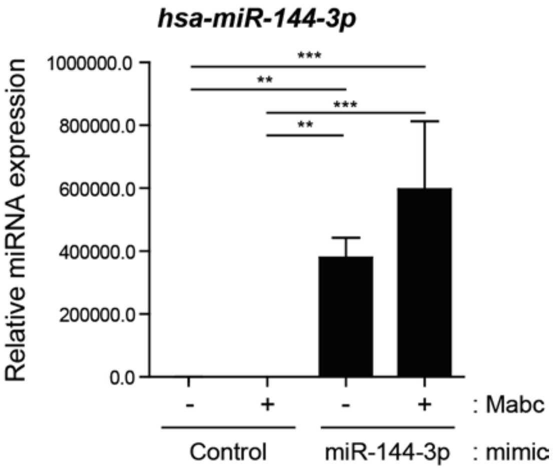

Supplementary Figure 6

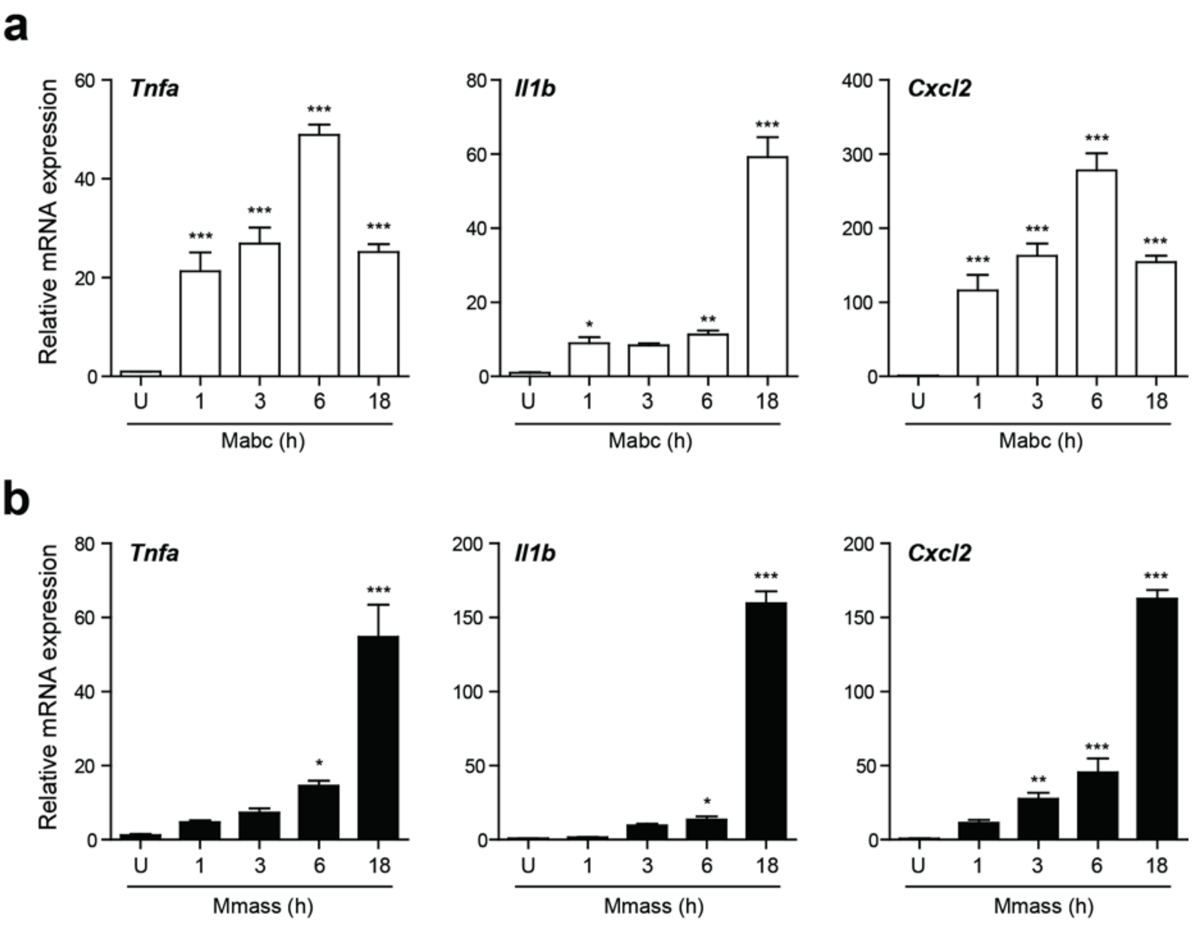

Supplementary table 2. Immune gene expression level based on NanoString n-Counter analysis

Probe Name	NS Probe ID	Class Name	Normalized expression level																							
		Group	HC-1	HC-2	HC-3	HC-4	HC-5	HC-6	Mabc-1	Mabc-2	Mabc-3	Mabc-4	Mabc-5	Mabc-6	Mabc-7	Mmass-1	Mmass-2	Mmass-3	Mmass-4	Mmass-5	Mmass-6	Mmass-7	Mmass-8	Mmass-9	Mmass-1(	Mmass-11
ABCB1	NM_000927.3:3910	Endogenous	246.06	229.9	101.39	164.84	114.51	186.98	595.2	681.98	495.17	519.87	190.59	428.4	182.13	644.73	689.32	558.75	282.83	339.07	249.09	162.21	439.09	341.76	86.09	276.93
ABL1	NM_005157.3:3200	Endogenous	152.96	150.85	121.29	105.47	119.63	119.78	261.7	205.29	179.72	215.93	144.55	259.14	148.35	321.88	222.76	225.86	187.14	284.67	121.32	165.52	166.74	214.44	93.92	251.53
ADA	NM_000022.2:1300	Endogenous	239.41	245.35	221.74	198.44	195.29	238.59	349.46	308.52	389.4	400.14	172.39	277.11	232.07	297.49	266.74	270.05	232.87	241.92	241.57	196.42	226.03	184.28	113.48	154.79
AHR	NM_001621.3:1900	Endogenous	1467.04	878.73	1470.67	1053.11	1010.18	1487.05	3690.06	3972.44	2826.89	5308.19	2791.43	3043.73	2441.1	3570.88	3921.78	2804.54	3049.27	4254.44	2515.6	4228.45	3410.8	2238.18	2210.96	2514.14
AICDA	NM_020661.1:567	Endogenous	6.65	1.82	11.37	4.69	4.09	6.82	3.99	2.32	1	6.14	4.28	1.5	7.34	6.83	2.87	3.93	5.08	4.86	8.59	6.62	7.41	2.51	5.22	13.3
AIRE	NM_000383.2:1864	Endogenous	13.3	4.54	8.53	7.03	5.11	5.84	11.97	8.12	2.81	8.19	12.85	13.48	7.34	5.85	2.87	3.93	11.01	7.77	8.59	6.62	9.26	13.4	5.22	9.67
APP	NM_000484.3:1725	Endogenous	481.48	588.85	712.59	545.31	560.3	490.82	479.51	606.6	526.06	302.92	362.98	395.45	440.63	453.55	473.25	554.82	366.66	611.11	333.91	470.07	301.06	367.73	324.8	452.28
ARG1	NM_000045.2:505	Endogenous	17.29	7.27	6.63	3.91	1.02	6.82	19.15	22.04	6.55	15.35	33.19	38.95	23.5	6.83	22.95	17.68	7.62	4.86	7.52	14.34	18.53	9.21	74.35	31.44
ARG2	NM_001172.3:1150	Endogenous	21.28	16.36	8.53	19.53	25.56	20.45	18.35	22.04	14.04	19.44	19.27	20.97	17.63	26.34	8.6	15.71	16.09	14.57	11.81	9.93	19.45	25.97	504.8	15.72
ARHGDIB	NM_001175.4:833	Endogenous	3533.94	3024.2	3296.69	2924.97	2845.48	3213.67	1338.8	1583.18	1636.23	1367.21	2277.47	2618.33	2905.23	1709.85	1509.63	1664.46	1534.37	1538.94	2861.32	1918.91	1757.28	1973.49	2480.98	2641.11
ATG10	NM_001131028.1:985	Endogenous	57.19	64.52	44.54	43.75	60.32	51.61	39.09	41.75	37.44	35.82	17.13	31.46	30.84	16.58	23.9	21.6	18.63	14.57	36.5	23.17	16.67	24.29	30	30.23
ATG12	NM_004707.2:25	Endogenous	65.17	37.26	47.38	34.37	30.67	34.08	39.09	46.39	35.57	44	43.9	76.39	74.91	36.09	42.07	50.08	33.87	37.89	47.24	56.28	46.32	39.37	24.78	54.42
ATG16L1	NM_198890.2:1975	Endogenous	432.27	439.82	219.84	339.06	287.31	266.83	320.74	357.23	250.86	247.65	482.91	609.65	333.41	378.45	346.1	396.72	478.43	411.94	396.18	372.97	380.73	403.74	172.18	341.02
ATG5	NM_004849.2:1104	Endogenous	492.12	383.48	351.56	318.75	337.41	393.43	337.49	389.71	311.71	257.89	376.9	572.2	323.13	437.95	382.43	394.76	423.39	399.31	345.72	417.11	470.58	433.9	219.14	350.7
ATG7	NM_001136031.2:810	Endogenous	164.93	129.95	262.48	158.59	152.34	206.45	88.56	128.74	136.66	121.78	86.73	79.39	88.13	88.76	76.49	56.96	49.96	68.98	140.65	87.17	85.22	78.74	131.74	74.98
ATM	NM_000051.3:30	Endogenous	33.25	39.07	24.64	37.5	50.1	29.22	23.14	41.75	26.21	11.26	21.41	29.96	48.47	14.63	10.52	19.64	20.32	23.32	36.5	17.66	36.13	26.8	26.09	19.35
B2M	NM_004048.2:235	Endogenous	142959	92138.2	92682.6	88513.2	87097.3	84329.7	64074.7	99986.1	82383.3	89813.5	68973.1	111167	106254	82616.9	87074.7	102322	76634.8	77209.5	87625	89299.6	63613	67348.2	88638.1	94416.2
B3GAT1	NM_018644.3:2388	Endogenous	87.78	40.89	19.9	26.56	27.61	27.27	9.57	29	30.89	15.35	12.85	41.94	39.66	10.73	26.77	12.77	14.4	11.66	26.84	14.34	17.6	25.13	27.39	39.91
BATF	NM_006399.3:293	Endogenous	77.14	65.43	71.07	52.34	46.01	56.48	84.57	67.27	118.88	94.15	47.11	101.86	52.88	75.1	84.13	68.74	68.59	51.49	65.49	76.14	79.67	74.55	45.65	67.72
BATF3	NM_018664.2:770	Endogenous	93.1	29.99	76.76	50	64.41	51.61	29.52	34.8	27.15	27.63	17.13	25.46	35.25	29.26	41.11	25.53	22.02	22.35	66.57	47.45	26.86	25.13	48.26	20.56
BAX	NM_138761.3:342	Endogenous	909.75	711.52	734.39	659.37	566.44	708.96	529.77	453.5	546.66	507.59	525.74	542.24	615.41	502.32	561.21	366.28	395.45	414.85	767.67	539.59	402.96	446.46	490.46	627.63
BCAP31	NM_005745.7:495	Endogenous	462.86	353.49	370.51	329.68	341.5	360.32	297.6	401.3	373.49	341.8	237.71	316.06	243.82	320.9	384.34	355.48	232.02	351.7	315.66	302.35	195.46	211.92	251.75	273.3
BCL10	NM_003921.2:1250	Endogenous	1101.28	789.67	799.77	873.43	817.96	1009.87	982.95	1060.09	808.75	846.32	992.58	1154.88	1202.92	1078.77	1016.3	1049.74	1150.78	1033.73	938.38	1134.36	1056.03	939	1068.31	1112.56
BCL2	NM_000657.2:947	Endogenous	252.71	308.05	302.28	245.31	348.66	276.57	111.7	97.43	160.07	103.36	207.72	212.7	304.04	112.17	147.23	93.29	101.61	73.84	318.88	172.14	174.15	151.61	176.09	313.21
BCL2L11	NM_138621.4:257	Endogenous	231.43	166.29	172.46	179.69	147.23	169.45	265.68	199.49	146.02	167.83	356.56	596.16	240.88	276.03	349.92	296.56	241.33	317.7	243.72	443.59	410.37	521.85	189.14	388.19
BCL3	NM_005178.2:450	Endogenous	288.62	118.13	158.25	148.44	230.05	186.98	459.56	547.44	384.72	436.98	398.32	522.77	750.54	592.06	461.78	549.91	420	535.33	347.87	542.9	551.17	379.45	513.93	420.84
BCL6	NM_001706.2:675	Endogenous	561.28	201.73	395.15	282.81	250.5	444.07	386.16	539.32	346.34	287.57	482.91	823.85	738.79	644.73	546.87	706.05	375.13	616.94	550.79	934.63	628.99	505.1	703.07	576.84
BID	NM_001196.2:1875	Endogenous	21.28	25.44	37.9	19.53	29.65	23.37	33.51	60.31	29.02	38.89	32.12	29.96	52.88	39.02	33.46	32.41	18.63	28.18	21.47	34.21	34.27	27.64	41.74	35.07
BLNK	NM_013314.2:930	Endogenous	63.84	49.98	68.23	54.69	52.14	85.7	14.36	11.6	29.02	13.3	26.77	19.47	22.03	17.56	22.95	12.77	12.7	13.6	30.06	19.86	18.53	16.75	28.7	36.28
BST1	NM_004334.2:710	Endogenous	512.07	242.63	1125.75	461.71	393.64	483.02	119.68	140.34	87.05	108.48	339.43	456.86	541.98	236.04	221.81	233.71	116.86	195.28	548.64	566.07	299.21	290.66	744.81	564.74
BST2	NM_004335.2:560	Endogenous	363.1	258.08	361.98	202.34	176.88	243.46	90.16	71.91	106.71	93.13	106	127.32	210.03	83.88	66.92	93.29	81.29	60.24	237.28	161.1	106.53	81.25	223.05	95.53
BTK	NM_000061.1:570	Endogenous	247.39	168.11	358.19	214.84	211.65	282.41	57.45	56.83	76.76	91.08	113.5	110.84	107.22	90.71	65.97	55.97	50.81	88.41	141.72	119.17	58.36	105.54	127.83	82.23
BTLA	NM_181780.2:305	Endogenous	288.62	328.05	223.63	288.28	320.03	373.95	258.5	187.89	276.14	113.59	177.74	137.81	136.6	146.31	199.82	115.87	176.13	168.08	243.72	126.9	176.01	175.91	118.7	168.09
C14orf166	NM_016039.2:210	Endogenous	1774.28	1514.83	1566.38	1535.92	1456.99	1566.91	1586.93	1406.88	1301.12	1229.06	1899.5	2281.3	1546.62	1578.17	1548.83	1612.42	1805.34	1641.93	1752.22	1735.74	1862.88	1894.75	1099.61	1633.77
C1QA	NM_015991.2:718	Endogenous	35.91	8.18	29.38	20.31	12.27	12.66	7.98	13.92	9.36	6.14	24.63	23.97	17.63	10.73	19.12	14.73	10.16	10.69	17.18	64	19.45	23.45	14.35	16.93
C1QB	NM_000491.3:819	Endogenous	50.54	14.54	44.54	17.19	10.22	20.45	14.36	17.4	15.91	15.35	22.49	34.45	20.56	10.73	22.95	23.57	2.54	19.43	22.55	96	21.31	32.67	11.74	18.14
C1QBP	NM_001212.3:745	Endogenous	594.53	622.47	627.31	552.34	597.11	665.13	430.04	425.66	512.96	354.08	520.38	600.66	539.04	409.66	370.95	355.48	448.8	444	544.35	370.76	576.19	502.59	408.28	494.6
C1R	NM_001733.4:760	Endogenous	11.97	6.36	4.74	8.59	8.18	4.87	19.95	34.8	24.34	27.63	16.06	14.98	23.5	20.48	14.34	33.39	32.18	16.52	10.74	11.03	22.23	14.24	13.04	13.3
C1S	NM_001734.2:775	Endogenous	7.98	11.81	10.42	13.28	6.13	7.79	28.72	27.84	19.66	23.54	16.06	26.96	13.22	18.53	16.25	11.78	22.86	19.43	11.81	14.34	15.75	20.94	7.83	12.09
C2	NM_000063.3:1075	Endogenous	43.89	11.81	45.48	15.62	23.52	21.42	40.69	75.39	39.31	23.54	24.63	58.42	29.38	36.09	63.1	53.03	23.71	38.86	31.14	72.83	35.2	41.04	22.17	30.23
C3	NM_000064.2:4396	Endogenous	14.63	14.54	18.95	9.37	5.11	22.4	14.36	25.52	11.23	27.63	8.57	28.46	26.44	25.36	14.34	17.68	16.94	14.57	16.1	22.07	21.31	12.56	28.7	10.88
C4A/B	NM_007293.2:1217	Endogenous	6.65	14.54	18	9.37	12.27	10.71	16.75	15.08	8.42	23.54	14.99	13.48	7.34	12.68	16.25	15.71	10.16	13.6	6.44	13.24	12.04	9.21	11.74	15.72
C4BPA	NM_000715.3:690	Endogenous	3.99	3.63	2.84	3.91	12.27	3.9	3.19	3.48	1.87	3.07	2.14	8.99	1.47	3.9	4.78	18.66	3.39	6.8	9.66	2.21	7.41	5.03	7.83	9.67
C5	NM_001735.2:2592	Endogenous	31.92	15.45	28.43	8.59	16.36	20.45	13.56	12.76	9.36	10.23	18.2	16.48	11.75	8.78	13.38	5.89	6.77	9.72	19.33	11.03	16.67	15.08	40.44	18.14
C6	NM_000065.2:3170	Endogenous	9.31	10	7.58	10.16	16.36	8.76	17.55	25.52	13.1	11.26	11.78	7.49	16.16	12.68	12.43	14.73	9.31	13.6	10.74	16.55	8.34	19.27	18.26	15.72
C7	NM_000587.2:310	Endogenous	6.65	7.27	2.84	6.25	4.09	3.9	7.98	3.48	2.81	11.26	5.35	8.99	7.34	4.88	5.74	8.84	5.08	6.8	3.22	7.72	8.34	7.54	5.22	4.84
C8A	NM_000562.2:690	Endogenous	7.98	7.27	6.63	7.03	11.25	11.69	21.54	17.4	13.1	11.26	7.5	25.46	7.34	9.75	13.38	9.82	10.16	10.69	11.81	11.03	15.75	9.21	10.44	7.26
C8B	NM_000066.2:620	Endogenous	3.99	2.73	3.79	5.47	5.11	6.82	9.57	9.28	1	4.09	5.35	4.49	8.81	6.83	5.74	6.87	3.39	1.94	3.22	11.03	5.56	4.19	3.91	7.26
C8G	NM_000606.2:407	Endogenous	2.66	5.45	2.84	7.81	7.16	3.9	15.16	10.44	3.74	8.19	3.21	13.48	4.41	3.9	7.65	4.91	3.39	3.89	10.74	6.62	4.63	4.19	9.13	7.26
C9	NM_001737.3:602	Endogenous	1.33	4.54	1.9	8.59	5.11	3.9	17.55	23.2	5.62	5.12	6.42	14.98	4.41	3.9	7.65	4.91	4.23	11.66	2.15	7.72	22.23	20.1	7.83	13.3
CAMP	NM_004345.3:220	Endogenous	17.29	10	55.91	9.37	8.18	13.63	28.72	33.64	19.66	24.56	76.02	49.43	24.97	13.66	20.08	12.77	17.78	11.66	11.81	19.86	40.76	13.4	63.92	117.3
CARD9	NM_052813.4:1525	Endogenous	37.24	11.81	55.91	25.78	22.49	28.24	24.73	29	29.95	25.58	20.34	14.98	36.72	11.7	9.56	33.39	12.7	23.32	25.77	20.97	13.9	16.75	41.74	19.35
CASP1	NM_001223.3:971	Endogenous	1014.83	535.23	1208.19	564.84	551.1	717.72	308.77	484.81	542.91	924.1	224.86	320.55	465.6	298.47	261.01	379.05	213.39	199.17	424.1	261.52	237.14	221.14	641.77	406.33
CASP10	NM_032977.3:20	Endogenous	47.88	11.81	45.48	21.09	13.29	23.37	59.84	46.39	27.15	48.1	20.34	49.43	27.91	22.43	24.86	41.24	15.24	40.81	20.4	22.07	41.69	33.51	13.04	19.35
CASP2	NM_032982.2:1710	Endogenous	372.41	289.88	358.19	271.87	340.48	329.16	277.65	274.88	253.67	274.26	201.3	244.16	202.69	235.07	299.25	211.13	248.11	220.54	224.4	179.86	213.06	208.57	138.27	256.37
CASP3	NM_032991.2:685	Endogenous	259.36	207.19	212.26	210.15	248.46	286.31	374.99	363.03	287.37	387.86	235.56	358	210.03	448.68	430.23	426.18	320.08	371.13	183.6	187.59	276.05	321.66	109.57	305.95
CASP8	NM_001228.4:301	Endogenous	2323.59	1820.16	1468.78	1476.55	1363.95	1541.59	2814.02	2579.48	2540.46	2342.48	2328.87	3307.36	2222.25	2805.2	2508.72	2459.87	1967.93	2190.86	1753.29	1897.95	2083.35	2117.56	1123.09	2136.84
CCBP2	NM_001296.3:1345	Endogenous	7.98	5.45	5.69	5.47	6.13	1.95	12.77	10.44	9.36	24.56	2.14	14.98	10.28	16.58	12.43	3.93	8.47	15.54	7.52	6.62	12.04	14.24	3.91	14.51
CCL11	NM_002986.2:378	Endogenous	9.31	5.45	6.63	7.03	6.13	1.95	12.77	9.28	13.1	11.26	1.07	20.97	16.16	4.88	5.74	14.73	6.77	6.8	6.44	8.83	14.82	11.73	7.83	9.67
CCL13	NM_005408.2:320	Endogenous	1.33	2.73	3.79	3.91	4.09	3.9	14.36	13.92	4.68	8.19	4.28	8.99	4.41	3.9	9.56	6.87	2.54	7.77	9.66	5.52	12.97	10.89	13.04	4.84
CCL15	NM_032965.3:112	Endogenous	2.66	1	1	2.34	3.07	6.82	11.17	4.64	3.74	6.14	6.42	13.48	2.94	1.95	2.87	5.89	2.54	4.86	6.44	11.03	12.97	11.73	3.91	7.26
CCL16	NM_004590.2:367	Endogenous	1.33	1.82	2.84	3.91	4.09	3.9	9.57	5.8	1.87	3.07	4.28	13.48	2.94	1.95	1.91	3.93	3.39	2.91	1.07	4.41	6.48	7.54	6.52	1.21
CCL18	NM_002988.2:585	Endogenous	3.99	4.54	7.58	10.16	3.07	1.95	18.35	13.92	8.42	14.33	11.78	20.97	13.22	7.8	8.6	4.91	11.01	9.72	9.66	13.24	10.19	15.08	9.13	13.3
CCL19	NM_006274.2:401	Endogenous	5.32	3.63	8.53	6.25	8.18	7.79	12.77	11.6	8.42	6.14	10.71	7.49	8.81	9.75	5.74	5.89	2.54	12.63	9.66	12.14	12.97	13.4	14.35	2.42
CCL2	NM_002982.3:123	Endogenous	59.85	18.17	18	25.78	10.22	90.57	1047.58	7875.3	6362.38	820.74	113.5	792.39	176.25	489.64	1126.25	1613.4	1166.02	1362.12	241.57	534.07	1845.28	107.22	446.11	351.91
CCL20	NM_004591.1:35	Endogenous	14.63	7.27	6.63	17.19	8.18	34.08	1127.36	690.1	384.72	520.89	9.64	49.43	38.19	375.52	21.99	80.52	209.16	438.17	30.06	37.52	72.25	30.16	37.83	35.07
CCL22	NM_002990.3:797	Endogenous	5.32	2.73	2.84	6.25	2.04	3.9	320.74	148.46	1001.58	1406.1	6.42	11.98	4.41	29.26	55.45	12.77	18.63	25.26	6.44	5.52	9.26	10.05	3.91	15.72
CCL23	NM_145898.1:336	Endogenous	7.98	2.73	5.69	3.12	3.07	2.92	13.56	22.04	9.36	8.19	7.5	13.48	4.41	8.78	5.74	11.78	9.31	6.8	6.44	18.76	2.78	4.19	3.91	2.42
CCL24	NM_002991.2:18	Endogenous	7.98	2.73	5.69	2.34	5.11	1.95	124.46	344.47	1508.92	1511.51	4.28	14.98	1.47	57.55	107.08	27.5	8.47	38.86	4.29	15.45	1.85	10.05	1.3	10.88
CCL26	NM_006072.4:184	Endogenous	5.32	1.82	1.9	3.12	1.02	4.87	7.98	5.8	4.68	6.14	3.21	8.99	7.34	5.85	1	2.95	1.69	4.86	1.07	3.31	9.26	6.7	1.3	3.63
CCL3	NM_002983.2:159	Endogenous	751.48	256.26	707.86	853.12	475.44	373.95	6895.03	13357.9	2843.74	4262.31	854.45	3572.49	1370.36	1873.71	467.52	2215.35	2787.61	2178.23	478.85	1172.98	2505.76	1252.28	644.38	1010.98
CCL4	NM_002984.2:35	Endogenous	706.26	407.1	262.48	716.4	157.46	366.16	8908.81	13468	2834.38	5818.85	645.66	2456.56	1499.61	2368.23	792.58	973.15	2901.08	2185.03	663.52	878.35	4404.77	2063.12	499.59	1017.02
CCL5	NM_002985.2:280	Endogenous	6904.28	6388.27	2943.24	3878.87	3944.61	4329.69	1750.49	3204.63	3989.47	3573.59	3415.67	7073.08	2467.53	3681.09	2899.75	2731.88	3799.52	1985.86	4147.56	2234.5	3208.86	4787.97	1587.46	4065.67
CCL7	NM_006273.2:120	Endogenous	14.63	1.82	4.74	1.56	5.11	7.79	144.41	671.55	414.67	130.99	23.56	140.8	39.66	86.81	172.09	579.37	201.53	261.35	41.87	143.45	375.17	22.62	92.61	174.14
CCL8	NM_005623.2:689	Endogenous	2.66	1	6.63	2.34	3.07	7.79	5.58	11.6	2.81	8.19	3.21	4.49	4.41	4.88	5.74	11.78	6.77	7.77	2.15	6.62	18.53	5.03	6.52	6.05
CCND3	NM_001760.2:1215	Endogenous	669.01	602.48	555.29	625.77	540.88	471.34	317.55	345.63	211.55	266.07	327.65	456.86	359.85	213.61	267.7	298.52	344.64	250.66	455.23	307.87	259.38	295.69	369.15	349.49
CCR1	NM_001295.2:535	Endogenous	899.11	238.08	585.62	386.71	292.42	786.86	509.83	694.74	566.31	483.03	416.52	1003.59	536.1	614.49	713.23	1288.36	409.84	540.18	498.18	621.25	609.53	231.19	802.21	610.7
CCR10	NM_016602.2:843	Endogenous	3.99	1.82	6.63	3.91	6.13	3.9	12.77	5.8	3.74	7.16	5.35	5.99	10.28	6.83	8.6	4.91	6.77	5.83	3.22	8.83	8.34	7.54	3.91	4.84
CCR2	NM_001123041.2:743	Endogenous	184.88	109.95	570.45	145.31	188.13	176.27	32.71	23.2	54.29	92.1	41.76	52.43	64.63	83.88	45.89	30.44	35.56	29.15	53.68	35.31	42.61	36.86	80.87	48.37
CCR5	NM_000579.1:2730	Endogenous	146.31	92.69	120.34	71.09	84.86	89.59	83.77	177.46	228.4	308.03	46.04	109.35	70.5	140.46	149.15	115.87	71.13	88.41	89.11	61.79	115.79	65.34	56.09	72.56
CCR6	NM_031409.2:935	Endogenous	50.54	46.34	47.38	46.87	36.81	46.74	44.68	96.27	80.5	67.54	44.97	109.35	33.78	85.83	63.1	155.15	39.8	47.61	37.58	48.55	88	84.6	24.78	47.16
CCR7	NM_001838.2:1610	Endogenous	234.09	461.63	293.76	458.59	676.86	510.29	1115.4	850.16	981.92	800.27	392.96	452.37	631.57	501.35	567.9	538.13	608.84	682.03	431.61	311.18	985.63	579.65	216.53	533.3
CCR8	NM_005201.2:245	Endogenous	26.6	5.45	9.48	5.47	6.13	5.84	15.96	11.6	7.49	8.19	14.99	16.48	13.22	10.73	9.56	12.77	3.39	6.8	11.81	9.93	8.34	8.38	1.3	4.84
CCRL1	NM_016557.2:854	Endogenous	5.32	7.27	7.58	8.59	6.13	8.76	18.35	10.44	18.72	22.51	10.71	25.46	26.44	11.7	8.6	7.86	10.16	9.72	10.74	11.03	17.6	16.75	5.22	14.51
CCRL2	NM_003965.4:1110	Endogenous	115.71	74.51	87.18	72.66	56.23	72.06	1038.01	1527.51	746.04	522.94	120.99	341.52	239.41	410.64	489.51	708.99	342.1	602.36	90.19	268.14	411.3	147.43	200.88	175.35
CD14	NM_000591.2:885	Endogenous	1274.19	450.72	2236.33	1219.52	1135.94	1633.13	582.43	605.44	358.51	891.35	526.81	576.69	1517.24	1109.01	1133.89	581.33	520.77	1085.23	1085.47	999.73	340.89	361.86	1674.85	1535.81
CD160	NM_007053.2:500	Endogenous	160.94	186.29	72.02	203.12	144.17	112.97	38.3	29	43.06	30.7	91.01	100.36	61.69	54.62	26.77	23.57	38.95	31.09	210.44	37.52	62.99	131.51	143.48	88.28
CD163	NM_004244.4:1630	Endogenous	160.94	116.32	436.84	171.09	166.66	196.72	193.08	517.29	764.76	749.1	132.77	281.61	149.81	46.82	91.78	123.73	48.27	62.18	115.96	110.35	63.92	106.38	194.36	136.65
CD164	NM_006016.4:2575	Endogenous	2113.45	1618.42	1682.93	1414.05	1331.23	1509.45	782.69	1018.34	914.53	882.14	1376.98	1858.89	1222.02	996.84	928.34	992.78	1009.37	1076.48	1488.1	1219.32	1123.66	1454.99	1040.91	1612
CD19	NM_001770.4:1770	Endogenous	99.75	89.05	67.28	110.16	84.86	115.89	11.97	20.88	39.31	11.26	57.82	23.97	61.69	22.43	46.85	17.68	23.71	24.29	133.13	71.72	26.86	35.18	52.18	78.6
CD1A	NM_001763.2:1815	Endogenous	15.96	5.45	22.74	9.37	8.18	13.63	15.96	8.12	1	8.19	8.57	5.99	8.81	4.88	4.78	4.91	4.23	1	12.88	6.62	10.19	10.05	9.13	12.09
CD1D	NM_001766.3:1428	Endogenous	183.55	90.87	366.72	127.34	130.87	171.4	150.79	115.98	94.54	267.1	132.77	212.7	76.38	243.85	442.66	93.29	103.31	133.1	133.13	201.93	113.01	147.43	66.52	108.84
CD2	NM_001767.3:687	Endogenous	200.84	203.55	154.46	99.22	139.05	131.47	126.86	121.78	198.44	140.2	79.24	98.86	77.84	103.39	84.13	69.72	85.53	54.41	112.73	62.9	121.35	97.17	50.87	91.91
CD209	NM_021155.2:1532	Endogenous	25.27	1.82	18.95	7.81	9.2	9.74	14.36	18.56	12.17	10.23	6.42	14.98	14.69	13.66	13.38	9.82	7.62	13.6	11.81	14.34	8.34	9.21	11.74	10.88
CD22	NM_001771.2:2515	Endogenous	325.86	287.15	138.35	307.03	281.17	413.88	71.01	80.03	136.66	32.75	219.5	116.84	168.91	88.76	127.16	71.68	77.9	80.64	419.8	320	200.09	180.09	183.92	198.33
CD24	NM_013230.2:95	Endogenous	49.21	63.61	71.07	56.25	72.59	55.51	42.29	35.95	21.53	31.72	70.67	44.94	27.91	13.66	33.46	13.75	25.4	19.43	57.98	36.41	41.69	30.99	53.48	53.21
CD244	NM_016382.2:1150	Endogenous	559.95	560.68	522.13	467.96	468.28	514.19	174.73	132.22	377.23	345.9	173.46	181.25	284.94	223.36	361.39	128.64	123.63	122.42	375.78	147.86	186.2	248.78	335.23	237.02
CD247	NM_198053.1:1490	Endogenous	1496.3	1704.75	721.12	1696.08	1386.44	1413.04	948.65	1181.87	1671.8	733.75	1556.86	2383.16	1202.92	1321.64	1125.29	1068.4	1356.55	1024.99	1212.17	822.08	1458.99	1946.68	730.47	2246.88
CD27	NM_001242.4:330	Endogenous	272.66	399.83	346.82	306.25	356.83	377.85	113.29	69.59	115.13	98.24	104.93	70.4	183.6	54.62	60.23	61.86	76.21	61.21	208.29	78.35	99.12	67.85	152.62	149.95
CD274	NM_014143.3:1245	Endogenous	33.25	23.63	10.42	28.12	24.54	22.4	39.09	156.58	62.72	89.03	14.99	50.93	38.19	33.16	58.32	108.02	33.87	25.26	39.73	49.66	40.76	32.67	18.26	33.86
CD276	NM_001024736.1:2120	Endogenous	9.31	4.54	6.63	6.25	6.13	3.9	8.78	24.36	19.66	16.37	7.5	17.97	11.75	9.75	13.38	11.78	4.23	11.66	5.37	6.62	15.75	11.73	10.44	9.67
CD28	NM_001243078.1:2065	Endogenous	430.94	622.47	397.04	463.28	597.11	518.08	728.44	664.59	567.25	383.76	566.42	546.73	594.85	441.85	413.02	487.06	625.77	478.01	502.47	342.07	617.87	503.42	193.05	476.46
CD34	NM_001025109.1:1580	Endogenous	3.99	2.73	5.69	3.91	3.07	3.9	4.79	3.48	3.74	4.09	4.28	7.49	5.88	3.9	1.91	4.91	4.23	2.91	1.07	5.52	3.71	5.03	2.61	6.05
CD36	NM_001001548.2:705	Endogenous	1207.68	477.98	2460.91	1010.93	1086.86	1425.7	194.68	421.02	411.86	828.93	286.96	251.65	638.92	205.81	203.64	334.86	201.53	300.21	660.3	282.49	147.29	397.04	1369.62	366.42
CD3D	NM_000732.4:110	Endogenous	1915.27	2247.25	1373.07	1392.95	1404.85	1469.52	1168.05	1359.33	1599.72	1457.27	857.67	891.25	1067.8	1136.32	827	846.47	829	715.06	1011.39	410.49	818.89	583.84	551.76	759.44
CD3E	NM_000733.2:75	Endogenous	1717.09	2071.87	1199.66	1361.7	1314.87	1344.87	451.58	503.37	523.26	443.12	875.87	946.67	1036.95	456.48	360.44	360.39	680.81	440.11	1263.7	498.76	700.32	588.86	597.42	780
CD3EAP	NM_012099.1:555	Endogenous	9.31	6.36	13.27	5.47	8.18	6.82	7.98	18.56	5.62	5.12	10.71	10.49	5.88	4.88	5.74	3.93	1	1.94	8.59	2.21	7.41	5.86	7.83	6.05
CD4	NM_000616.4:975	Endogenous	739.51	520.69	817.78	655.46	693.22	670	184.3	177.46	318.26	277.33	455.07	597.66	556.66	410.64	392.94	195.41	197.3	325.47	650.64	556.14	346.45	345.11	512.63	447.44
CD40	NM_001250.4:1265	Endogenous	308.57	169.93	153.51	132.03	191.2	254.17	147.6	112.5	283.63	210.81	198.09	122.83	279.07	215.56	283.95	109.98	196.45	151.56	313.51	157.79	228.81	154.13	109.57	310.79
CD40LG	NM_000074.2:1225	Endogenous	180.89	224.45	181.94	212.5	155.41	118.81	130.05	119.46	143.22	120.76	206.65	242.66	95.47	135.58	112.82	131.59	132.1	115.62	111.66	101.52	193.61	137.37	56.09	82.23
CD44	NM_001001392.1:429	Endogenous	4075.27	2819.74	3432.2	3092.16	3277.97	3745.39	4578.07	5025.58	4254.38	4990.95	4322.6	4358.89	4557.59	4968.6	4507.85	4631.03	3700.45	4574.08	4085.29	4529.7	3924.92	3157.92	2786.21	4729.58
CD45R0	NM_080921.3:258	Endogenous	3708.17	1702.02	3423.67	1692.17	1855.75	2298.26	483.5	904.67	1526.71	1009.04	588.91	991.61	2128.25	772.5	793.53	834.69	560.57	509.1	1247.6	660.97	638.25	624.04	2044	1399.16
CD45RA	NM_002838.4:258	Endogenous	2840.98	3647.58	1454.56	2585.13	2804.58	2682.93	2443.82	2033.2	1568.83	2042.64	2240	2982.32	2483.69	2524.29	2102.39	1815.69	2318.49	1743.94	3248.91	1692.71	2056.48	2273.36	1588.76	3629.11
CD45RB	ENST00000367367.1:13	Endogenous	1573.45	1092.28	1423.29	1146.08	1132.87	1248.46	385.36	553.24	553.21	674.4	395.1	428.4	848.95	470.13	456.04	465.46	366.66	347.82	640.98	444.69	267.71	334.22	700.46	801.77
CD46	NM_172350.1:365	Endogenous	2296.99	1536.64	1755.9	1457.8	1449.83	1606.84	1313.26	1442.84	1288.01	1261.81	1264.55	1644.69	2210.5	1112.91	1371.95	1193.11	1229.53	1102.72	1368.92	1282.22	1132.92	1183.59	1686.59	1661.58
CD48	NM_001778.2:270	Endogenous	3333.1	3131.43	3652.04	3216.37	3099.04	3085.12	1874.95	1452.12	1765.4	1393.82	2978.81	2820.55	2451.38	2290.2	2285	1617.33	2219.42	1796.41	2691.68	2629.54	2686.4	2571.57	1657.9	2383.53
CD5	NM_014207.2:1295	Endogenous	518.72	697.89	317.45	481.25	461.12	488.87	524.19	735.34	733.87	602.76	595.33	711.5	655.07	637.9	538.27	594.1	654.56	578.08	571.19	525.25	723.48	625.72	174.79	518.79
CD53	NM_001040033.1:835	Endogenous	3657.63	2431.72	3701.32	2439.82	2421.16	2837.77	2518.82	3728.88	2628.45	2491.89	1797.78	2595.86	3730.68	2598.42	2774.5	3183.59	1576.71	2494.95	2061.44	2444.16	2202.85	2513.77	3559.72	3566.23
CD55	NM_000574.3:101	Endogenous	15.96	12.72	18.95	21.09	18.4	22.4	20.74	35.95	20.59	21.49	13.92	28.46	24.97	15.61	20.08	24.55	7.62	29.15	16.1	27.59	39.83	17.59	32.61	15.72
CD58	NM_001779.2:478	Endogenous	973.59	511.61	712.59	508.59	491.8	690.45	988.54	1322.21	986.6	949.68	739.88	1366.09	1089.83	1506.97	1100.43	1410.13	619.85	1102.72	600.18	1039.46	1518.28	1048.73	769.6	1019.44
CD59	NM_000611.4:730	Endogenous	384.38	341.68	281.44	351.56	349.68	396.35	329.51	300.4	280.82	323.38	352.27	488.32	468.54	351.14	372.87	331.91	348.03	294.38	406.92	335.45	462.25	283.96	507.41	429.3
CD6	NM_006725.3:1280	Endogenous	397.68	467.08	238.79	310.93	282.2	321.37	438.02	535.84	450.24	396.04	352.27	417.91	358.38	510.13	353.74	399.67	357.34	398.34	290.96	225.11	434.46	353.49	109.57	354.33
CD7	NM_006137.6:440	Endogenous	187.54	179.02	129.82	191.4	172.79	150.95	102.13	197.17	137.6	129.97	137.06	196.22	126.31	132.65	137.67	160.06	127.86	118.53	176.08	81.66	93.56	146.59	92.61	211.63
CD70	NM_001252.2:190	Endogenous	10.64	7.27	13.27	4.69	11.25	9.74	24.73	17.4	20.59	15.35	14.99	41.94	17.63	9.75	5.74	11.78	8.47	11.66	18.25	28.69	28.72	27.64	13.04	9.67
CD74	NM_001025159.1:964	Endogenous	10260	5843.04	9891.97	7267.9	6345.32	8323.41	5217.15	6366.35	6227.58	10351.3	7502.71	8985.9	8201.61	8581.42	9773.86	5675.86	6025.71	7301.24	9691.97	10977.2	5698.87	6300.75	5721.11	8223.25
CD79A	NM_001783.3:695	Endogenous	864.53	701.53	513.6	756.24	843.52	1221.2	655.83	339.83	737.61	156.57	571.78	307.07	641.85	580.35	1070.79	364.32	544.48	452.74	1069.37	961.11	537.28	508.45	540.02	956.56
CD79B	NM_021602.2:24	Endogenous	321.87	210.82	235	237.5	276.06	409.99	138.83	75.39	201.25	81.87	115.64	88.38	151.28	124.85	190.26	54.01	116.01	114.64	275.93	155.59	98.19	87.11	125.22	164.47
CD80	NM_005191.3:1288	Endogenous	22.61	29.08	30.32	19.53	19.43	14.61	75	53.35	91.73	151.46	36.41	49.43	35.25	54.62	40.15	37.32	23.71	37.89	37.58	49.66	84.3	38.53	11.74	37.49
CD81	NM_004356.3:735	Endogenous	2178.62	1931.93	1214.82	1424.2	1408.94	1605.86	1045.19	1127.36	1493.95	866.79	1179.96	1867.88	1064.86	1199.72	1025.86	919.14	1042.39	962.81	1668.47	1197.25	1389.52	1269.87	943.08	1330.23
CD82	NM_002231.3:1211	Endogenous	90.44	76.33	76.76	51.56	77.71	54.54	228.19	354.91	203.12	230.26	67.46	116.84	111.63	276.03	297.34	300.49	89.76	247.75	53.68	146.76	160.26	92.98	93.92	106.42
CD83	NM_004233.3:1960	Endogenous	4008.76	1539.36	1125.75	2192.17	1173.77	2471.61	5930.43	8347.35	4554.85	2802.99	4820.49	3869.08	7424.63	4052.71	3830	5241.82	3476.89	5060.83	4794.98	7897.45	6534.43	2538.06	5432.84	4457.49
CD86	NM_175862.3:1265	Endogenous	262.02	145.39	398.94	196.87	188.13	314.55	570.46	461.62	471.77	1086.81	227	405.93	254.1	835.9	1078.44	584.28	300.61	683.97	217.95	480	274.2	251.29	190.44	411.16
CD8A	NM_001768.5:1320	Endogenous	981.58	693.35	418.84	270.31	384.44	679.74	268.08	468.57	546.66	642.67	301.95	461.35	251.16	485.74	383.38	326.02	517.38	384.74	447.72	185.38	274.2	348.46	185.23	250.33
CD8B	NM_004931.3:440	Endogenous	182.22	225.36	158.25	92.97	144.17	186.98	106.91	100.91	106.71	154.53	78.16	80.89	116.03	101.44	109.95	82.49	141.41	92.3	155.68	54.07	55.58	67.01	56.09	48.37
CD9	NM_001769.2:405	Endogenous	216.8	84.51	220.79	210.15	315.94	178.21	501.85	737.66	843.39	1190.17	138.13	166.27	157.16	740.32	402.5	486.08	218.47	756.84	115.96	316.69	153.77	248.78	112.18	227.35
CD96	NM_005816.4:1355	Endogenous	1165.12	1337.63	775.13	967.18	1080.73	899.83	532.96	623.99	692.68	715.33	495.75	633.61	735.85	438.92	349.92	324.05	392.91	297.3	1001.73	279.18	390.92	383.64	442.19	740.09
CD97	NM_078481.2:1370	Endogenous	2263.74	1358.53	1393.92	1434.36	1281.13	1198.8	796.26	1198.11	951.97	998.8	1647.88	3210	1216.14	1368.46	983.79	1185.25	883.2	927.84	1299.13	1529.39	1813.78	2160.28	1453.11	1395.53
CD99	NM_002414.3:625	Endogenous	3971.52	3583.06	2618.21	2623.41	2784.13	2646.9	1091.46	1894.01	1723.28	2075.38	2706.84	3579.98	2367.66	2550.63	1900.66	1771.5	2340.51	2452.21	2671.28	2082.23	1734.12	2558.16	1875.73	2801.95
CDH5	NM_001795.3:3405	Endogenous	1.33	3.63	1	3.12	6.13	2.92	8.78	6.96	1.87	2.05	2.14	3	4.41	4.88	4.78	1.96	2.54	2.91	2.15	4.41	9.26	4.19	3.91	6.05
CDKN1A	NM_000389.2:1975	Endogenous	570.59	209	636.79	253.12	315.94	300.92	481.1	456.98	353.83	413.44	651.01	754.94	696.2	557.92	346.1	708.01	315.85	582.93	558.31	971.04	623.43	493.37	592.2	327.72

CEACAM1	NM_001712.3:2455	Endogenous	41.23	10.9	14.21	14.06	14.31	16.56	15.16	23.2	22.47	18.42	23.56	29.96	33.78	13.66	15.3	25.53	11.85	11.66	19.33	16.55	8.34	11.73	44.35	15.72
CEACAM6	NM_002483.4:1217	Endogenous	11.97	6.36	18.95	10.16	6.13	7.79	22.34	23.2	11.23	32.75	42.83	28.46	16.16	5.85	8.6	9.82	10.16	6.8	13.96	9.93	17.6	3.35	14.35	25.4
CEACAM8	NM_001816.3:825	Endogenous	9.31	4.54	14.21	5.47	8.18	3.9	19.15	19.72	11.23	30.7	61.03	26.96	10.28	9.75	15.3	4.91	11.01	15.54	7.52	15.45	26.86	8.38	20.87	42.33
CEBPB	NM_005194.2:1420	Endogenous	3459.45	1475.75	2666.54	1863.26	1649.21	2296.31	2958.43	4951.35	2666.82	2330.2	3187.61	6237.26	5155.38	3899.58	4216.25	8149.48	2310.87	4274.84	2896.75	5182.95	3490.47	4046.66	4608.46	3724.65
CFB	NM_001710.5:2029	Endogenous	43.89	19.08	25.59	21.09	26.58	19.48	30.32	38.27	45.87	29.68	32.12	41.94	35.25	19.51	35.37	28.48	22.02	33.03	13.96	23.17	28.72	21.78	26.09	36.28
CFD	NM_001928.2:859	Endogenous	230.1	107.23	390.41	163.28	124.74	150.95	63.83	114.82	41.19	71.64	150.97	233.67	164.5	145.33	164.44	74.63	89.76	200.14	176.08	227.31	109.31	118.11	185.23	156
CFH	NM_001014975.2:702	Endogenous	133	96.32	42.64	65.62	41.92	38.95	16.75	30.16	44.93	28.65	13.92	40.44	19.09	35.11	26.77	24.55	47.42	12.63	61.2	20.97	25.94	22.62	10.44	20.56
CFI	NM_000204.3:1780	Endogenous	6.65	7.27	5.69	7.81	7.16	11.69	18.35	13.92	21.53	8.19	8.57	14.98	24.97	10.73	10.52	9.82	9.31	17.49	15.03	8.83	25.94	11.73	7.83	18.14
CFP	NM_002621.2:380	Endogenous	732.86	354.4	1306.74	535.93	586.89	651.5	272.07	260.96	244.31	413.44	486.12	672.56	615.41	380.4	429.27	294.59	200.69	354.62	595.88	669.8	326.07	443.11	710.9	547.81
CHUK	NM_001278.3:860	Endogenous	410.98	308.05	291.86	250.78	270.95	246.38	305.58	392.03	309.83	281.43	412.24	564.71	452.38	391.13	334.62	366.28	361.58	375.02	353.24	408.28	443.72	438.09	305.23	556.28
CIITA	NM_000246.3:470	Endogenous	90.44	77.24	117.5	68.75	61.35	104.2	14.36	13.92	14.98	42.98	25.7	41.94	24.97	30.24	44.94	14.73	13.55	31.09	65.49	27.59	32.42	19.27	35.22	30.23
CISH	NM_145071.2:558	Endogenous	22.61	22.72	70.12	12.5	51.12	10.71	16.75	15.08	24.34	25.58	12.85	26.96	16.16	36.09	17.21	15.71	10.16	12.63	5.37	22.07	18.53	20.1	16.96	15.72
CLEC4A	NM_194448.2:388	Endogenous	508.08	237.17	748.6	388.28	362.97	480.1	221.8	185.57	136.66	209.79	276.25	341.52	428.88	299.44	290.64	185.59	154.11	236.09	356.46	360.83	196.39	236.22	564.81	303.53
CLEC4E	NM_014358.2:570	Endogenous	440.25	69.06	450.11	230.47	152.34	285.34	711.68	534.69	244.31	388.88	113.5	344.52	324.6	192.15	189.3	411.45	144.8	237.06	118.1	199.73	188.05	86.28	558.28	442.6
CLEC5A	NM_013252.2:615	Endogenous	22.61	22.72	144.03	32.03	15.34	49.67	160.37	357.23	1105.48	953.78	48.18	95.87	154.22	221.41	160.62	151.23	105	186.54	51.54	97.1	90.78	59.47	70.44	322.88
CLEC6A	NM_001007033.1:342	Endogenous	22.61	19.99	60.65	21.87	23.52	24.35	15.96	25.52	6.55	14.33	13.92	16.48	24.97	16.58	9.56	8.84	4.23	16.52	39.73	18.76	21.31	22.62	35.22	24.19
CLEC7A	NM_197954.2:55	Endogenous	1981.77	587.94	919.17	1152.33	867.04	1162.77	1648.36	1591.3	710.47	934.33	922.98	1804.97	3275.36	2351.65	3290.78	3082.44	1165.17	2372.54	1593.32	3526.65	1621.1	1672.77	2337.49	3839.53
CLU	NM_001831.2:2340	Endogenous	9.31	6.36	4.74	9.37	4.09	7.79	15.16	12.76	5.62	7.16	5.35	11.98	2.94	2.93	5.74	1.96	3.39	1	6.44	11.03	4.63	7.54	9.13	8.47
CMKLR1	NM_004072.1:770	Endogenous	226.11	162.66	108.97	108.59	89.98	104.2	20.74	24.36	91.73	74.71	21.41	28.46	48.47	39.02	22.95	11.78	7.62	6.8	55.83	12.14	22.23	31.83	63.92	36.28
CR1	NM_000651.4:1695	Endogenous	406.99	270.8	578.98	344.53	436.59	406.09	110.9	75.39	76.76	66.52	230.21	281.61	759.35	135.58	177.83	182.65	77.9	159.34	289.89	344.28	174.15	168.37	687.42	501.86
CR2	NM_001006658.1:485	Endogenous	26.6	31.81	17.06	24.22	38.85	40.9	15.16	13.92	11.23	9.21	12.85	7.49	13.22	4.88	5.74	12.77	7.62	10.69	15.03	12.14	5.56	7.54	14.35	3.63
CRADD	NM_003805.3:675	Endogenous	46.55	42.71	38.85	53.12	60.32	44.8	69.41	119.46	278.01	37.86	31.05	52.43	38.19	74.13	69.79	48.12	37.26	37.89	38.65	43.03	30.57	47.75	23.48	56.84
CSF1	NM_000757.4:823	Endogenous	115.71	29.99	34.11	28.91	36.81	42.85	128.45	364.19	91.73	82.89	78.16	118.33	258.5	100.46	217.98	677.57	173.59	253.58	120.25	243.86	205.65	57.8	165.66	106.42
CSF1R	NM_005211.2:3775	Endogenous	78.47	34.53	103.29	44.53	50.1	50.64	39.89	34.8	47.74	86.99	18.2	17.97	36.72	19.51	28.68	13.75	14.4	26.23	32.21	22.07	12.04	7.54	43.05	33.86
CSF2	NM_000758.2:475	Endogenous	5.32	8.18	2.84	1.56	2.04	4.87	11.17	6.96	7.49	6.14	1.07	8.99	5.88	5.85	2.87	7.86	3.39	4.86	5.37	4.41	11.12	8.38	2.61	8.47
CSF2RB	NM_000395.2:3300	Endogenous	823.3	337.13	809.25	466.4	599.16	402.2	534.56	489.45	497.05	766.5	489.33	440.38	1343.92	746.17	691.24	612.76	296.37	573.22	417.66	722.77	444.65	244.59	1301.79	847.72
CSF3R	NM_156038.2:90	Endogenous	2210.54	571.58	1488.68	934.37	872.15	1090.7	1674.69	2196.73	960.39	1015.18	1127.49	1959.25	5045.23	1452.34	1518.23	3079.5	916.22	1804.18	1517.09	2569.96	1908.27	1339.39	5633.72	3061.95
CTLA4-TM	NM_005214.3:554	Endogenous	138.32	106.32	65.38	149.22	119.63	100.31	116.49	103.23	124.5	92.1	76.02	109.35	92.53	70.23	62.14	95.25	73.67	61.21	60.13	59.59	52.8	61.99	46.96	72.56
CTLA4_all	NM_005214.3:405	Endogenous	464.19	445.27	361.98	586.71	374.22	401.22	331.91	353.75	454.92	359.2	435.79	462.85	383.35	256.53	249.53	321.11	348.03	288.55	284.52	283.59	311.25	278.94	164.35	380.93
CTNNB1	NM_001098210.1:1815	Endogenous	2403.4	1613.88	1642.19	1696.86	1495.84	1810.37	2822.8	2718.66	2696.78	2971.85	3279.69	4785.79	2561.54	3740.59	3150.24	3302.41	2443.82	4014.47	2222.49	3876.45	3991.62	3730.86	1535.28	2674.98
CTSC	NM_001814.4:260	Endogenous	2247.78	1502.11	1923.62	1487.48	1352.7	1591.25	1004.5	1374.41	1266.48	1766.33	996.86	1204.31	967.92	2029.77	1709.45	871.02	856.95	785.02	1146.67	1050.49	925.42	893.77	957.43	1036.37
CTSG	NM_001911.2:160	Endogenous	2.66	5.45	26.53	6.25	2.04	6.82	11.17	1.16	3.74	9.21	23.56	7.49	11.75	7.8	8.6	4.91	7.62	7.77	4.29	6.62	13.9	7.54	6.52	18.14
CTSS	NM_004079.3:685	Endogenous	24844	10298.5	34005.5	17857.6	15538.2	19270.4	4410.52	4842.32	3195.7	4919.31	11676.5	13437.7	18010.1	7715.28	8096.92	6694.18	3770.73	7142.88	16311.1	18520.5	6357.5	9391.66	17946	15036.5
CUL9	NM_015089.2:2675	Endogenous	117.04	159.03	131.72	122.66	178.93	129.52	177.92	144.98	160.07	168.86	117.78	116.84	126.31	116.07	134.81	87.4	121.09	94.24	147.09	103.73	119.5	108.89	104.35	119.72
CX3CL1	NM_002996.3:140	Endogenous	3.99	1.82	2.84	1.56	2.04	2.92	7.98	3.48	1	4.09	5.35	8.99	8.81	1.95	7.65	4.91	5.08	7.77	3.22	1.1	4.63	6.7	9.13	2.42
CX3CR1	NM_001337.3:1040	Endogenous	1480.34	747.87	1241.35	659.37	667.66	1025.45	108.51	155.42	334.17	323.38	98.51	212.7	254.1	203.85	331.75	95.25	135.49	60.24	446.64	76.14	95.41	145.75	627.42	258.79
CXCL1	NM_001511.1:742	Endogenous	315.22	51.8	95.71	81.25	75.66	197.69	1260.61	1928.81	2614.41	1400.99	134.91	128.82	577.23	446.73	239.97	972.16	270.97	983.21	123.47	243.86	285.31	84.6	463.06	511.53
CXCL10	NM_001565.1:40	Endogenous	45.22	12.72	67.28	23.44	10.22	24.35	209.84	226.17	31.83	212.86	8.57	28.46	19.09	75.1	65.97	46.15	62.66	137.96	12.88	75.04	200.09	21.78	32.61	10.88
CXCL11	NM_005409.4:282	Endogenous	17.29	5.45	20.85	13.28	11.25	8.76	23.14	18.56	10.3	26.61	10.71	20.97	7.34	10.73	11.47	12.77	13.55	15.54	13.96	20.97	27.79	14.24	19.57	12.09
CXCL12	NM_000609.5:210	Endogenous	6.65	4.54	9.48	4.69	4.09	10.71	3.99	6.96	3.74	4.09	5.35	8.99	14.69	6.83	6.69	12.77	6.77	3.89	16.1	6.62	9.26	5.86	19.57	12.09
CXCL13	NM_006419.2:210	Endogenous	9.31	3.63	5.69	3.12	5.11	1	7.18	5.8	9.36	14.33	5.35	4.49	13.22	6.83	8.6	4.91	3.39	6.8	5.37	4.41	1.85	5.03	5.22	7.26
CXCL2	NM_002089.3:854	Endogenous	456.21	95.42	156.35	187.5	129.85	913.46	13116.7	13135.2	11121.3	10155.9	829.83	1870.88	1587.74	6266.83	3110.08	5478.48	4138.23	9121.93	784.85	2560.02	2479.82	793.25	845.25	2331.53
CXCL9	NM_002416.1:1975	Endogenous	19.95	12.72	17.06	10.16	11.25	10.71	8.78	26.68	9.36	38.89	11.78	16.48	19.09	7.8	6.69	9.82	7.62	8.74	12.88	9.93	13.9	11.73	6.52	12.09
CXCR1	NM_000634.2:1950	Endogenous	103.74	21.81	8.53	14.84	19.43	8.76	23.14	54.51	16.85	8.19	18.2	38.95	99.88	13.66	14.34	63.83	18.63	11.66	34.36	20.97	7.41	15.08	131.74	178.98
CXCR2	NM_001557.2:2055	Endogenous	1191.72	181.74	132.66	185.15	194.27	161.66	78.99	186.73	82.37	31.72	83.52	185.74	1264.61	55.6	111.86	340.75	95.69	106.87	369.34	182.07	108.38	92.14	2287.92	1018.23
CXCR3	NM_001504.1:80	Endogenous	134.33	103.59	107.08	67.19	74.64	150.95	116.49	156.58	238.69	181.14	212.01	205.21	123.38	322.85	119.51	163.99	185.45	223.46	109.51	109.24	284.39	166.69	46.96	120.93
CXCR4	NM_003467.2:1335	Endogenous	21303.4	15642.6	9141.47	15556.1	9919.8	13230.6	23418.5	29597.9	20545.5	15392.4	19308.7	32401.1	20692	23329.2	21275.3	31432.3	21421.9	20906.9	9882.01	21895.9	21832.1	22293.9	14293.7	20448.1
CXCR6	NM_006564.1:95	Endogenous	34.58	24.54	14.21	12.5	13.29	35.06	50.26	153.1	36.51	88.01	32.12	61.41	29.38	76.08	34.42	101.14	33.02	65.09	25.77	37.52	42.61	38.53	26.09	25.4
CYBB	NM_000397.3:2686	Endogenous	2394.09	1105	4159.95	1785.92	1782.13	2077.2	1009.28	995.14	864.92	1385.64	700.27	696.52	1332.17	839.8	1127.2	527.32	369.2	647.06	1250.82	1095.73	572.48	494.21	1450.5	890.05
DEFB1	NM_005218.3:40	Endogenous	7.98	1.82	6.63	6.25	6.13	5.84	12.77	4.64	5.62	5.12	6.42	11.98	11.75	2.93	12.43	2.95	3.39	6.8	4.29	5.52	11.12	10.89	15.65	8.47
DEFB103A	NM_001081551.2:106	Endogenous	5.32	9.09	6.63	6.25	6.13	5.84	31.12	20.88	9.36	19.44	16.06	20.97	11.75	16.58	12.43	7.86	11.01	16.52	7.52	13.24	9.26	8.38	11.74	10.88
DEFB103B	NM_018661.3:114	Endogenous	9.31	3.63	2.84	1	6.13	2.92	20.74	17.4	5.62	7.16	11.78	8.99	2.94	9.75	6.69	3.93	5.93	8.74	10.74	13.24	15.75	9.21	11.74	8.47
DEFB4A	NM_004942.2:97	Endogenous	3.99	3.63	6.63	1.56	7.16	5.84	55.05	19.72	5.62	11.26	13.92	34.45	14.69	10.73	13.38	3.93	4.23	20.4	10.74	6.62	20.38	18.43	5.22	8.47
DPP4	NM_001935.3:2700	Endogenous	74.48	102.68	90.97	122.66	132.92	88.62	20.74	32.48	24.34	34.79	80.31	31.46	74.91	22.43	12.43	27.5	12.7	16.52	96.63	43.03	44.46	34.34	56.09	70.14
DUSP4	NM_057158.2:3115	Endogenous	183.55	45.44	36.96	45.31	30.67	45.77	266.48	470.89	321.07	210.81	235.56	431.4	257.03	794.94	285.86	366.28	142.26	409.02	79.45	180.97	367.76	387.83	44.35	220.09
EBI3	NM_005755.2:485	Endogenous	3.99	2.73	7.58	2.34	3.07	2.92	1.6	5.8	14.98	14.33	1.07	3	4.41	2.93	1.91	1	1.69	1.94	2.15	2.21	5.56	1	5.22	1.21
EDNRB	NM_003991.2:560	Endogenous	3.99	1.82	1.9	3.12	6.13	4.87	82.98	182.09	283.63	123.83	17.13	40.44	11.75	43.89	76.49	94.27	20.32	41.78	19.33	43.03	29.64	22.62	13.04	85.86
EGR1	NM_001964.2:1505	Endogenous	496.11	134.49	394.2	171.09	245.39	306.76	434.83	312	153.51	183.18	108.15	163.27	229.13	41.94	121.42	183.63	134.64	165.16	283.45	143.45	109.31	88.79	669.16	270.88
EGR2	NM_000399.3:1891	Endogenous	192.86	58.16	139.3	133.59	170.75	241.51	280.84	273.72	96.41	78.8	65.32	110.84	237.94	87.78	124.29	276.92	96.53	173.91	159.98	160	208.43	66.17	255.66	149.95
ENTPD1	NM_001098175.1:8830	Endogenous	150.3	72.7	180.99	67.97	100.2	131.47	71.81	47.55	37.44	55.26	53.54	89.87	108.69	111.19	107.08	72.67	44.88	49.55	88.04	61.79	73.18	57.8	127.83	126.98
EOMES	NM_005442.2:1670	Endogenous	31.92	62.7	51.17	25	33.74	36.03	53.46	82.35	94.54	119.73	31.05	52.43	26.44	82.91	70.75	56.96	90.61	18.46	52.61	18.76	50.95	67.85	23.48	50.79
ETS1	NM_005238.3:4625	Endogenous	5290.93	6472.78	4097.41	5330.42	5493.62	5247.05	3956.55	4507.13	4347.05	4211.14	3127.64	5268.12	3338.52	3196.33	3503.98	3137.44	4017.99	2254.98	4399.88	2000.57	3122.71	4044.98	2434.02	5075.44
FADD	NM_003824.2:1560	Endogenous	9.31	14.54	10.42	5.47	11.25	11.69	19.95	23.2	16.85	16.37	10.71	11.98	20.56	15.61	19.12	20.62	19.48	13.6	13.96	15.45	20.38	10.05	6.52	15.72
FAS	NM_000043.3:90	Endogenous	57.19	33.62	52.12	21.87	22.49	36.03	19.95	26.68	23.4	22.51	10.71	52.43	30.84	25.36	29.64	12.77	17.78	15.54	33.28	23.17	17.6	23.45	23.48	24.19
FCAR	NM_133280.1:75	Endogenous	9.31	3.63	11.37	7.03	3.07	17.53	24.73	26.68	20.59	14.33	8.57	16.48	17.63	15.61	8.6	17.68	10.16	12.63	11.81	11.03	21.31	20.1	11.74	15.72
FCER1A	NM_002001.2:114	Endogenous	492.12	667.91	981.71	635.15	690.15	469.39	81.38	53.35	105.77	56.29	147.76	95.87	470.01	79.98	118.55	24.55	68.59	60.24	303.85	116.97	90.78	93.82	627.42	157.21
FCER1G	NM_004106.1:36	Endogenous	924.38	422.55	1428.98	958.58	668.68	1074.15	865.67	1120.4	658.98	1074.53	700.27	907.73	1119.2	871.02	1032.55	1292.29	570.73	845.25	879.33	1005.25	388.14	489.18	1351.36	922.7
FCGR1A/B	NM_000566.3:1545	Endogenous	45.22	5.45	24.64	14.84	21.47	19.48	43.08	120.62	18.72	46.05	8.57	28.46	92.53	25.36	42.07	243.53	12.7	39.83	7.52	40.83	49.1	13.4	104.35	39.91
FCGR2A	NM_021642.3:60	Endogenous	2412.71	562.49	2146.31	1064.05	1177.86	1258.2	1035.61	1605.22	748.85	1604.64	910.13	1683.64	3710.11	1293.36	1232.37	1837.29	595.29	1051.22	1256.19	1912.29	786.47	753.04	4024.08	2446.42
FCGR2A/C	NM_201563.4:1166	Endogenous	3730.78	1124.99	3860.51	1504.67	1683.97	2697.54	1073.91	1890.54	797.52	2159.3	1191.74	2144.99	3732.15	1774.22	1583.25	1865.77	773.11	1070.65	2109.75	2625.13	1158.86	1101.5	4489.76	2423.44
FCGR2B	NM_001002273.1:870	Endogenous	182.22	145.39	313.65	157.81	226.98	215.22	50.26	46.39	51.48	246.63	83.52	95.87	142.47	40.97	63.1	51.06	32.18	46.63	166.42	92.69	62.99	61.99	212.62	111.26
FCGR3A/B	NM_000570.4:696	Endogenous	12255.1	3383.15	3327.96	3810.9	3205.38	4030.72	773.92	3347.29	1377.88	1419.41	1228.14	2434.09	7379.1	1235.81	1230.46	2522.71	1121.99	679.12	3946.79	3072.03	1381.18	1480.12	13794.1	3938.7
FCGRT	NM_004107.4:1276	Endogenous	1260.89	656.09	1867.71	1053.11	950.88	1147.18	562.49	671.55	512.02	975.27	1098.58	1208.81	1636.21	937.34	1075.57	870.04	486.05	1005.56	1076.89	1532.7	677.16	947.37	1505.28	1073.86
FKBP5	NM_001145775.1:540	Endogenous	602.51	661.54	904.96	582.81	543.94	540.48	1151.3	1403.4	661.79	687.7	846.96	2905.93	577.23	423.32	699.84	1367.9	827.31	529.5	420.88	926.91	581.74	1104.01	496.98	1235.91
FN1	NM_212482.1:1776	Endogenous	7.98	4.54	3.79	3.91	7.16	12.66	268.08	1999.56	1485.52	4243.89	6.42	11.98	10.28	140.46	253.36	182.65	148.19	377.94	4.29	22.07	30.57	7.54	6.52	21.77
FOXP3	NM_014009.3:1230	Endogenous	19.95	18.17	12.32	21.09	17.38	16.56	28.72	20.88	26.21	27.63	12.85	10.49	27.91	14.63	21.03	15.71	8.47	15.54	24.69	19.86	11.12	5.03	20.87	24.19
FYN	NM_002037.3:765	Endogenous	1770.29	1518.46	1284.94	1361.7	1232.05	1355.59	1169.65	1599.42	1642.78	1813.4	1208.87	2332.23	1083.95	1969.3	1700.84	1458.24	1652.08	1200.84	1259.41	851.87	1234.82	1270.71	686.12	2233.58
GATA3	NM_001002295.1:2835	Endogenous	50.54	21.81	17.06	27.34	19.43	18.5	33.51	34.8	44.93	32.75	26.77	49.43	27.91	39.99	25.81	25.53	37.26	30.12	25.77	14.34	26.86	25.97	6.52	29.02
GBP1	NM_002053.1:2110	Endogenous	1147.83	300.78	614.99	262.5	244.37	278.52	1510.33	4037.39	902.36	3458.97	132.77	503.29	837.2	574.5	1625.31	2477.54	530.93	809.31	338.2	1292.15	717.92	159.99	811.34	315.63
GBP5	NM_052942.3:1955	Endogenous	1167.78	379.84	362.93	406.25	253.57	307.73	573.66	1947.37	664.6	1823.64	293.38	805.87	828.39	317.97	952.24	1958.07	392.91	443.03	383.3	1017.39	454.84	215.27	504.8	700.19
GFI1	NM_005263.2:2235	Endogenous	433.6	340.77	175.31	203.12	184.04	292.15	356.64	541.64	592.52	559.78	292.31	605.15	189.47	644.73	544.96	477.24	530.93	361.42	256.61	178.76	480.77	379.45	104.35	406.33
GNLY	NM_006433.2:305	Endogenous	3867.78	3475.83	1293.47	3433.56	3273.88	2558.28	559.29	839.72	1848.71	983.45	1852.39	3753.74	1459.96	1293.36	1650.17	1201.95	1897.64	805.42	2823.74	877.25	1355.24	2258.29	1607.03	4809.39
GP1BB	NM_000407.4:146	Endogenous	522.71	900.54	269.12	415.62	811.82	372.98	66.22	205.29	134.79	615.04	262.33	226.18	67.56	304.32	212.25	514.56	556.34	582.93	115.96	171.04	125.98	302.39	176.09	93.12
GPI	NM_000175.2:1695	Endogenous	430.94	381.66	422.63	367.18	350.7	386.61	246.54	235.45	320.13	370.46	355.49	355	317.25	295.54	252.4	240.59	239.64	240.95	358.6	336.55	230.66	281.45	236.1	310.79
GPR183	NM_004951.3:405	Endogenous	1431.13	1057.74	1193.03	1907.79	1209.56	1718.83	3205.77	2182.81	2441.24	2337.36	2398.47	2696.22	2405.85	1979.05	1677.9	2258.56	1749.46	2578.51	1146.67	2503.75	1884.18	1750.68	1407.45	2199.72
GZMA	NM_006144.2:155	Endogenous	3822.56	3515.82	1665.88	2264.82	2070.46	2430.7	256.91	469.73	780.67	736.82	727.04	1115.94	1017.86	471.11	631	347.62	397.14	222.49	2313.75	473.38	476.14	977.53	1196.14	929.95
GZMB	NM_004131.3:540	Endogenous	4786.84	3267.74	1068.89	3667.15	2095	2565.09	698.12	1462.56	1877.73	1495.14	1588.98	4110.24	1486.4	1804.46	1750.56	1213.73	1222.76	812.22	1876.77	957.8	1238.52	3166.29	1327.88	4739.25
GZMK	NM_002104.2:700	Endogenous	1004.19	973.23	718.28	389.84	460.1	462.57	74.2	161.22	197.51	229.23	236.63	211.2	257.03	181.42	87.96	91.32	171.05	174.88	634.54	185.38	221.4	188.47	224.36	193.49
HAMP	NM_021175.2:99	Endogenous	7.98	8.18	5.69	6.25	3.07	5.84	2.39	16.24	5.62	5.12	1.07	16.48	10.28	7.8	8.6	3.93	6.77	3.89	4.29	7.72	11.12	7.54	2.61	2.42
HAVCR2	NM_032782.3:955	Endogenous	239.41	101.78	159.2	190.62	138.03	144.13	418.87	481.33	497.98	568.99	240.92	624.62	323.13	616.44	674.03	692.3	287.06	542.13	178.23	488.83	518.75	413.8	190.44	764.28
HFE	NM_139011.2:875	Endogenous	18.62	5.45	18	8.59	6.13	13.63	13.56	9.28	9.36	7.16	9.64	19.47	7.34	4.88	6.69	1.96	2.54	7.77	12.88	1.1	12.97	8.38	6.52	9.67
HLA-A	NM_002116.5:1000	Endogenous	11197.7	8660.05	7375.15	5984.31	6448.59	8083.85	5229.92	7911.25	5492.78	7199.37	7518.77	11148.9	7929.89	7842.08	8124.65	7194.01	5747.12	5655.42	6991.7	6534.68	5838.75	7357.02	6108.52	7812.09
HLA-B	NM_005514.6:937	Endogenous	17760.1	10063.1	11321	10372.6	9937.18	9586.48	8497.92	13323.1	10680.4	10846.6	10536.1	16752.5	13547.9	12906.3	13898.3	12045	9221.48	10431.6	13141.7	12198.7	10456.6	11236.2	10837	13609.5
HLA-C	NM_002117.4:895	Endogenous	4273.44	1012.31	2999.15	1635.14	931.45	1974.95	1808.73	3006.3	2566.67	2919.66	1719.62	3937.98	3532.39	3619.64	2792.67	2055.29	2929.03	1691.48	2995.52	2636.16	2857.77	1436.56	2614.02	4371.62
HLA-DMA	NM_006120.3:380	Endogenous	1230.29	699.71	1133.33	803.12	712.65	993.32	536.95	598.48	652.43	1481.83	1043.98	1225.28	1058.98	832	1018.21	602.94	784.12	943.38	1090.84	1432.29	894.85	771.47	763.08	1144
HLA-DMB	NM_002118.3:20	Endogenous	1593.4	816.03	1507.63	993.74	1043.92	1342.93	394.94	386.23	401.57	812.55	791.28	970.64	890.07	611.56	913.04	307.36	386.98	597.51	950.19	1029.53	503.01	692.73	708.29	742.51
HLA-DOB	NM_002120.3:230	Endogenous	195.52	164.48	266.27	181.25	186.09	320.39	152.39	164.7	98.29	104.38	89.94	139.3	130.72	104.37	85.09	161.05	176.98	171.97	235.13	130.21	101.9	121.46	207.4	124.56
HLA-DPA1	NM_033554.2:857	Endogenous	5679.3	2722.51	4920.87	3086.69	2690.06	3090.97	1810.33	2493.65	2063.07	5039.05	3940.34	3747.75	3580.86	3445.05	3220.03	1637.95	2566.6	3381.01	3380.97	4516.46	2754.02	3762.7	2814.9	2919.25
HLA-DPB1	NM_002121.4:931	Endogenous	8280.88	4480.87	6019.14	5283.54	4914.91	5110.71	2963.22	4487.41	4577.32	8251.39	5313.03	5897.23	5390.39	6582.86	7289.05	2225.17	4680.17	5613.65	5164.33	7800.35	4815.14	5113.81	3386.23	5386.23
HLA-DQA1	NM_002122.3:261	Endogenous	1666.55	4.54	1285.89	1656.23	1614.45	1600.99	1765.65	833.92	4.68	6556.69	1255.98	900.24	1139.77	914.91	947.46	882.8	6.77	1494.25	6.44	4009.97	3.71	1910.66	725.25	899.72
HLA-DQB1	NM_002123.3:384	Endogenous	806.01	4.54	610.25	364.06	372.17	379.8	762.75	199.49	2.81	2815.28	935.83	1017.07	317.25	675.94	824.13	593.12	4.23	274.95	9.66	1963.05	1.85	362.7	628.72	838.05
HLA-DRA	NM_019111.3:335	Endogenous	17402.3	8730.02	17674.6	11756.1	11180.5	13786.7	13139	17926.5	15023.7	31496.1	12958.2	16881.3	14194.2	16434.2	17820.1	12069.6	10674.6	17081.9	13805.2	22310.8	12008.2	10793.9	10606.1	15693.1
HLA-DRB1	NM_002124.2:104	Endogenous	582.56	348.95	240.69	1554.67	1262.72	492.76	1400.23	477.85	487.69	6524.97	2267.84	465.85	621.29	1013.42	1251.49	217.02	251.49	2356.02	547.57	6037.02	503.93	359.35	397.84	646.98
HLA-DRB3	NM_022555.3:698	Endogenous	4242.85	2338.12	3557.28	3032.78	2808.67	3557.44	2620.94	3296.26	4652.2	7223.93	4851.54	3861.59	3711.58	4336.55	5628.36	1776.41	2768.14	4039.73	4525.5	6066.82	3643.31	2789.35	2520.11	5495.07
HRAS	NM_005343.2:396	Endogenous	59.85	39.07	42.64	43.75	47.03	40.9	39.09	67.27	70.2	46.05	49.25	43.44	36.72	39.02	43.02	16.69	42.34	35.95	38.65	43.03	44.46	35.18	18.26	44.74
ICAM1	NM_000201.2:2253	Endogenous	585.22	156.3	242.58	218.75	185.06	251.25	1891.71	2731.42	922.95	924.1	552.5	1162.37	1586.27	1140.22	1085.13	1887.37	719.77	1372.81	606.62	1792.02	1598.87	655.87	1356.58	672.37
ICAM2	NM_000873.3:415	Endogenous	404.33	294.42	376.2	302.34	314.91	336.95	179.52	173.98	175.04	219	144.55	161.77	183.6	140.46	148.19	136.5	131.25	149.62	226.54	123.59	190.83	145.75	168.27	172.93
ICAM3	NM_002162.3:1225	Endogenous	2479.21	1530.28	1745.47	1455.45	1515.27	1537.69	844.92	1032.26	718.89	709.19	1366.27	1301.68	2667.29	1083.65	1241.93	1173.47	1071.18	1042.48	1628.75	1742.36	1392.3	1228.82	2180.96	1985.67
ICAM4	NM_001039132.1:463	Endogenous	9.31	12.72	26.53	14.84	10.22	9.74	18.35	22.04	8.42	15.35	7.5	14.98	24.97	21.46	12.43	15.71	15.24	29.15	20.4	13.24	28.72	11.73	19.57	16.93
ICAM5	NM_003259.3:327	Endogenous	13.3	3.63	10.42	7.81	3.07	4.87	35.11	60.31	46.8	42.98	4.28	23.97	41.13	24.38	21.03	66.77	20.32	39.83	7.52	15.45	21.31	11.73	20.87	18.14
ICOS	NM_012092.2:640	Endogenous	404.33	265.34	233.11	321.87	321.05	371.03	432.44	321.27	410.93	222.07	486.12	314.56	540.51	358.94	226.59	364.32	437.79	380.85	289.89	332.14	377.95	254.64	178.7	324.09
ICOSLG	NM_015259.4:1190	Endogenous	216.8	106.32	90.97	149.22	119.63	215.22	374.99	255.16	256.48	340.78	284.82	329.54	336.35	479.89	453.18	279.87	274.36	472.18	255.53	465.66	314.96	292.34	183.92	318.05
IDO1	NM_002164.3:50	Endogenous	31.92	14.54	28.43	17.19	24.54	8.76	70.21	156.58	64.59	29.68	23.56	26.96	29.38	25.36	27.73	25.53	20.32	35.95	23.62	20.97	28.72	21.78	33.91	24.19
IFI16	NM_005531.1:2255	Endogenous	833.94	640.64	1066.99	591.4	522.47	666.11	449.99	639.07	777.86	719.43	291.24	735.47	468.54	502.32	626.22	593.12	415.77	332.27	492.81	368.56	520.61	446.46	637.85	603.44
IFI35	NM_005533.3:415	Endogenous	159.61	121.77	233.11	126.56	121.67	140.23	59.84	99.75	73.95	127.92	53.54	137.81	96.94	61.45	58.32	70.7	46.57	42.75	148.17	75.04	32.42	39.37	187.83	72.56
IFIH1	NM_022168.2:185	Endogenous	239.41	176.29	222.69	158.59	151.32	150.95	293.61	454.66	195.64	255.84	461.49	1333.13	503.79	198.98	252.4	432.07	276.9	258.43	324.25	717.25	651.22	718.7	472.19	273.3
IFIT2	NM_001547.4:1995	Endogenous	247.39	69.97	133.61	66.41	32.72	149	61.43	332.87	216.23	103.36	351.2	2104.55	1186.77	79.01	97.52	373.15	279.44	102.98	424.1	734.9	415	1918.2	559.59	199.53
IFITM1	NM_003641.3:482	Endogenous	6993.39	6039.32	4322.94	5502.29	4762.57	3939.18	3891.92	4957.15	3771.37	2646.42	3806.5	9147.68	6816.56	3534.79	2996.31	8110.2	4432.07	2875.8	6017.89	4515.35	4585.41	3801.23	8314.27	7462.6
IFNA1/13	NM_024013.1:585	Endogenous	11.97	1.82	1.9	4.69	11.25	4.87	23.94	25.52	14.04	22.51	10.71	25.46	24.97	8.78	17.21	15.71	8.47	14.57	9.66	15.45	21.31	14.24	15.65	9.67
IFNA2	NM_000605.3:611	Endogenous	15.96	9.09	6.63	7.81	6.13	8.76	13.56	22.04	3.74	11.26	7.5	16.48	20.56	19.51	10.52	6.87	9.31	3.89	9.66	8.83	9.26	10.89	7.83	12.09
IFNAR1	NM_000629.2:3123	Endogenous	90.44	49.07	61.59	57.81	69.53	71.09	58.24	62.63	55.23	67.54	17.13	35.95	70.5	40.97	59.28	54.01	33.02	57.32	34.36	26.48	59.29	38.53	63.92	32.65
IFNAR2	NM_000874.3:631	Endogenous	1117.24	888.72	1165.55	765.62	1016.31	1011.82	1674.69	1483.43	1581.94	2488.82	714.19	1014.08	1125.08	1713.75	1893.97	1439.59	857.79	1229.02	877.18	1024.01	1062.52	768.96	751.34	933.58
IFNB1	NM_002176.2:610	Endogenous	7.98	8.18	1	4.69	5.11	9.74	19.15	20.88	7.49	8.19	8.57	20.97	19.09	6.83	5.74	9.82	4.23	13.6	9.66	12.14	26.86	13.4	14.35	13.3
IFNG	NM_000619.2:970	Endogenous	260.69	109.95	65.38	146.09	31.7	77.91	76.59	90.47	81.44	29.68	268.76	459.86	220.32	93.64	115.68	48.12	92.3	45.66	302.77	189.79	172.3	271.4	199.57	286.6
IFNGR1	NM_000416.1:1140	Endogenous	3358.37	1608.43	2126.41	1817.17	1409.96	1846.4	3838.46	4747.21	2786.64	3860.13	3249.71	6259.73	3945.12	5171.48	4534.62	5793.7	2950.2	3979.49	1421.53	4626.8	4291.75	4328.94	1672.25	3798.42
IGF2R	NM_000876.1:2605	Endogenous	595.86	403.47	389.46	404.68	376.26	371.03	333.5	538.16	309.83	282.45	321.22	414.92	581.63	383.33	472.3	515.54	272.66	305.07	395.11	311.18	356.64	486.67	543.94	586.51
IKBKAP	NM_003640.3:1130	Endogenous	111.72	85.42	88.13	75.78	66.46	76.93	20.74	34.8	41.19	33.77	24.63	32.95	42.59	17.56	20.08	14.73	29.64	18.46	47.24	16.55	31.5	25.97	48.26	44.74
IKBKB	NM_001556.1:1995	Endogenous	246.06	251.71	206.58	210.15	222.89	197.69	118.88	125.26	123.56	142.25	96.37	158.78	138.06	124.85	129.07	80.52	95.69	121.44	186.82	97.1	98.19	110.57	110.87	185.02
IKBKE	NM_014002.2:2470	Endogenous	180.89	167.2	203.73	130.47	186.09	159.71	90.96	88.15	103.9	133.04	56.75	70.4	111.63	59.5	50.67	34.37	42.34	50.52	94.48	47.45	49.1	61.15	93.92	83.44
IKBKG	NM_003639.2:470	Endogenous	317.88	264.44	232.16	238.28	251.52	220.09	246.54	256.32	287.37	283.47	218.43	290.59	230.6	254.57	250.49	243.53	222.7	241.92	222.25	172.14	201.02	195.17	182.62	222.51
IKZF1	NM_006060.3:4485	Endogenous	377.73	314.42	255.85	269.53	297.53	289.23	167.55	196.01	236.82	164.76	108.15	151.29	198.28	121.92	112.82	88.38	128.71	113.67	229.76	81.66	112.09	120.62	166.96	166.88
IKZF2	NM_016260.2:870	Endogenous	329.85	253.53	110.87	200	223.92	243.46	84.57	96.27	136.66	187.28	72.81	118.33	101.35	93.64	100.39	44.19	68.59	59.26	171.79	46.35	79.67	73.71	110.87	66.51
IKZF3	NM_183232.2:1176	Endogenous	603.84	402.56	350.61	270.31	354.79	395.38	235.37	271.4	267.71	289.61	217.36	317.55	212.97	286.76	245.71	211.13	227.78	137.96	337.13	107.04	297.36	220.3	160.44	216.46
IL10	NM_000572.2:230	Endogenous	15.96	6.36	11.37	7.81	12.27	12.66	31.91	23.2	29.02	4.09	13.92	43.44	44.06	21.46	28.68	62.85	18.63	38.86	19.33	44.14	21.31	48.58	15.65	68.93
IL10RA	NM_001558.2:150	Endogenous	2904.82	2294.51	2545.25	2239.82	2141.01	2200.88	2609.77	2836.96	2593.81	3258.39	2615.83	4072.79	2736.32	4191.22	4232.5	2778.03	2448.05	2889.41	2566.06	3447.21	2477.97	2531.36	1824.86	2376.28
IL11RA	NM_147162.1:400	Endogenous	47.88	72.7	75.81	60.16	72.59	50.64	31.12	46.39	35.57	38.89	36.41	35.95	73.44	25.36	38.24	15.71	45.73	31.09	55.83	29.79	42.61	41.88	32.61	49.58
IL12A	NM_000882.2:775	Endogenous	58.52	55.43	35.06	36.72	41.92	41.88	23.94	38.27	20.59	12.28	51.4	85.38	39.66	27.31	21.99	24.55	41.49	25.26	62.27	44.14	42.61	45.23	35.22	70.14
IL12B	NM_002187.2:1435	Endogenous	10.64	7.27	10.42	5.47	4.09	8.76	23.94	18.56	4.68	17.4	7.5	25.46	8.81	14.63	10.52	5.89	4.23	11.66	11.81	14.34	13.9	9.21	6.52	9.67
IL12RB1	NM_005535.1:225	Endogenous	139.65	89.96	61.59	91.41	86.91	70.12	30.32	39.43	43.06	27.63	66.39	82.38	92.53	62.42	39.2	35.35	38.11	34	80.52	50.76	50.95	71.2	61.31	76.19
IL13	NM_002188.2:516	Endogenous	10.64	3.63	5.69	7.03	6.13	2.92	14.36	12.76	8.42	7.16	8.57	17.97	8.81	2.93	6.69	6.87	10.16	3.89	4.29	8.83	12.04	5.03	9.13	10.88
IL13RA1	NM_001560.2:1230	Endogenous	543.99	195.37	676.58	350.78	335.36	346.69	443.61	421.02	273.33	632.44	361.91	438.88	600.73	186.3	239.97	347.62	198.15	239	302.77	422.62	249.19	244.59	648.29	480.09
IL15	NM_172174.1:1685	Endogenous	142.32	63.61	151.62	68.75	56.23	91.54	42.29	90.47	33.7	105.41	82.45	127.32	86.66	83.88	99.43	75.61	28.79	69.95	84.82	108.14	63.92	65.34	40.44	44.74
IL16	NM_004513.4:1262	Endogenous	1040.1	1044.11	1074.58	903.9	1056.19	1007.92	779.5	895.39	1032.47	810.5	700.27	756.44	710.88	835.9	879.58	754.16	752.79	683.97	784.85	648.83	852.24	660.9	567.42	753.39
IL17A	NM_002190.2:240	Endogenous	3.99	3.63	3.79	7.03	7.16	8.76	3.99	10.44	4.68	4.09	1.07	3	11.75	1	6.69	6.87	1.69	7.77	4.29	3.31	7.41	10.05	6.52	3.63
IL17B	NM_014443.2:177	Endogenous	1.33	6.36	3.79	4.69	3.07	4.87	7.18	6.96	6.55	4.09	2.14	11.98	4.41	3.9	4.78	7.86	3.39	2.91	3.22	5.52	1	8.38	6.52	3.63

IL17F	NM_052872.3:210	Endogenous	13.3	10	5.69	10.16	10.22	7.79	22.34	23.2	5.62	10.23	8.57	17.97	14.69	12.68	17.21	8.84	9.31	8.74	9.66	13.24	16.67	12.56	11.74	15.72
IL18	NM_001562.2:48	Endogenous	49.21	23.63	63.49	79.69	58.28	49.67	101.33	100.91	51.48	51.17	38.55	73.4	51.41	48.77	47.8	37.32	34.72	55.38	51.54	71.72	66.7	66.17	30	41.12
IL18R1	NM_003855.2:2025	Endogenous	260.69	229.9	126.98	221.87	179.95	150.95	185.1	281.84	241.5	433.91	192.73	340.02	146.88	179.47	222.76	357.44	141.41	77.72	212.59	82.76	129.69	145.75	96.53	399.07
IL18RAP	NM_003853.2:2412	Endogenous	577.24	328.95	126.98	709.37	417.16	293.13	212.23	476.69	308.9	781.85	417.59	492.81	352.5	313.1	287.78	977.07	128.71	187.51	493.89	194.21	303.84	363.54	486.54	1072.65
IL19	NM_013371.3:1030	Endogenous	3.99	1	6.63	6.25	5.11	3.9	7.98	5.8	3.74	4.09	3.21	13.48	7.34	2.93	1.91	9.82	2.54	1.94	3.22	3.31	3.71	3.35	1.3	7.26
IL1A	NM_000575.3:1085	Endogenous	7.98	4.54	4.74	7.03	9.2	16.56	404.51	171.66	255.54	57.31	5.35	17.97	49.94	198	12.43	32.41	89.76	214.71	7.52	14.34	46.32	13.4	9.13	10.88
IL1B	NM_000576.2:840	Endogenous	275.32	51.8	122.24	160.15	184.04	349.61	8133.3	8520.17	4875.92	4693.15	114.57	188.74	846.01	2810.08	109.95	509.65	976.34	2788.36	274.86	214.07	381.65	119.78	605.24	116.09
IL1R1	NM_000877.2:4295	Endogenous	25.27	7.27	17.06	14.84	13.29	21.42	125.26	278.36	310.77	117.69	29.98	211.2	80.78	157.04	147.23	303.43	99.07	212.77	16.1	129.1	124.13	103.03	45.65	214.05
IL1R2	NM_173343.1:113	Endogenous	109.06	58.16	41.69	53.91	55.21	48.69	77.39	153.1	64.59	136.11	64.24	148.29	296.69	82.91	110.9	195.41	105.85	104.93	64.42	141.24	83.37	79.58	285.66	327.72
IL1RAP	NM_002182.2:460	Endogenous	263.35	150.85	147.83	146.87	147.23	149	272.87	371.15	210.61	144.29	192.73	398.44	515.54	148.26	188.35	738.45	183.75	226.37	205.07	372.97	199.16	262.18	597.42	584.09
IL1RL1	NM_016232.4:700	Endogenous	27.93	14.54	36.01	25.78	26.58	11.69	30.32	31.32	23.4	45.03	32.12	44.94	44.06	22.43	34.42	82.49	11.01	22.35	6.44	14.34	9.26	19.27	9.13	44.74
IL1RL2	NM_003854.2:595	Endogenous	7.98	5.45	3.79	9.37	9.2	2.92	19.15	24.36	28.08	19.44	9.64	29.96	8.81	26.34	18.17	24.55	16.09	20.4	6.44	25.38	24.08	19.27	11.74	20.56
IL1RN	NM_000577.3:480	Endogenous	105.07	29.99	72.02	36.72	30.67	74.01	379.78	447.7	243.37	299.85	107.07	286.1	508.19	444.77	312.63	492.96	326.01	537.27	187.89	314.49	455.76	152.45	528.28	240.65
IL2	NM_000586.2:300	Endogenous	18.62	6.36	9.48	7.03	5.11	5.84	15.16	8.12	2.81	5.12	25.7	35.95	10.28	7.8	4.78	19.64	15.24	12.63	10.74	12.14	10.19	15.92	11.74	16.93
IL20	NM_018724.3:292	Endogenous	6.65	1	1.9	6.25	8.18	4.87	18.35	11.6	1.87	12.28	6.42	25.46	5.88	3.9	11.47	1	1	8.74	10.74	7.72	13.9	14.24	5.22	7.26
IL21	NM_021803.2:65	Endogenous	11.97	7.27	8.53	3.91	7.16	4.87	11.97	17.4	9.36	6.14	12.85	20.97	24.97	13.66	6.69	7.86	5.08	11.66	15.03	12.14	19.45	13.4	7.83	12.09
IL21R	NM_021798.2:2080	Endogenous	53.2	59.98	56.86	59.37	52.14	48.69	75	61.47	97.35	45.03	93.15	136.31	61.69	150.21	97.52	105.07	60.97	68.98	42.95	59.59	127.84	116.43	28.7	102.79
IL22	NM_020525.4:319	Endogenous	3.99	2.73	1.9	3.91	4.09	7.79	12.77	11.6	3.74	3.07	5.35	8.99	10.28	5.85	5.74	8.84	2.54	6.8	10.74	8.83	7.41	8.38	11.74	10.88
IL22RA2	NM_181310.1:290	Endogenous	3.99	1.82	1.9	1	1.02	1.95	1.6	1.16	3.74	1.02	3.21	5.99	1.47	5.85	1	7.86	1	2.91	3.22	4.41	2.78	3.35	1.3	12.09
IL23A	NM_016584.2:411	Endogenous	54.53	40.89	41.69	68.75	58.28	49.67	88.56	80.03	71.14	62.43	74.95	71.9	105.75	61.45	55.45	51.06	90.61	62.18	77.3	47.45	109.31	41.88	63.92	67.72
IL23R	NM_144701.2:710	Endogenous	9.31	9.09	6.63	8.59	5.11	10.71	13.56	12.76	6.55	10.23	7.5	16.48	8.81	10.73	2.87	9.82	2.54	12.63	12.88	7.72	11.12	7.54	7.83	9.67
IL26	NM_018402.1:80	Endogenous	5.32	9.09	9.48	4.69	7.16	10.71	19.95	9.28	9.36	5.12	18.2	32.95	10.28	10.73	13.38	9.82	5.08	7.77	16.1	16.55	13.9	14.24	9.13	10.88
IL27	NM_145659.3:143	Endogenous	6.65	2.73	3.79	3.12	5.11	1.95	8.78	10.44	6.55	8.19	1.07	4.49	8.81	6.83	6.69	3.93	5.93	6.8	4.29	6.62	2.78	5.86	9.13	4.84
IL28A	NM_172138.1:589	Endogenous	5.32	8.18	8.53	4.69	2.04	6.82	38.3	22.04	9.36	14.33	8.57	20.97	8.81	5.85	6.69	5.89	6.77	12.63	7.52	11.03	25.01	13.4	3.91	8.47
IL28A/B	NM_172139.2:104	Endogenous	13.3	3.63	4.74	8.59	7.16	5.84	11.17	11.6	8.42	9.21	5.35	16.48	5.88	2.93	5.74	10.8	3.39	6.8	5.37	5.52	9.26	5.86	2.61	12.09
IL29	NM_172140.1:233	Endogenous	3.99	2.73	4.74	3.91	1.02	3.9	3.99	8.12	5.62	3.07	1.07	4.49	1.47	2.93	1.91	1.96	2.54	4.86	5.37	6.62	2.78	3.35	1.3	3.63
IL2RA	NM_000417.1:1000	Endogenous	63.84	56.34	75.81	59.37	60.32	48.69	39.09	45.23	80.5	71.64	52.47	100.36	57.28	31.21	38.24	31.42	23.71	26.23	54.76	36.41	64.84	49.42	31.31	60.47
IL2RB	NM_000878.2:1980	Endogenous	1033.45	765.14	422.63	907.8	763.77	726.48	246.54	305.04	634.65	548.52	475.41	597.66	465.6	594.01	487.59	273.97	371.74	299.24	754.79	272.55	415.93	552.01	500.89	972.28
IL2RG	NM_000206.1:595	Endogenous	3849.16	3419.49	2015.54	2558.57	2400.71	2349.88	853.7	1017.18	1368.51	1120.58	1449.79	1778.01	2044.53	860.29	786.84	836.65	941.62	736.44	2392.12	1145.39	1213.51	1302.54	1800.08	1737.77
IL3	NM_000588.3:130	Endogenous	9.31	6.36	4.74	2.34	7.16	4.87	4.79	17.4	5.62	6.14	9.64	11.98	4.41	7.8	9.56	4.91	4.23	2.91	5.37	9.93	2.78	8.38	5.22	8.47
IL32	NM_001012633.1:758	Endogenous	53402.7	36981.1	31230	25659.1	43168.8	43328.1	17148.2	33955.4	37095.9	31921.8	42183.1	54150.6	63931.2	20311.4	30737.5	28235	29657.8	26899.5	33683	34093.6	32538.8	24345.3	42167.5	41444
IL4	NM_000589.2:625	Endogenous	13.3	2.73	18	9.37	11.25	4.87	15.16	9.28	4.68	10.23	11.78	10.49	13.22	4.88	7.65	6.87	6.77	5.83	10.74	6.62	9.26	5.03	15.65	12.09
IL4R	NM_000418.2:705	Endogenous	864.53	579.76	502.23	546.09	599.16	774.2	462.75	572.96	576.61	403.21	637.09	690.53	959.11	640.83	602.32	693.28	533.47	601.39	701.1	620.14	826.3	670.11	628.72	905.77
IL5	NM_000879.2:105	Endogenous	9.31	1.82	3.79	9.37	12.27	2.92	16.75	22.04	2.81	10.23	3.21	17.97	4.41	6.83	10.52	6.87	4.23	5.83	6.44	13.24	12.04	10.05	2.61	6.05
IL6	NM_000600.1:220	Endogenous	21.28	19.08	10.42	7.03	9.2	18.5	212.23	132.22	43.99	24.56	22.49	19.47	23.5	32.19	43.02	28.48	21.17	33.03	20.4	51.86	17.6	18.43	23.48	29.02
IL6R	NM_000565.2:993	Endogenous	270	190.83	424.52	206.25	213.69	240.54	222.6	284.16	316.39	259.93	133.84	275.61	348.1	344.31	348.96	337.8	183.75	303.13	225.47	240.55	271.42	165.85	293.49	312
IL6ST	NM_002184.2:2505	Endogenous	1008.18	1658.4	1016.77	1271.86	1604.22	1457.84	1542.25	1628.41	980.99	1104.21	2354.57	2311.26	1850.65	1151.93	1175.96	1580.99	1823.13	1690.51	1316.31	1590.08	2025.92	1786.69	1037	1354.42
IL7	NM_000880.2:38	Endogenous	42.56	13.63	21.79	18.75	15.34	16.56	1	1.16	1	1.02	12.85	32.95	1.47	1	4.78	1	1	1	4.29	9.93	9.26	14.24	1.3	4.84
IL7R	NM_002185.2:1610	Endogenous	1693.15	1979.18	1611.86	1850.76	1811.78	1563.99	2122.29	2067.99	1738.26	1516.63	886.58	1225.28	1117.73	979.28	1071.75	1164.63	1374.33	948.24	1113.39	552.83	1258.9	841.83	606.55	1102.88
IL8	NM_000584.2:25	Endogenous	20954.9	3729.37	4436.65	7430.39	4511.05	13645.5	137110	166129	106296	106313	12306.1	26742	104208	52847.2	47447.6	165592	54901.2	90728.7	15961.1	38699.4	53165.7	13025.4	66660.2	49930.9
IL9	NM_000590.1:300	Endogenous	3.99	1	3.79	2.34	2.04	3.9	1.6	13.92	4.68	6.14	2.14	5.99	7.34	5.85	1.91	4.91	1	3.89	7.52	5.52	5.56	3.35	1.3	1.21
ILF3	NM_001137673.1:730	Endogenous	1461.72	1494.84	1128.59	1192.18	1106.29	1249.44	1449.7	1736.28	1428.42	1113.42	1929.48	1993.71	1536.33	1724.48	1619.58	1599.65	1718.97	1783.78	1686.73	1783.19	2074.09	1753.19	1086.57	1732.93
IRAK1	NM_001569.3:1995	Endogenous	344.48	249.9	297.55	255.47	223.92	300.92	588.81	731.86	591.59	383.76	464.7	633.61	302.57	724.71	879.58	688.37	334.48	740.33	341.43	529.66	528.02	526.04	272.62	362.79
IRAK2	NM_001570.3:1285	Endogenous	93.1	67.24	44.54	53.12	74.64	72.06	479.51	742.3	325.75	258.91	118.85	437.39	215.91	243.85	370	451.71	195.61	332.27	99.85	333.24	536.35	257.16	138.27	146.33
IRAK3	NM_007199.1:1735	Endogenous	130.34	56.34	239.74	95.31	80.77	132.44	224.2	215.73	99.22	141.22	95.3	272.62	105.75	150.21	140.54	166.94	56.73	91.33	82.67	144.55	92.63	178.42	105.66	156
IRAK4	NM_016123.1:2175	Endogenous	328.52	273.52	303.23	269.53	282.2	314.55	220.21	293.44	254.61	267.1	157.4	223.19	268.79	176.54	208.42	168.9	143.95	154.48	260.9	151.17	173.23	176.74	180.01	264.84
IRF1	NM_002198.1:510	Endogenous	2500.49	1097.73	930.54	1213.27	1018.36	879.38	2160.58	2914.67	1449.02	1841.03	1098.58	1854.4	2780.38	2009.29	2208.51	3338.74	1605.5	2290.93	1506.35	3069.82	2744.76	1388.81	2033.57	1523.72
IRF3	NM_001571.5:1303	Endogenous	54.53	53.61	40.75	42.97	36.81	47.72	25.53	20.88	27.15	31.72	34.26	34.45	41.13	15.61	17.21	20.62	28.79	15.54	34.36	18.76	19.45	19.27	23.48	30.23
IRF4	NM_002460.1:325	Endogenous	408.32	324.41	266.27	309.37	231.07	300.92	325.52	235.45	288.31	170.9	444.36	1088.97	321.66	356.01	312.63	415.38	303.15	306.04	269.49	523.04	528.94	510.12	238.71	701.39
IRF5	NM_002200.3:1845	Endogenous	183.55	82.69	253.01	92.19	87.93	141.21	90.96	104.39	117.94	128.94	95.3	128.82	73.44	84.86	119.51	127.66	88.07	110.76	108.44	113.66	130.61	83.76	63.92	85.86
IRF7	NM_001572.3:1763	Endogenous	203.5	114.5	289.02	152.34	105.31	128.55	80.58	172.82	77.69	117.69	111.36	278.61	224.72	99.49	95.61	210.14	81.29	112.7	209.36	156.69	80.59	84.6	341.75	131.81
IRF8	NM_002163.2:253	Endogenous	917.73	510.7	938.12	710.93	511.22	746.94	669.4	743.46	551.34	377.62	799.85	933.19	559.6	687.64	1021.08	835.67	531.78	730.61	721.5	1214.91	810.55	860.26	549.15	663.91
IRGM	NM_001145805.1:1412	Endogenous	3.99	10.9	12.32	7.03	8.18	7.79	22.34	17.4	9.36	11.26	6.42	19.47	16.16	10.73	9.56	11.78	7.62	7.77	10.74	9.93	9.26	11.73	7.83	6.05
ITGA2B	NM_000419.3:740	Endogenous	473.5	888.72	232.16	528.9	930.43	416.8	69.41	139.18	88.93	557.73	266.62	173.76	67.56	185.32	198.86	661.86	486.05	627.62	96.63	194.21	93.56	252.97	110.87	107.63
ITGA4	NM_000885.4:975	Endogenous	1157.14	999.59	989.29	858.59	1053.12	923.2	455.57	454.66	585.04	497.36	412.24	552.73	490.57	419.41	660.64	405.56	514.84	434.29	783.77	400.56	376.1	402.07	418.71	608.28
ITGA5	NM_002205.2:925	Endogenous	486.8	311.69	454.85	333.59	311.85	321.37	598.39	840.88	853.68	670.3	476.48	930.2	638.92	1113.89	835.6	837.63	541.94	833.59	412.29	638.9	868.91	638.28	418.71	551.44
ITGA6	NM_000210.1:3065	Endogenous	325.86	642.46	472.85	512.49	823.07	511.27	423.66	270.24	360.38	237.42	182.03	199.22	518.48	115.1	132.89	129.62	221.01	122.42	487.44	134.62	148.22	110.57	268.71	483.72
ITGAE	NM_002208.4:3405	Endogenous	222.12	175.38	203.73	192.97	223.92	227.88	386.16	283	267.71	313.15	193.8	226.18	155.69	293.59	391.03	237.64	246.41	279.81	155.68	203.04	216.76	245.43	112.18	223.72
ITGAL	NM_002209.2:3905	Endogenous	2805.07	2188.19	1655.45	1481.23	1376.22	1667.21	501.05	608.91	896.74	718.4	1147.84	1460.45	1373.3	749.09	644.39	382.97	696.9	494.52	1630.9	921.39	717.92	968.32	1037	1378.6
ITGAM	NM_000632.3:515	Endogenous	750.15	573.4	858.52	643.74	521.45	579.43	151.59	158.9	117.94	154.53	495.75	666.57	672.7	399.91	381.47	356.46	200.69	321.58	459.53	509.8	377.95	584.68	723.94	646.98
ITGAX	NM_000887.3:700	Endogenous	1489.65	824.2	1405.29	999.99	738.21	745.96	1471.24	2050.59	1150.41	993.69	1281.68	2052.12	2357.38	2839.34	2662.64	3225.81	1113.52	2938.95	1292.69	2469.54	1673.9	1381.27	1981.39	2603.63
ITGB1	NM_033666.2:2000	Endogenous	3483.39	2956.96	2496.92	2273.41	2428.32	1788.94	1105.82	1550.7	2247.47	1823.64	1343.78	2248.35	1471.71	1809.33	1623.4	1787.21	1243.93	1688.56	1419.38	1560.29	1101.42	1704.6	1150.48	1805.49
ITGB2	NM_000211.2:520	Endogenous	2894.18	1891.04	3055.05	2208.57	2009.11	1907.75	588.02	772.45	759.14	775.71	1526.88	1800.48	2355.91	1527.45	1234.28	1167.58	966.18	1167.81	2134.44	1921.12	1123.66	1558.02	1824.86	2225.11
ITLN1	NM_017625.2:975	Endogenous	18.62	10	13.27	14.84	13.29	8.76	11.97	12.76	13.1	5.12	9.64	16.48	16.16	16.58	12.43	10.8	5.08	8.74	10.74	11.03	12.04	16.75	36.52	16.93
ITLN2	NM_080878.2:135	Endogenous	1.33	1	1	1.56	2.04	1.95	7.98	2.32	1	1.02	6.42	3	5.88	1	1.91	1	1.69	4.86	1.07	3.31	7.41	4.19	3.91	4.84
JAK1	NM_002227.1:285	Endogenous	1983.1	1850.14	1337.06	1706.23	1740.21	1646.76	1312.47	1500.83	1366.64	1281.25	1748.53	1905.33	1675.87	1882.49	1600.45	1496.54	1652.92	1567.12	1598.69	1342.91	1743.38	1615.81	1189.62	1683.35
JAK2	NM_004972.2:455	Endogenous	1306.11	873.28	1626.08	808.59	943.72	904.7	461.96	673.87	408.12	854.51	442.22	605.15	749.07	507.2	640.56	497.87	372.58	419.71	750.49	574.9	301.06	389.5	879.17	503.07
JAK3	NM_000215.2:1715	Endogenous	558.62	488.89	360.09	363.28	448.86	411.93	411.69	416.38	424.03	308.03	268.76	344.52	472.94	306.27	256.23	317.18	313.31	307.98	421.95	295.73	354.79	241.24	357.41	418.42
KCNJ2	NM_000891.2:620	Endogenous	22.61	10	17.06	14.84	12.27	13.63	27.92	51.03	22.47	21.49	3.21	25.46	69.03	25.36	14.34	25.53	11.85	21.37	21.47	24.28	23.16	14.24	52.18	15.72
KIR3DL1	NM_013289.2:1691	Endogenous	45.22	12.72	22.74	21.87	30.67	29.22	11.97	45.23	24.34	8.19	5.35	107.85	41.13	64.38	20.08	16.69	5.08	13.6	52.61	11.03	14.82	28.48	3.91	131.81
KIR3DL2	NM_006737.2:884	Endogenous	79.8	36.35	22.74	41.41	18.4	60.38	27.92	59.15	66.46	37.86	32.12	115.34	32.31	107.29	30.59	36.33	15.24	16.52	48.31	29.79	37.98	98.84	6.52	168.09
KIR3DL3	NM_153443.3:539	Endogenous	1.33	2.73	4.74	1	4.09	1.95	2.39	4.64	1.87	2.05	2.14	1.5	5.88	1.95	1	4.91	2.54	4.86	3.22	3.31	2.78	3.35	2.61	6.05
KIR_Activatin	NM_001083539.1:1146	Endogenous	331.18	184.47	24.64	63.28	106.33	60.38	7.98	98.59	102.97	69.59	127.42	161.77	30.84	130.7	70.75	64.81	53.35	14.57	128.84	32	45.39	169.2	131.74	520
KIR_Activatin	NM_014512.1:718	Endogenous	109.06	79.97	29.38	54.69	43.97	46.74	13.56	59.15	70.2	39.91	42.83	107.85	20.56	100.46	62.14	45.17	24.56	7.77	47.24	32	46.32	82.09	36.52	163.26
KIR_Inhibiting	NM_014218.2:872	Endogenous	281.97	169.02	59.7	214.06	106.33	223.01	116.49	230.81	157.26	70.61	129.56	386.46	110.16	334.56	171.14	182.65	154.96	69.95	137.43	64	116.72	280.61	90	955.35
KIR_Inhibiting	NM_014511.3:592	Endogenous	421.63	224.45	88.13	330.47	159.5	307.73	164.36	289.96	235.89	160.67	150.97	545.24	179.19	645.7	250.49	233.71	164.28	96.18	204	103.73	169.52	433.06	123.92	1439.07
KIT	NM_000222.2:2644	Endogenous	11.97	9.09	9.48	9.37	10.22	2.92	11.97	11.6	16.85	17.4	6.42	13.48	23.5	12.68	8.6	21.6	7.62	5.83	10.74	11.03	25.94	9.21	7.83	10.88
KLRAP1	NR_028045.1:414	Endogenous	91.77	81.78	62.54	39.06	70.55	86.67	67.02	62.63	114.2	132.01	40.69	130.32	49.94	60.47	108.04	49.1	112.62	26.23	76.23	37.52	82.44	77.9	18.26	53.21
KLRB1	NM_002258.2:85	Endogenous	2731.92	2076.41	582.77	2712.47	1332.25	1434.47	1367.52	2202.53	989.41	1878.9	1828.83	2479.02	1034.01	2241.43	1110.95	1716.51	1166.02	1191.13	2052.85	739.32	1231.11	2664.54	1056.57	1349.58
KLRC1	NM_002259.3:335	Endogenous	114.38	181.74	80.55	228.9	160.52	239.56	62.23	60.31	47.74	357.15	116.71	200.72	77.84	70.23	59.28	59.9	170.2	46.63	124.55	55.17	28.72	131.51	73.05	160.84
KLRC2	NM_002260.3:942	Endogenous	449.56	204.46	139.3	285.93	220.85	343.77	205.05	192.53	350.09	418.56	192.73	238.17	255.57	255.55	229.46	113.91	268.43	38.86	275.93	60.69	105.6	207.74	56.09	394.23
KLRC3	NM_007333.2:598	Endogenous	1069.36	605.2	236.9	607.81	534.74	861.85	392.54	170.5	715.15	692.82	269.83	838.82	508.19	660.33	716.09	105.07	677.43	97.16	1046.82	228.42	403.89	608.97	225.66	761.86
KLRC4	NM_013431.2:276	Endogenous	915.07	1131.35	540.13	571.87	512.25	652.47	483.5	521.93	1032.47	737.85	327.65	913.72	381.88	891.5	873.84	333.87	693.52	189.45	746.2	239.45	483.55	685.19	224.36	640.93
KLRD1	NM_002262.3:542	Endogenous	1429.8	1049.57	460.53	910.93	650.28	781.99	428.45	494.09	684.26	645.74	428.3	1178.85	343.69	456.48	601.37	540.09	519.93	149.62	679.63	198.62	366.83	840.99	352.19	862.23
KLRF1	NM_016523.1:275	Endogenous	1113.25	898.72	400.83	910.15	984.62	1026.43	248.93	206.45	498.92	513.73	460.42	1108.45	445.04	515	437.88	298.52	502.14	152.53	815.98	196.42	438.16	743.83	524.37	1105.3
KLRF2	NM_001190765.1:535	Endogenous	3.99	1	5.69	3.12	3.07	4.87	5.58	12.76	6.55	7.16	7.5	7.49	13.22	5.85	2.87	2.95	8.47	2.91	5.37	6.62	3.71	4.19	2.61	6.05
KLRG1	NM_005810.3:65	Endogenous	458.87	785.13	397.99	392.96	439.65	400.25	143.61	201.81	295.79	420.6	280.54	362.49	182.13	198	179.74	95.25	174.44	47.61	290.96	71.72	95.41	180.09	126.53	93.12
KLRG2	NM_198508.2:1347	Endogenous	9.31	6.36	2.84	3.91	3.07	4.87	7.18	4.64	7.49	2.05	3.21	10.49	1.47	1.95	6.69	2.95	1.69	4.86	5.37	6.62	4.63	10.89	3.91	6.05
KLRK1	NM_007360.1:760	Endogenous	3124.28	3340.44	1540.79	2173.42	1634.9	1987.61	1116.99	1267.7	1729.83	1425.55	831.97	1885.86	828.39	1126.57	1399.68	809.15	1504.73	421.66	1625.53	497.66	1006.01	1336.88	585.68	1283.07
LAG3	NM_002286.5:1735	Endogenous	276.65	308.05	111.82	152.34	100.2	147.05	62.23	96.27	116.07	150.43	104.93	202.22	80.78	91.69	71.7	55.97	111.78	53.44	206.14	70.62	113.94	106.38	57.39	102.79
LAIR1	NM_002287.3:1195	Endogenous	522.71	276.25	510.76	317.18	279.13	419.73	346.27	422.18	455.86	388.88	292.31	393.95	312.85	354.06	278.22	258.26	154.11	320.61	271.64	380.69	428.9	185.96	307.84	330.14
LAMP3	NM_014398.3:1400	Endogenous	26.6	22.72	12.32	21.09	21.47	6.82	34.31	37.11	128.24	230.26	21.41	31.46	36.72	22.43	16.25	18.66	14.4	24.29	25.77	23.17	37.98	17.59	39.13	13.3
LCK	NM_005356.2:1260	Endogenous	718.23	912.35	578.98	595.31	604.27	586.25	475.52	533.53	594.4	434.93	275.18	287.6	431.82	379.42	395.81	277.9	411.54	347.82	554.01	166.62	290.87	232.03	279.14	393.02
LCP2	NM_005565.3:1210	Endogenous	3009.9	2284.51	2658.01	2031.23	2225.87	2117.13	3594.32	5461.67	2776.34	2367.04	1888.79	3347.81	4208.03	2618.9	3122.51	6063.74	2160.15	3207.1	2231.07	3857.69	3558.09	2098.3	3641.89	2320.65
LEF1	NM_016269.3:1165	Endogenous	381.72	1006.86	620.68	830.46	1004.05	823.87	678.17	426.82	404.38	298.82	344.78	271.12	693.26	226.29	222.76	216.04	454.72	309.93	773.04	250.49	456.69	262.18	356.1	453.49
LGALS3	NM_001177388.1:495	Endogenous	1278.18	551.59	1555.96	858.59	822.05	1073.17	1820.7	3767.15	2127.66	1404.06	1149.98	1755.54	1332.17	2091.22	2297.43	2660.19	1110.98	2288.01	1407.57	1993.95	1855.47	1279.92	3658.85	945.67
LIF	NM_002309.3:1240	Endogenous	15.96	4.54	1	9.37	6.13	6.82	19.15	38.27	14.04	13.3	12.85	17.97	42.59	13.66	23.9	23.57	9.31	30.12	9.66	17.66	23.16	12.56	13.04	16.93
LILRA1	NM_006863.1:1719	Endogenous	272.66	109.05	406.52	172.65	174.84	182.11	61.43	51.03	56.16	74.71	83.52	92.87	143.94	81.93	51.63	28.48	22.02	55.38	180.38	102.62	44.46	62.82	204.79	73.77
LILRA2	NM_006866.2:317	Endogenous	384.38	180.83	421.68	234.37	197.33	216.19	102.92	111.34	54.29	65.5	158.47	224.69	336.35	128.75	159.66	150.24	71.13	119.5	215.81	290.21	112.09	131.51	416.1	169.3
LILRA3	NM_006865.3:1123	Endogenous	6.65	8.18	5.69	134.37	3.07	4.87	13.56	38.27	9.36	8.19	8.57	7.49	10.28	7.8	3.82	5.89	5.93	7.77	4.29	7.72	9.26	1.68	5.22	12.09
LILRA4	NM_012276.3:1577	Endogenous	22.61	15.45	36.96	16.41	9.2	12.66	4.79	8.12	7.49	4.09	18.2	17.97	10.28	4.88	3.82	3.93	2.54	5.83	10.74	11.03	10.19	10.05	15.65	15.72
LILRA5	NM_181879.2:545	Endogenous	767.44	255.35	891.69	441.4	388.53	521	785.88	381.59	366.93	1042.81	476.48	967.64	879.79	898.33	1135.81	793.44	315.85	511.04	720.43	909.25	375.17	535.25	622.2	1000.09
LILRA6	NM_024318.2:1900	Endogenous	101.08	33.62	143.09	60.16	48.06	86.67	47.07	25.52	38.38	54.24	62.1	43.44	69.03	36.09	48.76	40.26	34.72	36.92	41.87	56.28	28.72	37.69	79.57	54.42
LILRB1	NM_001081637.1:2332	Endogenous	686.3	364.39	604.57	275.78	303.67	426.54	330.31	292.28	332.3	455.4	158.47	293.59	261.44	303.34	359.48	123.73	154.96	186.54	308.14	311.18	170.45	163.34	234.79	193.49
LILRB2	NM_005874.1:595	Endogenous	1046.75	339.86	1144.7	575.78	547.01	629.1	429.24	367.67	378.17	731.71	486.12	620.13	989.95	579.38	679.76	366.28	265.89	510.07	686.07	836.42	427.97	421.33	930.04	711.07
LILRB3	NM_006864.2:2235	Endogenous	577.24	182.65	580.88	282.03	261.75	284.36	258.5	303.88	156.32	283.47	189.52	296.58	846.01	269.21	377.65	502.78	155.81	258.43	327.47	358.62	290.87	193.5	907.86	495.81
LILRB4	NM_001081438.1:1825	Endogenous	300.59	121.77	335.45	146.87	92.02	156.79	208.24	263.28	260.22	625.28	149.9	322.05	196.82	200.93	255.27	188.54	126.17	173.91	236.21	304.55	155.63	159.15	211.31	192.28
LILRB5	NM_001081442.1:327	Endogenous	14.63	3.63	7.58	8.59	2.04	6.82	4.79	9.28	7.49	13.3	5.35	10.49	4.41	1.95	6.69	9.82	5.08	7.77	7.52	2.21	4.63	6.7	7.83	8.47
LITAF	NM_004862.3:1000	Endogenous	3001.92	1977.36	1327.58	1710.14	1477.44	1686.69	2605.78	4210.21	2184.76	1775.54	2213.23	4887.65	3817.33	3523.08	3181.79	5247.72	2190.63	2827.23	1622.31	2944.03	3289.45	3251.73	2696.2	3827.44
LTA	NM_000595.2:885	Endogenous	102.41	83.6	57.8	67.97	81.8	63.3	78.99	55.67	64.59	62.43	193.8	426.9	120.44	83.88	81.27	68.74	92.3	80.64	113.81	217.38	306.62	251.29	61.31	85.86
LTB4R	NM_181657.3:2400	Endogenous	133	109.05	147.83	114.84	86.91	118.81	68.62	86.99	67.4	69.59	70.67	107.85	111.63	92.66	71.7	84.45	55.89	70.92	110.59	70.62	62.07	102.19	82.18	136.65
LTB4R2	NM_019839.4:1250	Endogenous	184.88	180.83	211.31	147.65	145.19	148.02	104.52	118.3	171.3	140.2	149.9	182.74	149.81	124.85	114.73	116.86	120.24	149.62	188.96	90.48	113.94	127.32	86.09	194.7
LTBR	NM_002342.1:1435	Endogenous	199.51	93.6	294.7	135.94	108.38	157.76	138.03	173.98	147.9	193.42	123.14	163.27	168.91	193.13	200.77	191.49	99.92	142.82	130.99	194.21	101.9	115.59	157.83	141.49
LTF	NM_002343.2:590	Endogenous	25.27	12.72	103.29	7.03	5.11	13.63	56.65	33.64	57.1	81.87	129.56	79.39	30.84	27.31	50.67	26.51	55.04	24.29	11.81	25.38	70.4	19.27	80.87	187.44
LY96	NM_015364.2:360	Endogenous	357.78	141.76	483.27	214.06	176.88	238.59	125.26	212.25	99.22	97.22	163.82	272.62	450.91	158.01	183.56	289.68	93.99	182.65	234.06	285.8	140.8	138.21	615.68	309.58
MAF	NM_005360.4:2198	Endogenous	118.37	86.33	49.28	55.47	47.03	52.59	46.28	52.19	50.55	32.75	56.75	107.85	44.06	59.5	71.7	67.76	44.03	48.58	44.02	51.86	87.08	73.71	27.39	82.23
MALT1	NM_006785.2:909	Endogenous	1000.2	952.33	831.99	896.87	895.67	924.17	1067.53	924.39	980.05	881.12	1177.82	1625.22	866.57	1098.28	875.76	1047.78	889.12	1121.17	789.14	1115.6	1157.93	1208.72	487.85	995.26
MAP4K1	NM_007181.3:780	Endogenous	684.97	636.1	506.96	437.5	466.24	531.72	224.2	208.77	307.03	259.93	337.28	365.49	374.54	224.34	217.03	165.96	257.42	166.14	531.46	242.76	289.95	285.64	253.05	362.79
MAP4K2	NM_004579.2:1610	Endogenous	397.68	485.25	323.13	339.06	389.55	360.32	318.34	289.96	319.2	303.94	279.46	260.63	343.69	192.15	208.42	239.6	276.9	243.86	354.31	237.24	199.16	216.95	204.79	268.46
MAP4K4	NM_004834.3:3315	Endogenous	1016.16	876	860.42	842.96	927.36	897.88	765.94	866.4	818.11	575.13	729.18	1023.07	1051.64	993.91	1144.41	1050.72	706.22	953.1	919.06	835.32	966.18	857.75	987.43	898.51
MAPK1	NM_138957.2:430	Endogenous	1820.84	1467.57	1609.02	1318.74	1357.81	1416.94	885.62	1055.45	964.14	1052.02	782.71	1262.73	1293.99	1086.58	1001.96	908.33	780.73	773.36	1055.41	683.04	779.98	938.16	2257.92	1220.19
MAPK11	NM_002751.5:1310	Endogenous	10.64	6.36	10.42	5.47	8.18	5.84	13.56	9.28	4.68	9.21	10.71	17.97	4.41	8.78	8.6	13.75	9.31	13.6	10.74	9.93	8.34	10.89	9.13	16.93
MAPK14	NM_001315.1:450	Endogenous	786.06	580.67	755.24	506.24	607.33	639.81	1049.18	1191.15	677.7	805.39	579.27	937.69	920.92	918.81	1215.16	1163.65	784.12	760.73	529.32	782.35	724.4	763.09	628.72	979.53
MAPKAPK2	NM_004759.3:710	Endogenous	569.26	462.54	311.76	374.21	370.13	366.16	761.95	1065.89	683.32	732.73	813.77	1333.13	781.39	1092.43	950.33	1380.67	992.43	1112.43	554.01	984.29	997.67	877.01	521.76	960.19
MARCO	NM_006770.3:1434	Endogenous	143.65	10	80.55	43.75	32.72	66.22	43.08	109.02	36.51	83.92	22.49	32.95	16.16	63.4	61.19	110.96	26.25	73.84	6.44	216.28	46.32	32.67	67.83	54.42
MASP1	NM_139125.3:35	Endogenous	3.99	4.54	4.74	7.03	6.13	3.9	8.78	9.28	5.62	10.23	7.5	16.48	8.81	5.85	6.69	2.95	4.23	5.83	5.37	4.41	10.19	13.4	2.61	13.3
MASP2	NM_139208.1:330	Endogenous	6.65	4.54	8.53	6.25	3.07	1.95	6.38	12.76	7.49	3.07	5.35	13.48	16.16	3.9	4.78	2.95	7.62	9.72	10.74	4.41	9.26	8.38	5.22	10.88
MBL2	NM_000242.2:1756	Endogenous	1.33	1.82	3.79	3.12	3.07	4.87	10.37	15.08	3.74	5.12	3.21	8.99	7.34	6.83	7.65	8.84	3.39	5.83	7.52	6.62	8.34	10.05	6.52	8.47
MBP	NM_002385.2:132	Endogenous	328.52	318.96	264.38	255.47	264.81	296.05	233.77	266.76	322	317.24	120.99	316.06	286.41	375.52	309.77	240.59	176.98	219.57	273.78	124.69	233.44	190.98	349.58	305.95
MCL1	NM_021960.3:1260	Endogenous	10274.6	5674.02	6910.83	7034.3	7577.37	7099.29	7845.28	6801.29	5434.74	5704.23	10323.1	14053.3	11419.7	7218.81	7138.95	10898.1	7096.9	7173.96	7648.78	13642.1	7364.44	10025.8	12479.2	9910.23
MIF	NM_002415.1:319	Endogenous	477.49	418.92	367.67	316.4	389.55	372.01	302.39	334.03	513.89	435.95	399.39	405.93	336.35	255.55	177.83	266.12	289.6	248.72	390.81	225.11	266.79	224.49	272.62	401.49
MME	NM_000902.2:5059	Endogenous	123.69	13.63	11.37	16.41	21.47	14.61	23.14	55.67	14.04	8.19	16.06	31.46	145.41	14.63	19.12	44.19	11.85	14.57	44.02	49.66	17.6	16.75	281.75	169.3
MR1	NM_001531.2:7695	Endogenous	99.75	79.97	116.55	74.22	99.18	81.8	106.11	100.91	98.29	95.17	54.61	113.84	98.41	83.88	94.65	79.54	80.44	68.01	80.52	65.1	105.6	72.04	57.39	101.58
MRC1	NM_002438.2:525	Endogenous	5.32	4.54	15.16	14.84	13.29	8.76	7.98	10.44	25.27	38.89	7.5	4.49	7.34	4.88	5.74	3.93	2.54	1.94	6.44	8.83	2.78	3.35	6.52	9.67
MS4A1	NM_152866.2:620	Endogenous	861.87	688.81	633	797.65	955.99	926.12	216.22	91.63	393.14	128.94	472.2	137.81	389.22	173.62	231.37	103.11	182.06	162.25	926.57	414.9	221.4	367.73	393.93	468
MSR1	NM_002445.3:326	Endogenous	119.7	37.26	87.18	58.59	43.97	87.65	1188	2540.04	770.37	597.64	106	411.92	127.78	352.11	1402.55	1459.23	476.74	905.49	80.52	470.07	289.95	307.42	138.27	279.35
MUC1	NM_001018017.1:725	Endogenous	13.3	4.54	5.69	7.03	5.11	4.87	24.73	19.72	18.72	17.4	10.71	16.48	13.22	21.46	12.43	17.68	11.85	13.6	10.74	22.07	12.04	13.4	9.13	12.09
MX1	NM_002462.2:1485	Endogenous	1002.86	910.53	1705.68	746.09	530.65	957.28	362.22	959.19	433.39	537.27	321.22	1578.79	1038.42	358.94	333.67	1157.76	558.88	461.49	1876.77	609.11	445.57	242.92	2461.41	650.6
MYD88	NM_002468.3:2145	Endogenous	1155.81	641.55	1348.43	711.71	673.79	915.41	464.35	567.16	516.7	544.43	617.82	1005.09	1025.2	786.16	705.58	603.92	539.4	548.93	875.04	902.63	509.49	484.16	1090.48	985.58
NCAM1	NM_000615.5:1620	Endogenous	203.5	159.93	84.34	186.72	122.69	141.21	35.9	38.27	39.31	34.79	66.39	179.75	101.35	45.84	73.62	33.39	67.74	19.43	164.27	43.03	69.48	144.07	99.13	194.7
NCF4	NM_000631.4:210	Endogenous	246.06	109.05	288.07	109.37	142.12	169.45	77.39	110.18	85.18	110.52	95.3	112.34	290.82	63.4	72.66	121.77	43.19	51.49	163.2	126.9	68.55	80.41	520.46	183.81
NCR1	NM_004829.5:602	Endogenous	524.04	325.32	112.76	447.65	297.53	310.65	127.66	153.1	210.61	310.08	192.73	381.96	205.63	150.21	154.88	122.75	122.78	83.55	370.41	98.21	134.32	291.5	206.1	449.86
NFATC1	NM_172389.1:1984	Endogenous	182.22	152.66	129.82	142.19	190.18	137.31	131.65	133.38	129.18	157.6	117.78	146.79	123.38	145.33	124.29	107.04	88.91	102.01	141.72	114.76	137.1	92.98	80.87	118.51
NFATC2	NM_012340.3:1815	Endogenous	1191.72	1126.81	600.78	885.93	782.17	815.1	505.04	687.78	749.78	663.14	487.19	736.97	602.2	990.01	644.39	572.5	533.47	564.47	717.21	312.28	701.24	727.07	502.2	813.86
NFATC3	NM_004555.2:2190	Endogenous	1488.32	1443.04	974.13	1124.99	1167.64	1242.62	796.26	778.25	901.42	776.73	655.3	760.93	950.29	865.16	851.85	656.95	799.36	656.77	1153.12	478.9	582.67	631.58	645.68	784.84

NFIL3	NM_005384.2:1795	Endogenous	796.7	372.57	474.75	432.81	309.8	568.72	558.5	424.5	250.86	335.66	861.95	1354.1	1057.51	441.85	515.32	925.03	378.51	624.71	686.07	1361.67	467.8	757.23	1646.16	1211.72
NFKB1	NM_003998.2:1675	Endogenous	272.66	225.36	192.36	197.65	187.11	241.51	464.35	540.48	448.37	428.79	723.82	813.36	450.91	523.78	429.27	462.51	481.82	573.22	370.41	719.46	1154.23	840.99	303.93	342.23
NFKB2	NM_002502.2:825	Endogenous	508.08	308.96	346.82	367.18	320.03	327.21	1089.07	1236.39	887.38	916.93	667.07	802.87	1041.36	1117.79	941.73	842.54	862.87	989.04	682.85	855.18	1275.58	787.38	748.73	685.67
NFKBIA	NM_020529.1:945	Endogenous	8932.6	5038.82	4848.86	5731.19	4760.52	5603.48	31919.7	33925.3	16977.3	16705.4	11840.3	17706.7	25612.4	16278.2	6449.62	13019.1	12661.1	16141.4	16303.6	17561.6	18378.7	11362.6	20490.9	11672.2
NFKBIZ	NM_001005474.1:2030	Endogenous	2304.97	1296.74	1658.3	1505.45	1787.24	2016.82	4203.08	4233.41	1946.06	1869.69	2130.78	2741.16	5362.48	2639.39	1196.99	1792.12	2231.28	2708.7	3112.55	3278.38	2724.38	1763.24	2953.17	2737.86
NLRP3	NM_001079821.2:415	Endogenous	590.54	234.45	522.13	389.06	293.44	505.42	1792.77	1296.7	717.96	918.98	919.77	1707.61	991.42	1793.73	678.81	632.4	581.74	1306.74	820.28	1459.88	884.66	1039.52	646.98	719.53
NOD1	NM_006092.1:3285	Endogenous	107.73	87.24	101.39	88.28	78.73	67.19	47.07	52.19	50.55	60.38	69.6	47.93	76.38	46.82	61.19	36.33	44.03	30.12	86.97	29.79	27.79	51.93	44.35	70.14
NOD2	NM_022162.1:4080	Endogenous	462.86	209.91	403.68	264.84	210.62	290.2	342.28	366.51	200.32	357.15	289.1	497.3	606.6	485.74	451.26	566.6	173.59	310.9	324.25	513.11	533.57	361.86	443.5	361.58
NOS2	NM_000625.4:605	Endogenous	6.65	10	7.58	8.59	5.11	5.84	10.37	10.44	11.23	9.21	6.42	8.99	5.88	6.83	7.65	3.93	4.23	1	7.52	11.03	6.48	9.21	5.22	12.09
NOTCH1	NM_017617.3:735	Endogenous	230.1	110.86	147.83	121.09	142.12	119.78	231.38	227.33	196.57	176.02	87.8	94.37	290.82	174.59	182.61	230.77	95.69	177.79	147.09	113.66	164.89	99.68	275.23	165.67
NOTCH2	NM_024408.3:2842	Endogenous	676.99	379.84	907.8	524.99	531.67	619.36	388.55	408.26	436.2	747.06	184.17	193.23	458.26	419.41	602.32	191.49	152.42	293.41	330.69	264.83	149.14	112.24	345.67	365.21
NT5E	NM_002526.2:1214	Endogenous	73.15	120.86	82.44	65.62	72.59	137.31	40.69	26.68	68.33	67.54	42.83	37.45	41.13	21.46	21.03	11.78	43.19	17.49	79.45	36.41	32.42	37.69	53.48	35.07
PAX5	NM_016734.1:2288	Endogenous	262.02	184.47	126.03	200	177.91	306.76	117.28	49.87	119.82	24.56	95.3	43.44	142.47	68.28	130.98	59.9	65.2	60.24	370.41	131.31	80.59	88.79	139.57	165.67
PDCD1	NM_005018.1:175	Endogenous	22.61	15.45	11.37	22.66	14.31	9.74	11.17	22.04	20.59	33.77	12.85	64.41	22.03	26.34	12.43	46.15	22.02	15.54	15.03	19.86	17.6	28.48	11.74	24.19
PDCD1LG2	NM_025239.3:235	Endogenous	33.25	4.54	9.48	7.03	5.11	13.63	9.57	23.2	14.98	51.17	3.21	7.49	8.81	12.68	11.47	11.78	5.93	8.74	6.44	13.24	10.19	4.19	5.22	13.3
PDCD2	NM_144781.2:288	Endogenous	93.1	60.88	61.59	59.37	69.53	49.67	42.29	42.91	40.25	42.98	43.9	56.92	41.13	18.53	40.15	33.39	47.42	34	61.2	46.35	45.39	50.26	36.52	48.37
PDGFB	NM_033016.2:1480	Endogenous	10.64	6.36	2.84	6.25	12.27	4.87	11.17	11.6	8.42	9.21	1.07	20.97	14.69	7.8	3.82	4.91	8.47	5.83	7.52	3.31	4.63	5.03	7.83	10.88
PDGFRB	NM_002609.3:840	Endogenous	158.28	98.14	14.21	67.19	48.06	40.9	12.77	15.08	25.27	9.21	24.63	68.9	51.41	20.48	21.99	17.68	22.02	11.66	38.65	29.79	31.5	61.15	56.09	112.47
PECAM1	NM_000442.3:1365	Endogenous	2568.32	1157.7	2788.78	1570.3	1824.05	2273.92	1233.48	581.08	757.27	940.47	858.74	1056.02	2167.91	1650.35	1215.16	721.76	589.36	984.19	1752.22	1358.36	586.38	835.13	2104	1574.51
PIGR	NM_002644.2:215	Endogenous	3.99	1.82	2.84	3.91	2.04	4.87	6.38	4.64	2.81	8.19	3.21	10.49	7.34	7.8	5.74	1.96	4.23	7.77	7.52	1.1	13.9	7.54	5.22	6.05
PLA2G2A	NM_000300.2:715	Endogenous	5.32	5.45	3.79	3.12	1.02	2.92	8.78	9.28	3.74	6.14	4.28	5.99	5.88	3.9	5.74	6.87	1	6.8	4.29	2.21	8.34	6.7	3.91	14.51
PLA2G2E	NM_014589.1:266	Endogenous	10.64	4.54	6.63	9.37	4.09	4.87	4.79	5.8	7.49	6.14	5.35	11.98	10.28	6.83	7.65	8.84	4.23	9.72	11.81	13.24	8.34	10.05	9.13	13.3
PLAU	NM_002658.2:793	Endogenous	23.94	19.08	15.16	17.97	11.25	9.74	1030.82	1798.91	603.76	210.81	97.44	248.65	402.44	240.92	677.85	1197.04	262.5	602.36	46.17	463.45	1650.75	299.88	217.84	164.47
PLAUR	NM_001005376.1:440	Endogenous	1185.07	273.52	687.96	578.12	366.04	879.38	1646.77	2463.49	1031.53	420.6	1340.57	2083.58	3185.76	1705.94	1368.13	2180.98	829.85	1736.17	1145.6	2400.02	1707.25	1033.65	2780.99	2503.25
PML	NM_002675.3:281	Endogenous	234.09	152.66	235.95	150	138.03	154.84	173.13	286.48	180.66	222.07	168.11	298.08	208.57	221.41	202.69	287.72	185.45	180.71	245.87	186.48	164.89	159.15	221.75	192.28
POU2F2	NM_002698.2:908	Endogenous	400.34	267.16	500.33	302.34	326.16	371.03	91.75	102.07	153.51	137.13	185.24	112.34	287.88	161.91	140.54	56.96	82.14	109.79	303.85	165.52	124.13	113.92	258.27	118.51
PPARG	NM_015869.3:1035	Endogenous	5.32	5.45	6.63	3.91	2.04	1.95	54.25	75.39	62.72	28.65	5.35	34.45	5.88	37.06	65.01	93.29	20.32	37.89	4.29	18.76	42.61	28.48	5.22	49.58
PPBP	NM_002704.2:330	Endogenous	10544.6	4539.03	3993.18	9855.37	17572.8	7909.53	2892.21	9449.2	5827.89	6416.49	6162.13	3585.97	2009.28	5285.6	3446.62	10032.9	18507.3	8563.29	2152.7	4529.7	2309.38	8705.63	1856.17	1800.65
PRDM1	NM_001198.3:798	Endogenous	1189.06	850.56	480.43	679.68	439.65	552.17	1669.9	1961.29	1229.98	1312.98	1051.47	2949.37	906.23	1324.57	915.91	1549.57	1370.1	1086.2	747.27	1262.36	1013.42	1616.65	637.85	1423.35
PRF1	NM_005041.3:2120	Endogenous	4808.12	3817.51	1550.27	4116.36	2894.55	1926.26	696.52	1302.5	1484.59	1718.23	999	2751.64	1172.08	1370.41	1388.21	1012.42	966.18	526.58	2008.83	606.9	833.71	1870.46	1827.47	2964
PRKCD	NM_006254.3:2165	Endogenous	520.05	328.95	637.73	338.28	329.23	358.37	217.02	366.51	251.8	272.21	330.86	346.01	521.41	292.61	334.62	528.31	265.89	297.3	353.24	442.49	297.36	211.92	452.63	292.65
PSMB10	NM_002801.2:221	Endogenous	716.9	429.82	651	414.84	465.21	513.21	263.29	378.11	403.44	508.61	313.73	419.41	509.66	360.89	537.31	306.38	245.57	276.89	474.56	387.31	276.98	239.57	409.58	366.42
PSMB5	NM_001130725.1:716	Endogenous	115.71	109.05	120.34	111.72	127.81	129.52	135.63	144.98	158.19	132.01	70.67	121.33	92.53	115.1	111.86	117.84	117.7	88.41	73.01	103.73	135.25	108.89	54.78	99.16
PSMB7	NM_002799.2:420	Endogenous	1403.2	1174.06	1231.88	1123.43	1079.71	1306.89	1295.71	1450.96	1236.53	1169.71	1408.03	1731.57	1202.92	1388.94	1451.31	1371.83	1264.25	1388.35	1407.57	1408.01	1527.54	1353.63	973.09	1472.93
PSMB8	NM_004159.4:1215	Endogenous	2269.06	1459.4	1884.77	1406.24	1429.38	1667.21	1119.39	1518.23	1357.28	1284.32	1131.78	1751.05	1401.21	1172.41	1287.82	1500.47	1094.89	1016.25	1364.63	1329.67	1323.75	1065.48	1553.54	1283.07
PSMB9	NM_002800.4:455	Endogenous	2229.16	1175.88	1799.49	1044.52	1018.36	1408.17	453.98	552.08	816.24	852.46	366.19	536.25	1088.36	395.03	476.12	491.97	307.38	265.23	959.86	384	364.98	309.09	1183.09	605.86
PSMC2	NM_002803.3:446	Endogenous	719.56	528.87	605.51	535.15	481.57	556.06	660.62	750.41	747.91	680.54	895.14	1229.78	619.82	808.59	734.26	793.44	916.22	791.82	625.95	816.56	842.05	849.37	318.27	724.37
PSMD7	NM_002811.3:580	Endogenous	1756.99	1284.01	1282.1	1214.05	1104.25	1350.72	1649.96	1707.28	1462.12	1276.14	1835.26	2619.83	1573.05	1906.87	1700.84	1783.28	1736.75	1824.58	1504.2	1897.95	2049.07	1882.19	859.6	1694.23
PTAFR	NM_000952.3:1035	Endogenous	293.94	64.52	214.16	180.47	142.12	242.49	236.96	328.23	186.28	333.62	98.51	208.21	293.75	240.92	359.48	303.43	128.71	238.03	107.37	295.73	239.92	124.81	289.58	307.16
PTGER4	NM_000958.2:1380	Endogenous	336.5	209	173.41	271.87	157.46	225.93	264.09	308.52	189.08	128.94	322.29	585.68	277.6	269.21	207.47	247.46	221.01	219.57	206.14	390.62	342.75	336.73	178.7	260
PTGS2	NM_000963.1:495	Endogenous	1327.39	190.83	336.4	444.53	331.27	342.79	1055.56	1225.95	524.19	624.25	106	148.29	1407.08	254.57	166.36	534.2	286.21	370.16	191.11	332.14	348.31	98.84	2141.83	544.19
PTK2	NM_005607.3:1005	Endogenous	204.83	247.17	93.81	175	202.44	201.58	199.46	274.88	90.8	154.53	145.62	139.3	143.94	235.07	178.78	266.12	155.81	266.21	126.69	164.42	155.63	147.43	143.48	117.3
PTPN2	NM_002828.2:185	Endogenous	396.35	279.88	297.55	308.59	280.15	341.82	217.02	286.48	232.14	219	428.3	512.28	345.16	375.52	296.38	238.62	248.95	322.56	299.55	382.9	394.62	352.65	213.92	322.88
PTPN22	NM_015967.4:2505	Endogenous	603.84	593.39	395.15	464.84	399.78	515.16	335.9	421.02	486.75	577.18	305.16	629.12	327.54	486.72	470.38	303.43	382.75	264.26	410.14	215.17	328.85	485.83	228.27	660.28
PTPN6	NM_002831.5:1734	Endogenous	1671.87	1021.4	1869.61	1031.24	1149.23	1315.66	697.32	933.67	691.75	651.88	845.89	906.23	1863.87	871.02	1021.08	1071.34	676.58	835.54	1409.72	1427.88	933.76	698.59	1755.73	1058.14
PTPRC_all	NM_080921.2:90	Endogenous	15044.2	12097.7	11312.4	10400.7	11278.6	11287.8	7916.29	9675.37	8125.91	7807.25	7348.52	9964.03	10426.8	9265.16	8232.68	8630.65	7105.36	6406.44	9806.85	6217.99	6975.37	7825.27	8524.28	12074.9
PYCARD	NM_013258.3:714	Endogenous	142.32	65.43	212.26	85.94	109.4	128.55	20.74	29	19.66	28.65	61.03	77.89	86.66	63.4	26.77	35.35	27.94	30.12	89.11	98.21	28.72	38.53	134.35	58.05
RAF1	NM_002880.2:1990	Endogenous	751.48	537.05	622.57	460.15	508.16	564.83	1000.51	1166.8	728.25	776.73	733.46	1090.47	865.11	1034.88	1091.83	1181.33	752.79	895.77	552.94	800.01	924.49	882.04	640.46	856.19
RAG1	NM_000448.2:2300	Endogenous	7.98	9.09	3.79	7.03	6.13	3.9	15.16	4.64	4.68	10.23	6.42	26.96	2.94	2.93	4.78	3.93	3.39	5.83	6.44	7.72	12.04	5.86	6.52	6.05
RAG2	NM_000536.3:655	Endogenous	7.98	2.73	2.84	1	5.11	1.95	18.35	9.28	2.81	5.12	14.99	11.98	5.88	3.9	8.6	7.86	3.39	6.8	8.59	4.41	15.75	11.73	5.22	3.63
RARRES3	NM_004585.3:640	Endogenous	1077.34	767.86	576.14	646.09	565.41	707.01	369.41	397.82	554.15	455.4	352.27	564.71	446.51	410.64	413.98	266.12	392.06	303.13	624.87	354.21	378.87	411.28	356.1	386.98
RELA	NM_021975.2:360	Endogenous	178.23	104.5	125.08	96.09	99.18	112.97	139.62	189.05	171.3	171.93	176.67	241.16	183.6	189.22	172.09	198.36	165.12	149.62	138.5	164.42	214.91	149.94	109.57	152.37
RELB	NM_006509.2:250	Endogenous	186.21	117.22	126.98	135.94	137.01	150.95	340.68	395.5	291.11	275.28	551.43	618.63	412.72	463.31	391.99	346.64	377.67	460.52	338.2	467.87	717.92	512.64	277.84	261.21
RORC	NM_001001523.1:1350	Endogenous	15.96	30.9	19.9	17.97	16.36	22.4	23.14	60.31	43.99	44	16.06	35.95	13.22	32.19	21.03	30.44	11.01	12.63	26.84	27.59	24.08	20.1	28.7	19.35
RUNX1	NM_001754.4:635	Endogenous	355.12	294.42	292.81	261.72	261.75	261.96	278.45	310.84	348.21	359.2	232.35	420.91	342.22	370.65	356.61	395.74	190.53	315.76	314.58	323.31	302.91	253.81	194.36	354.33
S100A8	NM_002964.3:115	Endogenous	11818.8	4108.3	17049.2	7088.99	6993.55	10930.4	2474.14	3988.68	1647.46	2620.84	8153.72	7248.34	29666.2	4379.47	4851.08	7997.27	3128.87	4009.61	10684	15302.8	5986.96	4381.71	28445.1	14951.8
S100A9	NM_002965.2:75	Endogenous	13631.7	5508.63	20893.6	8390.54	8081.44	12341.5	2679.98	6422.02	1923.6	1921.88	10013.6	11006.6	29757.3	5239.75	6262.23	11679.7	4482.03	5756.47	12425.5	17570.4	6922.57	6695.28	26759.8	16447.7
S1PR1	NM_001400.3:1065	Endogenous	186.21	179.93	142.14	135.15	209.6	174.32	213.82	223.85	245.25	176.02	188.45	220.19	211.5	261.4	239.97	246.48	231.17	248.72	151.39	120.28	276.05	141.56	71.74	162.05
SELE	NM_000450.2:1505	Endogenous	7.98	2.73	8.53	4.69	4.09	6.82	22.34	29	14.98	15.35	7.5	10.49	17.63	10.73	12.43	19.64	10.16	12.63	6.44	9.93	14.82	9.21	10.44	14.51
SELL	NR_029467.1:1585	Endogenous	1480.34	1391.24	2092.3	1477.33	1410.98	1754.86	335.9	385.07	507.34	365.34	555.72	823.85	1664.12	254.57	404.42	517.5	277.75	309.93	1290.54	861.8	395.55	334.22	2210.96	1417.3
SELPLG	NM_003006.3:2297	Endogenous	97.09	46.34	62.54	54.69	72.59	56.48	51.06	62.63	67.4	44	37.48	55.42	48.47	43.89	43.98	44.19	38.95	37.89	22.55	29.79	42.61	37.69	35.22	54.42
SERPING1	NM_000062.2:305	Endogenous	206.16	49.98	217.95	60.16	25.56	59.4	23.14	167.02	23.4	153.5	36.41	242.66	183.6	48.77	94.65	186.58	43.19	81.61	135.28	225.11	57.43	41.04	266.1	105.21
SH2D1A	NM_001114937.2:495	Endogenous	361.77	378.93	232.16	301.56	293.44	294.1	558.5	589.2	555.08	651.88	334.07	444.88	232.07	520.85	514.36	446.8	582.59	327.41	282.37	185.38	418.71	378.61	120.01	354.33
SIGIRR	NM_021805.2:469	Endogenous	540	454.36	368.62	378.9	335.36	334.03	183.51	193.69	238.69	167.83	270.9	337.03	306.97	205.81	188.35	165.96	206.62	152.53	396.18	176.55	263.08	294.85	194.36	347.07
SKI	NM_003036.2:3335	Endogenous	502.76	500.7	415.05	485.15	498.96	383.69	1121.78	1246.83	1028.73	827.9	655.3	1992.21	740.26	1704.97	941.73	1300.15	642.71	1178.5	423.02	700.7	1186.65	1281.59	375.67	961.39
SLAMF1	NM_003037.2:580	Endogenous	147.64	136.31	93.81	104.69	114.51	92.51	98.93	121.78	133.86	110.52	132.77	206.71	108.69	115.1	108.04	104.09	99.07	83.55	132.06	123.59	140.8	149.1	62.61	81.02
SLAMF6	NM_001184714.1:1032	Endogenous	148.97	145.39	98.55	111.72	93.04	126.6	27.13	46.39	73.95	52.19	21.41	35.95	29.38	29.26	27.73	20.62	23.71	14.57	65.49	17.66	25.01	23.45	60	44.74
SLAMF7	NM_021181.3:215	Endogenous	305.91	175.38	152.56	153.12	146.21	170.42	124.46	190.21	253.67	252.77	107.07	278.61	95.47	172.64	190.26	111.95	120.24	61.21	134.21	111.45	176.93	195.17	97.83	113.67
SLC2A1	NM_006516.2:2500	Endogenous	78.47	64.52	36.96	62.5	58.28	54.54	39.09	63.79	61.78	45.03	51.4	104.85	67.56	57.55	75.53	59.9	62.66	40.81	63.35	52.97	85.22	56.12	133.05	100.37
SMAD3	NM_005902.3:4220	Endogenous	512.07	441.64	357.24	351.56	328.21	369.09	836.95	864.08	802.2	878.05	461.49	780.41	353.97	986.11	880.54	732.56	515.69	675.23	398.33	736.01	668.82	545.31	234.79	380.93
SMAD5	NM_005903.5:1044	Endogenous	176.9	186.29	109.92	136.72	112.47	139.26	98.14	97.43	119.82	99.27	104.93	185.74	67.56	117.05	134.81	138.46	165.97	115.62	143.87	109.24	80.59	137.37	57.39	129.4
SOCS1	NM_003745.1:1025	Endogenous	296.6	223.54	183.83	235.15	258.68	170.42	347.07	386.23	247.12	189.32	305.16	609.65	371.6	230.19	195.99	372.17	267.58	302.15	248.02	278.07	296.43	348.46	198.27	309.58
SOCS3	NM_003955.3:1870	Endogenous	707.59	367.12	311.76	440.62	720.83	542.43	1014.07	1164.48	695.49	292.68	515.03	596.16	1373.3	597.91	417.8	1034.03	529.24	857.88	697.88	872.84	406.67	350.97	931.34	882.79
SPP1	NM_000582.2:760	Endogenous	6.65	3.63	5.69	6.25	9.2	7.79	19.15	307.36	302.35	444.14	3.21	19.47	13.22	13.66	21.99	24.55	5.08	5.83	5.37	14.34	12.97	8.38	6.52	19.35
SRC	NM_005417.3:1410	Endogenous	154.29	87.24	180.04	121.87	173.82	109.07	477.12	559.04	497.98	507.59	216.29	319.05	246.75	544.26	540.18	497.87	212.54	457.6	178.23	371.87	398.33	199.36	114.79	147.53
STAT1	NM_007315.2:205	Endogenous	1590.74	578.85	1363.59	717.96	642.1	538.53	1539.85	2223.41	948.23	2268.8	431.51	880.77	1236.7	951.97	2008.69	2117.16	750.25	1248.45	788.07	1345.12	1020.83	398.72	1090.48	654.23
STAT2	NM_005419.2:1965	Endogenous	1119.9	698.8	1525.63	782.8	680.95	716.75	506.64	775.93	505.47	745.01	336.21	582.68	622.76	526.71	635.78	782.64	385.29	478.01	738.68	481.11	411.3	330.03	768.29	407.53
STAT3	NM_139276.2:4535	Endogenous	1803.54	1194.05	1155.12	1034.36	1076.64	1068.3	1768.04	1888.22	1511.73	1666.04	1547.23	2411.62	1697.9	1771.29	1632.96	2300.79	1542.84	1630.27	1216.46	1913.4	1970.33	1793.39	1257.45	1907.07
STAT4	NM_003151.2:789	Endogenous	985.57	1048.66	497.49	809.37	734.12	747.91	1249.44	1500.83	1159.77	1355.96	898.35	1258.24	638.92	1467.95	1188.39	1209.8	1078.8	912.29	819.21	551.73	926.34	1037.84	345.67	1255.26
STAT5A	NM_003152.2:3460	Endogenous	279.31	248.08	313.65	235.15	234.14	226.9	341.48	360.71	316.39	321.34	292.31	417.91	311.38	473.06	381.47	386.9	348.03	363.36	267.34	280.28	460.39	382.8	194.36	281.77
STAT5B	NM_012448.3:200	Endogenous	928.37	825.11	767.55	744.52	730.03	659.29	635.09	734.18	899.55	801.29	439.01	516.78	1035.48	507.2	433.1	468.41	446.26	422.63	731.17	453.52	504.86	392.85	842.64	748.56
STAT6	NM_003153.3:2030	Endogenous	865.86	597.93	845.26	634.37	621.65	701.16	289.62	332.87	437.14	389.9	253.77	280.11	484.69	324.8	361.39	265.14	202.38	205.97	632.39	250.49	252.89	251.29	538.72	495.81
SYK	NM_003177.3:1685	Endogenous	873.84	438	1077.42	564.06	696.29	689.48	441.21	411.74	381.91	581.27	376.9	474.83	665.35	396.01	491.42	352.53	276.9	380.85	533.61	481.11	254.74	300.71	700.46	434.14
TAGAP	NM_054114.3:169	Endogenous	4971.72	2487.15	2608.74	4034.33	2306.65	3142.58	3402.04	5248.26	3188.21	1621.01	3203.67	3371.77	5421.23	2567.21	2562.26	3802.24	2680.92	2127.71	3607.51	4080.59	4335.29	2607.58	5295.88	2867.25
TAL1	NM_003189.2:4635	Endogenous	77.14	79.06	62.54	100.78	160.52	71.09	32.71	41.75	15.91	76.75	51.4	34.45	22.03	51.7	23.9	55.97	89.76	65.09	22.55	41.93	40.76	47.75	76.96	27.81
TAP1	NM_000593.5:2075	Endogenous	434.93	215.37	267.22	176.56	176.88	189.9	346.27	550.92	317.32	499.4	156.33	311.56	282	288.71	443.61	477.24	291.29	291.47	257.68	358.62	314.03	223.65	303.93	250.33
TAP2	NM_000544.3:909	Endogenous	593.2	387.11	293.76	328.9	300.6	260.02	114.09	170.5	182.53	236.4	251.62	226.18	293.75	232.14	314.55	190.5	221.86	142.82	358.6	219.59	213.99	206.9	216.53	285.4
TAPBP	NM_003190.4:1536	Endogenous	1311.43	956.88	962.76	966.4	877.26	941.7	562.49	706.34	768.5	855.53	853.38	1160.87	1252.86	1019.27	769.63	715.87	609.68	699.52	1315.24	933.53	858.72	797.44	917	1146.42
TBK1	NM_013254.2:1610	Endogenous	311.23	211.73	272.91	212.5	187.11	266.83	497.06	613.55	374.42	485.07	345.85	597.66	365.72	646.68	555.47	600.97	411.54	476.06	294.18	414.9	428.9	422.17	226.97	438.98
TBX21	NM_013351.1:890	Endogenous	1657.24	1209.5	465.27	874.99	660.5	785.89	458.76	708.66	858.36	739.89	516.1	1411.02	465.6	907.11	850.9	623.56	765.49	422.63	709.69	387.31	495.59	959.94	410.89	939.63
TCF4	NM_003199.1:1455	Endogenous	156.95	131.76	165.83	130.47	130.87	181.13	114.09	86.99	165.68	112.57	100.65	98.86	148.35	98.51	165.4	59.9	100.77	86.47	144.94	129.1	107.46	76.23	88.7	199.53
TCF7	NM_003202.2:2420	Endogenous	895.12	1580.26	998.77	1192.18	1764.75	1450.05	1223.11	909.31	1090.51	763.43	728.11	465.85	1255.8	796.89	645.34	434.04	878.11	621.8	1232.57	439.18	943.02	417.15	572.63	819.91
TFRC	NM_003234.1:1220	Endogenous	617.14	483.44	455.79	456.25	394.67	421.67	642.27	625.15	518.58	376.6	907.99	1957.76	718.23	687.64	490.46	643.2	543.64	703.41	575.48	1433.39	871.69	1155.11	1729.64	824.74
TGFB1	NM_000660.3:1260	Endogenous	2065.56	1603.88	1245.14	1319.52	1361.9	1192.95	1438.53	1816.31	1276.78	1750.98	1898.43	2889.45	1642.09	2961.26	1743.86	2484.42	1673.24	2196.69	1561.11	1688.29	1601.65	2046.36	1061.78	2227.53
TGFBI	NM_000358.2:2030	Endogenous	811.33	495.25	1464.04	621.87	669.7	799.52	1173.64	949.91	1358.22	2215.58	566.42	645.6	757.89	1889.32	1932.21	983.95	552.95	1250.39	858.93	777.94	612.31	646.66	716.12	798.14
TGFBR1	NM_004612.2:4280	Endogenous	320.54	251.71	254.9	273.43	215.74	252.22	300.79	291.12	252.74	213.88	161.68	232.17	208.57	357.97	302.12	237.64	204.07	190.42	246.94	203.04	285.31	248.78	157.83	203.16
TGFBR2	NM_001024847.1:1760	Endogenous	904.43	909.62	790.3	738.27	877.26	839.45	1440.12	1435.88	1041.83	926.14	811.62	1285.2	1036.95	1284.58	1145.37	1331.57	1138.08	1212.5	945.9	833.11	1380.25	1164.32	586.98	1084.74
THY1	NM_006288.2:135	Endogenous	5.32	1	2.84	1	1.02	2.92	3.19	4.64	1	1.02	1.07	3	1.47	1	5.74	1	2.54	8.74	2.15	3.31	2.78	3.35	1.3	1.21
TICAM1	NM_014261.1:518	Endogenous	234.09	82.69	154.46	128.12	103.27	148.02	137.23	171.66	161.94	164.76	213.08	223.19	226.19	184.35	144.37	163.99	142.26	198.2	228.69	236.14	164.89	177.58	228.27	153.58
TIGIT	NM_173799.2:1968	Endogenous	434.93	246.26	207.52	203.9	256.63	247.36	108.51	95.11	229.33	236.4	91.01	137.81	136.6	95.59	130.98	67.76	79.6	67.04	234.06	54.07	159.33	94.65	74.35	140.28
TIRAP	NM_148910.2:661	Endogenous	37.24	39.07	23.69	27.34	28.63	23.37	27.13	26.68	29.02	34.79	14.99	37.45	36.72	27.31	35.37	22.59	20.32	18.46	31.14	29.79	25.94	21.78	28.7	37.49
TLR1	NM_003263.3:545	Endogenous	771.43	139.94	632.05	407.81	402.85	481.08	399.72	559.04	489.56	730.68	410.1	509.29	909.17	745.19	714.18	825.85	397.99	521.73	427.32	505.38	443.72	411.28	811.34	782.42
TLR2	NM_003264.3:180	Endogenous	524.04	198.1	690.8	253.9	306.73	514.19	626.31	1000.94	546.66	571.04	527.88	1138.4	1257.27	804.69	744.78	1798.01	480.97	919.09	523.95	1307.6	779.06	649.17	1159.61	1150.05
TLR3	NM_003265.2:230	Endogenous	66.5	47.25	31.27	48.44	22.49	40.9	30.32	38.27	30.89	40.93	20.34	53.92	35.25	48.77	50.67	26.51	35.56	22.35	36.5	29.79	36.13	43.56	24.78	54.42
TLR4	NM_138554.2:2570	Endogenous	676.99	176.29	760.92	449.21	419.2	631.05	415.68	504.53	323.88	482	356.56	495.8	923.86	522.81	623.36	655.96	248.11	502.29	409.07	598.07	351.08	255.48	774.82	887.63
TLR5	NM_003268.3:215	Endogenous	67.83	31.81	73.91	39.84	36.81	51.61	22.34	23.2	18.72	24.56	25.7	43.44	45.53	17.56	31.55	20.62	22.02	22.35	20.4	45.24	27.79	14.24	46.96	16.93
TLR7	NM_016562.3:4120	Endogenous	49.21	23.63	77.7	17.97	48.06	57.46	19.15	20.88	20.59	13.3	10.71	16.48	22.03	19.51	15.3	15.71	16.09	10.69	37.58	22.07	19.45	14.24	27.39	37.49
TLR8	NM_016610.2:2310	Endogenous	262.02	79.06	432.1	142.97	180.97	192.82	132.44	135.7	58.04	84.94	52.47	70.4	136.6	178.49	177.83	132.57	41.49	78.7	68.71	98.21	71.33	45.23	221.75	162.05
TLR9	NM_017442.2:985	Endogenous	18.62	10.9	25.59	14.06	21.47	21.42	14.36	13.92	14.98	11.26	18.2	19.47	14.69	19.51	9.56	16.69	13.55	14.57	12.88	15.45	12.97	11.73	20.87	29.02
TMEM173	NM_198282.1:725	Endogenous	796.7	632.47	872.74	578.12	560.3	631.05	342.28	312	475.52	340.78	351.2	408.93	403.91	348.21	304.99	301.47	239.64	253.58	399.4	326.62	267.71	283.96	324.8	412.37
TNF	NM_000594.2:1010	Endogenous	300.59	179.93	235	153.12	156.43	203.53	1128.96	1220.15	678.64	669.28	762.37	1601.26	684.45	690.57	189.3	192.47	370.04	474.12	615.21	1298.77	668.82	655.04	266.1	275.72
TNFAIP3	NM_006290.2:260	Endogenous	6199.35	4947.04	2299.82	5256.2	2355.72	3460.05	10178.2	12601.6	8008.9	6469.71	9292.99	18548.5	7055.97	8278.07	4404.6	8232.94	6474.51	7686.95	5002.2	7933.87	8937.37	10965.6	5024.56	7544.83
TNFAIP6	NM_007115.2:250	Endogenous	15.96	6.36	13.27	7.03	10.22	14.61	35.9	77.71	45.87	31.72	7.5	31.46	44.06	13.66	10.52	46.15	23.71	19.43	21.47	19.86	20.38	10.89	87.4	33.86
TNFRSF10C	NM_003841.3:682	Endogenous	762.12	102.68	108.03	126.56	133.94	116.86	23.14	60.31	27.15	17.4	35.33	73.4	1009.05	22.43	22.95	149.26	58.43	44.69	164.27	123.59	67.62	32.67	1123.09	345.86
TNFRSF11A	NM_003839.2:490	Endogenous	18.62	11.81	11.37	17.97	14.31	17.53	19.15	30.16	68.33	68.57	20.34	19.47	24.97	28.29	41.11	26.51	16.94	18.46	18.25	16.55	17.6	12.56	13.04	30.23
TNFRSF13B	NM_012452.2:160	Endogenous	41.23	22.72	58.75	21.87	38.85	46.74	7.98	11.6	19.66	8.19	10.71	11.98	10.28	1	4.78	2.95	7.62	9.72	25.77	16.55	7.41	4.19	14.35	7.26
TNFRSF13C	NM_052945.3:789	Endogenous	486.8	455.27	286.17	475	401.82	557.04	655.04	378.11	606.56	203.65	537.51	399.94	343.69	423.32	618.57	362.35	525.01	314.78	514.29	748.15	543.76	559.55	292.19	667.53
TNFRSF14	NM_003820.2:916	Endogenous	542.66	434.37	439.69	380.46	357.86	362.27	235.37	324.75	238.69	254.82	328.72	474.83	423.01	324.8	336.54	317.18	257.42	303.13	370.41	390.62	359.42	311.6	330.01	314.42
TNFRSF17	NM_001192.2:635	Endogenous	122.36	32.71	112.76	32.03	104.29	158.74	73.4	38.27	18.72	16.37	39.62	67.41	26.44	19.51	20.08	25.53	12.7	12.63	42.95	60.69	36.13	23.45	53.48	50.79
TNFRSF1B	NM_001066.2:835	Endogenous	611.82	353.49	623.52	439.06	389.55	458.68	469.93	651.83	443.69	496.33	501.11	891.25	769.64	954.9	675.94	721.76	344.64	574.19	477.78	702.9	563.22	608.97	521.76	966.23
TNFRSF4	NM_003327.2:200	Endogenous	33.25	19.99	20.85	23.44	20.45	3.9	31.12	19.72	24.34	28.65	17.13	28.46	16.16	29.26	6.69	24.55	9.31	27.2	21.47	16.55	20.38	14.24	20.87	53.21
TNFRSF8	NM_152942.2:2030	Endogenous	46.55	34.53	75.81	38.28	40.9	41.88	43.08	44.07	16.85	21.49	26.77	41.94	39.66	51.7	30.59	24.55	13.55	30.12	24.69	36.41	25.94	33.51	48.26	44.74
TNFRSF9	NM_001561.4:255	Endogenous	27.93	10	14.21	10.94	7.16	18.5	47.87	30.16	46.8	53.21	17.13	40.44	26.44	10.73	15.3	11.78	17.78	9.72	32.21	18.76	137.1	30.99	13.04	25.4
TNFSF10	NM_003810.2:115	Endogenous	525.37	219.91	819.67	267.97	225.96	246.38	120.48	233.13	125.43	183.18	131.7	253.15	301.1	208.73	211.29	233.71	181.21	218.6	197.55	125.79	217.69	139.89	357.41	180.19
TNFSF11	NM_003701.2:490	Endogenous	15.96	3.63	7.58	8.59	8.18	4.87	4.79	6.96	2.81	5.12	5.35	14.98	10.28	9.75	11.47	10.8	8.47	7.77	8.59	9.93	5.56	10.89	15.65	8.47
TNFSF12	NM_003809.2:339	Endogenous	319.21	229	321.24	264.84	255.61	262.94	107.71	97.43	107.65	134.06	175.6	227.68	220.32	132.65	112.82	86.41	116.86	106.87	254.46	182.07	113.94	139.05	173.49	195.91
TNFSF13B	NM_006573.4:1430	Endogenous	602.51	245.35	908.75	383.59	357.86	511.27	291.22	456.98	317.32	800.27	189.52	337.03	531.69	294.57	487.59	402.61	110.08	206.94	431.61	272.55	100.05	133.19	808.73	497.02
TNFSF15	NM_001204344.1:2338	Endogenous	19.95	9.09	8.53	4.69	8.18	7.79	37.5	33.64	36.51	55.26	11.78	22.47	27.91	13.66	13.38	10.8	12.7	18.46	16.1	27.59	36.13	14.24	19.57	14.51
TNFSF4	NM_003326.2:545	Endogenous	156.95	86.33	71.07	139.06	222.89	135.36	62.23	56.83	73.01	406.28	76.02	67.41	44.06	116.07	55.45	126.68	116.01	107.84	34.36	68.41	49.1	102.19	35.22	30.23
TNFSF8	NM_001244.3:518	Endogenous	164.93	246.26	304.18	214.84	164.61	251.25	391.75	568.32	319.2	376.6	341.57	632.11	229.13	277.98	293.51	515.54	306.54	341.02	191.11	388.42	393.7	430.55	200.88	490.98
TOLLIP	NM_019009.2:1320	Endogenous	238.08	209	252.06	166.4	190.18	201.58	169.14	249.37	210.61	215.93	129.56	194.73	183.6	190.2	181.65	244.51	171.9	247.75	146.02	167.73	192.68	165.85	155.22	154.79
TP53	NM_000546.2:1330	Endogenous	381.72	340.77	461.48	321.09	344.57	389.54	120.48	112.5	131.05	126.9	138.13	191.73	240.88	123.87	142.45	75.61	134.64	99.1	310.29	107.04	169.52	153.29	210.01	186.23
TRAF1	NM_005658.3:3735	Endogenous	247.39	287.15	217.95	167.19	218.8	207.43	249.73	223.85	289.24	273.24	297.67	199.22	296.69	198.98	149.15	107.04	221.01	199.17	245.87	180.97	359.42	230.35	126.53	216.46
TRAF2	NM_021138.3:1325	Endogenous	107.73	82.69	63.49	104.69	82.82	86.67	89.36	114.82	142.28	104.38	67.46	83.88	66.09	121.92	106.12	76.59	79.6	95.21	53.68	60.69	105.6	70.36	49.57	68.93
TRAF3	NM_145725.1:1795	Endogenous	219.46	201.73	237.85	167.19	199.38	259.04	465.95	494.09	490.49	460.51	354.42	437.39	279.07	536.46	472.3	455.64	322.62	389.59	246.94	391.73	469.66	366.05	173.49	247.91
TRAF4	NM_004295.2:1060	Endogenous	240.74	159.93	108.97	154.69	103.27	124.65	215.42	169.34	182.53	104.38	416.52	380.47	362.79	309.2	266.74	203.27	315	249.69	410.14	379.59	452.06	325.84	193.05	307.16
TRAF5	NM_004619.3:400	Endogenous	182.22	184.47	173.41	153.9	180.97	199.64	112.5	117.14	149.77	155.55	81.38	97.36	120.44	81.93	130.02	75.61	84.68	76.75	143.87	41.93	70.4	67.85	65.22	85.86
TRAF6	NM_145803.1:1839	Endogenous	642.41	525.24	367.67	453.9	431.47	440.18	454.78	482.49	343.53	380.69	560	692.03	575.76	600.84	509.58	517.5	618.15	583.9	567.97	497.66	465.95	534.42	283.06	587.72
TYK2	NM_003331.3:485	Endogenous	408.32	358.94	483.27	350	350.7	397.33	350.26	364.19	362.25	409.35	326.58	332.53	353.97	305.29	392.94	331.91	251.49	304.1	397.26	272.55	231.59	306.58	353.49	366.42
UBE2L3	NM_198157.1:990	Endogenous	180.89	106.32	155.41	122.66	103.27	120.76	45.48	44.07	52.42	41.96	56.75	68.9	70.5	48.77	42.07	49.1	55.89	55.38	103.07	29.79	44.46	40.21	74.35	61.67
VCAM1	NM_001078.3:2535	Endogenous	5.32	19.99	4.74	7.03	10.22	3.9	15.16	25.52	23.4	11.26	5.35	28.46	8.81	17.56	10.52	2.95	3.39	5.83	5.37	13.24	24.08	50.26	5.22	15.72
VTN	NM_000638.3:1346	Endogenous	6.65	8.18	6.63	2.34	5.11	12.66	9.57	10.44	2.81	8.19	6.42	11.98	11.75	1.95	5.74	6.87	10.16	6.8	8.59	6.62	12.97	10.05	13.04	3.63
XBP1	NM_005080.2:440	Endogenous	591.87	543.41	550.55	563.28	512.25	530.74	521.8	443.06	503.6	507.59	606.04	769.92	461.19	670.09	462.74	412.43	380.21	437.2	591.59	513.11	557.66	479.13	340.45	520
XCL1	NM_002995.1:620	Endogenous	25.27	17.27	17.06	28.12	10.22	17.53	35.9	46.39	17.79	18.42	24.63	67.41	19.09	17.56	15.3	20.62	14.4	16.52	32.21	18.76	48.17	32.67	16.96	32.65
XCR1	NM_005283.2:700	Endogenous	1.33	1	8.53	7.03	2.04	4.87	5.58	6.96	4.68	7.16	2.14	4.49	2.94	4.88	3.82	1.96	6.77	1.94	4.29	3.31	4.63	5.86	5.22	1.21

ZAP70	NM_001079.3:1175	Endogenous	1139.85	1310.37	700.27	942.96	886.46	860.87	652.64	708.66	706.72	598.67	708.83	928.7	807.82	706.18	667.33	629.45	736.7	533.38	873.96	390.62	704.02	834.29	417.41	879.16
ZBTB16	NM_006006.4:1585	Endogenous	148.97	154.48	123.19	221.87	131.9	131.47	153.19	213.41	100.16	93.13	182.03	633.61	85.19	132.65	135.76	282.81	160.04	129.22	102	187.59	121.35	356.84	138.27	327.72
ZEB1	NM_001128128.1:1450	Endogenous	97.09	79.97	71.07	79.69	79.75	68.17	235.37	303.88	227.46	146.34	126.35	166.27	173.31	209.71	195.99	262.19	195.61	189.45	91.26	134.62	274.2	170.04	67.83	151.16
sCTLA4	NM_001037631.1:560	Endogenous	119.7	69.97	44.54	73.44	48.06	55.51	63.83	93.95	58.04	40.93	84.59	103.36	69.03	34.14	43.02	63.83	53.35	36.92	51.54	54.07	46.32	40.21	43.05	79.81
ABCF1	NM_001090.2:850	Housekeeping	441.58	315.32	318.39	345.31	303.67	380.77	353.45	455.82	407.18	324.41	547.15	594.67	505.26	412.59	354.7	417.34	447.1	446.92	475.63	452.42	687.35	515.15	316.97	371.26
ALAS1	NM_000688.4:1615	Housekeeping	75.81	48.16	102.34	57.81	38.85	59.4	138.03	148.46	134.79	165.79	77.09	104.85	76.38	88.76	163.49	155.15	85.53	99.1	71.94	114.76	132.47	89.63	75.66	64.09
EEF1G	NM_001404.4:1150	Housekeeping	9661.47	11198.1	9754.57	10731.1	11679.4	10353.9	10808.5	9597.66	9507.52	7802.13	10780.3	5699.51	10694.1	9391.96	9909.62	8821.15	11263.9	9907.92	10311.5	9703.82	10921.6	10116.2	6467.23	9685.3
G6PD	NM_000402.2:1155	Housekeeping	536.01	339.86	523.07	385.93	393.64	410.96	104.52	135.7	189.08	207.74	237.71	283.1	377.47	159.96	174	145.33	178.67	153.51	390.81	226.21	153.77	219.46	485.24	362.79
GAPDH	NM_002046.3:972	Housekeeping	9303.68	5753.07	8995.54	7425.71	6742.03	8837.6	10001.9	11973	11367.5	14955.5	8761.9	12312.7	8963.91	12128.9	10816	13182.1	7439.84	10933.9	7643.42	12034.3	8580.73	10412.8	7289.01	10915.2
GUSB	NM_000181.1:1350	Housekeeping	243.4	178.11	258.69	235.15	167.68	218.14	275.26	395.5	392.21	291.66	241.99	411.92	204.16	316.02	412.06	314.23	215.93	351.7	214.73	270.35	258.45	258.83	147.4	262.42
HPRT1	NM_000194.1:240	Housekeeping	430.94	361.67	357.24	324.22	321.05	348.63	280.05	271.4	374.42	310.08	298.74	405.93	296.69	237.02	259.09	199.34	207.46	207.91	347.87	257.11	251.04	261.34	285.66	347.07
OAZ1	NM_004152.2:313	Housekeeping	6168.76	5503.18	6427.55	4731.2	5028.41	5291.85	2096.75	3283.5	3484.94	3976.79	3346.08	3331.33	4688.32	2738.87	2866.29	3687.35	2488.7	3317.86	4068.11	4097.14	2088.91	2724.02	18077.7	3614.6
POLR1B	NM_019014.3:3320	Housekeeping	126.35	101.78	108.97	82.03	91	146.08	93.35	78.87	100.16	72.66	88.87	103.36	47	99.49	81.27	54.99	72.82	80.64	138.5	88.28	103.75	77.06	45.65	116.09
POLR2A	NM_000937.2:3775	Housekeeping	751.48	621.56	576.14	623.43	541.9	554.11	852.9	872.2	745.1	823.81	781.64	1038.04	776.98	1254.34	914	930.92	792.59	889.94	780.55	689.66	822.59	964.13	469.59	991.63
PPIA	NM_021130.2:925	Housekeeping	319.21	349.86	289.96	321.09	313.89	309.68	409.3	470.89	449.31	384.78	379.04	413.42	342.22	362.84	387.21	370.21	456.42	376.96	357.53	313.38	370.54	332.54	186.53	397.86
RPL19	NM_000981.3:315	Housekeeping	18634	22067.2	19129.2	21664.6	21264.9	19893.6	21625.8	19246.3	19698.4	17622.3	21505.9	22074.6	19973.8	19693	20538.2	18837.4	23283.2	20232.6	22139	18883.5	22622.3	20994.7	13559.3	19798.7
SDHA	NM_004168.1:230	Housekeeping	348.47	345.31	277.65	297.65	303.67	295.07	341.48	415.22	275.2	338.73	322.29	425.4	309.91	360.89	395.81	303.43	325.17	358.5	361.82	295.73	277.9	284.8	170.88	359.16
TBP	NM_001172085.1:587	Housekeeping	327.19	349.86	270.07	272.65	274.02	335	266.48	347.95	322.94	277.33	305.16	362.49	292.29	323.83	292.56	292.63	359.04	298.27	235.13	226.21	353.86	329.19	151.31	327.72
TUBB	NM_178014.2:320	Housekeeping	666.35	501.61	645.31	536.71	570.53	599.89	386.96	342.15	585.97	480.98	360.84	473.34	486.16	466.23	470.38	323.07	418.31	378.91	498.18	381.8	409.44	454.84	306.53	420.84
NEG_A	ERCC_00096.1:230	Negative	5	12	9	17	2	9	7	8	15	8	7	8	4	14	12	7	10	9	11	10	7	10	8	11
NEG_B	ERCC_00041.1:440	Negative	7	9	5	9	4	7	13	10	9	16	8	12	11	9	11	14	13	14	6	12	9	6	9	11
NEG_C	ERCC_00019.1:140	Negative	4	6	3	1	4	1	1	5	2	4	3	3	2	4	4	1	4	2	2	4	3	4	5	2
NEG_D	ERCC_00076.1:355	Negative	2	6	1	6	7	4	7	4	2	2	1	4	4	4	5	5	10	2	4	7	5	5	3	4
NEG_E	ERCC_00098.1:785	Negative	9	8	5	8	13	6	6	9	8	3	6	7	8	4	5	7	10	2	7	11	10	6	8	11
NEG_F	ERCC_00126.1:220	Negative	12	13	9	18	13	8	10	11	11	10	15	12	5	12	8	9	11	8	13	7	10	6	7	12
NEG_G	ERCC_00144.1:15	Negative	1	3	4	2	3	1	2	4	4	2	1	4	1	2	5	1	2	3	3	3	3	2	2	1
NEG_H	ERCC_00154.1:115	Negative	5	3	4	5	7	5	4	8	7	9	2	9	12	7	6	13	9	5	9	5	7	5	6	8
POS_A	ERCC_00117.1:385	Positive	17236	21893	18732	17653	20573	22209	12947	14034	18611	16581	19551	16214	18483	15771	18219	16869	19268	16989	18214	17114	19476	17631	18171	20149
POS_B	ERCC_00112.1:695	Positive	5596	7451	6084	5850	6818	7551	4474	4772	6214	5625	6376	5361	6036	5059	5924	5713	6415	5755	6103	5469	6619	5864	6260	6786
POS_C	ERCC_00002.1:850	Positive	1473	1893	1610	1435	1765	1835	1184	1249	1660	1509	1780	1465	1583	1368	1587	1481	1677	1495	1624	1572	1741	1535	1663	1905
POS_D	ERCC_00092.1:540	Positive	356	449	366	356	411	409	270	314	402	308	346	301	326	289	361	329	400	344	378	346	394	346	348	397
POS_E	ERCC_00035.1:485	Positive	44	60	66	52	68	59	32	51	63	52	47	54	57	49	76	47	61	45	57	71	68	61	60	67
POS_F	ERCC_00034.1:195	Positive	26	33	28	23	32	27	13	28	32	25	28	25	28	21	23	23	32	27	30	24	27	21	19	31

Supplementary table 3. Human miRNA gene expression level based on NanoString n-Counter analysis

Probe Name	NS Probe ID	Class Name	Normalized expression level																					
		Group	HC-1	HC-2	HC-3	HC-4	HC-5	HC-6	Mabc-1	Mabc-2	Mabc-3	Mabc-4	Mabc-5	Mabc-6	Mabc-7	Mmass-1	Mmass-2	Mmass-3	Mmass-4	Mmass-5	Mmass-6	Mmass-7	Mmass-8	Mmass-9	Mmass-10
hsa-let-7a-5p	nmiR03113.1:1	Endogenous	7077.15	6675.92	6410.58	6389.46	5515.44	5729.53	5078.54	5154.83	6087.9	7404.88	7759.81	7674.71	7179.35	7489.05	5625.99	4113.83	5376.64	5280.35	6369.13	5344.22	4868.42	5935.68	4730.16
hsa-let-7b-5p	nmiR03114.1:1	Endogenous	1105.32	878.38	1060.31	959.39	722.04	1002.95	803.66	1079.97	1041.05	867.89	959.73	1175.38	989.94	960.26	866.25	699.81	588.32	625.26	866.98	679.23	856.8	609.7	958.56
hsa-let-7c-5p	nmiR03115.1:1	Endogenous	81.46	99.48	67.62	52.53	62.7	115.83	74.2	88.97	69.12	72.05	90.92	94.56	90.36	78.46	76.3	58.86	66.69	73.47	76.76	69.11	85.52	66.05	119.82
hsa-let-7d-5p	nmiR03116.1:1	Endogenous	955.75	902.26	921.01	812.85	982.95	844.74	671.36	759.67	784.75	933.39	937.28	943.68	799.26	893.63	677.43	765.21	882.87	853.48	643.46	855.25	619.24	804.04	716.2
hsa-let-7e-5p	nmiR03117.1:1	Endogenous	31.6	31.83	37.87	26.27	32.36	33.9	28.61	30.11	34.56	57.31	32.55	11.27	30.56	43.59	32.37	45.06	30.17	28.14	18.06	42.11	11.09	27.94	19.06
hsa-let-7f-5p	nmiR03118.1:1	Endogenous	300.56	296.44	228.56	261.27	208.32	276.87	168.06	195.74	237.58	322.59	390.63	403.9	447.8	467.05	270.51	207.11	300.91	379.85	302.54	309.92	248.65	347.4	344.03
hsa-let-7g-5p	nmiR03119.1:1	Endogenous	6386.15	7856.71	5687.02	6237.4	5046.22	5548.72	4986.47	3799.74	5137.56	5249.9	7146.92	7688.49	6166.83	5851.25	4411.39	3055.03	5252.78	3912.58	6807.13	3889.65	5064.81	5247.23	4731.07
hsa-let-7i-5p	nmiR03120.1:1	Endogenous	1122.18	1111.16	895.32	804.56	758.45	926.67	646.33	650.17	891.3	1038.19	1088.82	1345.08	920.17	1180.71	691.3	664.2	874.93	923.83	948.26	859.57	662	727.83	800.61
hsa-miR-1-3p	nmiR03122.1:1	Endogenous	9.13	54.71	22.99	41.47	22.25	22.6	5.36	2.74	12.96	11.46	25.82	19.41	13.29	16.19	8.48	10.9	8.73	15.63	13.55	32.4	6.33	17.15	8.17
hsa-miR-1-5p	nmiR04032.1:1	Endogenous	3.51	2.98	4.06	1.38	18.2	2.83	1	10.95	2.88	6.55	5.61	2.5	5.98	1.87	1.54	2.18	2.38	7.82	11.29	5.4	6.33	3.18	3.63
hsa-miR-100-5p	nmiR03180.1:1	Endogenous	9.83	9.95	13.52	9.68	14.16	19.78	5.36	6.84	7.2	24.56	7.86	4.38	10.63	6.23	6.17	11.63	13.5	12.51	11.29	6.48	12.67	6.35	8.17
hsa-miR-101-3p	nmiR03181.1:1	Endogenous	6.32	5.97	12.17	4.15	10.11	22.6	10.73	20.53	10.08	8.19	14.59	5.01	9.3	6.85	6.17	10.9	11.12	6.25	22.58	5.4	19	6.99	15.43
hsa-miR-103a-3p	nmiR03182.1:1	Endogenous	1.4	7.96	5.41	2.76	12.14	8.48	4.47	5.48	8.64	6.55	10.1	2.5	3.32	4.36	3.85	5.09	6.35	12.51	4.52	11.88	14.25	3.18	5.45
hsa-miR-105-5p	nmiR03183.1:1	Endogenous	9.13	19.9	12.17	5.53	14.16	14.13	12.52	23.27	21.6	14.74	12.35	5.01	12.62	6.85	10.02	22.53	16.67	20.32	36.12	12.96	20.59	13.97	19.06
hsa-miR-106a-5p+hsa-miR-17-5p	nmiR03184.1:1	Endogenous	200.14	203.93	194.75	193.54	210.34	177.99	144.82	184.79	231.82	225.98	268.28	253.61	252.47	250.96	200.38	218.01	227.07	195.39	171.59	197.61	197.97	209.58	216.04
hsa-miR-106b-5p	nmiR03185.1:1	Endogenous	134.13	100.47	136.6	106.44	141.58	124.31	132.3	156.04	113.75	132.64	121.23	154.05	158.79	147.59	127.16	183.13	118.3	132.87	119.66	119.86	93.44	140.36	178.82
hsa-miR-107	nmiR03186.1:1	Endogenous	219.8	191	210.98	226.71	335.74	228.84	157.34	183.42	231.82	203.05	198.68	225.43	194.67	277.12	235.06	192.57	281.06	297	153.53	254.85	164.71	273.09	161.58
hsa-miR-10a-5p	nmiR03125.1:1	Endogenous	8.43	24.87	5.41	30.41	22.25	14.13	3.58	9.58	15.84	19.65	16.84	21.92	9.97	16.81	10.79	10.9	13.5	9.38	20.32	9.72	11.09	15.88	18.15
hsa-miR-10b-5p	nmiR03126.1:1	Endogenous	2.81	4.97	5.41	6.91	4.05	11.3	6.26	10.95	7.2	11.46	4.49	6.26	4.65	3.11	9.25	5.09	4.76	9.38	13.55	7.56	7.92	6.35	9.98
hsa-miR-1178-3p	nmiR03724.1:1	Endogenous	1.4	7.96	5.41	4.15	14.16	19.78	6.26	6.84	10.08	8.19	6.73	1.88	3.32	2.49	5.39	3.63	7.15	6.25	15.8	10.8	11.09	3.81	4.54
hsa-miR-1180-3p	nmiR03726.1:1	Endogenous	4.92	3.98	9.47	2.76	8.09	8.48	10.73	23.27	11.52	4.91	3.37	3.13	7.31	8.1	7.71	13.08	4.76	9.38	13.55	4.32	20.59	3.18	9.98
hsa-miR-1183	nmiR03729.1:1	Endogenous	9.83	8.95	4.06	13.82	10.11	42.38	59.89	108.13	24.48	32.75	12.35	12.52	22.59	14.95	24.66	63.22	13.5	21.88	29.35	21.6	26.92	10.8	35.4
hsa-miR-1185-1-3p	nmiR03986.1:1	Endogenous	3.51	2.98	1.35	1.38	8.09	8.48	1	8.21	5.76	6.55	4.49	2.5	3.99	1.87	2.31	2.91	3.97	4.69	2.26	4.32	17.42	2.54	8.17
hsa-miR-1185-2-3p	nmiR03977.1:1	Endogenous	4.21	2.98	4.06	5.53	4.05	11.3	5.36	12.32	2.88	6.55	8.98	4.38	2.66	5.6	3.85	7.27	3.18	20.32	4.52	3.24	11.09	3.81	3.63
hsa-miR-1185-5p	nmiR03731.1:1	Endogenous	2.11	1.99	5.41	8.29	2.02	2.83	1	4.11	2.88	9.83	4.49	1.88	1.99	3.11	2.31	4.36	3.97	4.69	9.03	8.64	7.92	5.72	3.63
hsa-miR-1193	nmiR03732.1:1	Endogenous	21.07	30.84	25.7	15.21	20.23	33.9	17.88	16.43	18.72	18.01	26.94	16.28	24.58	18.68	14.64	14.53	26.99	14.07	27.09	17.28	38.01	28.58	22.69
hsa-miR-1197	nmiR03733.1:1	Endogenous	4.21	5.97	5.41	12.44	12.14	14.13	9.83	9.58	10.08	13.1	7.86	6.26	5.32	3.74	5.39	4.36	4.76	18.76	9.03	8.64	15.84	2.54	5.45
hsa-miR-1200	nmiR03734.1:1	Endogenous	2.11	1.99	1.35	4.15	6.07	5.65	1	2.74	1.44	6.55	5.61	1	1.99	1.25	1	1.45	1	10.94	9.03	5.4	3.17	1.91	2.72
hsa-miR-1202	nmiR03735.1:1	Endogenous	9.13	10.94	2.7	8.29	16.18	14.13	7.15	8.21	7.2	3.28	3.37	3.13	5.98	2.49	2.31	4.36	1	9.38	15.8	6.48	9.5	2.54	4.54
hsa-miR-1203	nmiR03736.1:1	Endogenous	6.32	12.93	8.11	9.68	14.16	22.6	8.94	4.11	11.52	14.74	8.98	2.5	5.98	5.6	3.08	9.45	9.53	15.63	13.55	6.48	11.09	5.08	8.17
hsa-miR-1204	nmiR03737.1:1	Endogenous	1	1.99	6.76	4.15	4.05	2.83	8.94	9.58	5.76	9.83	5.61	1.25	5.32	1	1.54	3.63	3.18	6.25	13.55	2.16	3.17	3.81	5.45
hsa-miR-1205	nmiR03738.1:1	Endogenous	2.11	2.98	4.06	1.38	6.07	8.48	3.58	2.74	7.2	4.91	2.24	1.25	2.66	5.6	1.54	3.63	4.76	7.82	4.52	8.64	3.17	4.45	4.54
hsa-miR-1206	nmiR03739.1:1	Endogenous	4.92	3.98	5.41	4.15	4.05	8.48	2.68	2.74	7.2	3.28	4.49	3.13	3.32	3.11	3.85	5.81	3.18	4.69	6.77	6.48	9.5	5.72	6.35
hsa-miR-122-5p	nmiR03187.1:1	Endogenous	1	1	1.35	1.38	2.02	2.83	1	1.37	1.44	1.64	1.12	1	1	1	1	1	1	1.56	2.26	1.08	1.58	1	1
hsa-miR-1224-3p	nmiR03742.1:1	Endogenous	2.11	2.98	1.35	1.38	8.09	5.65	4.47	6.84	1.44	6.55	7.86	2.5	1.33	1.25	2.31	3.63	3.18	3.13	9.03	6.48	11.09	5.08	4.54
hsa-miR-1224-5p	nmiR03452.1:1	Endogenous	6.32	6.96	4.06	2.76	6.07	11.3	5.36	10.95	4.32	4.91	6.73	3.76	3.99	1.25	3.85	6.54	4.76	6.25	6.77	6.48	12.67	1.91	6.35
hsa-miR-1226-3p	nmiR03745.1:1	Endogenous	4.92	8.95	1.35	6.91	4.05	16.95	1.79	10.95	11.52	8.19	4.49	3.76	5.32	4.36	6.94	2.91	3.97	15.63	13.55	10.8	12.67	5.08	9.98
hsa-miR-1228-3p	nmiR03747.1:1	Endogenous	5.62	2.98	5.41	6.91	10.11	5.65	3.58	8.21	4.32	8.19	3.37	4.38	1	3.74	1	2.91	2.38	1.56	6.77	5.4	7.92	1.27	6.35
hsa-miR-1233-3p	nmiR03750.1:1	Endogenous	1	3.98	2.7	1.38	4.05	11.3	2.68	4.11	1.44	1.64	3.37	1.88	1.99	3.11	3.08	2.91	1	4.69	2.26	2.16	6.33	1.27	3.63
hsa-miR-1234-3p	nmiR03751.1:1	Endogenous	4.21	9.95	5.41	4.15	10.11	22.6	4.47	10.95	5.76	8.19	3.37	6.89	3.32	5.6	8.48	7.99	6.35	14.07	15.8	9.72	12.67	6.35	6.35
hsa-miR-1236-3p	nmiR03752.1:1	Endogenous	9.83	15.92	14.88	9.68	14.16	31.08	5.36	10.95	7.2	8.19	14.59	1.25	7.31	6.23	10.02	5.81	6.35	23.45	27.09	12.96	22.17	8.26	13.62
hsa-miR-124-3p	nmiR03188.1:1	Endogenous	4.21	4.97	5.41	2.76	6.07	14.13	3.58	12.32	8.64	8.19	3.37	3.76	2.66	2.49	3.08	6.54	4.76	10.94	6.77	5.4	15.84	2.54	4.54
hsa-miR-1244	nmiR03757.1:1	Endogenous	11.24	10.94	2.7	11.06	4.05	14.13	12.52	20.53	12.96	16.38	23.57	10.02	15.95	8.72	6.17	14.53	6.35	9.38	22.58	8.64	19	16.51	9.98
hsa-miR-1245a	nmiR03760.1:1	Endogenous	3.51	4.97	1.35	4.15	14.16	14.13	6.26	5.48	5.76	3.28	7.86	1	2.66	1.25	3.08	4.36	3.97	6.25	9.03	4.32	6.33	6.35	6.35
hsa-miR-1245b-3p	nmiR03758.1:1	Endogenous	3.51	2.98	2.7	1.38	6.07	11.3	8.05	6.84	1.44	3.28	4.49	1.88	7.31	6.85	5.39	6.54	1.59	7.82	13.55	6.48	12.67	3.81	6.35
hsa-miR-1245b-5p	nmiR03759.1:1	Endogenous	3.51	1	8.11	1.38	6.07	8.48	1.79	2.74	7.2	3.28	6.73	3.13	1.99	3.74	3.85	5.09	5.56	4.69	6.77	6.48	9.5	5.08	2.72
hsa-miR-1246	nmiR03503.1:1	Endogenous	1	1	1.35	1.38	2.02	2.83	1	1.37	1.44	1.64	1.12	5.64	1	1	1	1	1	1.56	2.26	1.08	1.58	1	1
hsa-miR-1247-5p	nmiR03761.1:1	Endogenous	1	2.98	1.35	1.38	14.16	2.83	4.47	2.74	1.44	6.55	3.37	1.25	1.33	4.98	3.85	5.09	1	4.69	2.26	2.16	4.75	1.27	2.72
hsa-miR-1248	nmiR03762.1:1	Endogenous	4.21	4.97	4.06	5.53	4.05	11.3	2.68	15.06	1.44	3.28	2.24	1.88	2.66	3.74	5.39	15.26	3.18	6.25	11.29	6.48	6.33	1.91	8.17
hsa-miR-1249-3p	nmiR03763.1:1	Endogenous	3.51	4.97	12.17	2.76	10.11	16.95	1.79	8.21	8.64	3.28	4.49	1.88	2.66	4.36	5.39	2.91	3.97	7.82	4.52	5.4	9.5	6.99	5.45
hsa-miR-1249-5p	nmiR04035.1:1	Endogenous	3.51	2.98	5.41	5.53	4.05	16.95	2.68	8.21	2.88	9.83	4.49	1.25	1.99	4.36	5.39	4.36	6.35	4.69	4.52	7.56	4.75	1.91	1
hsa-miR-1250-5p	nmiR03764.1:1	Endogenous	4.21	3.98	1.35	1.38	14.16	5.65	1.79	6.84	2.88	3.28	1.12	3.76	4.65	4.36	3.85	4.36	3.97	7.82	9.03	5.4	7.92	5.08	9.98
hsa-miR-1252-5p	nmiR03766.1:1	Endogenous	3.51	6.96	1.35	1.38	10.11	11.3	4.47	2.74	5.76	8.19	3.37	1.88	4.65	1.25	3.85	2.91	3.97	12.51	4.52	6.48	9.5	4.45	4.54
hsa-miR-1253	nmiR03767.1:1	Endogenous	1	1	1.35	1.38	2.02	2.83	1	1.37	1.44	1.64	1.12	1	1	1	1	1	1	1.56	2.26	1.08	1.58	1	1
hsa-miR-1254	nmiR03768.1:1	Endogenous	4.92	7.96	12.17	11.06	14.16	11.3	7.15	10.95	12.96	16.38	8.98	5.64	7.97	5.6	10.02	7.99	6.35	17.19	15.8	11.88	11.09	5.08	9.98
hsa-miR-1255a	nmiR03769.1:1	Endogenous	2.11	1	1.35	4.15	4.05	2.83	7.15	8.21	10.08	8.19	3.37	7.51	5.32	5.6	2.31	8.72	3.18	12.51	2.26	9.72	9.5	6.99	10.89
hsa-miR-1255b-5p	nmiR03770.1:1	Endogenous	3.51	6.96	2.7	6.91	10.11	5.65	8.05	2.74	4.32	8.19	6.73	5.64	1.33	2.49	2.31	3.63	3.18	4.69	9.03	3.24	4.75	5.08	8.17
hsa-miR-1257	nmiR03772.1:1	Endogenous	6.32	10.94	4.06	13.82	12.14	11.3	5.36	10.95	14.4	24.56	8.98	5.01	5.32	3.74	8.48	7.27	5.56	9.38	31.61	4.32	11.09	6.99	9.08
hsa-miR-1258	nmiR03773.1:1	Endogenous	6.32	11.94	10.82	13.82	18.2	33.9	4.47	12.32	15.84	22.93	12.35	6.89	6.64	8.1	4.62	6.54	9.53	18.76	13.55	12.96	30.09	9.53	11.8
hsa-miR-125a-3p	nmiR03121.1:1	Endogenous	11.24	22.88	14.88	23.5	18.2	25.43	18.77	17.79	14.4	18.01	15.71	9.39	15.95	14.95	15.41	13.81	22.23	12.51	11.29	14.04	19	10.8	14.52
hsa-miR-125a-5p	nmiR03189.1:1	Endogenous	113.06	94.5	123.07	132.71	121.35	81.93	84.03	80.76	115.19	188.32	92.04	53.23	62.45	126.42	104.04	159.15	95.27	67.22	63.22	112.31	55.43	84.47	39.03
hsa-miR-125b-5p	nmiR03127.1:1	Endogenous	14.75	34.82	28.4	33.18	36.41	39.55	31.29	19.16	17.28	49.13	24.69	11.27	23.92	12.45	22.35	15.26	37.32	35.95	15.8	22.68	38.01	21.59	20.88
hsa-miR-126-3p	nmiR03191.1:1	Endogenous	2646.73	2351.64	1141.46	2095.72	2807.27	1794.01	1225.61	1502.92	1807.07	2767.41	2207.95	1925.57	1394.55	3532.79	1412.66	1597.28	3009.87	3090.36	722.48	2518.23	1064.27	2481.35	788.81
hsa-miR-1260a	nmiR03775.1:1	Endogenous	45.65	52.72	52.75	24.88	30.34	42.38	35.76	17.79	28.8	31.11	65.1	70.76	21.92	36.74	46.24	22.53	27.79	32.83	51.93	48.59	50.68	44.46	71.71
hsa-miR-1260b	nmiR03774.1:1	Endogenous	11.24	11.94	12.17	13.82	14.16	14.13	7.15	8.21	7.2	16.38	11.22	6.89	12.62	10.59	10.79	6.54	17.47	26.57	15.8	12.96	4.75	6.35	15.43
hsa-miR-1261	nmiR03776.1:1	Endogenous	1	5.97	4.06	9.68	6.07	11.3	4.47	16.43	14.4	6.55	10.1	3.76	7.31	3.74	4.62	4.36	11.12	7.82	18.06	10.8	14.25	4.45	5.45
hsa-miR-1262	nmiR03777.1:1	Endogenous	2.11	1.99	8.11	5.53	14.16	16.95	11.62	8.21	2.88	16.38	8.98	10.02	3.32	6.23	6.94	6.54	5.56	10.94	13.55	3.24	11.09	5.08	7.26
hsa-miR-1264	nmiR03779.1:1	Endogenous	22.47	27.85	17.58	30.41	26.29	59.33	25.92	36.96	15.84	14.74	28.06	20.04	26.58	22.42	16.96	19.62	29.38	20.32	27.09	15.12	30.09	17.15	23.6
hsa-miR-1266-5p	nmiR03781.1:1	Endogenous	4.21	6.96	1.35	4.15	18.2	14.13	3.58	6.84	4.32	11.46	11.22	3.13	3.32	2.49	6.94	4.36	1.59	7.82	15.8	8.64	7.92	2.54	3.63
hsa-miR-1268a	nmiR03784.1:1	Endogenous	4.92	2.98	5.41	4.15	22.25	11.3	8.05	5.48	7.2	6.55	4.49	6.26	2.66	4.36	3.85	5.81	6.35	10.94	4.52	11.88	6.33	5.72	4.54
hsa-miR-1268b	nmiR03783.1:1	Endogenous	6.32	9.95	4.06	6.91	8.09	19.78	2.68	8.21	17.28	13.1	5.61	5.64	1.33	3.74	5.39	2.91	2.38	7.82	13.55	7.56	17.42	5.08	8.17
hsa-miR-1269a	nmiR03786.1:1	Endogenous	6.32	2.98	2.7	5.53	6.07	2.83	5.36	1.37	7.2	4.91	7.86	1.88	4.65	3.74	8.48	5.09	6.35	6.25	2.26	6.48	15.84	3.81	8.17
hsa-miR-1269b	nmiR03785.1:1	Endogenous	1	5.97	2.7	2.76	6.07	2.83	4.47	5.48	11.52	6.55	3.37	3.13	3.32	3.11	2.31	3.63	1.59	1.56	6.77	7.56	7.92	2.54	1.82
hsa-miR-127-3p	nmiR03192.1:1	Endogenous	20.36	24.87	18.93	33.18	28.32	42.38	16.09	20.53	12.96	31.11	12.35	8.77	11.96	13.08	14.64	18.89	27.79	57.84	36.12	19.44	30.09	10.8	8.17
hsa-miR-127-5p	nmiR03193.1:1	Endogenous	2.11	3.98	2.7	9.68	6.07	11.3	3.58	5.48	4.32	6.55	1.12	2.5	1	2.49	5.39	3.63	7.15	1.56	2.26	4.32	3.17	3.81	8.17
hsa-miR-1270	nmiR03787.1:1	Endogenous	7.72	5.97	8.11	8.29	20.23	8.48	6.26	6.84	7.2	18.01	10.1	6.89	6.64	8.1	13.1	10.9	4.76	18.76	22.58	14.04	20.59	7.62	8.17
hsa-miR-1271-3p	nmiR03976.1:1	Endogenous	4.21	1.99	1.35	2.76	6.07	2.83	3.58	2.74	1.44	3.28	1.12	1	2.66	1	1.54	4.36	1	1.56	2.26	1.08	3.17	3.18	3.63
hsa-miR-1271-5p	nmiR03788.1:1	Endogenous	1	5.97	1.35	4.15	6.07	5.65	3.58	5.48	5.76	1.64	1.12	1.88	4.65	2.49	4.62	7.99	4.76	10.94	9.03	2.16	6.33	5.72	5.45
hsa-miR-1272	nmiR03789.1:1	Endogenous	1	1	1.35	1.38	4.05	11.3	1	1.37	1.44	1.64	1.12	1	1	1	1	1	1	9.38	2.26	1.08	1.58	1	1
hsa-miR-1273c	nmiR03790.1:1	Endogenous	2.81	1.99	4.06	6.91	8.09	2.83	1	5.48	1.44	8.19	4.49	5.64	2.66	2.49	4.62	1.45	4.76	4.69	11.29	1.08	6.33	2.54	2.72
hsa-miR-1275	nmiR03796.1:1	Endogenous	2.11	4.97	4.06	1.38	4.05	5.65	5.36	1.37	1.44	1.64	8.98	3.76	1.33	6.23	1.54	4.36	1.59	9.38	4.52	3.24	12.67	1.91	5.45
hsa-miR-1276	nmiR03797.1:1	Endogenous	2.81	6.96	2.7	4.15	2.02	8.48	6.26	2.74	11.52	8.19	4.49	1	3.32	1.87	5.39	5.09	1.59	10.94	2.26	6.48	3.17	5.08	9.08
hsa-miR-1277-3p	nmiR03798.1:1	Endogenous	17.56	20.89	21.64	26.27	18.2	25.43	8.94	16.43	12.96	18.01	24.69	20.04	12.62	19.3	14.64	7.27	8.73	20.32	9.03	12.96	14.25	14.61	16.34
hsa-miR-1278	nmiR03799.1:1	Endogenous	1.4	1.99	1.35	1.38	2.02	5.65	1	6.84	5.76	4.91	5.61	1.88	2.66	3.11	2.31	4.36	5.56	9.38	4.52	3.24	9.5	3.81	5.45
hsa-miR-1279	nmiR03800.1:1	Endogenous	5.62	4.97	5.41	5.53	8.09	11.3	3.58	5.48	7.2	9.83	5.61	8.77	3.99	8.1	4.62	2.91	3.97	14.07	6.77	8.64	14.25	8.26	9.08
hsa-miR-128-1-5p	nmiR04002.1:1	Endogenous	2.11	3.98	8.11	6.91	14.16	2.83	2.68	2.74	1.44	3.28	4.49	2.5	3.32	1.87	6.94	4.36	3.97	4.69	6.77	3.24	4.75	3.81	8.17
hsa-miR-128-2-5p	nmiR04030.1:1	Endogenous	2.11	1.99	2.7	2.76	6.07	11.3	3.58	2.74	1.44	3.28	1.12	1.25	2.66	1	1	1	1.59	4.69	2.26	5.4	11.09	3.81	3.63
hsa-miR-128-3p	nmiR03194.1:1	Endogenous	1	1	1.35	1.38	2.02	2.83	1	1.37	1.44	1.64	1.12	1	1	1	1	1	1	1.56	2.26	1.08	1.58	1	1
hsa-miR-1281	nmiR03802.1:1	Endogenous	1	5.97	4.06	1.38	6.07	8.48	1.79	1.37	2.88	6.55	4.49	2.5	1	1.87	3.08	2.91	2.38	3.13	9.03	2.16	1.58	3.81	4.54
hsa-miR-1283	nmiR03570.1:1	Endogenous	1	1	1.35	1.38	2.02	2.83	1	1.37	1.44	1.64	1.12	1	1	1	1	1	1	1.56	2.26	1.08	1.58	1	1
hsa-miR-1285-3p	nmiR03805.1:1	Endogenous	9.13	16.91	1.35	12.44	22.25	19.78	29.5	45.17	25.92	21.29	14.59	8.77	14.62	5.6	12.33	32.7	7.15	14.07	22.58	11.88	22.17	7.62	25.42
hsa-miR-1285-5p	nmiR03980.1:1	Endogenous	4.21	10.94	4.06	11.06	10.11	2.83	16.09	19.16	17.28	13.1	5.61	8.77	3.99	3.74	9.25	2.91	14.29	20.32	13.55	12.96	14.25	10.16	8.17
hsa-miR-1286	nmiR03806.1:1	Endogenous	4.92	3.98	4.06	4.15	4.05	2.83	4.47	1.37	7.2	19.65	11.22	1.25	4.65	3.11	1	5.09	2.38	6.25	18.06	7.56	11.09	1.91	5.45
hsa-miR-1287-3p	nmiR04026.1:1	Endogenous	5.62	3.98	9.47	1.38	18.2	25.43	2.68	12.32	5.76	6.55	5.61	4.38	5.98	5.6	5.39	4.36	2.38	7.82	4.52	10.8	7.92	6.35	9.98
hsa-miR-1287-5p	nmiR03807.1:1	Endogenous	7.72	9.95	6.76	6.91	20.23	14.13	7.15	12.32	8.64	8.19	13.47	6.89	4.65	4.98	9.25	7.27	8.73	3.13	11.29	17.28	11.09	5.72	10.89
hsa-miR-1288-3p	nmiR03808.1:1	Endogenous	4.21	6.96	1.35	1.38	4.05	2.83	3.58	5.48	2.88	9.83	2.24	1	1.99	3.11	2.31	5.09	1.59	4.69	2.26	3.24	4.75	2.54	6.35
hsa-miR-1289	nmiR03809.1:1	Endogenous	2.11	6.96	10.82	4.15	8.09	11.3	5.36	16.43	17.28	11.46	11.22	3.13	4.65	4.98	7.71	5.09	4.76	4.69	9.03	9.72	7.92	9.53	10.89
hsa-miR-129-2-3p	nmiR03195.1:1	Endogenous	4.92	10.94	12.17	5.53	18.2	11.3	11.62	13.69	8.64	6.55	12.35	5.01	3.99	4.98	5.39	5.09	3.97	14.07	22.58	3.24	12.67	3.18	7.26
hsa-miR-129-5p	nmiR03196.1:1	Endogenous	1.4	2.98	2.7	2.76	8.09	2.83	1.79	6.84	4.32	1.64	3.37	3.13	6.64	2.49	3.08	1.45	3.18	6.25	6.77	3.24	3.17	1.27	5.45
hsa-miR-1290	nmiR03810.1:1	Endogenous	9.13	1.99	4.06	6.91	4.05	19.78	3.58	5.48	11.52	4.91	7.86	3.13	5.98	4.98	7.71	6.54	3.18	7.82	15.8	10.8	11.09	2.54	12.71
hsa-miR-1291	nmiR03811.1:1	Endogenous	1.4	1.99	2.7	6.91	4.05	5.65	1	4.11	4.32	6.55	2.24	2.5	2.66	1.87	1.54	4.36	1	4.69	9.03	1.08	7.92	1	6.35
hsa-miR-1293	nmiR03813.1:1	Endogenous	3.51	7.96	4.06	4.15	6.07	11.3	5.36	5.48	10.08	13.1	10.1	3.76	4.65	4.98	5.39	3.63	3.97	6.25	18.06	2.16	6.33	1.91	8.17
hsa-miR-1295a	nmiR03815.1:1	Endogenous	4.92	1.99	2.7	8.29	10.11	19.78	5.36	13.69	5.76	8.19	3.37	1.88	4.65	5.6	6.94	5.09	7.15	10.94	20.32	10.8	7.92	6.99	6.35
hsa-miR-1296-3p	nmiR04019.1:1	Endogenous	2.81	3.98	5.41	1.38	12.14	2.83	3.58	4.11	14.4	6.55	7.86	3.13	1.99	1.25	5.39	1	4.76	15.63	18.06	7.56	7.92	4.45	6.35
hsa-miR-1296-5p	nmiR03816.1:1	Endogenous	2.11	2.98	4.06	2.76	6.07	11.3	4.47	4.11	4.32	1.64	4.49	1.88	3.32	3.74	1.54	2.18	3.97	7.82	6.77	3.24	6.33	4.45	9.08
hsa-miR-1297	nmiR03817.1:1	Endogenous	3.51	4.97	9.47	2.76	20.23	5.65	3.58	6.84	5.76	6.55	10.1	5.64	3.32	7.47	4.62	7.27	6.35	10.94	18.06	8.64	7.92	6.99	15.43
hsa-miR-1298-5p	nmiR03818.1:1	Endogenous	4.92	5.97	4.06	5.53	12.14	22.6	6.26	13.69	12.96	16.38	10.1	5.64	4.65	3.11	7.71	5.81	8.73	14.07	11.29	9.72	14.25	9.53	8.17
hsa-miR-1299	nmiR03819.1:1	Endogenous	11.94	12.93	5.41	5.53	14.16	25.43	53.64	83.5	15.84	9.83	10.1	11.9	21.92	11.83	28.52	51.6	14.29	28.14	22.58	19.44	23.76	6.35	21.79
hsa-miR-1301-3p	nmiR03820.1:1	Endogenous	4.21	2.98	2.7	4.15	10.11	11.3	2.68	5.48	1.44	3.28	4.49	1	1.33	2.49	3.08	2.18	3.97	1.56	6.77	6.48	7.92	1.91	1.82
hsa-miR-1302	nmiR03821.1:1	Endogenous	2.11	4.97	4.06	1.38	8.09	14.13	5.36	10.95	14.4	6.55	3.37	2.5	1.99	1	3.85	2.18	5.56	10.94	18.06	3.24	4.75	3.81	4.54
hsa-miR-1303	nmiR03822.1:1	Endogenous	4.21	4.97	2.7	5.53	6.07	14.13	5.36	10.95	4.32	21.29	3.37	5.01	5.98	6.85	5.39	6.54	10.32	7.82	11.29	10.8	7.92	3.81	8.17
hsa-miR-1304-3p	nmiR03981.1:1	Endogenous	1	1.99	2.7	2.76	10.11	2.83	3.58	6.84	10.08	13.1	3.37	1.88	6.64	1	3.85	5.09	2.38	9.38	11.29	6.48	4.75	1.91	2.72
hsa-miR-1304-5p	nmiR03823.1:1	Endogenous	12.64	27.85	13.52	9.68	20.23	25.43	8.94	10.95	14.4	19.65	10.1	6.89	15.95	16.19	12.33	13.08	15.09	20.32	20.32	19.44	15.84	16.51	14.52
hsa-miR-1305	nmiR03824.1:1	Endogenous	3.51	2.98	1.35	2.76	2.02	5.65	4.47	8.21	1.44	3.28	3.37	1.88	4.65	1.87	4.62	2.18	7.15	7.82	9.03	5.4	7.92	3.18	5.45
hsa-miR-1306-3p	nmiR03825.1:1	Endogenous	2.11	2.98	5.41	5.53	10.11	11.3	2.68	4.11	4.32	9.83	4.49	1	3.32	3.74	3.85	2.91	5.56	6.25	9.03	3.24	12.67	6.35	7.26
hsa-miR-1306-5p	nmiR03982.1:1	Endogenous	4.92	5.97	6.76	6.91	10.11	14.13	5.36	6.84	2.88	11.46	4.49	3.76	3.32	9.34	4.62	5.81	7.15	4.69	4.52	8.64	7.92	5.72	8.17
hsa-miR-1307-3p	nmiR03826.1:1	Endogenous	7.02	3.98	10.82	6.91	14.16	16.95	14.3	12.32	12.96	8.19	12.35	10.02	9.97	8.72	8.48	12.35	12.7	14.07	20.32	16.2	19	7.62	6.35
hsa-miR-1307-5p	nmiR03983.1:1	Endogenous	7.72	10.94	1.35	1.38	22.25	31.08	5.36	5.48	11.52	18.01	5.61	2.5	1.99	5.6	7.71	8.72	5.56	12.51	13.55	8.64	25.34	7.62	6.35
hsa-miR-130a-3p	nmiR03197.1:1	Endogenous	351.82	344.19	320.53	591.67	754.4	299.47	240.47	242.27	282.22	615.71	244.7	217.29	154.14	354.34	275.13	342.27	601.02	467.38	203.2	383.35	191.63	466.8	110.74
hsa-miR-130b-3p	nmiR03198.1:1	Endogenous	4.21	5.97	10.82	9.68	10.11	2.83	7.15	12.32	2.88	8.19	10.1	5.64	7.31	8.1	4.62	5.81	10.32	10.94	11.29	6.48	9.5	10.16	8.17
hsa-miR-132-3p	nmiR03199.1:1	Endogenous	17.56	23.87	18.93	23.5	26.29	19.78	67.94	71.18	146.87	214.52	30.31	39.45	39.86	65.39	80.92	42.15	31.76	56.27	33.87	42.11	44.34	29.21	33.59
hsa-miR-1322	nmiR03828.1:1	Endogenous	6.32	4.97	5.41	5.53	22.25	11.3	5.36	13.69	11.52	11.46	5.61	1.88	4.65	7.47	6.17	5.81	5.56	10.94	22.58	8.64	17.42	5.72	6.35
hsa-miR-1323	nmiR03829.1:1	Endogenous	2.81	1.99	1.35	2.76	2.02	8.48	8.05	8.21	12.96	6.55	3.37	2.5	5.32	1	5.39	2.18	6.35	3.13	4.52	4.32	3.17	3.81	6.35
hsa-miR-133a-3p	nmiR03200.1:1	Endogenous	2.11	9.95	5.41	4.15	18.2	8.48	1	6.84	2.88	4.91	8.98	3.13	2.66	3.11	2.31	2.18	3.18	7.82	11.29	7.56	6.33	3.18	4.54
hsa-miR-133a-5p	nmiR04003.1:1	Endogenous	2.11	2.98	2.7	1.38	12.14	11.3	3.58	2.74	7.2	3.28	1.12	1.88	1.33	1.87	2.31	1.45	3.18	4.69	4.52	11.88	4.75	2.54	2.72
hsa-miR-133b	nmiR03201.1:1	Endogenous	2.11	5.97	4.06	4.15	2.02	8.48	3.58	4.11	8.64	1.64	2.24	3.76	1.99	1	4.62	3.63	1	3.13	4.52	5.4	6.33	1.91	1.82
hsa-miR-134-3p	nmiR04005.1:1	Endogenous	2.81	2.98	1.35	2.76	4.05	2.83	4.47	5.48	2.88	4.91	4.49	1.88	1.33	1	6.94	4.36	4.76	7.82	11.29	5.4	3.17	3.81	2.72
hsa-miR-134-5p+hsa-miR-6728-5p	nmiR03202.1:1	Endogenous	2.11	3.98	4.06	1.38	2.02	2.83	6.26	5.48	2.88	4.91	2.24	1.25	3.32	3.11	5.39	1.45	3.18	12.51	9.03	3.24	3.17	1.91	1.82
hsa-miR-135a-5p	nmiR03203.1:1	Endogenous	1.4	3.98	4.06	9.68	12.14	14.13	8.94	4.11	5.76	22.93	3.37	5.01	3.32	4.98	3.08	2.91	5.56	6.25	6.77	12.96	11.09	7.62	4.54
hsa-miR-135b-5p	nmiR03204.1:1	Endogenous	4.92	4.97	4.06	6.91	16.18	5.65	5.36	13.69	5.76	8.19	5.61	3.76	2.66	4.36	9.25	1.45	6.35	9.38	9.03	4.32	6.33	5.08	1.82
hsa-miR-136-5p	nmiR03205.1:1	Endogenous	36.52	30.84	28.4	38.71	36.41	50.85	11.62	15.06	18.72	73.69	14.59	10.02	19.27	28.02	13.87	29.07	62.72	71.91	9.03	16.2	30.09	12.7	8.17
hsa-miR-137	nmiR03206.1:1	Endogenous	2.81	1.99	1.35	2.76	8.09	5.65	5.36	2.74	1.44	4.91	4.49	3.76	2.66	1.87	1.54	2.18	5.56	1.56	2.26	1.08	3.17	1.91	1
hsa-miR-138-5p	nmiR03207.1:1	Endogenous	2.81	5.97	8.11	8.29	16.18	2.83	10.73	13.69	10.08	18.01	8.98	4.38	5.32	6.23	1.54	8.72	6.35	6.25	9.03	8.64	11.09	3.81	7.26
hsa-miR-139-3p	nmiR03208.1:1	Endogenous	6.32	5.97	6.76	4.15	18.2	11.3	4.47	15.06	12.96	16.38	10.1	2.5	3.32	4.98	6.17	9.45	7.94	3.13	20.32	2.16	9.5	3.81	5.45
hsa-miR-139-5p	nmiR03209.1:1	Endogenous	1	1	2.7	6.91	2.02	11.3	1	5.48	7.2	11.46	5.61	1.88	3.32	2.49	4.62	2.91	3.18	3.13	2.26	3.24	3.17	6.99	1
hsa-miR-140-3p	nmiR03210.1:1	Endogenous	16.15	17.91	17.58	19.35	26.29	16.95	9.83	13.69	11.52	21.29	21.33	19.41	13.29	14.95	15.41	16.71	14.29	12.51	20.32	14.04	14.25	15.88	23.6
hsa-miR-140-5p	nmiR03211.1:1	Endogenous	167.13	153.19	219.1	156.21	151.69	141.26	122.47	106.76	139.67	147.38	159.39	159.05	178.06	147.59	144.89	133.71	142.12	140.68	153.53	153.34	188.47	167.03	186.99
hsa-miR-141-3p	nmiR03212.1:1	Endogenous	8.43	17.91	14.88	2.76	14.16	19.78	14.3	8.21	10.08	6.55	7.86	8.14	5.32	8.1	13.87	9.45	15.09	10.94	24.84	15.12	9.5	5.72	9.98
hsa-miR-142-3p	nmiR03213.1:1	Endogenous	40737.5	52592.6	47006.8	49431.9	41366.9	39024.7	37992.1	25430.6	34718.9	30469.4	42174.4	44980.6	40653.8	38092.3	39758	30955.8	41117.1	31777.4	44164	36719.5	40016.4	46659.2	45065
hsa-miR-142-5p	nmiR03214.1:1	Endogenous	11.94	10.94	5.41	11.06	10.11	31.08	14.3	16.43	14.4	14.74	14.59	21.29	21.26	12.45	10.79	15.26	17.47	14.07	27.09	17.28	20.59	17.78	25.42
hsa-miR-143-3p	nmiR03128.1:1	Endogenous	1	1	1.35	1.38	2.02	2.83	1	1.37	1.44	1.64	1.12	1	1	1	1	1	1	1.56	2.26	1.08	1.58	1	1
hsa-miR-144-3p	nmiR03215.1:1	Endogenous	1765.43	953.99	1479.57	2047.34	1357.12	2627.45	14088.7	27084.1	9979.94	2921.34	2352.75	2000.08	3373.75	2023.28	8591.58	27768.5	2795.5	4148.62	1359.17	3013.89	5517.76	1707.16	7242.74
hsa-miR-145-5p	nmiR03216.1:1	Endogenous	25.28	60.68	10.82	17.97	26.29	16.95	16.99	13.69	15.84	21.29	16.84	20.04	53.15	39.23	15.41	23.98	30.96	31.26	24.84	33.48	19	13.34	44.48
hsa-miR-1469	nmiR03832.1:1	Endogenous	1	2.98	2.7	5.53	2.02	8.48	5.36	4.11	5.76	6.55	5.61	1	1.99	3.11	5.39	6.54	1.59	3.13	9.03	3.24	4.75	3.18	5.45
hsa-miR-146a-5p	nmiR03217.1:1	Endogenous	363.76	410.84	243.44	243.3	218.43	271.22	157.34	140.98	191.51	199.78	434.41	521	232.54	382.98	157.99	142.43	426.35	295.44	259.64	184.66	245.48	192.44	341.3
hsa-miR-146b-3p	nmiR03218.1:1	Endogenous	3.51	3.98	4.06	6.91	8.09	2.83	7.15	9.58	11.52	1.64	2.24	3.76	1.33	2.49	5.39	5.81	3.18	1.56	15.8	6.48	7.92	3.18	5.45
hsa-miR-146b-5p	nmiR03219.1:1	Endogenous	115.87	207.91	175.82	215.65	190.12	124.31	137.67	110.87	113.75	80.24	217.76	165.94	158.79	173.74	119.46	119.18	158	146.94	173.85	117.7	177.38	167.03	254.16

hsa-miR-147a	nmiR03222.1:1	Endogenous	2.81	5.97	1.35	2.76	10.11	8.48	7.15	2.74	8.64	3.28	11.22	1.88	1.99	3.74	1	4.36	3.97	12.51	11.29	7.56	19	3.18	7.26
hsa-miR-147b	nmiR03221.1:1	Endogenous	1	2.98	5.41	1.38	6.07	5.65	1	2.74	7.2	3.28	3.37	1	1.99	1	4.62	1.45	1	3.13	11.29	4.32	12.67	1.91	3.63
hsa-miR-148a-3p	nmiR03223.1:1	Endogenous	292.13	168.12	206.92	204.6	218.43	141.26	211.87	142.35	188.63	265.28	245.83	306.84	395.31	213.6	227.35	311.03	254.06	243.85	232.55	313.16	218.56	198.15	318.61
hsa-miR-148b-3p	nmiR03130.1:1	Endogenous	382.72	421.78	424.67	558.49	786.77	392.7	379.93	416.11	440.61	558.4	287.36	292.44	279.04	368.66	370.7	433.11	558.94	609.63	295.77	489.18	334.17	389.95	253.26
hsa-miR-149-5p	nmiR03224.1:1	Endogenous	7.72	10.94	2.7	5.53	20.23	11.3	11.62	12.32	12.96	16.38	17.96	8.14	5.32	12.45	12.33	6.54	11.91	6.25	15.8	10.8	22.17	5.72	3.63
hsa-miR-150-5p	nmiR03225.1:1	Endogenous	45144.8	75002.9	56567.3	67061.7	57844.4	53885.3	49235.3	28209.2	47032.9	33259.7	62663.3	54289.1	46476.5	39979.8	35247.2	21346.7	43179.8	30248.7	71970.5	32430.3	57380.6	47132.4	33948
hsa-miR-151a-3p	nmiR03227.1:1	Endogenous	72.33	59.69	50.04	77.41	117.31	59.33	33.97	61.6	56.16	60.59	43.78	47.59	25.91	60.41	57.03	66.13	98.45	89.1	24.84	74.51	42.76	74.94	31.77
hsa-miR-151a-5p	nmiR03228.1:1	Endogenous	49.16	40.79	16.23	31.8	32.36	25.43	25.03	28.74	15.84	37.66	30.31	33.81	19.27	39.86	19.27	25.43	35.73	39.08	13.55	32.4	15.84	26.67	23.6
hsa-miR-151b	nmiR03226.1:1	Endogenous	3.51	6.96	2.7	5.53	2.02	8.48	6.26	15.06	2.88	1.64	4.49	4.38	7.97	3.74	9.25	7.27	2.38	7.82	11.29	4.32	7.92	3.18	7.26
hsa-miR-152-3p	nmiR03229.1:1	Endogenous	3.51	7.96	5.41	8.29	8.09	19.78	4.47	6.84	4.32	8.19	3.37	4.38	3.32	3.11	3.85	5.09	4.76	4.69	6.77	3.24	4.75	5.08	9.08
hsa-miR-152-5p	nmiR04004.1:1	Endogenous	2.81	1.99	1.35	1.38	2.02	8.48	7.15	12.32	2.88	4.91	3.37	1	1.99	1.87	3.85	2.18	4.76	1.56	6.77	2.16	4.75	3.81	2.72
hsa-miR-153-3p	nmiR03230.1:1	Endogenous	2.81	1.99	2.7	2.76	6.07	2.83	4.47	5.48	4.32	8.19	6.73	1.88	2.66	1.87	3.85	5.81	1	1.56	4.52	7.56	4.75	3.18	5.45
hsa-miR-1537-3p	nmiR03835.1:1	Endogenous	145.36	97.49	235.32	163.12	141.58	189.29	190.41	182.05	145.43	209.6	129.09	155.3	197.99	142.61	211.94	138.07	128.62	178.2	214.49	234.33	224.89	151.15	117.1
hsa-miR-154-5p	nmiR03231.1:1	Endogenous	11.24	9.95	22.99	15.21	22.25	8.48	14.3	19.16	7.2	11.46	4.49	9.39	14.62	9.34	12.33	14.53	12.7	28.14	9.03	12.96	14.25	10.16	9.08
hsa-miR-155-5p	nmiR03135.1:1	Endogenous	177.67	181.05	171.76	142.39	97.08	127.13	85.82	46.54	125.27	116.26	180.72	205.39	125.57	140.12	107.12	63.95	119.09	73.47	173.85	78.83	88.69	146.07	152.5
hsa-miR-15a-5p	nmiR03129.1:1	Endogenous	1962.75	1676.19	2661.61	2206.31	2327.93	2107.61	2341.26	2622.58	1657.32	1745.6	1387.4	1561.74	1573.93	1670.81	2133.25	2402.46	1703.03	1796.07	2158.41	2388.65	2100.04	2142.84	1479.59
hsa-miR-15b-5p	nmiR03131.1:1	Endogenous	3728.18	3061.91	3686.76	3381.36	3418.08	2816.74	3355.9	4044.75	3248.41	2929.53	3272.07	3436.59	3672.06	3441.25	3573.66	4447.38	3444.95	3526.48	2698.02	3346.48	2709.78	3428.29	3102.61
hsa-miR-16-5p	nmiR03132.1:1	Endogenous	1742.25	1448.39	1503.92	1198.54	1094.19	1361.75	1644.87	1750.67	1498.94	1226.5	1981.2	2655.09	2603.73	1705.68	1733.27	2349.41	1522.8	1857.03	1609.78	1821.72	1561.57	1444.86	3880.53
hsa-miR-181a-2-3p	nmiR03920.1:1	Endogenous	3.51	6.96	8.11	4.15	8.09	2.83	2.68	6.84	8.64	4.91	4.49	5.64	2.66	6.85	9.25	5.09	5.56	9.38	11.29	7.56	7.92	8.26	7.26
hsa-miR-181a-3p	nmiR03914.1:1	Endogenous	19.66	24.87	27.05	23.5	24.27	16.95	42.02	17.79	18.72	24.56	41.53	54.48	25.91	31.76	35.45	15.99	31.76	42.21	42.9	42.11	44.34	38.74	45.39
hsa-miR-181a-5p	nmiR03232.1:1	Endogenous	1504.9	1874.15	1940.75	2681.86	2024.55	1717.73	1901.44	1029.32	1471.58	1829.11	1940.79	1784.04	1494.87	1683.88	1642.33	1103.12	1617.28	1288.04	1941.67	1416.77	1664.51	2026.62	1843.59
hsa-miR-181b-2-3p	nmiR04033.1:1	Endogenous	2.81	6.96	2.7	1.38	16.18	22.6	9.83	8.21	10.08	9.83	5.61	7.51	5.98	2.49	5.39	2.18	6.35	6.25	11.29	8.64	9.5	5.08	10.89
hsa-miR-181b-5p+hsa-miR-181d-5p	nmiR03233.1:1	Endogenous	61.8	44.76	56.8	52.53	34.38	70.63	36.65	30.11	40.32	83.51	62.86	70.76	35.21	51.69	38.53	13.81	43.67	39.08	58.7	49.67	44.34	47.63	58.09
hsa-miR-181c-5p	nmiR03234.1:1	Endogenous	39.33	47.75	32.46	51.15	50.56	53.68	48.27	21.9	38.88	44.21	47.14	33.81	32.55	34.25	34.68	23.25	38.11	29.7	31.61	25.92	26.92	40.01	35.4
hsa-miR-181d-3p	nmiR04012.1:1	Endogenous	2.11	4.97	1.35	4.15	6.07	2.83	3.58	2.74	1.44	3.28	2.24	3.13	1	1	4.62	1.45	3.18	1.56	11.29	7.56	1.58	5.08	3.63
hsa-miR-182-3p	nmiR03913.1:1	Endogenous	2.81	1.99	1.35	2.76	4.05	11.3	3.58	4.11	5.76	4.91	3.37	1	1.99	1.25	1	2.91	3.18	7.82	13.55	2.16	3.17	3.18	5.45
hsa-miR-182-5p	nmiR03235.1:1	Endogenous	4.92	2.98	4.06	1.38	6.07	11.3	3.58	5.48	8.64	1.64	5.61	3.76	2.66	1.87	3.08	4.36	6.35	4.69	2.26	4.32	6.33	2.54	4.54
hsa-miR-1827	nmiR03839.1:1	Endogenous	5.62	7.96	12.17	2.76	10.11	14.13	8.05	6.84	8.64	13.1	13.47	2.5	4.65	2.49	4.62	5.09	3.97	10.94	11.29	6.48	4.75	6.99	11.8
hsa-miR-183-5p	nmiR03236.1:1	Endogenous	4.92	9.95	2.7	5.53	6.07	5.65	20.56	34.22	14.4	8.19	14.59	3.76	9.97	12.45	9.25	10.9	1.59	14.07	6.77	12.96	14.25	6.35	19.06
hsa-miR-184	nmiR03237.1:1	Endogenous	2.11	5.97	4.06	2.76	16.18	5.65	5.36	8.21	5.76	6.55	1.12	3.76	1.99	3.74	6.94	2.18	7.15	9.38	13.55	7.56	3.17	6.35	9.98
hsa-miR-185-5p	nmiR03238.1:1	Endogenous	76.54	47.75	75.74	47	95.06	48.03	58.11	94.45	82.07	73.69	62.86	47.59	51.16	78.46	61.65	121.36	95.27	73.47	38.38	78.83	60.18	66.05	54.46
hsa-miR-186-5p	nmiR03239.1:1	Endogenous	96.21	100.47	60.86	81.56	56.63	73.46	57.21	42.43	64.8	54.04	95.41	114.59	138.86	121.43	73.21	60.32	71.46	60.96	121.92	59.39	85.52	83.2	140.7
hsa-miR-187-3p	nmiR03240.1:1	Endogenous	1	2.98	1.35	8.29	6.07	2.83	6.26	6.84	5.76	8.19	4.49	2.5	1.33	3.11	4.62	6.54	1.59	4.69	11.29	3.24	4.75	1.91	6.35
hsa-miR-188-3p	nmiR03241.1:1	Endogenous	2.11	1	1.35	1.38	6.07	14.13	1.79	2.74	1.44	3.28	2.24	1.25	1.33	1.25	3.08	1.45	1.59	3.13	4.52	8.64	9.5	3.18	6.35
hsa-miR-188-5p	nmiR03242.1:1	Endogenous	7.72	9.95	10.82	16.59	12.14	25.43	16.99	9.58	15.84	14.74	10.1	6.89	4.65	8.1	4.62	7.99	8.73	25.01	24.84	11.88	20.59	5.08	11.8
hsa-miR-18a-5p	nmiR03133.1:1	Endogenous	42.84	48.74	60.86	49.77	78.88	64.98	62.58	80.76	53.28	32.75	42.65	31.31	31.23	44.84	47.78	69.04	60.34	53.15	29.35	63.71	57.01	60.97	47.2
hsa-miR-18b-5p	nmiR03134.1:1	Endogenous	4.92	7.96	4.06	6.91	6.07	5.65	6.26	9.58	5.76	4.91	7.86	4.38	3.99	1.87	4.62	8.72	3.97	6.25	6.77	6.48	6.33	4.45	9.08
hsa-miR-1908-3p	nmiR04027.1:1	Endogenous	2.81	1	8.11	6.91	8.09	5.65	1.79	1.37	8.64	6.55	7.86	3.13	3.32	2.49	1	4.36	2.38	3.13	6.77	3.24	3.17	3.81	3.63
hsa-miR-1908-5p	nmiR03840.1:1	Endogenous	7.02	3.98	8.11	9.68	8.09	16.95	8.05	6.84	2.88	16.38	5.61	6.89	3.99	4.36	4.62	6.54	9.53	9.38	9.03	5.4	6.33	4.45	9.08
hsa-miR-1909-3p	nmiR03841.1:1	Endogenous	3.51	7.96	1.35	5.53	4.05	5.65	6.26	10.95	8.64	3.28	4.49	2.5	3.99	2.49	7.71	4.36	2.38	4.69	15.8	5.4	9.5	1.27	1
hsa-miR-190a-3p	nmiR04006.1:1	Endogenous	3.51	4.97	5.41	5.53	14.16	5.65	6.26	4.11	7.2	14.74	6.73	3.13	5.32	6.23	3.08	5.09	7.15	12.51	9.03	6.48	14.25	1.27	8.17
hsa-miR-190a-5p	nmiR03244.1:1	Endogenous	23.17	21.88	33.81	22.12	34.38	42.38	112.64	212.16	148.31	57.31	47.14	27.55	57.14	41.1	50.09	135.89	17.47	32.83	33.87	20.52	77.6	19.05	65.36
hsa-miR-190b	nmiR03243.1:1	Endogenous	9.83	10.94	5.41	2.76	8.09	31.08	6.26	8.21	5.76	13.1	14.59	5.01	6.64	6.23	5.39	2.18	7.94	6.25	6.77	7.56	11.09	7.62	13.62
hsa-miR-191-5p	nmiR03245.1:1	Endogenous	1757.7	1802.53	2424.93	2791.07	3389.76	2376	2464.63	2258.49	1714.92	1907.71	1314.44	1352.59	1353.35	1554.98	2040	1951.18	2535.88	2505.74	1943.93	2524.71	1833.97	2410.86	1127.4
hsa-miR-1910-3p	nmiR04028.1:1	Endogenous	1.4	1	1.35	2.76	2.02	2.83	1.79	5.48	2.88	3.28	1.12	1.88	1	1.25	2.31	7.27	1	1.56	9.03	2.16	4.75	1	6.35
hsa-miR-1910-5p	nmiR03842.1:1	Endogenous	22.47	23.87	18.93	16.59	12.14	67.81	101.02	205.32	51.84	34.39	15.71	15.03	41.86	14.32	46.24	123.54	19.05	48.46	33.87	35.64	61.77	20.32	65.36
hsa-miR-1915-3p	nmiR03847.1:1	Endogenous	6.32	3.98	6.76	1.38	8.09	11.3	1.79	8.21	8.64	26.2	8.98	10.02	3.99	4.98	3.08	5.81	3.18	28.14	13.55	8.64	9.5	6.99	15.43
hsa-miR-192-5p	nmiR03137.1:1	Endogenous	7.02	6.96	9.47	5.53	24.27	16.95	10.73	16.43	5.76	9.83	11.22	6.89	11.96	6.85	9.25	11.63	6.35	10.94	18.06	4.32	15.84	10.16	13.62
hsa-miR-193a-3p	nmiR03246.1:1	Endogenous	5.62	7.96	8.11	2.76	10.11	11.3	6.26	8.21	4.32	6.55	7.86	1.25	2.66	6.23	8.48	6.54	3.97	1.56	6.77	6.48	11.09	6.35	11.8
hsa-miR-193a-5p+hsa-miR-193b-5p	nmiR03247.1:1	Endogenous	16.15	19.9	17.58	12.44	14.16	19.78	25.03	34.22	21.6	8.19	24.69	20.04	21.92	18.68	19.27	19.62	26.2	14.07	29.35	19.44	30.09	13.97	25.42
hsa-miR-193b-3p	nmiR03248.1:1	Endogenous	4.92	12.93	6.76	8.29	8.09	19.78	25.92	43.8	18.72	14.74	10.1	4.38	8.64	8.72	14.64	42.88	9.53	7.82	15.8	8.64	28.51	5.72	16.34
hsa-miR-194-5p	nmiR03249.1:1	Endogenous	66.01	108.43	63.56	76.03	42.47	98.88	55.43	83.5	90.71	49.13	51.63	48.22	67.1	48.57	64.74	66.13	65.9	67.22	124.18	59.39	126.7	76.21	60.82
hsa-miR-195-5p	nmiR03250.1:1	Endogenous	1.4	4.97	1.35	5.53	4.05	14.13	4.47	8.21	5.76	4.91	2.24	5.64	1.99	3.74	6.94	5.81	2.38	12.51	2.26	6.48	14.25	6.99	2.72
hsa-miR-196a-3p	nmiR03921.1:1	Endogenous	4.21	9.95	4.06	8.29	10.11	8.48	7.15	13.69	5.76	9.83	8.98	3.13	3.32	1.25	8.48	7.27	4.76	7.82	9.03	4.32	12.67	4.45	8.17
hsa-miR-196a-5p	nmiR03251.1:1	Endogenous	18.96	32.83	20.29	11.06	28.32	42.38	34.86	56.12	31.68	34.39	21.33	25.05	16.61	16.81	32.37	36.33	19.85	26.57	49.67	22.68	38.01	29.21	34.49
hsa-miR-196b-5p	nmiR03252.1:1	Endogenous	25.98	19.9	16.23	11.06	20.23	31.08	30.39	19.16	23.04	27.84	21.33	25.67	46.51	21.8	28.52	26.89	23.82	25.01	15.8	30.24	38.01	26.04	17.25
hsa-miR-197-3p	nmiR03253.1:1	Endogenous	33.01	30.84	32.46	30.41	20.23	33.9	16.99	12.32	30.24	29.48	31.43	41.33	41.86	41.1	21.58	20.35	19.85	37.52	27.09	29.16	31.67	30.49	29.05
hsa-miR-197-5p	nmiR03963.1:1	Endogenous	2.81	4.97	5.41	5.53	6.07	11.3	5.36	10.95	2.88	8.19	6.73	3.76	4.65	1	4.62	3.63	8.73	10.94	4.52	5.4	12.67	5.72	1.82
hsa-miR-1972	nmiR03848.1:1	Endogenous	5.62	12.93	9.47	8.29	14.16	8.48	8.94	9.58	10.08	18.01	5.61	5.01	5.32	5.6	8.48	5.81	3.97	7.82	11.29	5.4	6.33	4.45	7.26
hsa-miR-1973	nmiR03849.1:1	Endogenous	2.11	2.98	2.7	6.91	10.11	19.78	8.05	10.95	5.76	6.55	7.86	12.52	9.97	3.74	3.85	7.27	2.38	4.69	6.77	2.16	12.67	6.35	9.08
hsa-miR-1976	nmiR03850.1:1	Endogenous	2.81	1.99	4.06	4.15	8.09	5.65	5.36	4.11	2.88	1.64	4.49	1	1.99	3.74	2.31	5.81	1	4.69	4.52	2.16	4.75	3.18	6.35
hsa-miR-198	nmiR03254.1:1	Endogenous	4.21	7.96	8.11	4.15	10.11	2.83	6.26	2.74	10.08	6.55	3.37	2.5	5.32	1.87	6.17	2.18	7.15	9.38	6.77	4.32	11.09	6.35	9.08
hsa-miR-199a-3p+hsa-miR-199b-3p	nmiR03255.1:1	Endogenous	2067.39	2482.95	1959.69	3136.67	4601.26	2121.73	1334.67	2500.76	1781.16	206.33	1152.8	1092.72	807.23	1880.67	1143.69	1886.5	2874.1	2990.32	912.13	2088.45	996.17	1987.88	361.27
hsa-miR-199a-5p	nmiR03256.1:1	Endogenous	241.57	213.88	210.98	259.89	250.79	135.61	83.14	128.67	145.43	19.65	121.23	123.36	107.63	206.75	102.5	163.51	241.36	209.46	90.31	185.74	74.44	207.04	49.92
hsa-miR-199b-5p	nmiR03257.1:1	Endogenous	50.56	57.7	167.7	64.97	74.83	73.46	98.33	54.75	80.63	76.96	74.08	53.23	108.29	67.26	111.75	74.85	45.26	95.35	76.76	107.99	112.45	80.02	76.25
hsa-miR-19a-3p	nmiR03136.1:1	Endogenous	108.85	109.42	163.65	131.33	155.73	104.53	126.05	164.25	106.55	88.43	98.78	118.35	107.63	123.92	112.52	159.15	139.74	115.67	112.89	126.34	104.53	126.39	140.7
hsa-miR-19b-3p	nmiR03138.1:1	Endogenous	257.02	255.66	352.99	290.3	311.47	231.67	272.66	264.17	214.55	176.85	244.7	247.98	226.56	247.85	284.38	349.54	296.14	251.67	257.38	271.04	243.9	285.16	288.66
hsa-miR-200a-3p	nmiR03258.1:1	Endogenous	4.92	6.96	5.41	8.29	4.05	11.3	8.05	6.84	11.52	11.46	10.1	4.38	4.65	4.36	3.08	4.36	1.59	6.25	15.8	5.4	11.09	6.99	8.17
hsa-miR-200b-3p	nmiR03259.1:1	Endogenous	5.62	9.95	6.76	16.59	16.18	14.13	8.94	5.48	2.88	6.55	12.35	7.51	3.99	5.6	8.48	3.63	4.76	17.19	13.55	12.96	7.92	5.72	6.35
hsa-miR-200c-3p	nmiR03260.1:1	Endogenous	22.47	22.88	17.58	19.35	30.34	25.43	18.77	12.32	12.96	8.19	19.08	13.78	10.63	11.83	19.27	10.9	17.47	10.94	29.35	21.6	22.17	19.69	16.34
hsa-miR-202-3p	nmiR03261.1:1	Endogenous	2.81	3.98	1.35	1.38	4.05	14.13	4.47	8.21	4.32	1.64	3.37	1.88	3.32	1	1	6.54	5.56	7.82	13.55	1.08	14.25	3.18	6.35
hsa-miR-203a-3p	nmiR03262.1:1	Endogenous	1.4	2.98	1.35	4.15	6.07	8.48	3.58	8.21	2.88	9.83	2.24	2.5	4.65	3.74	2.31	1	3.97	7.82	13.55	2.16	7.92	3.18	2.72
hsa-miR-203a-5p	nmiR04031.1:1	Endogenous	2.81	1	1.35	5.53	2.02	2.83	3.58	9.58	5.76	4.91	3.37	3.76	3.32	1.87	3.08	4.36	4.76	6.25	11.29	5.4	11.09	1.91	3.63
hsa-miR-204-5p	nmiR03263.1:1	Endogenous	3.51	1.99	4.06	1.38	6.07	2.83	5.36	6.84	4.32	6.55	2.24	2.5	8.64	2.49	1	7.99	3.18	7.82	6.77	7.56	4.75	5.08	1.82
hsa-miR-205-5p	nmiR03264.1:1	Endogenous	3.51	5.97	1.35	2.76	14.16	2.83	2.68	8.21	7.2	3.28	4.49	4.38	3.32	3.11	1	3.63	5.56	12.51	2.26	5.4	7.92	6.99	8.17
hsa-miR-2053	nmiR03853.1:1	Endogenous	12.64	16.91	14.88	6.91	16.18	14.13	8.94	35.59	8.64	16.38	8.98	10.02	8.64	9.96	20.81	10.9	15.09	25.01	33.87	18.36	28.51	8.89	9.98
hsa-miR-206	nmiR03265.1:1	Endogenous	1.4	2.98	2.7	2.76	2.02	5.65	3.58	12.32	2.88	13.1	4.49	1.25	2.66	6.23	8.48	2.18	1	3.13	9.03	5.4	4.75	3.81	7.26
hsa-miR-208a-3p	nmiR03266.1:1	Endogenous	4.21	5.97	5.41	6.91	10.11	19.78	8.05	2.74	4.32	1.64	7.86	1.25	4.65	2.49	6.94	4.36	4.76	3.13	9.03	8.64	9.5	4.45	3.63
hsa-miR-208b-3p	nmiR03267.1:1	Endogenous	1.4	5.97	1.35	5.53	8.09	8.48	8.05	10.95	7.2	13.1	12.35	1.25	2.66	2.49	5.39	6.54	4.76	7.82	9.03	5.4	12.67	6.99	5.45
hsa-miR-208b-5p	nmiR04022.1:1	Endogenous	3.51	4.97	5.41	5.53	2.02	14.13	8.94	15.06	8.64	6.55	5.61	5.01	2.66	3.74	6.17	16.71	5.56	6.25	13.55	4.32	12.67	6.35	11.8
hsa-miR-20a-5p+hsa-miR-20b-5p	nmiR03139.1:1	Endogenous	464.88	423.77	428.72	388.45	406.53	395.53	354.01	461.28	372.93	356.98	453.49	509.73	450.45	454.6	395.36	534.12	490.66	434.56	390.59	437.34	318.33	429.33	567.33
hsa-miR-21-5p	nmiR03112.1:1	Endogenous	2759.09	1991.53	2327.55	1951.95	1541.17	1833.56	1201.47	1092.29	1604.05	1809.46	2487.45	3268.77	2207.75	2334.64	2074.68	1323.31	1518.03	1505.32	2083.91	2209.39	1514.06	1797.98	2244.81
hsa-miR-210-3p	nmiR03268.1:1	Endogenous	4.92	8.95	6.76	4.15	16.18	19.78	9.83	1.37	10.08	8.19	12.35	6.26	7.31	11.83	8.48	2.91	11.91	4.69	11.29	8.64	19	11.43	6.35
hsa-miR-210-5p	nmiR04001.1:1	Endogenous	1	6.96	6.76	5.53	14.16	5.65	2.68	8.21	4.32	9.83	6.73	1	1.99	3.11	2.31	5.09	3.18	4.69	6.77	3.24	6.33	3.18	3.63
hsa-miR-211-3p	nmiR03964.1:1	Endogenous	1	1	4.06	4.15	4.05	2.83	2.68	2.74	5.76	3.28	1.12	1.88	1.33	3.74	1.54	2.18	3.18	3.13	2.26	5.4	7.92	3.81	2.72
hsa-miR-211-5p	nmiR03270.1:1	Endogenous	2.11	2.98	2.7	5.53	6.07	5.65	3.58	6.84	4.32	6.55	5.61	5.01	1	1	3.08	2.91	1	7.82	6.77	5.4	4.75	1.91	4.54
hsa-miR-2110	nmiR03855.1:1	Endogenous	3.51	5.97	8.11	1.38	6.07	8.48	3.58	6.84	8.64	13.1	8.98	1.25	7.31	4.98	6.17	5.09	8.73	4.69	9.03	9.72	9.5	5.08	8.17
hsa-miR-2113	nmiR03856.1:1	Endogenous	1.4	1.99	2.7	5.53	8.09	5.65	2.68	6.84	5.76	6.55	2.24	4.38	2.66	1.87	3.85	1.45	4.76	1.56	4.52	5.4	4.75	3.18	3.63
hsa-miR-2116-5p	nmiR03753.1:1	Endogenous	4.21	8.95	6.76	2.76	4.05	8.48	4.47	2.74	5.76	8.19	4.49	5.64	6.64	1.87	6.17	4.36	6.35	12.51	9.03	3.24	3.17	7.62	7.26
hsa-miR-2117	nmiR03859.1:1	Endogenous	1.4	5.97	4.06	2.76	10.11	14.13	3.58	10.95	5.76	4.91	1.12	3.13	2.66	1.25	3.85	5.81	3.97	1.56	11.29	2.16	11.09	4.45	3.63
hsa-miR-212-3p	nmiR03271.1:1	Endogenous	5.62	20.89	13.52	13.82	10.11	31.08	9.83	27.38	12.96	19.65	16.84	6.26	8.64	6.23	13.1	13.08	19.85	23.45	33.87	19.44	33.26	7.62	11.8
hsa-miR-214-3p	nmiR03272.1:1	Endogenous	3.51	8.95	9.47	8.29	14.16	8.48	3.58	10.95	4.32	8.19	7.86	3.13	7.97	6.85	4.62	5.81	1	7.82	11.29	2.16	6.33	3.81	9.98
hsa-miR-215-5p	nmiR03273.1:1	Endogenous	4.21	6.96	6.76	4.15	14.16	11.3	7.15	6.84	12.96	9.83	6.73	6.26	6.64	4.36	6.17	5.81	8.73	6.25	13.55	9.72	14.25	5.72	4.54
hsa-miR-216a-5p	nmiR03274.1:1	Endogenous	4.21	7.96	9.47	8.29	8.09	14.13	6.26	9.58	7.2	11.46	8.98	1	3.99	2.49	6.17	7.27	4.76	4.69	4.52	11.88	14.25	1.91	4.54
hsa-miR-216b-5p	nmiR03275.1:1	Endogenous	8.43	8.95	1.35	8.29	14.16	25.43	6.26	8.21	4.32	13.1	5.61	3.76	7.31	4.36	9.25	11.63	11.12	12.51	18.06	8.64	6.33	8.89	9.98
hsa-miR-217	nmiR03276.1:1	Endogenous	21.07	17.91	12.17	5.53	24.27	8.48	13.41	16.43	17.28	6.55	24.69	11.27	17.27	10.59	22.35	10.9	15.09	10.94	24.84	10.8	17.42	14.61	25.42
hsa-miR-218-5p	nmiR03277.1:1	Endogenous	7.72	1.99	9.47	15.21	2.02	14.13	5.36	8.21	10.08	1.64	4.49	1.88	3.32	2.49	6.94	10.9	9.53	12.51	18.06	9.72	12.67	6.99	5.45
hsa-miR-219a-1-3p	nmiR03278.1:1	Endogenous	2.81	5.97	6.76	5.53	4.05	14.13	4.47	6.84	7.2	13.1	3.37	1	4.65	1.87	1.54	4.36	2.38	14.07	11.29	3.24	4.75	1.91	5.45
hsa-miR-219a-2-3p	nmiR03279.1:1	Endogenous	1.4	5.97	4.06	9.68	2.02	2.83	3.58	6.84	1.44	6.55	3.37	1.88	1.33	1	1.54	2.91	2.38	4.69	2.26	3.24	7.92	3.18	6.35
hsa-miR-219a-5p	nmiR03280.1:1	Endogenous	4.21	5.97	5.41	4.15	4.05	2.83	5.36	8.21	4.32	1.64	5.61	3.76	2.66	3.11	6.94	5.81	3.97	10.94	4.52	1.08	3.17	5.72	5.45
hsa-miR-219b-3p	nmiR03945.1:1	Endogenous	6.32	4.97	6.76	5.53	12.14	19.78	8.94	9.58	14.4	8.19	8.98	5.01	5.98	2.49	4.62	10.17	8.73	10.94	11.29	11.88	19	7.62	9.08
hsa-miR-22-3p	nmiR03140.1:1	Endogenous	120.78	88.53	68.97	80.18	72.81	107.36	59	88.97	97.91	153.93	159.39	168.45	120.92	170.01	113.29	106.1	120.68	153.19	110.63	110.15	95.02	130.2	149.77
hsa-miR-221-3p	nmiR03281.1:1	Endogenous	175.56	139.27	108.2	88.47	165.85	93.23	89.4	114.98	148.31	191.59	117.86	107.71	110.29	189.94	107.12	173.68	154.03	204.77	51.93	180.34	63.35	163.22	87.14
hsa-miR-221-5p	nmiR03922.1:1	Endogenous	9.83	11.94	8.11	9.68	2.02	2.83	8.94	4.11	8.64	4.91	7.86	15.66	11.96	8.72	6.94	7.27	11.12	18.76	11.29	9.72	7.92	7.62	13.62
hsa-miR-222-3p	nmiR03282.1:1	Endogenous	656.59	601.84	504.46	598.58	564.29	613.07	445.19	307.98	482.37	319.32	597.17	675.04	465.73	455.84	569.53	379.34	394.59	307.94	706.68	444.9	560.64	526.5	489.26
hsa-miR-223-3p	nmiR03283.1:1	Endogenous	36834.5	20616.7	37029.9	29992.6	31098.5	25116.1	39069.3	36432.8	32164.5	45212	30084	38817.5	70098.7	31934.6	37131.5	39777.9	29557.2	37869.1	30183.9	46982.5	39108.9	34037.8	32826.1
hsa-miR-224-5p	nmiR03284.1:1	Endogenous	4.21	5.97	4.06	6.91	14.16	16.95	2.68	5.48	1.44	6.55	3.37	1.88	4.65	3.74	3.08	4.36	10.32	6.25	9.03	4.32	4.75	2.54	4.54
hsa-miR-2278	nmiR03863.1:1	Endogenous	1.4	2.98	2.7	5.53	8.09	11.3	2.68	2.74	4.32	1.64	4.49	1.25	5.32	1.25	4.62	3.63	2.38	9.38	6.77	5.4	3.17	3.18	4.54
hsa-miR-23a-3p	nmiR03141.1:1	Endogenous	3125.66	3496.62	3982.94	4527.37	5458.81	3263.12	3789.46	3268.65	2917.24	3920.23	2219.17	2347	2409.73	2855.88	3256.91	2871.18	3408.43	4070.46	2998.3	3919.89	3039.2	3893.82	1788.22
hsa-miR-23b-3p	nmiR03142.1:1	Endogenous	61.8	65.65	39.22	49.77	50.56	76.28	37.55	49.28	44.64	63.86	47.14	50.72	39.2	81.58	53.95	39.97	76.22	87.54	54.19	53.99	44.34	81.93	44.48
hsa-miR-23c	nmiR03143.1:1	Endogenous	3.51	4.97	4.06	4.15	10.11	16.95	9.83	4.11	8.64	8.19	3.37	4.38	1.33	6.23	4.62	4.36	8.73	7.82	11.29	8.64	7.92	5.08	5.45
hsa-miR-24-3p	nmiR03144.1:1	Endogenous	75.84	59.69	87.91	78.8	72.81	67.81	43.8	45.17	50.4	50.76	60.61	58.86	47.17	71.61	54.72	58.86	77.81	79.72	63.22	63.71	50.68	83.2	52.65
hsa-miR-25-3p	nmiR03145.1:1	Endogenous	827.24	1157.91	1279.41	1267.66	1355.1	1079.23	1598.39	2024.43	1219.59	882.62	858.71	1033.86	886.96	727.98	1156.8	1861.07	1144.08	1202.07	1413.35	1229.96	1208.4	1072.69	1082.01
hsa-miR-25-5p	nmiR03918.1:1	Endogenous	1.4	6.96	4.06	5.53	14.16	11.3	6.26	2.74	1.44	3.28	1.12	1.88	5.98	1.87	3.85	5.81	3.18	3.13	13.55	9.72	4.75	2.54	9.98
hsa-miR-2682-5p	nmiR03864.1:1	Endogenous	4.92	3.98	1.35	2.76	2.02	8.48	2.68	4.11	8.64	1.64	1.12	3.13	2.66	1.87	3.85	4.36	2.38	4.69	4.52	2.16	9.5	2.54	4.54
hsa-miR-26a-5p	nmiR03146.1:1	Endogenous	599.71	587.91	474.71	515.64	426.75	449.21	332.55	217.64	501.09	640.27	653.29	657.51	941.43	864.36	421.56	341.55	596.26	626.83	489.93	455.7	429.19	551.91	597.28
hsa-miR-26b-5p	nmiR03147.1:1	Endogenous	1370.77	1399.64	903.43	1158.45	910.14	1155.51	874.29	662.49	1133.2	954.68	1662.41	2192.95	1571.94	1554.98	1000.35	839.33	1075.8	861.3	1408.84	902.76	1214.73	1327.37	1697.45
hsa-miR-27a-3p	nmiR03148.1:1	Endogenous	8.43	9.95	12.17	12.44	12.14	5.65	6.26	10.95	5.76	8.19	10.1	5.64	9.97	9.96	2.31	5.09	15.09	10.94	13.55	8.64	9.5	9.53	12.71
hsa-miR-27b-3p	nmiR03149.1:1	Endogenous	58.99	64.66	59.51	53.91	93.04	42.38	28.61	38.33	51.84	49.13	70.72	39.45	39.86	64.76	36.22	36.33	69.07	56.27	42.9	43.19	36.43	88.91	33.59
hsa-miR-28-3p	nmiR03150.1:1	Endogenous	23.88	18.9	25.7	11.06	10.11	22.6	14.3	4.11	17.28	26.2	31.43	25.05	21.26	26.78	10.79	9.45	15.09	21.88	18.06	16.2	17.42	20.96	18.15
hsa-miR-28-5p	nmiR03151.1:1	Endogenous	594.8	700.32	481.47	735.44	707.89	632.85	500.61	503.71	522.68	659.92	560.12	540.41	411.25	564.2	506.34	364.07	574.03	551.79	672.81	433.02	505.21	546.82	334.04
hsa-miR-296-3p	nmiR03285.1:1	Endogenous	2.81	4.97	1.35	4.15	16.18	36.73	6.26	8.21	7.2	4.91	8.98	5.64	7.31	4.98	6.94	9.45	8.73	9.38	9.03	8.64	17.42	3.81	8.17
hsa-miR-296-5p	nmiR03286.1:1	Endogenous	13.34	24.87	17.58	12.44	24.27	19.78	25.03	15.06	12.96	13.1	12.35	18.16	10.63	13.08	13.87	26.16	15.09	29.7	33.87	17.28	23.76	15.88	22.69
hsa-miR-297	nmiR03287.1:1	Endogenous	4.21	6.96	1.35	6.91	12.14	11.3	1	6.84	10.08	3.28	1.12	1.88	3.32	1.25	3.08	4.36	1.59	6.25	6.77	5.4	1.58	3.18	5.45
hsa-miR-298	nmiR03288.1:1	Endogenous	6.32	1.99	4.06	1.38	18.2	8.48	2.68	9.58	2.88	16.38	8.98	1.25	1.99	2.49	6.17	4.36	3.97	12.51	6.77	4.32	11.09	4.45	11.8
hsa-miR-299-3p	nmiR03289.1:1	Endogenous	9.13	17.91	16.23	9.68	10.11	19.78	13.41	10.95	11.52	22.93	15.71	14.4	19.93	12.45	15.41	16.71	22.23	20.32	24.84	12.96	23.76	12.7	12.71
hsa-miR-299-5p	nmiR03290.1:1	Endogenous	8.43	12.93	8.11	8.29	26.29	25.43	2.68	15.06	5.76	19.65	3.37	3.76	8.64	5.6	7.71	8.72	13.5	28.14	15.8	9.72	12.67	5.72	5.45
hsa-miR-29a-3p	nmiR03152.1:1	Endogenous	360.25	531.21	355.69	519.78	301.36	305.12	411.22	277.86	305.26	239.08	370.42	529.14	356.78	343.13	375.32	320.47	473.99	348.58	557.67	420.07	433.95	409.64	605.45
hsa-miR-29b-3p	nmiR03153.1:1	Endogenous	6252.73	8345.15	5785.75	6115.75	4712.5	5735.18	4762.98	3115.34	5563.77	5104.16	7652.05	8477.5	7055.78	6932.94	5467.23	3230.89	5268.66	3881.32	8006	4513.81	5918.45	6243.71	5787.66
hsa-miR-29c-3p	nmiR03155.1:1	Endogenous	495.78	765.97	499.05	673.23	566.31	468.99	491.67	266.91	457.89	384.82	517.47	541.66	572.04	514.38	483.99	322.65	570.85	429.87	548.63	512.93	536.89	599.54	648.12
hsa-miR-300	nmiR03291.1:1	Endogenous	3.51	1.99	6.76	5.53	6.07	11.3	8.05	9.58	8.64	3.28	3.37	6.26	1.99	3.11	3.08	3.63	3.18	4.69	13.55	3.24	9.5	5.08	6.35
hsa-miR-301a-3p	nmiR03292.1:1	Endogenous	123.59	93.51	152.83	141.01	177.98	104.53	116.21	113.61	123.83	152.29	103.27	99.57	103.64	133.89	137.18	111.18	152.44	126.62	124.18	153.34	91.86	151.79	88.05
hsa-miR-301a-5p	nmiR03965.1:1	Endogenous	4.21	3.98	2.7	4.15	12.14	16.95	5.36	13.69	12.96	18.01	6.73	6.26	5.32	4.98	4.62	3.63	3.18	7.82	18.06	16.2	12.67	5.72	11.8
hsa-miR-301b-3p	nmiR03293.1:1	Endogenous	9.13	2.98	5.41	5.53	12.14	14.13	6.26	6.84	8.64	9.83	12.35	3.13	1.99	4.36	7.71	5.81	7.15	10.94	13.55	4.32	14.25	6.99	10.89
hsa-miR-301b-5p	nmiR04034.1:1	Endogenous	6.32	3.98	4.06	8.29	2.02	8.48	3.58	4.11	2.88	11.46	4.49	1.88	3.32	3.11	5.39	6.54	7.15	6.25	4.52	8.64	6.33	6.99	6.35
hsa-miR-302a-3p	nmiR03294.1:1	Endogenous	3.51	1.99	1.35	8.29	4.05	2.83	6.26	8.21	5.76	6.55	8.98	1.88	4.65	3.11	3.85	6.54	6.35	9.38	6.77	4.32	4.75	3.18	7.26
hsa-miR-302a-5p	nmiR03915.1:1	Endogenous	4.21	2.98	2.7	1.38	10.11	2.83	4.47	8.21	5.76	4.91	3.37	3.76	5.32	3.11	3.08	5.81	3.97	4.69	6.77	7.56	4.75	3.81	4.54
hsa-miR-302b-3p	nmiR03295.1:1	Endogenous	9.83	14.92	13.52	5.53	26.29	39.55	17.88	30.11	23.04	19.65	6.73	6.26	10.63	5.6	12.33	13.08	8.73	29.7	42.9	21.6	26.92	9.53	15.43
hsa-miR-302c-3p	nmiR03296.1:1	Endogenous	5.62	12.93	6.76	6.91	20.23	16.95	5.36	9.58	5.76	9.83	6.73	6.26	3.32	5.6	3.08	7.27	3.18	12.51	15.8	7.56	11.09	6.35	18.15
hsa-miR-302d-3p	nmiR03297.1:1	Endogenous	1	1	1.35	1.38	2.02	2.83	1	1.37	1.44	1.64	1.12	1	1	1	1	1	1	1.56	2.26	1.08	1.58	1	1
hsa-miR-302e	nmiR03298.1:1	Endogenous	2.81	15.92	8.11	8.29	14.16	8.48	4.47	12.32	8.64	8.19	5.61	2.5	4.65	3.74	10.02	4.36	4.76	7.82	9.03	4.32	7.92	5.08	10.89
hsa-miR-302f	nmiR03299.1:1	Endogenous	1	3.98	4.06	1.38	12.14	11.3	4.47	10.95	10.08	6.55	7.86	3.76	3.32	4.36	6.17	2.91	2.38	7.82	13.55	2.16	1.58	10.8	8.17
hsa-miR-3065-3p	nmiR03933.1:1	Endogenous	2.81	1	4.06	2.76	2.02	5.65	2.68	6.84	5.76	1.64	11.22	1	4.65	3.11	3.08	2.91	5.56	10.94	4.52	3.24	6.33	3.18	3.63
hsa-miR-3065-5p	nmiR03931.1:1	Endogenous	1	1	1.35	1.38	2.02	2.83	1	1.37	1.44	1.64	1.12	1	1	1	1	1	1	1.56	2.26	1.08	1.58	1	1
hsa-miR-3074-3p	nmiR03928.1:1	Endogenous	2.11	15.92	4.06	11.06	4.05	8.48	13.41	20.53	4.32	13.1	10.1	4.38	9.3	3.11	4.62	12.35	10.32	14.07	6.77	2.16	11.09	4.45	3.63
hsa-miR-30a-3p	nmiR03912.1:1	Endogenous	2.11	4.97	8.11	6.91	2.02	16.95	7.15	1.37	4.32	6.55	4.49	3.76	6.64	2.49	6.94	2.18	5.56	7.82	9.03	7.56	11.09	3.81	5.45
hsa-miR-30a-5p	nmiR03156.1:1	Endogenous	16.85	17.91	20.29	27.65	28.32	11.3	24.14	12.32	17.28	18.01	20.2	16.91	25.25	21.8	19.27	14.53	20.64	20.32	24.84	15.12	14.25	18.42	19.97
hsa-miR-30b-5p	nmiR03157.1:1	Endogenous	109.55	82.57	77.09	60.83	52.59	70.63	55.43	36.96	86.39	98.25	115.62	110.21	139.52	127.66	63.2	45.78	74.63	95.35	90.31	69.11	71.27	73.67	110.74
hsa-miR-30c-5p	nmiR03158.1:1	Endogenous	37.22	48.74	45.98	30.41	36.41	33.9	36.65	21.9	30.24	27.84	32.55	41.33	50.49	43.59	43.93	31.97	38.9	43.77	56.44	32.4	25.34	43.19	53.56
hsa-miR-30d-5p	nmiR03159.1:1	Endogenous	136.23	172.1	190.69	178.33	232.59	180.81	169.85	160.15	139.67	122.81	115.62	105.83	105.64	134.51	165.7	148.25	176.26	129.74	119.66	165.22	158.37	161.32	124.36
hsa-miR-30e-3p	nmiR03916.1:1	Endogenous	42.13	42.78	37.87	64.97	64.72	42.38	46.49	34.22	41.76	40.94	29.18	23.8	39.86	31.76	44.7	43.6	47.64	50.02	31.61	48.59	53.85	52.08	36.31
hsa-miR-30e-5p	nmiR03160.1:1	Endogenous	132.72	189.01	139.3	152.06	143.6	132.78	140.35	98.55	128.15	88.43	144.8	169.07	158.12	135.76	128.7	122.81	132.59	106.29	155.79	115.54	126.7	147.98	168.84

hsa-miR-31-5p	nmiR03161.1:1	Endogenous	4.21	11.94	10.82	8.29	6.07	11.3	8.05	2.74	11.52	4.91	6.73	6.26	7.97	5.6	5.39	7.27	5.56	6.25	18.06	2.16	12.67	6.35	9.98
hsa-miR-3127-5p	nmiR03866.1:1	Endogenous	1.4	10.94	2.7	1.38	6.07	2.83	1	10.95	4.32	9.83	2.24	2.5	4.65	1.87	3.85	2.18	7.15	10.94	11.29	5.4	7.92	2.54	3.63
hsa-miR-3130-3p	nmiR03925.1:1	Endogenous	4.92	2.98	5.41	1.38	14.16	5.65	6.26	2.74	4.32	1.64	4.49	1	1.99	3.11	1.54	2.91	3.18	3.13	11.29	3.24	9.5	3.81	1.82
hsa-miR-3131	nmiR03867.1:1	Endogenous	4.21	3.98	10.82	9.68	6.07	11.3	7.15	10.95	10.08	8.19	4.49	3.76	5.98	1.87	5.39	5.81	1.59	10.94	15.8	5.4	14.25	6.99	9.98
hsa-miR-3136-5p	nmiR03868.1:1	Endogenous	2.11	3.98	1.35	5.53	6.07	11.3	4.47	5.48	4.32	6.55	6.73	1.88	4.65	4.36	4.62	1	2.38	9.38	9.03	5.4	12.67	5.72	6.35
hsa-miR-3140-3p	nmiR03926.1:1	Endogenous	2.81	1	1.35	1.38	6.07	8.48	3.58	1.37	4.32	3.28	1.12	1	1.33	1.25	2.31	2.18	3.97	1.56	2.26	5.4	6.33	2.54	1
hsa-miR-3140-5p	nmiR03944.1:1	Endogenous	2.81	3.98	2.7	1.38	2.02	5.65	1	5.48	2.88	6.55	4.49	1.25	1.33	6.23	4.62	1.45	5.56	6.25	4.52	1.08	6.33	6.35	2.72
hsa-miR-3144-3p	nmiR03927.1:1	Endogenous	1	1	1.35	1.38	2.02	2.83	1	1.37	1.44	1.64	1.12	1	1	1	1	1	1	1.56	2.26	1.08	1.58	1	1
hsa-miR-3144-5p	nmiR03870.1:1	Endogenous	1.4	7.96	6.76	1.38	12.14	2.83	2.68	13.69	4.32	11.46	6.73	3.13	5.32	3.11	4.62	4.36	6.35	12.51	15.8	5.4	4.75	2.54	7.26
hsa-miR-3147	nmiR03871.1:1	Endogenous	7.02	10.94	2.7	4.15	8.09	16.95	6.26	16.43	4.32	4.91	12.35	6.26	6.64	4.98	3.08	4.36	7.15	9.38	22.58	6.48	6.33	7.62	6.35
hsa-miR-3150b-3p	nmiR03941.1:1	Endogenous	2.81	1.99	1.35	2.76	2.02	2.83	7.15	5.48	2.88	6.55	5.61	1.25	1.99	1	2.31	3.63	3.18	1.56	4.52	1.08	3.17	5.72	1.82
hsa-miR-3151-5p	nmiR03872.1:1	Endogenous	5.62	5.97	2.7	1.38	12.14	16.95	9.83	13.69	5.76	11.46	5.61	4.38	5.98	3.74	5.39	2.91	10.32	10.94	13.55	9.72	6.33	8.26	9.08
hsa-miR-3158-3p	nmiR03929.1:1	Endogenous	4.92	7.96	4.06	4.15	10.11	8.48	3.58	5.48	7.2	8.19	6.73	3.76	7.97	5.6	4.62	5.81	2.38	6.25	9.03	7.56	4.75	1.91	3.63
hsa-miR-3161	nmiR03874.1:1	Endogenous	1.4	4.97	6.76	1.38	4.05	2.83	2.68	6.84	4.32	11.46	2.24	2.5	4.65	1.87	3.85	5.09	2.38	6.25	6.77	1.08	4.75	3.18	9.98
hsa-miR-3164	nmiR03875.1:1	Endogenous	7.02	3.98	2.7	4.15	6.07	16.95	8.94	6.84	4.32	6.55	5.61	1.25	2.66	3.11	8.48	5.81	3.97	6.25	11.29	7.56	12.67	1.27	6.35
hsa-miR-3168	nmiR03876.1:1	Endogenous	1.4	3.98	2.7	1.38	2.02	8.48	1	8.21	1.44	6.55	6.73	1	1.33	2.49	2.31	1	3.18	4.69	6.77	6.48	22.17	3.18	5.45
hsa-miR-3179	nmiR03930.1:1	Endogenous	2.11	4.97	1.35	4.15	2.02	5.65	3.58	5.48	1.44	4.91	7.86	3.13	1.99	2.49	1	2.18	2.38	1.56	2.26	3.24	3.17	1.27	1.82
hsa-miR-3180	nmiR03881.1:1	Endogenous	9.13	11.94	2.7	4.15	18.2	14.13	2.68	9.58	7.2	9.83	3.37	5.64	8.64	6.23	7.71	5.81	11.91	9.38	27.09	10.8	20.59	5.72	8.17
hsa-miR-3180-3p	nmiR03879.1:1	Endogenous	1	2.98	2.7	1.38	2.02	14.13	1	5.48	2.88	1.64	2.24	1	3.32	1.25	2.31	3.63	7.94	9.38	15.8	7.56	6.33	6.35	1
hsa-miR-3180-5p	nmiR03880.1:1	Endogenous	9.83	3.98	2.7	6.91	12.14	11.3	16.99	27.38	10.08	6.55	3.37	5.01	8.64	4.98	10.02	18.17	7.94	7.82	15.8	7.56	17.42	8.26	14.52
hsa-miR-3182	nmiR03882.1:1	Endogenous	6.32	5.97	5.41	1.38	8.09	11.3	4.47	4.11	4.32	8.19	4.49	7.51	2.66	1.25	5.39	1.45	3.97	4.69	6.77	9.72	4.75	3.18	7.26
hsa-miR-3185	nmiR03884.1:1	Endogenous	2.81	3.98	4.06	1.38	6.07	2.83	3.58	8.21	1.44	4.91	2.24	3.13	5.32	2.49	3.08	2.91	4.76	4.69	6.77	4.32	6.33	3.18	4.54
hsa-miR-3190-3p	nmiR03886.1:1	Endogenous	9.13	10.94	1.35	4.15	10.11	8.48	6.26	12.32	5.76	16.38	11.22	3.76	13.29	7.47	8.48	9.45	9.53	18.76	13.55	21.6	11.09	8.89	14.52
hsa-miR-3192-5p	nmiR03887.1:1	Endogenous	2.11	2.98	1.35	2.76	8.09	16.95	6.26	4.11	2.88	1.64	2.24	2.5	3.99	4.98	1	2.91	1.59	3.13	4.52	1.08	6.33	1.27	1
hsa-miR-3195	nmiR03889.1:1	Endogenous	7.02	6.96	1.35	5.53	6.07	8.48	17.88	13.69	7.2	3.28	12.35	27.55	11.96	11.83	9.25	11.63	3.97	9.38	6.77	8.64	17.42	12.7	15.43
hsa-miR-3196	nmiR03890.1:1	Endogenous	2.81	3.98	1.35	4.15	4.05	2.83	5.36	5.48	2.88	3.28	5.61	1.25	1	1	3.08	6.54	3.18	3.13	2.26	2.16	6.33	1.27	1.82
hsa-miR-32-5p	nmiR03162.1:1	Endogenous	468.39	555.08	628.89	543.28	570.35	531.14	581.07	505.08	470.85	445.41	398.49	455.25	366.74	453.98	527.15	454.19	543.86	442.37	492.19	481.62	506.8	567.15	394.86
hsa-miR-3202	nmiR03932.1:1	Endogenous	2.11	3.98	1.35	4.15	2.02	2.83	1.79	1.37	5.76	6.55	5.61	1.25	2.66	1.87	2.31	5.81	3.97	7.82	11.29	5.4	11.09	3.18	4.54
hsa-miR-320a	nmiR03300.1:1	Endogenous	4.92	7.96	9.47	4.15	16.18	19.78	7.15	6.84	12.96	16.38	14.59	3.76	6.64	4.36	13.87	7.27	9.53	18.76	9.03	7.56	11.09	8.26	9.08
hsa-miR-320b	nmiR03301.1:1	Endogenous	2.11	2.98	1.35	4.15	8.09	2.83	2.68	5.48	4.32	6.55	1.12	3.76	1.33	2.49	1.54	2.18	3.18	4.69	11.29	4.32	11.09	4.45	1.82
hsa-miR-320c	nmiR03302.1:1	Endogenous	10.53	24.87	9.47	6.91	18.2	25.43	16.09	20.53	7.2	14.74	29.18	10.65	17.27	8.72	12.33	14.53	18.26	18.76	22.58	18.36	17.42	10.16	11.8
hsa-miR-320d	nmiR03303.1:1	Endogenous	2.11	7.96	2.7	6.91	10.11	8.48	4.47	10.95	11.52	9.83	5.61	3.13	9.3	3.11	6.17	13.81	3.18	7.82	15.8	7.56	11.09	5.72	4.54
hsa-miR-320e	nmiR03304.1:1	Endogenous	10.53	10.94	17.58	6.91	6.07	16.95	8.94	16.43	18.72	16.38	15.71	13.78	16.61	9.96	12.33	9.45	8.73	15.63	20.32	7.56	23.76	7.62	11.8
hsa-miR-323a-3p	nmiR03307.1:1	Endogenous	14.75	22.88	14.88	16.59	18.2	25.43	8.94	21.9	14.4	27.84	11.22	6.26	13.95	10.59	6.94	10.9	34.14	34.39	18.06	14.04	22.17	6.99	8.17
hsa-miR-323a-5p	nmiR03308.1:1	Endogenous	3.51	1.99	5.41	5.53	16.18	5.65	4.47	10.95	2.88	4.91	8.98	5.64	2.66	3.11	5.39	7.27	2.38	4.69	4.52	2.16	3.17	4.45	4.54
hsa-miR-323b-3p	nmiR03305.1:1	Endogenous	9.13	16.91	10.82	5.53	24.27	25.43	21.45	38.33	17.28	16.38	8.98	8.14	15.28	11.83	10.02	26.89	11.12	15.63	18.06	7.56	15.84	13.34	24.51
hsa-miR-323b-5p	nmiR03306.1:1	Endogenous	1.4	3.98	2.7	2.76	4.05	11.3	7.15	4.11	4.32	6.55	4.49	1.25	1	2.49	3.85	5.81	2.38	3.13	15.8	5.4	4.75	2.54	3.63
hsa-miR-324-3p	nmiR03309.1:1	Endogenous	4.92	11.94	4.06	6.91	6.07	16.95	7.15	17.79	4.32	8.19	7.86	2.5	1.99	6.23	7.71	2.18	5.56	14.07	9.03	11.88	6.33	6.35	5.45
hsa-miR-324-5p	nmiR03310.1:1	Endogenous	28.79	21.88	40.57	24.88	38.43	28.25	20.56	16.43	27.36	16.38	17.96	18.79	13.95	24.29	25.43	27.61	28.58	28.14	33.87	24.84	15.84	28.58	22.69
hsa-miR-325	nmiR03311.1:1	Endogenous	5.62	13.93	8.11	11.06	22.25	31.08	8.94	9.58	12.96	16.38	7.86	3.13	5.98	8.72	9.25	10.17	7.94	14.07	15.8	14.04	19	5.08	14.52
hsa-miR-326	nmiR03312.1:1	Endogenous	7.72	6.96	10.82	5.53	12.14	16.95	13.41	16.43	12.96	13.1	13.47	8.14	8.64	6.85	8.48	9.45	6.35	12.51	6.77	6.48	14.25	10.16	16.34
hsa-miR-328-3p	nmiR03313.1:1	Endogenous	7.02	7.96	2.7	2.76	6.07	8.48	2.68	4.11	10.08	4.91	12.35	6.26	6.64	3.74	6.94	1.45	6.35	6.25	9.03	3.24	15.84	2.54	1.82
hsa-miR-328-5p	nmiR04008.1:1	Endogenous	3.51	4.97	1.35	4.15	8.09	19.78	4.47	9.58	1.44	3.28	6.73	3.13	2.66	5.6	3.85	6.54	4.76	3.13	9.03	7.56	6.33	3.18	5.45
hsa-miR-329-3p	nmiR03314.1:1	Endogenous	4.21	5.97	4.06	9.68	12.14	11.3	5.36	5.48	5.76	11.46	2.24	4.38	2.66	1.25	4.62	4.36	2.38	9.38	4.52	8.64	3.17	2.54	3.63
hsa-miR-329-5p	nmiR04010.1:1	Endogenous	4.92	9.95	6.76	1.38	12.14	14.13	3.58	10.95	7.2	18.01	4.49	3.13	2.66	3.11	5.39	5.81	6.35	10.94	11.29	7.56	9.5	7.62	9.98
hsa-miR-330-3p	nmiR03315.1:1	Endogenous	9.83	5.97	8.11	4.15	12.14	5.65	4.47	9.58	5.76	6.55	6.73	5.01	1.99	4.98	8.48	9.45	7.15	12.51	29.35	6.48	11.09	6.35	9.08
hsa-miR-330-5p	nmiR03316.1:1	Endogenous	4.21	4.97	5.41	5.53	18.2	8.48	9.83	15.06	14.4	14.74	7.86	3.13	10.63	5.6	6.94	7.27	7.94	17.19	13.55	8.64	20.59	7.62	9.08
hsa-miR-331-3p	nmiR03317.1:1	Endogenous	23.88	24.87	20.29	19.35	28.32	16.95	21.45	12.32	21.6	19.65	25.82	25.67	15.28	21.8	18.5	29.79	28.58	31.26	31.61	16.2	36.43	31.12	14.52
hsa-miR-331-5p	nmiR03318.1:1	Endogenous	4.92	2.98	6.76	1.38	6.07	2.83	5.36	5.48	10.08	6.55	4.49	1.25	4.65	3.11	6.94	5.81	3.97	9.38	18.06	6.48	4.75	1.91	4.54
hsa-miR-335-5p	nmiR03319.1:1	Endogenous	26.69	18.9	22.99	15.21	34.38	19.78	13.41	10.95	18.72	18.01	16.84	15.03	13.29	21.8	11.56	15.99	22.23	35.95	22.58	21.6	15.84	28.58	14.52
hsa-miR-337-3p	nmiR03320.1:1	Endogenous	14.04	12.93	10.82	23.5	16.18	14.13	11.62	15.06	12.96	16.38	11.22	8.14	13.95	9.96	5.39	10.17	25.41	39.08	18.06	8.64	7.92	6.99	8.17
hsa-miR-337-5p	nmiR03321.1:1	Endogenous	15.45	9.95	21.64	15.21	6.07	16.95	8.05	4.11	11.52	24.56	8.98	4.38	6.64	11.83	7.71	10.17	20.64	28.14	6.77	8.64	9.5	4.45	4.54
hsa-miR-338-5p	nmiR03323.1:1	Endogenous	2.11	1.99	1.35	5.53	6.07	5.65	1.79	4.11	4.32	4.91	5.61	1.25	3.32	2.49	2.31	1	3.18	12.51	9.03	6.48	6.33	1.91	4.54
hsa-miR-339-3p	nmiR03324.1:1	Endogenous	4.92	8.95	5.41	8.29	10.11	2.83	6.26	4.11	5.76	6.55	12.35	4.38	1.33	7.47	6.17	2.91	7.94	7.82	9.03	7.56	11.09	8.26	9.98
hsa-miR-339-5p	nmiR03325.1:1	Endogenous	3.51	6.96	4.06	6.91	10.11	2.83	1	5.48	4.32	19.65	7.86	3.76	3.32	4.98	6.17	4.36	7.94	9.38	15.8	7.56	6.33	5.08	4.54
hsa-miR-33a-5p	nmiR03163.1:1	Endogenous	23.88	13.93	31.11	23.5	28.32	31.08	8.94	21.9	14.4	11.46	14.59	11.9	9.3	13.7	16.96	12.35	22.23	17.19	18.06	17.28	17.42	24.13	18.15
hsa-miR-33b-5p	nmiR03164.1:1	Endogenous	2.11	3.98	1.35	2.76	4.05	8.48	5.36	4.11	5.76	9.83	3.37	3.76	1	3.11	3.08	3.63	3.97	1.56	11.29	4.32	7.92	3.81	3.63
hsa-miR-340-5p	nmiR03326.1:1	Endogenous	340.59	238.75	382.74	341.45	303.38	330.55	235.11	157.41	240.46	412.66	344.61	350.05	386.01	395.44	292.86	211.47	273.91	353.27	209.97	375.79	229.64	337.88	241.46
hsa-miR-342-3p	nmiR03328.1:1	Endogenous	2337.75	3485.68	2228.82	3037.14	2536.26	2361.88	2040.89	1092.29	2302.4	1562.2	3631.27	3143.53	3123.94	2548.87	2086.24	983.95	2143.67	1630.37	3345.99	1551.76	2225.16	2348.62	2954.65
hsa-miR-342-5p	nmiR03329.1:1	Endogenous	13.34	28.85	14.88	19.35	40.45	14.13	19.67	10.95	17.28	8.19	24.69	16.28	19.93	12.45	11.56	12.35	15.88	18.76	9.03	17.28	26.92	19.69	19.97
hsa-miR-345-3p	nmiR03967.1:1	Endogenous	1	3.98	6.76	1.38	6.07	2.83	1	8.21	10.08	3.28	3.37	3.13	1.99	3.11	3.08	6.54	1	14.07	9.03	5.4	9.5	1	2.72
hsa-miR-345-5p	nmiR03330.1:1	Endogenous	11.24	15.92	18.93	22.12	4.05	22.6	25.03	13.69	18.72	13.1	21.33	14.4	15.95	16.19	17.73	20.35	17.47	14.07	9.03	17.28	17.42	16.51	14.52
hsa-miR-346	nmiR03331.1:1	Endogenous	4.92	4.97	2.7	5.53	6.07	5.65	5.36	12.32	8.64	4.91	7.86	1.88	5.32	2.49	3.08	2.18	4.76	7.82	20.32	5.4	4.75	5.72	7.26
hsa-miR-34a-5p	nmiR03165.1:1	Endogenous	9.83	5.97	10.82	12.44	24.27	25.43	9.83	9.58	10.08	16.38	6.73	10.65	6.64	9.34	9.25	7.27	9.53	6.25	24.84	14.04	23.76	8.26	10.89
hsa-miR-34b-3p	nmiR03166.1:1	Endogenous	11.24	11.94	16.23	5.53	10.11	19.78	8.05	13.69	4.32	13.1	12.35	5.01	4.65	7.47	10.02	5.09	5.56	7.82	9.03	5.4	14.25	5.72	14.52
hsa-miR-34c-3p	nmiR03167.1:1	Endogenous	3.51	10.94	10.82	11.06	12.14	16.95	5.36	9.58	4.32	9.83	7.86	4.38	5.98	5.6	10.02	7.99	1.59	26.57	15.8	15.12	20.59	6.35	11.8
hsa-miR-34c-5p	nmiR03168.1:1	Endogenous	13.34	5.97	5.41	8.29	16.18	8.48	8.05	8.21	14.4	9.83	7.86	5.01	3.32	2.49	5.39	5.09	3.97	4.69	6.77	8.64	9.5	4.45	9.08
hsa-miR-3605-3p	nmiR03934.1:1	Endogenous	2.81	2.98	2.7	2.76	2.02	5.65	1	2.74	4.32	3.28	3.37	1.88	3.32	3.11	1.54	2.18	1.59	1.56	4.52	9.72	7.92	3.18	2.72
hsa-miR-3605-5p	nmiR03892.1:1	Endogenous	1	1.99	4.06	2.76	2.02	2.83	1.79	4.11	4.32	4.91	3.37	2.5	1.99	1.25	3.08	1.45	3.18	3.13	2.26	4.32	9.5	2.54	3.63
hsa-miR-361-3p	nmiR03332.1:1	Endogenous	127.81	177.07	160.94	170.04	125.4	146.91	135.88	93.08	135.35	140.83	132.45	139.64	127.56	124.55	135.64	71.94	153.23	68.78	173.85	101.51	136.2	149.25	138.88
hsa-miR-361-5p	nmiR03333.1:1	Endogenous	131.32	128.33	135.24	135.48	153.71	101.71	81.35	52.01	115.19	124.45	144.8	113.97	114.94	153.82	118.69	78.48	140.53	84.41	133.21	115.54	83.94	149.25	127.08
hsa-miR-3613-3p	nmiR03936.1:1	Endogenous	2.11	3.98	6.76	1.38	6.07	8.48	1.79	4.11	4.32	4.91	1.12	1.25	1.99	3.74	4.62	3.63	3.97	4.69	11.29	1.08	6.33	5.08	4.54
hsa-miR-3613-5p	nmiR03935.1:1	Endogenous	3.51	4.97	5.41	8.29	8.09	11.3	2.68	8.21	2.88	4.91	2.24	3.76	6.64	5.6	4.62	8.72	3.18	9.38	15.8	10.8	15.84	2.54	4.54
hsa-miR-3614-3p	nmiR03937.1:1	Endogenous	2.81	4.97	10.82	1.38	4.05	5.65	11.62	15.06	7.2	6.55	8.98	5.64	5.32	6.23	6.94	10.17	1.59	6.25	4.52	5.4	11.09	5.08	5.45
hsa-miR-3614-5p	nmiR03893.1:1	Endogenous	6.32	2.98	4.06	8.29	8.09	11.3	6.26	4.11	5.76	8.19	4.49	2.5	2.66	3.11	3.08	4.36	4.76	14.07	9.03	10.8	14.25	3.81	4.54
hsa-miR-3615	nmiR03938.1:1	Endogenous	6.32	5.97	2.7	5.53	18.2	22.6	8.94	10.95	10.08	13.1	6.73	5.01	8.64	7.47	6.17	9.45	5.56	15.63	13.55	6.48	26.92	6.35	11.8
hsa-miR-362-3p	nmiR03334.1:1	Endogenous	16.85	11.94	20.29	13.82	18.2	11.3	16.09	13.69	5.76	16.38	21.33	18.16	15.28	20.55	12.33	13.08	6.35	17.19	15.8	20.52	9.5	19.69	17.25
hsa-miR-362-5p	nmiR03335.1:1	Endogenous	33.71	24.87	40.57	45.62	34.38	28.25	33.08	31.48	28.8	27.84	20.2	25.67	16.61	26.15	37.76	17.44	21.44	31.26	56.44	46.43	47.51	34.3	22.69
hsa-miR-363-3p	nmiR03336.1:1	Endogenous	93.4	123.35	91.97	149.3	141.58	189.29	109.06	182.05	131.03	111.35	125.72	98.94	103.64	105.24	141.81	218.74	84.16	106.29	151.27	93.95	137.79	129.56	173.38
hsa-miR-363-5p	nmiR03917.1:1	Endogenous	1.4	4.97	1.35	2.76	8.09	2.83	4.47	4.11	4.32	3.28	1.12	3.13	3.99	3.74	2.31	1.45	2.38	9.38	20.32	5.4	6.33	3.18	9.08
hsa-miR-365a-3p+hsa-miR-365b-3p	nmiR03337.1:1	Endogenous	16.85	7.96	12.17	6.91	16.18	16.95	15.2	10.95	10.08	11.46	13.47	20.04	40.53	20.55	14.64	15.99	8.73	43.77	22.58	24.84	25.34	16.51	20.88
hsa-miR-365b-5p	nmiR03985.1:1	Endogenous	2.81	9.95	8.11	6.91	10.11	11.3	12.52	15.06	4.32	6.55	6.73	5.01	8.64	8.1	6.94	10.9	13.5	14.07	9.03	9.72	11.09	7.62	7.26
hsa-miR-367-3p	nmiR03154.1:1	Endogenous	4.21	3.98	8.11	2.76	6.07	8.48	13.41	16.43	5.76	11.46	4.49	6.89	7.31	4.98	8.48	12.35	7.94	3.13	9.03	4.32	9.5	5.72	8.17
hsa-miR-369-3p	nmiR03338.1:1	Endogenous	4.92	5.97	5.41	8.29	6.07	8.48	7.15	5.48	8.64	9.83	8.98	2.5	2.66	5.6	3.85	5.09	6.35	7.82	4.52	5.4	6.33	8.26	9.08
hsa-miR-369-5p	nmiR03339.1:1	Endogenous	2.11	1.99	4.06	2.76	2.02	11.3	1.79	2.74	2.88	4.91	3.37	1.88	1.99	1.25	1	1.45	5.56	4.69	4.52	3.24	4.75	4.45	2.72
hsa-miR-3690	nmiR03895.1:1	Endogenous	10.53	22.88	20.29	15.21	22.25	14.13	24.14	15.06	12.96	3.28	21.33	11.27	19.93	13.08	16.18	17.44	17.47	15.63	13.55	12.96	23.76	17.15	22.69
hsa-miR-370-3p	nmiR03340.1:1	Endogenous	7.72	5.97	5.41	8.29	4.05	16.95	7.15	10.95	5.76	13.1	7.86	4.38	5.32	3.11	3.85	5.81	7.94	15.63	11.29	6.48	9.5	6.99	3.63
hsa-miR-370-5p	nmiR04007.1:1	Endogenous	12.64	21.88	12.17	12.44	16.18	19.78	22.35	10.95	21.6	18.01	6.73	12.52	11.96	12.45	8.48	7.27	16.67	20.32	24.84	5.4	30.09	12.07	11.8
hsa-miR-371a-5p	nmiR03345.1:1	Endogenous	10.53	12.93	5.41	9.68	8.09	14.13	6.26	16.43	5.76	14.74	11.22	6.26	2.66	6.85	11.56	6.54	11.91	12.51	18.06	11.88	23.76	5.08	15.43
hsa-miR-371b-5p	nmiR03343.1:1	Endogenous	2.81	3.98	5.41	4.15	4.05	8.48	5.36	6.84	5.76	4.91	3.37	3.13	5.32	1.25	5.39	7.99	1.59	6.25	15.8	4.32	4.75	3.81	1.82
hsa-miR-372-3p	nmiR03346.1:1	Endogenous	3.51	2.98	5.41	2.76	2.02	5.65	4.47	4.11	5.76	8.19	2.24	3.76	1	1.25	6.94	2.91	1	3.13	9.03	5.4	4.75	4.45	6.35
hsa-miR-373-3p	nmiR03347.1:1	Endogenous	1	7.96	2.7	2.76	16.18	2.83	7.15	4.11	5.76	4.91	3.37	1.25	1.33	2.49	3.08	1.45	3.97	6.25	18.06	3.24	3.17	2.54	5.45
hsa-miR-374a-3p	nmiR03924.1:1	Endogenous	4.92	8.95	5.41	1.38	20.23	19.78	8.94	13.69	15.84	6.55	10.1	8.77	1.33	3.11	3.08	7.99	8.73	7.82	9.03	6.48	12.67	5.08	9.98
hsa-miR-374a-5p	nmiR03348.1:1	Endogenous	1993.65	1975.62	1860.96	2065.31	2220.74	1745.98	1702.09	1754.78	1743.72	1897.89	1802.73	1929.32	1612.46	1964.74	1722.48	1922.84	2061.1	1824.21	1677.51	1788.25	1458.63	1974.54	1456.9
hsa-miR-374b-5p	nmiR03349.1:1	Endogenous	117.98	123.35	85.2	92.62	125.4	84.76	87.61	71.18	70.56	99.89	124.6	129	104.31	128.28	100.96	103.19	158	121.93	85.79	102.59	77.6	122.58	103.48
hsa-miR-374c-5p	nmiR03350.1:1	Endogenous	2.81	4.97	5.41	2.76	8.09	8.48	6.26	6.84	2.88	6.55	3.37	1.88	1.99	1.87	4.62	4.36	4.76	6.25	6.77	5.4	1.58	3.18	5.45
hsa-miR-375	nmiR03351.1:1	Endogenous	2.11	2.98	1.35	2.76	4.05	11.3	2.68	5.48	18.72	4.91	2.24	1	3.32	3.11	2.31	2.91	4.76	12.51	11.29	4.32	4.75	5.08	8.17
hsa-miR-376a-2-5p	nmiR03992.1:1	Endogenous	7.72	4.97	9.47	5.53	18.2	14.13	9.83	8.21	2.88	9.83	11.22	1.88	4.65	4.98	2.31	2.91	7.94	4.69	11.29	7.56	11.09	5.08	4.54
hsa-miR-376a-3p	nmiR03352.1:1	Endogenous	51.97	43.77	62.21	60.83	70.79	67.81	38.44	72.55	36	50.76	35.92	16.91	39.2	39.23	35.45	46.51	84.95	118.8	33.87	32.4	45.93	28.58	26.32
hsa-miR-376b-3p	nmiR03353.1:1	Endogenous	4.21	3.98	5.41	4.15	4.05	14.13	3.58	4.11	10.08	9.83	6.73	1.88	5.98	6.85	3.08	7.27	5.56	6.25	6.77	8.64	15.84	5.72	5.45
hsa-miR-376c-3p	nmiR03354.1:1	Endogenous	14.04	14.92	10.82	19.35	20.23	16.95	9.83	12.32	8.64	21.29	7.86	5.64	11.29	11.21	4.62	13.81	19.85	39.08	4.52	11.88	6.33	6.35	11.8
hsa-miR-376c-5p	nmiR03988.1:1	Endogenous	1.4	2.98	5.41	2.76	4.05	2.83	1	4.11	2.88	3.28	1.12	1.25	1	1.87	1.54	3.63	2.38	1.56	6.77	1.08	6.33	1.27	1.82
hsa-miR-377-3p	nmiR03176.1:1	Endogenous	19.66	7.96	18.93	12.44	26.29	11.3	8.05	16.43	10.08	4.91	8.98	3.13	6.64	11.83	5.39	15.99	21.44	26.57	11.29	11.88	14.25	10.8	9.98
hsa-miR-378b	nmiR03355.1:1	Endogenous	7.02	3.98	8.11	4.15	14.16	11.3	8.05	8.21	11.52	6.55	3.37	5.01	8.64	8.72	3.85	2.18	4.76	7.82	13.55	10.8	12.67	7.62	9.98
hsa-miR-378c	nmiR03356.1:1	Endogenous	5.62	4.97	2.7	2.76	4.05	16.95	3.58	5.48	1.44	1.64	3.37	2.5	3.32	1.25	1	4.36	2.38	7.82	15.8	7.56	6.33	3.18	6.35
hsa-miR-378d	nmiR03358.1:1	Endogenous	5.62	7.96	12.17	6.91	4.05	14.13	6.26	6.84	10.08	11.46	7.86	3.13	4.65	6.85	10.02	10.9	11.12	9.38	22.58	15.12	19	9.53	3.63
hsa-miR-378e	nmiR03359.1:1	Endogenous	1	1	1.35	1.38	2.02	2.83	1	1.37	1.44	1.64	1.12	1	1	1	1	1	1	1.56	2.26	1.08	1.58	1	1
hsa-miR-378f	nmiR03360.1:1	Endogenous	2.11	5.97	2.7	4.15	4.05	2.83	8.94	6.84	1.44	4.91	4.49	2.5	2.66	6.23	7.71	2.91	4.76	4.69	4.52	3.24	11.09	3.81	1
hsa-miR-378g	nmiR03361.1:1	Endogenous	26.69	43.77	70.33	44.24	52.59	56.5	61.68	53.38	41.76	44.21	22.45	26.3	15.28	17.44	38.53	30.52	32.55	56.27	65.47	58.31	47.51	48.9	30.86
hsa-miR-378h	nmiR03362.1:1	Endogenous	5.62	3.98	9.47	12.44	8.09	8.48	9.83	5.48	4.32	4.91	7.86	4.38	3.32	4.36	7.71	2.91	3.18	6.25	13.55	6.48	6.33	6.35	9.08
hsa-miR-378i	nmiR03179.1:1	Endogenous	35.81	36.81	100.08	58.06	62.7	59.33	75.99	42.43	17.28	32.75	34.8	39.45	28.57	36.12	50.87	35.61	42.87	43.77	56.44	59.39	68.1	50.81	16.34
hsa-miR-379-5p	nmiR03363.1:1	Endogenous	16.15	20.89	28.4	26.27	32.36	25.43	12.52	24.64	18.72	21.29	8.98	5.64	10.63	13.7	10.02	12.35	35.73	32.83	29.35	18.36	26.92	17.15	11.8
hsa-miR-380-3p	nmiR03364.1:1	Endogenous	6.32	6.96	2.7	12.44	14.16	16.95	8.94	8.21	10.08	21.29	5.61	3.13	3.32	4.98	10.02	10.9	4.76	10.94	11.29	9.72	14.25	3.81	3.63
hsa-miR-381-3p	nmiR03365.1:1	Endogenous	8.43	5.97	6.76	4.15	14.16	16.95	3.58	6.84	2.88	14.74	6.73	3.13	1.99	3.74	4.62	5.81	8.73	1.56	13.55	5.4	12.67	3.18	7.26
hsa-miR-381-5p	nmiR03989.1:1	Endogenous	2.81	1.99	2.7	5.53	6.07	5.65	3.58	4.11	5.76	1.64	4.49	1	2.66	3.11	1	5.09	1	7.82	2.26	1.08	3.17	4.45	6.35
hsa-miR-382-3p	nmiR03966.1:1	Endogenous	1	1.99	4.06	1.38	10.11	2.83	8.94	5.48	4.32	4.91	1.12	2.5	1.33	1.25	6.17	1.45	4.76	6.25	2.26	5.4	4.75	3.81	8.17
hsa-miR-382-5p	nmiR03366.1:1	Endogenous	35.11	22.88	25.7	40.09	34.38	45.2	8.94	16.43	27.36	44.21	17.96	6.26	13.29	27.4	10.02	14.53	25.41	45.33	9.03	22.68	20.59	15.24	13.62
hsa-miR-383-5p	nmiR03367.1:1	Endogenous	8.43	17.91	6.76	6.91	2.02	14.13	14.3	17.79	20.16	11.46	10.1	8.77	13.95	6.85	10.02	7.99	16.67	21.88	18.06	9.72	12.67	7.62	14.52
hsa-miR-384	nmiR03368.1:1	Endogenous	4.92	4.97	9.47	11.06	10.11	5.65	13.41	19.16	17.28	9.83	6.73	6.26	3.99	6.23	6.17	15.99	4.76	9.38	4.52	6.48	17.42	6.99	8.17
hsa-miR-3916	nmiR03939.1:1	Endogenous	4.21	5.97	1.35	8.29	4.05	5.65	4.47	6.84	2.88	3.28	1.12	1.88	5.32	2.49	5.39	1.45	7.15	6.25	9.03	14.04	6.33	5.08	6.35
hsa-miR-3918	nmiR03940.1:1	Endogenous	17.56	22.88	9.47	16.59	20.23	11.3	20.56	28.74	21.6	19.65	23.57	11.9	15.95	14.32	13.1	22.53	15.88	21.88	24.84	10.8	19	14.61	13.62
hsa-miR-3928-3p	nmiR03896.1:1	Endogenous	8.43	6.96	8.11	6.91	16.18	16.95	6.26	19.16	5.76	16.38	7.86	6.26	5.98	8.1	6.94	5.09	14.29	10.94	9.03	8.64	19	10.16	7.26
hsa-miR-3934-5p	nmiR03897.1:1	Endogenous	5.62	5.97	4.06	4.15	16.18	14.13	5.36	9.58	5.76	13.1	11.22	3.76	2.66	6.23	3.85	2.91	3.18	9.38	2.26	10.8	15.84	4.45	5.45
hsa-miR-409-3p	nmiR03369.1:1	Endogenous	8.43	8.95	9.47	9.68	8.09	2.83	6.26	12.32	10.08	13.1	10.1	5.01	9.3	4.98	4.62	9.45	16.67	12.51	20.32	8.64	14.25	6.35	5.45
hsa-miR-409-5p	nmiR03370.1:1	Endogenous	2.81	4.97	6.76	5.53	6.07	5.65	3.58	1.37	4.32	8.19	4.49	3.13	3.99	1.87	2.31	2.91	3.97	10.94	6.77	4.32	3.17	2.54	6.35
hsa-miR-410-3p	nmiR03371.1:1	Endogenous	4.92	5.97	6.76	5.53	6.07	14.13	2.68	15.06	11.52	11.46	8.98	5.64	9.97	4.36	10.79	8.72	5.56	10.94	13.55	12.96	17.42	12.07	10.89
hsa-miR-411-5p	nmiR03373.1:1	Endogenous	9.13	5.97	10.82	9.68	16.18	14.13	7.15	8.21	10.08	11.46	5.61	3.76	6.64	6.85	13.1	5.09	17.47	15.63	11.29	8.64	7.92	7.62	9.08
hsa-miR-412-3p	nmiR03375.1:1	Endogenous	2.11	2.98	1.35	6.91	12.14	14.13	3.58	9.58	1.44	8.19	3.37	3.76	2.66	3.11	3.85	2.91	1.59	3.13	2.26	2.16	9.5	4.45	4.54
hsa-miR-421	nmiR03376.1:1	Endogenous	23.17	23.87	27.05	41.47	30.34	33.9	27.71	20.53	18.72	13.1	20.2	21.29	22.59	22.42	24.66	13.81	23.82	31.26	24.84	25.92	30.09	29.21	17.25
hsa-miR-422a	nmiR03190.1:1	Endogenous	1	1	1.35	1.38	2.02	2.83	1	1.37	1.44	1.64	1.12	1	1	1	1	1	1	1.56	2.26	1.08	1.58	1	1
hsa-miR-423-3p	nmiR03378.1:1	Endogenous	31.6	30.84	27.05	15.21	34.38	33.9	26.82	16.43	23.04	39.3	34.8	25.05	33.22	41.72	35.45	21.07	39.7	45.33	56.44	35.64	33.26	32.39	31.77
hsa-miR-423-5p	nmiR03380.1:1	Endogenous	72.33	52.72	81.15	33.18	30.34	93.23	27.71	26.01	83.51	88.43	96.53	87.04	89.03	86.56	43.93	31.97	57.16	42.21	83.54	48.59	60.18	41.28	102.57
hsa-miR-424-5p	nmiR03381.1:1	Endogenous	150.28	191	243.44	172.8	184.05	149.74	267.29	474.97	223.18	617.35	168.37	184.73	237.85	102.75	205	235.45	160.38	203.21	246.1	328.28	247.06	169.57	118
hsa-miR-425-5p	nmiR03382.1:1	Endogenous	48.45	38.8	55.45	56.68	44.5	16.95	43.8	50.64	27.36	37.66	38.16	35.69	41.86	39.86	55.49	60.32	45.26	46.89	36.12	62.63	52.26	56.52	50.83
hsa-miR-4284	nmiR03898.1:1	Endogenous	4.21	4.97	5.41	1.38	4.05	2.83	1	2.74	2.88	4.91	2.24	1.25	1.99	3.11	1	2.91	1.59	6.25	9.03	7.56	6.33	3.18	2.72
hsa-miR-4286	nmiR03899.1:1	Endogenous	14.04	7.96	9.47	11.06	10.11	8.48	7.15	13.69	5.76	8.19	13.47	10.65	13.95	9.34	10.02	13.08	15.88	12.51	22.58	17.28	14.25	11.43	14.52
hsa-miR-429	nmiR03383.1:1	Endogenous	2.81	4.97	4.06	5.53	10.11	8.48	8.05	13.69	4.32	4.91	7.86	4.38	4.65	2.49	7.71	10.17	3.97	6.25	6.77	3.24	14.25	4.45	4.54
hsa-miR-431-5p	nmiR03384.1:1	Endogenous	10.53	7.96	4.06	9.68	8.09	14.13	5.36	9.58	14.4	6.55	2.24	3.13	5.98	5.6	6.17	6.54	18.26	26.57	9.03	6.48	6.33	7.62	4.54
hsa-miR-432-5p	nmiR03385.1:1	Endogenous	9.13	11.94	6.76	5.53	16.18	11.3	7.15	8.21	14.4	6.55	6.73	5.64	7.31	4.98	5.39	3.63	10.32	18.76	9.03	10.8	3.17	5.08	4.54
hsa-miR-433-3p	nmiR03386.1:1	Endogenous	2.11	3.98	5.41	8.29	6.07	14.13	2.68	13.69	5.76	6.55	1.12	3.13	1	2.49	1.54	2.18	5.56	3.13	13.55	5.4	9.5	5.08	4.54
hsa-miR-433-5p	nmiR04009.1:1	Endogenous	4.21	7.96	12.17	1.38	14.16	19.78	4.47	8.21	4.32	19.65	14.59	5.01	9.97	4.98	7.71	5.81	4.76	10.94	6.77	7.56	7.92	12.7	9.98
hsa-miR-4421	nmiR03900.1:1	Endogenous	7.72	6.96	6.76	5.53	8.09	16.95	20.56	42.43	14.4	24.56	3.37	6.26	11.29	6.85	13.87	23.25	5.56	20.32	6.77	16.2	22.17	8.26	10.89
hsa-miR-4425	nmiR03901.1:1	Endogenous	3.51	5.97	2.7	8.29	14.16	22.6	9.83	6.84	2.88	3.28	5.61	6.26	6.64	3.74	5.39	5.81	10.32	10.94	6.77	15.12	7.92	6.35	9.98
hsa-miR-4431	nmiR03902.1:1	Endogenous	1	3.98	4.06	1.38	14.16	11.3	1	5.48	4.32	9.83	1.12	1.25	3.32	1	1	2.18	3.18	4.69	11.29	4.32	6.33	1.91	5.45
hsa-miR-4435	nmiR03903.1:1	Endogenous	2.81	2.98	2.7	5.53	4.05	2.83	3.58	9.58	5.76	6.55	3.37	1.88	3.99	3.11	3.08	4.36	5.56	4.69	13.55	6.48	9.5	3.81	4.54
hsa-miR-4443	nmiR03904.1:1	Endogenous	107.44	118.38	129.83	96.77	84.95	76.28	90.29	50.64	105.11	96.61	182.97	209.78	118.26	89.05	123.31	82.84	106.39	60.96	277.7	170.62	166.29	140.99	201.52
hsa-miR-4448	nmiR03905.1:1	Endogenous	1	9.95	4.06	1.38	4.05	2.83	1	8.21	4.32	4.91	1.12	2.5	2.66	2.49	3.85	2.91	7.15	3.13	13.55	3.24	3.17	3.81	6.35
hsa-miR-4451	nmiR03906.1:1	Endogenous	7.72	16.91	8.11	9.68	10.11	2.83	5.36	9.58	7.2	11.46	5.61	2.5	5.32	5.6	5.39	2.91	9.53	6.25	18.06	5.4	17.42	5.72	9.98
hsa-miR-4454+hsa-miR-7975	nmiR03907.1:1	Endogenous	1235.24	1107.18	1019.74	819.76	677.55	1180.94	853.72	744.62	910.02	1290.37	1421.08	1791.56	1987.18	1317.71	1330.2	695.45	897.16	1026.99	1571.4	986.99	1268.58	1316.57	1737.39
hsa-miR-4455	nmiR03908.1:1	Endogenous	8.43	8.95	5.41	9.68	14.16	14.13	8.94	8.21	7.2	9.83	15.71	8.77	9.97	9.34	6.94	5.09	7.15	6.25	22.58	7.56	6.33	7.62	11.8
hsa-miR-4458	nmiR03909.1:1	Endogenous	3.51	2.98	1.35	1.38	6.07	2.83	3.58	6.84	2.88	3.28	4.49	1	2.66	1.87	1.54	3.63	6.35	4.69	13.55	3.24	9.5	1.91	3.63
hsa-miR-4461	nmiR03910.1:1	Endogenous	4.92	1	5.41	1.38	4.05	8.48	4.47	6.84	4.32	13.1	3.37	3.13	2.66	4.98	3.85	5.81	3.18	6.25	13.55	8.64	11.09	3.81	1.82

hsa-miR-448	nmiR03387.1:1	Endogenous	3.51	7.96	4.06	2.76	2.02	2.83	3.58	1.37	4.32	3.28	3.37	2.5	1	1.25	1.54	3.63	3.18	3.13	2.26	2.16	12.67	3.81	4.54
hsa-miR-4485-3p	nmiR03170.1:1	Endogenous	1	3.98	1.35	4.15	14.16	5.65	3.58	8.21	4.32	6.55	4.49	3.76	1.33	1.87	5.39	5.81	4.76	12.51	9.03	2.16	14.25	6.99	5.45
hsa-miR-4488	nmiR03269.1:1	Endogenous	3.51	9.95	2.7	2.76	8.09	11.3	5.36	5.48	11.52	11.46	5.61	11.9	9.97	8.1	4.62	4.36	5.56	6.25	20.32	5.4	12.67	6.35	8.17
hsa-miR-449a	nmiR03388.1:1	Endogenous	1	2.98	1.35	1.38	6.07	2.83	5.36	1.37	1.44	3.28	1.12	3.13	1	1.25	2.31	2.91	4.76	6.25	11.29	3.24	3.17	1.91	1.82
hsa-miR-449b-5p	nmiR03389.1:1	Endogenous	1.4	5.97	1.35	1.38	14.16	8.48	9.83	10.95	4.32	6.55	6.73	1.88	3.32	1.25	5.39	6.54	3.18	7.82	18.06	5.4	12.67	5.08	9.98
hsa-miR-449c-5p	nmiR03390.1:1	Endogenous	1	6.96	2.7	2.76	2.02	2.83	6.26	9.58	11.52	9.83	6.73	4.38	3.32	1.87	3.08	4.36	3.18	15.63	13.55	11.88	12.67	4.45	4.54
hsa-miR-450a-1-3p	nmiR03968.1:1	Endogenous	6.32	7.96	6.76	5.53	2.02	8.48	8.94	15.06	5.76	18.01	4.49	1.88	11.29	5.6	9.25	7.99	5.56	10.94	18.06	5.4	12.67	5.72	10.89
hsa-miR-450a-2-3p	nmiR04029.1:1	Endogenous	2.81	4.97	4.06	2.76	4.05	19.78	5.36	2.74	7.2	1.64	13.47	5.64	4.65	7.47	3.85	2.91	3.18	4.69	11.29	1.08	6.33	6.99	4.54
hsa-miR-450a-5p	nmiR03391.1:1	Endogenous	97.61	82.57	120.37	77.41	72.81	76.28	92.97	105.4	84.95	219.43	121.23	124.61	225.89	74.11	97.11	91.56	78.6	75.03	103.86	136.06	102.94	109.24	92.59
hsa-miR-450b-3p	nmiR03392.1:1	Endogenous	4.92	6.96	6.76	4.15	8.09	33.9	8.05	12.32	5.76	4.91	3.37	2.5	5.32	5.6	7.71	2.91	7.15	10.94	18.06	5.4	14.25	6.35	5.45
hsa-miR-450b-5p	nmiR03393.1:1	Endogenous	8.43	4.97	10.82	9.68	12.14	19.78	10.73	10.95	17.28	18.01	6.73	8.77	7.31	6.85	4.62	8.72	6.35	7.82	9.03	10.8	9.5	5.72	9.98
hsa-miR-4516	nmiR03357.1:1	Endogenous	31.6	13.93	10.82	17.97	2.02	16.95	12.52	15.06	46.08	22.93	29.18	64.5	65.11	46.71	28.52	17.44	18.26	29.7	29.35	30.24	28.51	28.58	81.7
hsa-miR-451a	nmiR03394.1:1	Endogenous	27822	16242.6	17250.4	35509.8	16685.9	58778.5	181393	294694	95620.9	54789.9	21839.3	29824.7	61971.3	17076.1	68566.1	169405	23206.4	51764	33791.8	40533.6	98656.1	21389	82111
hsa-miR-452-5p	nmiR03395.1:1	Endogenous	1	1	1.35	5.53	4.05	5.65	4.47	8.21	2.88	4.91	5.61	2.5	2.66	3.74	3.08	3.63	1	4.69	11.29	1.08	7.92	3.18	4.54
hsa-miR-4521	nmiR03488.1:1	Endogenous	2.11	2.98	2.7	1.38	8.09	14.13	6.26	15.06	2.88	4.91	5.61	1.88	3.99	1.87	3.08	2.18	3.97	6.25	4.52	2.16	4.75	3.81	7.26
hsa-miR-4524a-5p	nmiR03942.1:1	Endogenous	2.11	4.97	1.35	4.15	4.05	8.48	1.79	5.48	1.44	4.91	5.61	1.88	1	1.25	3.08	1.45	5.56	7.82	6.77	1.08	3.17	2.54	1.82
hsa-miR-4531	nmiR03851.1:1	Endogenous	2.11	3.98	1.35	6.91	2.02	5.65	1.79	5.48	2.88	6.55	6.73	1.25	5.32	4.36	6.17	2.18	4.76	6.25	6.77	7.56	9.5	3.81	5.45
hsa-miR-4532	nmiR03888.1:1	Endogenous	2.81	7.96	1.35	4.15	10.11	2.83	2.68	8.21	1.44	4.91	1.12	1.25	2.66	3.11	3.08	5.81	4.76	3.13	2.26	5.4	4.75	4.45	2.72
hsa-miR-4536-3p	nmiR03952.1:1	Endogenous	3.51	1.99	2.7	1.38	4.05	8.48	5.36	6.84	2.88	9.83	3.37	1.25	1.99	1.87	4.62	3.63	5.56	6.25	2.26	4.32	3.17	4.45	4.54
hsa-miR-4536-5p	nmiR03943.1:1	Endogenous	9.13	12.93	6.76	8.29	4.05	19.78	3.58	10.95	1.44	8.19	3.37	4.38	3.32	7.47	10.02	5.81	4.76	14.07	18.06	5.4	15.84	6.35	9.08
hsa-miR-454-3p	nmiR03397.1:1	Endogenous	41.43	44.76	45.98	38.71	32.36	8.48	33.97	31.48	50.4	37.66	55	48.22	67.77	51.06	47.78	46.51	42.08	45.33	22.58	51.83	49.1	41.92	78.06
hsa-miR-455-3p	nmiR03398.1:1	Endogenous	1.4	1	1.35	1.38	6.07	5.65	2.68	5.48	2.88	1.64	1.12	2.5	1	1.87	2.31	1	3.18	1.56	9.03	4.32	1.58	1.27	1
hsa-miR-455-5p	nmiR03400.1:1	Endogenous	2.81	3.98	4.06	2.76	8.09	11.3	3.58	8.21	4.32	8.19	4.49	3.76	3.99	2.49	3.08	4.36	3.97	4.69	15.8	2.16	3.17	8.26	2.72
hsa-miR-4647	nmiR03578.1:1	Endogenous	2.81	2.98	1.35	1.38	14.16	2.83	6.26	6.84	4.32	6.55	6.73	3.13	1.33	1.25	3.85	1.45	6.35	3.13	11.29	7.56	11.09	1	5.45
hsa-miR-4707-3p	nmiR03947.1:1	Endogenous	4.21	7.96	10.82	4.15	6.07	16.95	8.94	2.74	2.88	6.55	3.37	3.76	5.98	5.6	3.08	6.54	3.97	10.94	15.8	14.04	15.84	5.08	5.45
hsa-miR-4707-5p	nmiR03946.1:1	Endogenous	7.02	4.97	8.11	6.91	14.16	8.48	6.26	9.58	5.76	11.46	8.98	3.13	6.64	8.1	4.62	5.09	2.38	12.51	22.58	8.64	15.84	10.8	7.26
hsa-miR-4741	nmiR03642.1:1	Endogenous	6.32	4.97	4.06	6.91	6.07	11.3	1.79	12.32	2.88	8.19	3.37	2.5	5.98	6.23	5.39	4.36	6.35	7.82	6.77	7.56	15.84	3.81	7.26
hsa-miR-4755-5p	nmiR03948.1:1	Endogenous	7.72	7.96	6.76	2.76	16.18	8.48	4.47	6.84	7.2	8.19	3.37	4.38	4.65	1.87	7.71	4.36	7.15	12.51	4.52	6.48	6.33	3.81	9.98
hsa-miR-4787-3p	nmiR03950.1:1	Endogenous	1	1	1.35	1.38	4.05	2.83	1	1.37	1.44	1.64	1.12	1	1	1	1	1	1	1.56	2.26	1.08	1.58	1	1
hsa-miR-4787-5p	nmiR03949.1:1	Endogenous	3.51	9.95	8.11	2.76	14.16	14.13	4.47	10.95	11.52	16.38	8.98	6.26	5.32	6.23	6.17	6.54	5.56	10.94	9.03	11.88	11.09	5.08	6.35
hsa-miR-4792	nmiR03668.1:1	Endogenous	1.4	1	2.7	1.38	6.07	8.48	6.26	6.84	2.88	3.28	4.49	1.25	3.32	1	2.31	2.18	2.38	7.82	4.52	1.08	12.67	4.45	2.72
hsa-miR-483-3p	nmiR03401.1:1	Endogenous	8.43	9.95	4.06	6.91	18.2	14.13	7.15	5.48	7.2	8.19	4.49	11.9	11.96	13.08	10.79	6.54	7.15	6.25	9.03	5.4	19	9.53	9.08
hsa-miR-483-5p	nmiR03402.1:1	Endogenous	3.51	3.98	4.06	1.38	4.05	14.13	7.15	8.21	4.32	3.28	2.24	7.51	7.31	4.36	3.08	2.18	6.35	7.82	2.26	2.16	14.25	3.18	6.35
hsa-miR-484	nmiR03403.1:1	Endogenous	8.43	8.95	12.17	8.29	14.16	14.13	8.94	10.95	8.64	16.38	12.35	12.52	21.26	13.7	13.1	14.53	13.5	17.19	22.58	16.2	14.25	13.97	11.8
hsa-miR-485-3p	nmiR03404.1:1	Endogenous	35.11	18.9	39.22	35.94	24.27	36.73	21.45	19.16	38.88	37.66	20.2	23.8	63.78	30.51	30.06	24.71	33.35	57.84	31.61	33.48	25.34	26.04	24.51
hsa-miR-485-5p	nmiR03405.1:1	Endogenous	2.11	1.99	1.35	4.15	12.14	5.65	6.26	4.11	1.44	11.46	3.37	1.88	1.33	6.23	2.31	2.18	1	4.69	4.52	1.08	7.92	1.91	1.82
hsa-miR-486-3p	nmiR03406.1:1	Endogenous	21.77	23.87	13.52	22.12	38.43	31.08	35.76	58.86	34.56	18.01	17.96	7.51	15.95	17.44	22.35	38.51	22.23	18.76	42.9	17.28	20.59	26.04	19.97
hsa-miR-487a-3p	nmiR03407.1:1	Endogenous	7.02	4.97	5.41	11.06	12.14	14.13	4.47	4.11	5.76	13.1	6.73	6.89	4.65	5.6	5.39	5.09	11.91	10.94	9.03	5.4	7.92	3.81	8.17
hsa-miR-487b-3p	nmiR03408.1:1	Endogenous	7.72	11.94	6.76	6.91	4.05	8.48	5.36	8.21	8.64	11.46	4.49	2.5	3.99	5.6	4.62	9.45	12.7	15.63	2.26	9.72	11.09	5.72	5.45
hsa-miR-487b-5p	nmiR04015.1:1	Endogenous	3.51	2.98	2.7	4.15	6.07	16.95	1.79	5.48	2.88	8.19	6.73	2.5	1.33	4.36	3.85	2.18	3.97	4.69	4.52	4.32	7.92	5.08	5.45
hsa-miR-488-3p	nmiR03409.1:1	Endogenous	7.72	16.91	5.41	8.29	10.11	16.95	8.05	15.06	10.08	8.19	6.73	8.14	6.64	4.36	8.48	11.63	11.12	14.07	13.55	7.56	6.33	15.24	4.54
hsa-miR-489-3p	nmiR03410.1:1	Endogenous	2.81	7.96	5.41	1.38	8.09	11.3	4.47	12.32	8.64	9.83	7.86	1.25	4.65	3.11	4.62	2.91	5.56	12.51	9.03	4.32	11.09	4.45	1.82
hsa-miR-490-3p	nmiR03412.1:1	Endogenous	2.11	2.98	4.06	2.76	8.09	2.83	7.15	9.58	4.32	8.19	4.49	3.13	3.32	1.87	6.17	4.36	8.73	6.25	11.29	5.4	15.84	5.72	9.98
hsa-miR-490-5p	nmiR03413.1:1	Endogenous	4.92	2.98	1.35	2.76	8.09	8.48	5.36	4.11	4.32	6.55	2.24	1.88	1.33	1	6.94	5.81	5.56	4.69	6.77	1.08	11.09	4.45	4.54
hsa-miR-491-3p	nmiR03414.1:1	Endogenous	3.51	4.97	5.41	16.59	10.11	8.48	8.05	20.53	10.08	8.19	7.86	3.76	5.98	1	5.39	10.9	4.76	9.38	15.8	5.4	11.09	5.08	8.17
hsa-miR-491-5p	nmiR03415.1:1	Endogenous	17.56	7.96	13.52	5.53	18.2	31.08	50.06	90.34	28.8	19.65	15.71	11.9	23.92	12.45	20.04	60.32	23.02	20.32	29.35	23.76	34.84	6.99	27.23
hsa-miR-492	nmiR03416.1:1	Endogenous	2.11	4.97	4.06	1.38	4.05	2.83	1	5.48	1.44	3.28	3.37	2.5	1	1.25	2.31	2.91	1	3.13	9.03	4.32	4.75	1.27	1
hsa-miR-493-3p	nmiR03417.1:1	Endogenous	6.32	7.96	2.7	4.15	10.11	19.78	8.94	5.48	2.88	8.19	1.12	5.01	5.32	3.74	6.17	9.45	5.56	7.82	13.55	12.96	7.92	7.62	7.26
hsa-miR-494-3p	nmiR03418.1:1	Endogenous	1	1	1.35	1.38	2.02	2.83	1	1.37	1.44	1.64	1.12	1	1	1	1	1	1	1.56	2.26	1.08	1.58	1	1
hsa-miR-494-5p	nmiR04011.1:1	Endogenous	1.4	1.99	2.7	1.38	6.07	2.83	2.68	4.11	2.88	4.91	5.61	1.25	2.66	2.49	2.31	1	2.38	12.51	6.77	7.56	4.75	2.54	2.72
hsa-miR-495-3p	nmiR03419.1:1	Endogenous	48.45	47.75	54.1	87.09	48.54	64.98	17.88	41.06	34.56	42.58	25.82	15.03	22.59	39.23	16.96	28.34	67.49	76.59	27.09	17.28	31.67	12.7	13.62
hsa-miR-495-5p	nmiR03990.1:1	Endogenous	2.81	7.96	5.41	1.38	6.07	2.83	2.68	6.84	5.76	8.19	4.49	1.25	1.99	3.11	2.31	3.63	3.18	10.94	4.52	3.24	11.09	2.54	1.82
hsa-miR-496	nmiR03420.1:1	Endogenous	9.83	2.98	8.11	2.76	8.09	2.83	3.58	4.11	4.32	9.83	3.37	3.76	9.3	6.23	6.94	7.99	9.53	4.69	18.06	9.72	7.92	6.35	9.98
hsa-miR-497-5p	nmiR03421.1:1	Endogenous	5.62	4.97	2.7	4.15	2.02	11.3	10.73	10.95	11.52	11.46	6.73	1.25	3.99	3.74	3.85	5.81	7.94	18.76	18.06	6.48	9.5	4.45	5.45
hsa-miR-498	nmiR03422.1:1	Endogenous	1.4	3.98	2.7	4.15	4.05	2.83	3.58	4.11	4.32	6.55	1.12	3.76	3.32	1.87	3.08	2.91	2.38	3.13	4.52	4.32	4.75	2.54	6.35
hsa-miR-499a-3p	nmiR03426.1:1	Endogenous	4.92	9.95	10.82	4.15	10.11	2.83	8.94	13.69	12.96	3.28	4.49	6.26	5.98	4.98	3.85	3.63	9.53	7.82	11.29	4.32	22.17	6.35	9.08
hsa-miR-499a-5p	nmiR03428.1:1	Endogenous	6.32	4.97	8.11	9.68	6.07	8.48	8.94	5.48	8.64	13.1	12.35	4.38	6.64	8.1	3.85	7.27	5.56	4.69	18.06	5.4	30.09	8.26	9.98
hsa-miR-499b-3p	nmiR03424.1:1	Endogenous	6.32	3.98	4.06	5.53	6.07	8.48	5.36	5.48	7.2	9.83	5.61	6.26	3.32	2.49	6.94	9.45	3.18	6.25	9.03	9.72	11.09	4.45	3.63
hsa-miR-499b-5p	nmiR03425.1:1	Endogenous	6.32	5.97	9.47	5.53	10.11	5.65	8.94	12.32	20.16	3.28	8.98	4.38	9.97	4.36	4.62	9.45	3.18	14.07	24.84	11.88	9.5	6.99	10.89
hsa-miR-5001-3p	nmiR03954.1:1	Endogenous	9.83	4.97	1.35	1.38	2.02	5.65	14.3	13.69	10.08	6.55	10.1	6.89	15.95	6.23	10.79	7.27	6.35	6.25	6.77	10.8	12.67	3.81	13.62
hsa-miR-5001-5p	nmiR03953.1:1	Endogenous	2.81	1.99	5.41	1.38	6.07	2.83	2.68	4.11	2.88	3.28	3.37	3.13	1.99	1.87	2.31	2.91	3.18	4.69	11.29	7.56	4.75	3.18	2.72
hsa-miR-500a-5p+hsa-miR-501-5p	nmiR03429.1:1	Endogenous	14.04	9.95	28.4	16.59	10.11	14.13	11.62	15.06	4.32	14.74	12.35	6.89	7.97	7.47	11.56	14.53	9.53	20.32	11.29	17.28	12.67	13.34	9.98
hsa-miR-501-3p	nmiR03431.1:1	Endogenous	2.11	1.99	4.06	5.53	6.07	5.65	5.36	5.48	5.76	4.91	2.24	2.5	1.99	1.87	5.39	1.45	2.38	1.56	9.03	3.24	3.17	1.27	7.26
hsa-miR-5010-3p	nmiR03956.1:1	Endogenous	2.81	2.98	5.41	1.38	8.09	5.65	2.68	4.11	2.88	4.91	3.37	1	1.33	1.25	4.62	1.45	2.38	1.56	2.26	4.32	3.17	4.45	3.63
hsa-miR-5010-5p	nmiR03955.1:1	Endogenous	1.4	2.98	2.7	1.38	6.07	5.65	5.36	1.37	2.88	1.64	3.37	3.76	5.98	2.49	3.08	3.63	3.18	6.25	4.52	1.08	4.75	1.91	3.63
hsa-miR-502-3p	nmiR03432.1:1	Endogenous	3.51	1.99	12.17	5.53	8.09	11.3	4.47	13.69	7.2	6.55	7.86	6.26	5.98	4.36	4.62	5.09	8.73	10.94	2.26	19.44	11.09	6.99	11.8
hsa-miR-502-5p	nmiR03434.1:1	Endogenous	1	1	1.35	1.38	2.02	2.83	1	1.37	1.44	1.64	1.12	1	1	1	1	1	1	1.56	2.26	1.08	1.58	1	1
hsa-miR-503-3p	nmiR03991.1:1	Endogenous	3.51	6.96	5.41	1.38	8.09	2.83	3.58	6.84	5.76	3.28	3.37	1.25	5.32	5.6	5.39	6.54	2.38	1.56	11.29	5.4	9.5	3.18	5.45
hsa-miR-503-5p	nmiR03435.1:1	Endogenous	27.39	34.82	16.23	13.82	32.36	36.73	22.35	31.48	31.68	85.15	31.43	20.04	19.27	23.66	27.74	16.71	30.17	21.88	15.8	28.08	28.51	22.23	14.52
hsa-miR-504-3p	nmiR04013.1:1	Endogenous	1.4	2.98	2.7	1.38	2.02	2.83	3.58	4.11	1.44	1.64	4.49	3.13	1	1.25	3.85	5.09	7.15	6.25	9.03	6.48	7.92	1.91	1.82
hsa-miR-504-5p	nmiR03437.1:1	Endogenous	6.32	3.98	1.35	1.38	8.09	2.83	2.68	6.84	4.32	8.19	3.37	5.64	3.32	3.11	4.62	5.81	5.56	6.25	11.29	2.16	7.92	3.18	5.45
hsa-miR-505-3p	nmiR03438.1:1	Endogenous	27.39	29.84	24.34	23.5	30.34	25.43	22.35	36.96	21.6	27.84	25.82	26.3	39.86	26.15	31.6	34.15	24.61	21.88	36.12	29.16	33.26	17.15	19.97
hsa-miR-506-3p	nmiR03439.1:1	Endogenous	13.34	8.95	9.47	11.06	16.18	31.08	44.7	87.6	25.92	22.93	12.35	10.65	23.92	11.83	22.35	69.04	19.85	34.39	20.32	20.52	41.18	15.24	31.77
hsa-miR-506-5p	nmiR03969.1:1	Endogenous	4.92	3.98	2.7	1.38	12.14	8.48	9.83	12.32	4.32	13.1	2.24	6.26	3.99	1.87	3.08	8.72	6.35	14.07	9.03	7.56	14.25	1.91	12.71
hsa-miR-507	nmiR03440.1:1	Endogenous	3.51	6.96	1.35	5.53	4.05	2.83	5.36	5.48	2.88	9.83	4.49	3.76	3.32	4.36	8.48	5.81	2.38	1.56	4.52	12.96	9.5	4.45	4.54
hsa-miR-508-3p	nmiR03441.1:1	Endogenous	1	6.96	1.35	1.38	4.05	11.3	2.68	4.11	5.76	8.19	7.86	5.64	1	3.74	6.17	6.54	1.59	6.25	18.06	11.88	9.5	4.45	5.45
hsa-miR-508-5p	nmiR03442.1:1	Endogenous	2.81	3.98	6.76	2.76	6.07	2.83	1	4.11	1.44	3.28	7.86	1.88	3.32	1.25	4.62	2.18	3.97	14.07	11.29	3.24	6.33	1.91	1.82
hsa-miR-509-3-5p	nmiR03443.1:1	Endogenous	3.51	3.98	1.35	5.53	12.14	8.48	6.26	8.21	7.2	8.19	6.73	4.38	3.99	4.36	5.39	2.18	4.76	9.38	4.52	6.48	22.17	3.81	3.63
hsa-miR-509-3p	nmiR03444.1:1	Endogenous	6.32	16.91	5.41	8.29	8.09	11.3	13.41	12.32	7.2	13.1	19.08	10.65	6.64	8.1	11.56	6.54	7.15	18.76	15.8	10.8	19	15.24	12.71
hsa-miR-509-5p	nmiR03445.1:1	Endogenous	9.13	11.94	4.06	13.82	16.18	5.65	7.15	6.84	14.4	14.74	11.22	5.01	2.66	3.11	8.48	10.17	3.97	15.63	20.32	8.64	15.84	6.35	9.08
hsa-miR-510-3p	nmiR04014.1:1	Endogenous	3.51	2.98	2.7	1.38	4.05	2.83	4.47	9.58	4.32	3.28	5.61	3.13	1.33	1	6.94	1	3.18	6.25	9.03	6.48	11.09	5.08	7.26
hsa-miR-510-5p	nmiR03446.1:1	Endogenous	1.4	3.98	1.35	2.76	2.02	11.3	1.79	5.48	2.88	1.64	2.24	1.25	3.32	1.25	3.08	1.45	1.59	14.07	4.52	4.32	9.5	1.27	4.54
hsa-miR-511-5p	nmiR03447.1:1	Endogenous	4.21	5.97	1.35	4.15	6.07	2.83	4.47	6.84	5.76	6.55	6.73	3.13	1.99	4.98	5.39	4.36	3.18	10.94	6.77	4.32	12.67	3.18	7.26
hsa-miR-512-3p	nmiR03448.1:1	Endogenous	2.11	5.97	1.35	1.38	10.11	8.48	2.68	6.84	2.88	4.91	3.37	4.38	3.32	1.87	2.31	3.63	3.18	7.82	2.26	4.32	6.33	2.54	3.63
hsa-miR-512-5p	nmiR03449.1:1	Endogenous	2.11	3.98	5.41	5.53	6.07	2.83	2.68	8.21	1.44	3.28	3.37	3.13	3.32	2.49	1.54	3.63	2.38	9.38	13.55	2.16	9.5	3.18	1
hsa-miR-513a-3p	nmiR03450.1:1	Endogenous	1.4	6.96	4.06	6.91	2.02	14.13	1.79	1.37	2.88	3.28	2.24	1.88	2.66	1.87	6.17	2.91	3.18	10.94	9.03	3.24	4.75	1.27	3.63
hsa-miR-513a-5p	nmiR03451.1:1	Endogenous	4.21	7.96	6.76	5.53	10.11	19.78	1.79	9.58	5.76	11.46	3.37	4.38	3.99	4.98	4.62	6.54	3.97	6.25	15.8	7.56	9.5	4.45	1.82
hsa-miR-513b-5p	nmiR03453.1:1	Endogenous	11.94	19.9	5.41	12.44	12.14	28.25	43.8	105.4	34.56	29.48	15.71	13.15	27.24	14.95	34.68	53.05	11.12	21.88	13.55	16.2	41.18	7.62	35.4
hsa-miR-513c-3p	nmiR03984.1:1	Endogenous	8.43	4.97	4.06	5.53	2.02	16.95	5.36	5.48	2.88	9.83	2.24	3.13	5.32	6.23	4.62	10.9	7.15	12.51	11.29	10.8	11.09	7.62	4.54
hsa-miR-513c-5p	nmiR03454.1:1	Endogenous	3.51	6.96	5.41	4.15	8.09	5.65	2.68	6.84	10.08	4.91	3.37	2.5	1.99	3.74	5.39	2.18	4.76	9.38	4.52	7.56	14.25	4.45	6.35
hsa-miR-514a-3p	nmiR03456.1:1	Endogenous	3.51	8.95	5.41	8.29	12.14	11.3	3.58	13.69	15.84	14.74	11.22	6.26	5.32	8.72	7.71	7.27	3.97	10.94	13.55	14.04	20.59	9.53	8.17
hsa-miR-514a-5p	nmiR03970.1:1	Endogenous	7.72	8.95	9.47	13.82	18.2	22.6	59	114.98	31.68	29.48	12.35	8.14	35.21	11.21	23.12	69.76	18.26	20.32	24.84	17.28	41.18	12.7	29.95
hsa-miR-514b-3p	nmiR03455.1:1	Endogenous	4.92	1	1.35	4.15	4.05	5.65	3.58	8.21	8.64	8.19	4.49	3.13	1.33	5.6	5.39	5.09	4.76	6.25	22.58	2.16	9.5	3.18	7.26
hsa-miR-514b-5p	nmiR03220.1:1	Endogenous	2.11	11.94	9.47	5.53	12.14	16.95	6.26	5.48	5.76	3.28	1.12	4.38	1.99	3.11	5.39	4.36	3.18	6.25	11.29	9.72	9.5	8.26	3.63
hsa-miR-515-3p	nmiR03457.1:1	Endogenous	5.62	5.97	5.41	1.38	12.14	5.65	5.36	5.48	8.64	8.19	10.1	1.88	5.32	3.74	6.94	3.63	1.59	9.38	6.77	4.32	1.58	5.72	7.26
hsa-miR-515-5p	nmiR03458.1:1	Endogenous	2.11	5.97	4.06	1.38	10.11	2.83	3.58	2.74	7.2	3.28	3.37	3.13	4.65	4.98	1.54	8.72	3.97	10.94	15.8	4.32	7.92	3.18	9.98
hsa-miR-516a-3p+hsa-miR-516b-3p	nmiR03341.1:1	Endogenous	8.43	5.97	4.06	9.68	14.16	16.95	4.47	4.11	5.76	11.46	11.22	2.5	5.32	3.74	7.71	5.81	6.35	6.25	6.77	6.48	9.5	8.26	7.26
hsa-miR-516a-5p	nmiR03459.1:1	Endogenous	2.81	6.96	2.7	2.76	8.09	5.65	4.47	4.11	7.2	6.55	6.73	1	3.99	3.74	4.62	2.91	2.38	6.25	6.77	4.32	3.17	4.45	3.63
hsa-miR-516b-5p	nmiR03460.1:1	Endogenous	4.92	4.97	6.76	5.53	8.09	5.65	9.83	17.79	12.96	8.19	7.86	5.64	8.64	4.98	6.17	11.63	5.56	14.07	11.29	7.56	14.25	9.53	12.71
hsa-miR-517a-3p	nmiR03461.1:1	Endogenous	4.92	3.98	4.06	4.15	10.11	14.13	5.36	10.95	10.08	11.46	7.86	1.88	7.31	3.11	6.17	5.09	5.56	21.88	27.09	5.4	15.84	3.81	12.71
hsa-miR-517b-3p	nmiR03462.1:1	Endogenous	5.62	13.93	6.76	1.38	8.09	11.3	9.83	9.58	10.08	3.28	2.24	5.64	9.97	4.98	8.48	5.81	5.56	9.38	15.8	6.48	6.33	4.45	5.45
hsa-miR-517c-3p+hsa-miR-519a-3p	nmiR03463.1:1	Endogenous	1.4	1.99	8.11	5.53	6.07	5.65	1.79	5.48	12.96	6.55	5.61	3.13	2.66	4.98	4.62	5.81	3.18	7.82	4.52	6.48	17.42	1	4.54
hsa-miR-518b	nmiR03465.1:1	Endogenous	28.09	24.87	17.58	16.59	34.38	22.6	16.99	12.32	20.16	22.93	23.57	20.66	11.29	23.04	11.56	21.8	24.61	28.14	11.29	35.64	17.42	20.32	16.34
hsa-miR-518c-3p	nmiR03466.1:1	Endogenous	5.62	2.98	2.7	2.76	2.02	8.48	3.58	5.48	5.76	4.91	7.86	2.5	3.32	3.11	3.08	6.54	1.59	3.13	2.26	5.4	4.75	5.72	9.08
hsa-miR-518d-3p	nmiR03467.1:1	Endogenous	4.21	1	5.41	5.53	4.05	2.83	12.52	9.58	2.88	6.55	1.12	1	3.32	1.87	3.08	4.36	1	3.13	22.58	8.64	7.92	4.45	3.63
hsa-miR-518e-3p	nmiR03468.1:1	Endogenous	1.4	3.98	4.06	1.38	2.02	2.83	1	2.74	1.44	4.91	1.12	1	1.33	1	3.85	4.36	6.35	4.69	11.29	1.08	1.58	2.54	5.45
hsa-miR-518f-3p	nmiR03469.1:1	Endogenous	5.62	14.92	4.06	6.91	10.11	14.13	7.15	10.95	5.76	14.74	2.24	3.13	7.97	5.6	9.25	7.27	7.94	3.13	18.06	11.88	12.67	4.45	6.35
hsa-miR-5196-3p+hsa-miR-6732-3p	nmiR03958.1:1	Endogenous	10.53	9.95	9.47	9.68	18.2	19.78	10.73	8.21	15.84	18.01	7.86	9.39	21.26	9.96	7.71	7.27	16.67	25.01	27.09	23.76	15.84	8.89	13.62
hsa-miR-5196-5p	nmiR03957.1:1	Endogenous	7.72	20.89	14.88	13.82	10.11	16.95	8.94	12.32	15.84	11.46	13.47	8.77	11.29	10.59	11.56	7.99	16.67	9.38	15.8	15.12	22.17	16.51	9.08
hsa-miR-519b-3p	nmiR03470.1:1	Endogenous	11.24	28.85	8.11	11.06	16.18	14.13	18.77	15.06	11.52	13.1	12.35	5.01	10.63	10.59	9.25	23.25	7.15	17.19	11.29	9.72	23.76	6.35	9.98
hsa-miR-519b-5p+hsa-miR-519c-5p	nmiR03471.1:1	Endogenous	2.11	2.98	2.7	1.38	6.07	2.83	4.47	4.11	8.64	1.64	3.37	3.13	1	3.74	6.17	2.18	4.76	7.82	9.03	7.56	6.33	3.18	4.54
hsa-miR-519c-3p	nmiR03472.1:1	Endogenous	5.62	5.97	1.35	4.15	2.02	2.83	3.58	8.21	7.2	6.55	5.61	1.25	1.99	4.98	5.39	5.09	2.38	1.56	6.77	5.4	12.67	3.18	7.26
hsa-miR-519d-3p	nmiR03473.1:1	Endogenous	5.62	9.95	2.7	6.91	8.09	28.25	4.47	8.21	10.08	8.19	8.98	3.13	1.33	4.98	4.62	2.18	3.18	7.82	11.29	14.04	12.67	7.62	8.17
hsa-miR-519e-3p	nmiR03474.1:1	Endogenous	3.51	2.98	4.06	5.53	2.02	11.3	7.15	9.58	7.2	6.55	6.73	2.5	5.98	1.87	4.62	1.45	2.38	4.69	4.52	6.48	4.75	3.81	2.72
hsa-miR-520a-3p	nmiR03475.1:1	Endogenous	1	5.97	1.35	1.38	4.05	8.48	3.58	2.74	2.88	4.91	1.12	1	3.32	1.87	3.08	2.18	3.18	3.13	11.29	4.32	11.09	2.54	4.54
hsa-miR-520a-5p	nmiR03476.1:1	Endogenous	7.72	6.96	8.11	6.91	16.18	25.43	8.05	17.79	14.4	13.1	14.59	8.14	5.98	7.47	8.48	10.9	8.73	12.51	31.61	8.64	11.09	8.89	7.26
hsa-miR-520b	nmiR03477.1:1	Endogenous	3.51	3.98	5.41	4.15	4.05	11.3	1	6.84	8.64	3.28	4.49	2.5	3.32	3.11	1.54	2.18	4.76	10.94	11.29	7.56	4.75	6.99	6.35
hsa-miR-520c-3p	nmiR03478.1:1	Endogenous	5.62	11.94	10.82	15.21	20.23	19.78	9.83	13.69	11.52	18.01	16.84	3.13	5.32	4.98	7.71	7.27	6.35	15.63	9.03	11.88	12.67	4.45	10.89
hsa-miR-520d-3p	nmiR03479.1:1	Endogenous	10.53	10.94	2.7	8.29	24.27	28.25	12.52	8.21	11.52	9.83	15.71	16.28	17.94	14.32	12.33	10.9	9.53	17.19	13.55	15.12	17.42	12.07	10.89
hsa-miR-520d-5p+hsa-miR-527+hsa	nmiR03480.1:1	Endogenous	33.71	55.71	29.75	17.97	54.61	53.68	38.44	16.43	31.68	31.11	43.78	26.93	35.21	29.89	23.89	29.07	26.99	20.32	67.73	28.08	34.84	31.76	28.14
hsa-miR-520e	nmiR03481.1:1	Endogenous	4.92	2.98	5.41	2.76	4.05	5.65	6.26	5.48	15.84	11.46	3.37	1	1	4.36	3.08	2.91	3.18	4.69	6.77	2.16	9.5	4.45	9.08
hsa-miR-520f-3p	nmiR03482.1:1	Endogenous	1.4	4.97	4.06	4.15	4.05	8.48	1	5.48	4.32	6.55	6.73	4.38	3.32	2.49	6.17	5.09	1.59	4.69	4.52	1.08	1.58	1.27	4.54
hsa-miR-520g-3p	nmiR03483.1:1	Endogenous	6.32	6.96	5.41	2.76	2.02	11.3	1	5.48	5.76	11.46	6.73	2.5	3.99	4.98	6.17	2.91	5.56	10.94	9.03	10.8	12.67	4.45	8.17
hsa-miR-520h	nmiR03484.1:1	Endogenous	4.21	5.97	4.06	4.15	12.14	14.13	4.47	4.11	2.88	3.28	3.37	3.13	3.32	3.74	5.39	5.09	4.76	9.38	6.77	8.64	12.67	2.54	3.63
hsa-miR-521	nmiR03485.1:1	Endogenous	1	1	1.35	1.38	2.02	2.83	1	1.37	1.44	1.64	1.12	1	1	1	1	1	1	9.38	4.52	10.8	1.58	1.91	3.63
hsa-miR-522-3p	nmiR03486.1:1	Endogenous	3.51	4.97	8.11	5.53	2.02	8.48	2.68	9.58	8.64	14.74	4.49	3.76	3.32	3.74	1	5.09	7.94	9.38	20.32	7.56	6.33	3.81	1.82
hsa-miR-523-3p	nmiR03487.1:1	Endogenous	2.81	5.97	10.82	2.76	8.09	28.25	13.41	15.06	11.52	6.55	4.49	6.89	5.98	4.98	8.48	18.89	7.15	9.38	13.55	12.96	6.33	5.08	5.45
hsa-miR-524-3p	nmiR03489.1:1	Endogenous	2.81	4.97	4.06	9.68	8.09	2.83	1.79	9.58	4.32	8.19	6.73	1.25	1.99	1	1	4.36	1.59	4.69	4.52	1.08	14.25	1.91	5.45
hsa-miR-525-3p	nmiR03491.1:1	Endogenous	3.51	9.95	1.35	4.15	4.05	11.3	8.05	20.53	1.44	13.1	1.12	3.13	3.99	4.98	3.85	7.99	4.76	14.07	13.55	9.72	4.75	1	9.08
hsa-miR-525-5p	nmiR03492.1:1	Endogenous	2.81	3.98	1.35	4.15	12.14	14.13	8.05	6.84	1.44	13.1	2.24	1.25	2.66	3.11	1	2.91	3.18	7.82	11.29	5.4	3.17	4.45	8.17
hsa-miR-526a+hsa-miR-518c-5p+hs	nmiR03493.1:1	Endogenous	16.85	10.94	10.82	4.15	18.2	31.08	16.99	28.74	21.6	19.65	19.08	12.52	14.62	11.21	12.33	33.43	18.26	28.14	20.32	19.44	20.59	7.62	31.77
hsa-miR-526b-5p	nmiR03494.1:1	Endogenous	2.11	5.97	2.7	6.91	6.07	2.83	3.58	1.37	11.52	4.91	3.37	1.88	3.32	3.11	3.08	3.63	3.18	1.56	9.03	2.16	4.75	3.18	4.54
hsa-miR-532-3p	nmiR03495.1:1	Endogenous	9.83	14.92	13.52	11.06	6.07	19.78	9.83	13.69	12.96	11.46	11.22	7.51	10.63	6.23	11.56	9.45	7.94	14.07	29.35	8.64	14.25	10.8	8.17
hsa-miR-532-5p	nmiR03496.1:1	Endogenous	9.83	6.96	16.23	4.15	20.23	8.48	8.94	20.53	2.88	4.91	14.59	6.89	9.97	11.21	10.79	8.72	5.56	3.13	11.29	11.88	11.09	8.26	10.89
hsa-miR-539-3p	nmiR03971.1:1	Endogenous	2.81	6.96	4.06	1.38	8.09	14.13	4.47	2.74	7.2	11.46	8.98	4.38	5.32	3.74	3.85	7.27	4.76	4.69	4.52	4.32	9.5	5.08	4.54
hsa-miR-539-5p	nmiR03497.1:1	Endogenous	4.21	5.97	4.06	4.15	4.05	8.48	5.36	6.84	5.76	3.28	4.49	5.64	3.99	3.11	4.62	6.54	4.76	12.51	6.77	5.4	9.5	5.08	10.89
hsa-miR-541-3p	nmiR03498.1:1	Endogenous	2.11	1	8.11	5.53	10.11	5.65	5.36	13.69	5.76	3.28	2.24	1	1	3.11	3.08	2.91	4.76	6.25	18.06	11.88	12.67	3.18	6.35
hsa-miR-542-3p	nmiR03499.1:1	Endogenous	1.4	1	6.76	1.38	4.05	22.6	1.79	9.58	2.88	9.83	1.12	1	1	1	1	4.36	7.15	6.25	24.84	8.64	7.92	8.26	7.26
hsa-miR-542-5p	nmiR03500.1:1	Endogenous	14.75	17.91	14.88	17.97	26.29	33.9	10.73	16.43	11.52	21.29	13.47	10.02	15.95	18.68	15.41	10.9	16.67	15.63	24.84	19.44	22.17	17.15	24.51
hsa-miR-543	nmiR03501.1:1	Endogenous	30.2	22.88	18.93	42.85	54.61	28.25	18.77	34.22	31.68	44.21	12.35	8.77	8.64	24.91	10.02	23.98	40.49	56.27	9.03	14.04	17.42	14.61	9.98
hsa-miR-544a	nmiR03372.1:1	Endogenous	3.51	1.99	4.06	1.38	10.11	11.3	1	4.11	4.32	3.28	4.49	3.76	3.99	3.11	3.08	3.63	3.97	7.82	2.26	4.32	4.75	3.18	3.63
hsa-miR-545-3p	nmiR03504.1:1	Endogenous	18.26	11.94	22.99	9.68	18.2	25.43	28.61	15.06	25.92	9.83	11.22	12.52	9.97	18.68	23.12	18.89	19.05	35.95	18.06	12.96	25.34	17.78	13.62
hsa-miR-548a-3p	nmiR03505.1:1	Endogenous	11.24	5.97	5.41	9.68	10.11	28.25	12.52	23.27	5.76	9.83	11.22	6.26	5.98	8.72	11.56	21.8	8.73	14.07	20.32	11.88	12.67	8.26	13.62
hsa-miR-548a-5p	nmiR03374.1:1	Endogenous	5.62	1.99	2.7	1.38	12.14	11.3	6.26	8.21	2.88	9.83	7.86	1.88	3.99	5.6	3.08	3.63	3.97	7.82	9.03	11.88	11.09	1.27	5.45
hsa-miR-548aa+hsa-miR-548t-3p	nmiR03377.1:1	Endogenous	1	1	1.35	1.38	2.02	2.83	1	1.37	1.44	1.64	1.12	1	1	1	1	1	1	1.56	2.26	1.08	1.58	1	1
hsa-miR-548ad-3p	nmiR03508.1:1	Endogenous	1	1	5.41	4.15	6.07	2.83	1	15.06	1.44	1.64	3.37	1	1	1.25	1.54	3.63	7.94	12.51	2.26	8.64	11.09	1.27	3.63
hsa-miR-548ah-5p	nmiR03511.1:1	Endogenous	5.62	5.97	12.17	8.29	6.07	36.73	7.15	16.43	14.4	16.38	6.73	5.01	3.99	8.1	8.48	5.81	8.73	18.76	22.58	7.56	17.42	5.08	12.71
hsa-miR-548ai+hsa-miR-570-5p	nmiR03379.1:1	Endogenous	3.51	7.96	4.06	4.15	12.14	8.48	6.26	6.84	4.32	11.46	11.22	5.01	5.98	1.25	9.25	5.81	5.56	7.82	11.29	5.4	11.09	4.45	9.98
hsa-miR-548ak	nmiR03513.1:1	Endogenous	3.51	10.94	5.41	9.68	10.11	8.48	8.05	6.84	10.08	13.1	7.86	3.76	5.98	7.47	4.62	2.91	7.15	3.13	15.8	11.88	6.33	4.45	8.17
hsa-miR-548al	nmiR03514.1:1	Endogenous	3.51	7.96	4.06	1.38	12.14	19.78	6.26	2.74	7.2	13.1	5.61	5.01	3.32	4.36	3.08	6.54	2.38	6.25	18.06	6.48	9.5	4.45	6.35
hsa-miR-548ar-3p	nmiR03960.1:1	Endogenous	4.21	3.98	1.35	2.76	14.16	16.95	2.68	6.84	7.2	18.01	4.49	3.13	1.33	2.49	5.39	3.63	1.59	6.25	9.03	3.24	11.09	5.08	5.45
hsa-miR-548ar-5p	nmiR03959.1:1	Endogenous	1	1	1.35	1.38	2.02	2.83	1	1.37	1.44	1.64	1.12	1	1	1	1	1	1	1.56	2.26	1.08	1.58	1	1

hsa-miR-548b-3p	nmiR03517.1:1	Endogenous	9.13	16.91	12.17	8.29	18.2	25.43	7.15	6.84	8.64	14.74	10.1	4.38	3.99	3.74	9.25	3.63	5.56	7.82	13.55	7.56	26.92	9.53	10.89
hsa-miR-548c-5p+hsa-miR-548o-5p	nmiR03520.1:1	Endogenous	1.4	5.97	9.47	2.76	2.02	5.65	1.79	8.21	5.76	4.91	4.49	1	5.32	7.47	6.17	9.45	2.38	9.38	13.55	5.4	11.09	2.54	4.54
hsa-miR-548d-3p	nmiR03521.1:1	Endogenous	7.02	7.96	5.41	8.29	6.07	16.95	9.83	15.06	12.96	6.55	7.86	8.14	8.64	6.23	6.17	7.27	7.15	10.94	13.55	10.8	9.5	6.35	5.45
hsa-miR-548d-5p	nmiR03522.1:1	Endogenous	19.66	21.88	17.58	20.74	34.38	25.43	29.5	30.11	18.72	24.56	22.45	25.05	25.91	19.93	22.35	39.97	30.17	31.26	51.93	27	39.59	26.04	31.77
hsa-miR-548e-3p	nmiR03523.1:1	Endogenous	3.51	4.97	1.35	5.53	4.05	2.83	5.36	6.84	7.2	4.91	3.37	3.13	1.99	1.25	5.39	5.81	4.76	6.25	13.55	4.32	7.92	3.18	4.54
hsa-miR-548e-5p	nmiR04024.1:1	Endogenous	3.51	6.96	5.41	2.76	18.2	22.6	2.68	4.11	11.52	4.91	10.1	1.88	2.66	3.11	10.79	5.81	9.53	18.76	15.8	9.72	11.09	5.08	11.8
hsa-miR-548g-3p	nmiR03525.1:1	Endogenous	2.11	5.97	2.7	8.29	4.05	19.78	5.36	8.21	4.32	11.46	6.73	5.64	2.66	1.87	1.54	5.81	4.76	9.38	13.55	8.64	19	7.62	9.08
hsa-miR-548h-5p	nmiR03526.1:1	Endogenous	1	1	1.35	2.76	2.02	2.83	2.68	1.37	1.44	8.19	2.24	1	1	1	1	1	5.56	7.82	11.29	1.08	6.33	1	2.72
hsa-miR-548i	nmiR03527.1:1	Endogenous	4.21	7.96	6.76	6.91	14.16	22.6	3.58	9.58	10.08	11.46	6.73	3.76	3.32	6.85	7.71	7.27	7.94	7.82	15.8	10.8	7.92	4.45	4.54
hsa-miR-548j-3p	nmiR04025.1:1	Endogenous	6.32	6.96	12.17	1.38	12.14	8.48	8.94	17.79	11.52	13.1	3.37	5.64	8.64	6.23	6.17	17.44	3.18	12.51	18.06	7.56	19	6.35	20.88
hsa-miR-548j-5p	nmiR03396.1:1	Endogenous	4.21	4.97	5.41	1.38	2.02	16.95	7.15	4.11	15.84	11.46	5.61	5.64	4.65	2.49	6.94	2.91	5.56	14.07	6.77	5.4	9.5	5.72	5.45
hsa-miR-548k	nmiR03528.1:1	Endogenous	1	4.97	10.82	4.15	6.07	8.48	1	8.21	1.44	4.91	1.12	3.13	1	1.87	1.54	2.91	6.35	10.94	2.26	3.24	6.33	6.35	4.54
hsa-miR-548l	nmiR03529.1:1	Endogenous	5.62	2.98	6.76	6.91	8.09	19.78	2.68	6.84	2.88	14.74	1.12	5.01	3.99	4.36	3.85	9.45	3.97	7.82	9.03	9.72	3.17	4.45	3.63
hsa-miR-548m	nmiR03530.1:1	Endogenous	3.51	4.97	6.76	2.76	6.07	14.13	8.94	6.84	4.32	9.83	3.37	3.76	8.64	4.36	4.62	5.81	5.56	10.94	20.32	6.48	4.75	6.35	7.26
hsa-miR-548n	nmiR03531.1:1	Endogenous	18.26	23.87	17.58	9.68	12.14	19.78	16.99	19.16	20.16	19.65	20.2	16.28	17.27	13.7	21.58	21.07	10.32	20.32	22.58	12.96	31.67	17.78	18.15
hsa-miR-548o-3p+hsa-miR-548ah-3	nmiR03532.1:1	Endogenous	1.4	1	2.7	4.15	4.05	2.83	1	4.11	5.76	1.64	2.24	1	1.99	3.11	1.54	1.45	1.59	1.56	2.26	1.08	3.17	1.27	3.63
hsa-miR-548q	nmiR03533.1:1	Endogenous	1.4	2.98	5.41	2.76	6.07	5.65	1	4.11	5.76	4.91	5.61	1.88	3.99	2.49	3.08	2.18	1	3.13	6.77	5.4	3.17	4.45	4.54
hsa-miR-548v	nmiR03537.1:1	Endogenous	6.32	6.96	2.7	4.15	12.14	14.13	3.58	9.58	4.32	4.91	7.86	7.51	3.99	4.98	6.94	5.81	4.76	9.38	24.84	16.2	14.25	3.81	5.45
hsa-miR-548y	nmiR03540.1:1	Endogenous	4.92	3.98	2.7	2.76	18.2	19.78	5.36	16.43	14.4	11.46	4.49	6.89	2.66	8.1	13.1	6.54	15.09	15.63	18.06	7.56	17.42	8.26	8.17
hsa-miR-548z+hsa-miR-548h-3p	nmiR03541.1:1	Endogenous	4.92	8.95	6.76	5.53	14.16	42.38	10.73	10.95	12.96	19.65	4.49	3.76	5.98	9.34	6.17	4.36	7.15	10.94	18.06	10.8	19	11.43	12.71
hsa-miR-549a	nmiR03411.1:1	Endogenous	4.21	7.96	8.11	6.91	8.09	11.3	7.15	4.11	7.2	11.46	7.86	5.64	3.32	4.98	6.17	6.54	7.15	12.51	24.84	5.4	11.09	5.08	6.35
hsa-miR-550a-5p	nmiR03542.1:1	Endogenous	1.4	2.98	4.06	2.76	6.07	11.3	4.47	1.37	2.88	4.91	4.49	1.88	2.66	1.25	3.85	2.18	3.18	3.13	11.29	1.08	3.17	1.91	3.63
hsa-miR-551a	nmiR03544.1:1	Endogenous	3.51	4.97	5.41	5.53	4.05	5.65	3.58	10.95	8.64	1.64	5.61	3.76	1.33	3.74	2.31	2.91	1.59	7.82	13.55	3.24	15.84	5.08	9.08
hsa-miR-551b-3p	nmiR03545.1:1	Endogenous	7.72	12.93	5.41	4.15	8.09	19.78	8.94	15.06	11.52	6.55	7.86	4.38	3.32	7.47	5.39	2.18	9.53	9.38	18.06	11.88	9.5	8.26	3.63
hsa-miR-552-3p	nmiR03546.1:1	Endogenous	2.11	4.97	2.7	2.76	8.09	8.48	2.68	10.95	2.88	6.55	2.24	1.88	3.32	3.74	4.62	7.27	3.97	17.19	6.77	4.32	9.5	3.81	4.54
hsa-miR-553	nmiR03547.1:1	Endogenous	3.51	3.98	5.41	2.76	6.07	5.65	4.47	12.32	5.76	11.46	1.12	3.13	4.65	3.74	2.31	5.09	1.59	3.13	11.29	5.4	14.25	6.99	4.54
hsa-miR-554	nmiR03548.1:1	Endogenous	4.21	1	5.41	4.15	10.11	2.83	4.47	8.21	4.32	8.19	2.24	3.76	3.32	2.49	4.62	2.91	4.76	4.69	6.77	8.64	1.58	6.35	4.54
hsa-miR-555	nmiR03549.1:1	Endogenous	9.83	8.95	8.11	12.44	8.09	36.73	4.47	8.21	7.2	11.46	11.22	6.89	9.97	8.1	12.33	7.27	10.32	12.51	13.55	15.12	9.5	8.89	11.8
hsa-miR-556-3p	nmiR03550.1:1	Endogenous	4.21	6.96	1.35	5.53	2.02	16.95	7.15	19.16	8.64	11.46	6.73	1.25	2.66	6.23	6.17	12.35	3.18	9.38	13.55	6.48	9.5	2.54	12.71
hsa-miR-556-5p	nmiR03551.1:1	Endogenous	2.81	3.98	5.41	4.15	2.02	2.83	5.36	8.21	8.64	3.28	4.49	1	6.64	1.25	5.39	6.54	5.56	15.63	4.52	3.24	3.17	3.81	3.63
hsa-miR-561-3p	nmiR03555.1:1	Endogenous	1.4	7.96	9.47	1.38	12.14	14.13	2.68	12.32	10.08	13.1	2.24	2.5	1.33	1.25	5.39	5.09	7.94	6.25	18.06	5.4	17.42	3.18	9.98
hsa-miR-561-5p	nmiR03972.1:1	Endogenous	5.62	9.95	9.47	4.15	10.11	25.43	5.36	5.48	4.32	4.91	6.73	2.5	2.66	6.23	6.17	5.09	3.97	1.56	11.29	11.88	9.5	6.99	4.54
hsa-miR-562	nmiR03556.1:1	Endogenous	4.92	1.99	5.41	4.15	4.05	2.83	1	2.74	5.76	4.91	5.61	1	1.33	1.87	6.17	4.36	1.59	3.13	11.29	5.4	9.5	3.81	1.82
hsa-miR-563	nmiR03557.1:1	Endogenous	1.4	1	5.41	1.38	10.11	11.3	3.58	8.21	5.76	8.19	5.61	5.01	5.32	1	2.31	2.91	3.18	7.82	20.32	7.56	6.33	6.99	2.72
hsa-miR-564	nmiR03558.1:1	Endogenous	9.13	6.96	14.88	15.21	14.16	14.13	10.73	16.43	15.84	21.29	14.59	13.15	13.29	6.85	13.87	11.63	11.91	21.88	24.84	16.2	28.51	10.8	18.15
hsa-miR-566	nmiR03559.1:1	Endogenous	1	1	1.35	1.38	6.07	16.95	1	4.11	4.32	6.55	1.12	1	1	1	1	1	4.76	10.94	4.52	5.4	7.92	5.08	4.54
hsa-miR-567	nmiR03560.1:1	Endogenous	4.21	5.97	4.06	1.38	14.16	11.3	3.58	5.48	7.2	1.64	4.49	3.76	3.99	4.98	9.25	6.54	4.76	6.25	4.52	7.56	14.25	7.62	8.17
hsa-miR-568	nmiR03561.1:1	Endogenous	2.81	4.97	4.06	4.15	10.11	14.13	11.62	27.38	7.2	9.83	6.73	1	6.64	1.87	7.71	15.26	8.73	6.25	15.8	7.56	14.25	4.45	12.71
hsa-miR-570-3p	nmiR03427.1:1	Endogenous	1	1	1.35	1.38	2.02	2.83	1	1.37	1.44	1.64	1.12	1	1	1	1	1	1	1.56	2.26	1.08	1.58	1	1
hsa-miR-571	nmiR03562.1:1	Endogenous	5.62	11.94	8.11	4.15	14.16	22.6	14.3	10.95	8.64	16.38	11.22	5.64	8.64	9.34	3.85	11.63	5.56	6.25	13.55	16.2	9.5	7.62	11.8
hsa-miR-572	nmiR03563.1:1	Endogenous	1	6.96	4.06	2.76	6.07	8.48	4.47	6.84	1.44	3.28	2.24	1.25	2.66	1.87	4.62	3.63	3.18	9.38	6.77	9.72	6.33	2.54	2.72
hsa-miR-573	nmiR03564.1:1	Endogenous	1	1	5.41	1.38	10.11	8.48	6.26	4.11	1.44	4.91	3.37	3.13	4.65	4.36	4.62	1.45	3.18	4.69	9.03	3.24	7.92	1	7.26
hsa-miR-574-3p	nmiR03565.1:1	Endogenous	17.56	8.95	17.58	16.59	26.29	25.43	8.05	9.58	12.96	18.01	14.59	13.15	15.28	14.95	19.27	9.45	15.09	20.32	29.35	17.28	6.33	17.78	13.62
hsa-miR-574-5p	nmiR03566.1:1	Endogenous	11.94	20.89	16.23	33.18	22.25	36.73	20.56	21.9	28.8	21.29	29.18	16.91	15.95	16.81	13.87	20.35	22.23	6.25	24.84	20.52	22.17	16.51	15.43
hsa-miR-575	nmiR03567.1:1	Endogenous	8.43	10.94	9.47	12.44	28.32	16.95	10.73	20.53	8.64	11.46	8.98	6.26	3.99	11.83	15.41	10.9	7.15	17.19	24.84	12.96	17.42	11.43	9.98
hsa-miR-576-3p	nmiR03568.1:1	Endogenous	2.81	4.97	4.06	2.76	4.05	8.48	3.58	8.21	8.64	6.55	3.37	3.76	3.99	3.11	1	2.18	3.97	6.25	9.03	8.64	3.17	5.08	2.72
hsa-miR-576-5p	nmiR03569.1:1	Endogenous	8.43	12.93	5.41	9.68	18.2	16.95	21.45	17.79	15.84	8.19	10.1	11.27	17.94	5.6	6.17	18.17	15.88	15.63	20.32	20.52	26.92	9.53	17.25
hsa-miR-577	nmiR03571.1:1	Endogenous	9.83	9.95	8.11	11.06	12.14	22.6	6.26	8.21	11.52	9.83	13.47	6.26	9.97	11.21	10.79	7.27	13.5	10.94	31.61	10.8	11.09	9.53	10.89
hsa-miR-578	nmiR03572.1:1	Endogenous	3.51	9.95	8.11	11.06	12.14	5.65	5.36	5.48	7.2	6.55	10.1	5.64	3.32	6.85	11.56	8.72	3.97	14.07	11.29	14.04	19	6.99	5.45
hsa-miR-579-3p	nmiR03573.1:1	Endogenous	1	1	1.35	1.38	2.02	2.83	1	1.37	1.44	1.64	1.12	1	1	1	1	1	1	1.56	2.26	1.08	1.58	1	1
hsa-miR-579-5p	nmiR04016.1:1	Endogenous	4.21	5.97	9.47	8.29	10.11	11.3	3.58	13.69	15.84	16.38	11.22	2.5	4.65	1.87	4.62	6.54	7.15	7.82	11.29	6.48	11.09	5.08	8.17
hsa-miR-580-3p	nmiR03574.1:1	Endogenous	2.81	2.98	6.76	6.91	10.11	11.3	1	2.74	4.32	6.55	8.98	2.5	3.32	3.74	4.62	4.36	5.56	4.69	9.03	6.48	7.92	6.35	5.45
hsa-miR-582-3p	nmiR03576.1:1	Endogenous	4.21	2.98	6.76	2.76	16.18	5.65	3.58	8.21	5.76	6.55	4.49	3.13	5.98	4.36	4.62	7.27	3.97	14.07	6.77	16.2	11.09	5.72	6.35
hsa-miR-582-5p	nmiR03577.1:1	Endogenous	135.53	55.71	66.27	35.94	52.59	33.9	97.44	72.55	50.4	70.41	53.88	73.89	136.2	52.93	59.34	124.99	55.58	76.59	92.57	64.79	74.44	43.82	65.36
hsa-miR-584-3p	nmiR03973.1:1	Endogenous	6.32	8.95	1.35	2.76	16.18	5.65	5.36	9.58	4.32	9.83	4.49	2.5	5.32	4.36	6.17	2.18	2.38	10.94	13.55	6.48	6.33	5.08	10.89
hsa-miR-584-5p	nmiR03580.1:1	Endogenous	2.11	5.97	5.41	2.76	6.07	14.13	5.36	16.43	11.52	8.19	4.49	3.13	2.66	3.74	3.85	6.54	4.76	6.25	9.03	5.4	9.5	6.35	2.72
hsa-miR-585-3p	nmiR03581.1:1	Endogenous	2.81	7.96	1.35	1.38	2.02	14.13	6.26	4.11	1.44	6.55	4.49	1.88	1	4.36	3.08	5.09	1	7.82	9.03	5.4	4.75	6.99	7.26
hsa-miR-587	nmiR03583.1:1	Endogenous	4.21	15.92	5.41	11.06	18.2	22.6	2.68	13.69	12.96	6.55	8.98	1.88	3.99	9.96	6.17	9.45	5.56	25.01	15.8	2.16	12.67	8.89	11.8
hsa-miR-589-5p	nmiR03585.1:1	Endogenous	2.11	11.94	4.06	2.76	4.05	11.3	3.58	1.37	5.76	1.64	5.61	2.5	3.32	4.98	6.94	4.36	3.97	6.25	15.8	4.32	7.92	5.08	9.08
hsa-miR-590-3p	nmiR03586.1:1	Endogenous	2.81	2.98	2.7	4.15	12.14	16.95	5.36	2.74	5.76	8.19	4.49	3.13	1.99	3.11	1.54	5.09	4.76	6.25	11.29	7.56	4.75	2.54	8.17
hsa-miR-590-5p	nmiR03587.1:1	Endogenous	66.71	55.71	51.39	52.53	56.63	39.55	47.38	53.38	47.52	55.68	66.23	74.52	73.08	66.63	57.8	53.05	62.72	60.96	72.25	59.39	58.6	67.96	94.4
hsa-miR-591	nmiR03588.1:1	Endogenous	4.92	4.97	6.76	2.76	12.14	14.13	6.26	8.21	8.64	11.46	6.73	2.5	5.32	6.23	4.62	1	6.35	12.51	13.55	5.4	14.25	7.62	9.08
hsa-miR-592	nmiR03589.1:1	Endogenous	9.83	7.96	10.82	4.15	18.2	33.9	5.36	12.32	12.96	16.38	10.1	6.89	6.64	9.96	10.79	12.35	11.91	12.51	33.87	2.16	20.59	8.26	16.34
hsa-miR-593-3p	nmiR03590.1:1	Endogenous	14.04	9.95	6.76	6.91	22.25	11.3	8.05	6.84	11.52	18.01	10.1	5.64	9.3	13.7	5.39	10.17	7.94	18.76	40.64	9.72	12.67	8.89	7.26
hsa-miR-595	nmiR03591.1:1	Endogenous	3.51	9.95	6.76	11.06	16.18	22.6	3.58	10.95	5.76	9.83	4.49	7.51	5.98	8.1	6.94	5.81	9.53	20.32	22.58	6.48	17.42	6.35	8.17
hsa-miR-596	nmiR03592.1:1	Endogenous	15.45	23.87	16.23	13.82	12.14	19.78	17.88	27.38	14.4	21.29	14.59	10.02	17.94	11.21	17.73	15.99	11.91	18.76	33.87	18.36	30.09	15.88	22.69
hsa-miR-597-5p	nmiR03433.1:1	Endogenous	6.32	8.95	9.47	1.38	18.2	8.48	7.15	8.21	8.64	1.64	7.86	3.13	2.66	2.49	2.31	5.09	5.56	7.82	11.29	7.56	9.5	3.81	9.08
hsa-miR-598-3p	nmiR03593.1:1	Endogenous	18.96	31.83	27.05	24.88	26.29	31.08	7.15	12.32	17.28	22.93	25.82	30.68	14.62	24.91	17.73	13.81	24.61	18.76	24.84	30.24	26.92	18.42	31.77
hsa-miR-599	nmiR03594.1:1	Endogenous	1	1	1.35	4.15	6.07	2.83	1.79	2.74	4.32	6.55	3.37	2.5	1.99	1	2.31	4.36	1	3.13	9.03	5.4	3.17	2.54	6.35
hsa-miR-600	nmiR03595.1:1	Endogenous	7.02	6.96	4.06	6.91	16.18	11.3	3.58	1.37	10.08	8.19	6.73	6.26	13.29	5.6	6.17	8.72	10.32	15.63	9.03	9.72	6.33	7.62	7.26
hsa-miR-601	nmiR03596.1:1	Endogenous	5.62	2.98	8.11	2.76	2.02	5.65	3.58	9.58	4.32	4.91	1.12	4.38	3.32	2.49	2.31	2.18	1	3.13	6.77	4.32	7.92	2.54	3.63
hsa-miR-603	nmiR03598.1:1	Endogenous	1	1	1.35	1.38	2.02	2.83	1	1.37	1.44	1.64	1.12	1	1	1	1	1	1	1.56	2.26	1.08	1.58	1	1
hsa-miR-604	nmiR03599.1:1	Endogenous	3.51	5.97	6.76	9.68	4.05	8.48	7.15	4.11	11.52	8.19	4.49	1.88	4.65	3.11	5.39	5.09	3.97	6.25	9.03	8.64	11.09	4.45	3.63
hsa-miR-605-5p	nmiR03600.1:1	Endogenous	4.92	9.95	4.06	2.76	14.16	19.78	7.15	13.69	2.88	13.1	7.86	3.76	3.99	4.98	10.02	7.27	7.15	10.94	18.06	6.48	14.25	6.35	7.26
hsa-miR-606	nmiR03601.1:1	Endogenous	1.4	2.98	2.7	4.15	6.07	8.48	5.36	5.48	2.88	1.64	5.61	1.88	3.32	3.74	2.31	4.36	3.18	1.56	9.03	5.4	3.17	1.91	3.63
hsa-miR-607	nmiR03602.1:1	Endogenous	7.02	7.96	5.41	6.91	14.16	19.78	8.05	13.69	11.52	14.74	13.47	5.01	4.65	5.6	2.31	1.45	7.94	18.76	11.29	9.72	11.09	8.26	6.35
hsa-miR-608	nmiR03603.1:1	Endogenous	11.94	11.94	12.17	11.06	26.29	22.6	16.09	38.33	12.96	14.74	11.22	10.02	5.32	11.21	11.56	10.9	10.32	20.32	38.38	12.96	31.67	12.7	18.15
hsa-miR-610	nmiR03605.1:1	Endogenous	3.51	4.97	6.76	5.53	10.11	5.65	2.68	4.11	4.32	3.28	4.49	3.13	4.65	2.49	4.62	4.36	1	7.82	6.77	6.48	12.67	1.91	7.26
hsa-miR-612	nmiR03607.1:1	Endogenous	13.34	22.88	14.88	23.5	30.34	50.85	20.56	30.11	34.56	29.48	14.59	10.02	12.62	9.34	13.87	12.35	10.32	18.76	33.87	21.6	28.51	12.07	19.97
hsa-miR-613	nmiR03608.1:1	Endogenous	4.21	8.95	13.52	6.91	10.11	14.13	8.05	17.79	12.96	9.83	5.61	10.02	10.63	6.85	9.25	10.9	5.56	4.69	15.8	9.72	14.25	4.45	6.35
hsa-miR-614	nmiR03609.1:1	Endogenous	1	3.98	2.7	2.76	2.02	11.3	5.36	5.48	1.44	3.28	2.24	1.25	3.32	1.87	3.85	1.45	1	10.94	6.77	3.24	6.33	4.45	3.63
hsa-miR-615-3p	nmiR03610.1:1	Endogenous	2.81	2.98	1.35	2.76	10.11	8.48	3.58	8.21	2.88	8.19	1.12	2.5	1.33	2.49	3.85	3.63	3.97	10.94	11.29	7.56	9.5	1	3.63
hsa-miR-615-5p	nmiR03611.1:1	Endogenous	1	4.97	2.7	1.38	6.07	2.83	1.79	6.84	1.44	1.64	3.37	1	1	1	1	1	3.18	4.69	6.77	1.08	11.09	3.18	1.82
hsa-miR-616-3p	nmiR03612.1:1	Endogenous	2.81	2.98	4.06	5.53	10.11	8.48	4.47	5.48	10.08	6.55	6.73	3.13	6.64	5.6	3.08	5.09	4.76	6.25	15.8	6.48	12.67	2.54	8.17
hsa-miR-617	nmiR03613.1:1	Endogenous	2.11	3.98	4.06	6.91	2.02	11.3	2.68	9.58	4.32	6.55	4.49	2.5	1.33	6.85	3.85	2.91	1.59	3.13	11.29	5.4	4.75	4.45	6.35
hsa-miR-619-3p	nmiR03615.1:1	Endogenous	1.4	1	2.7	1.38	6.07	2.83	1.79	5.48	5.76	6.55	2.24	3.76	1.99	2.49	4.62	2.18	2.38	4.69	6.77	2.16	1.58	3.18	5.45
hsa-miR-620	nmiR03616.1:1	Endogenous	3.51	8.95	6.76	9.68	20.23	14.13	8.05	13.69	15.84	11.46	10.1	4.38	3.99	1.87	5.39	4.36	6.35	9.38	15.8	2.16	11.09	5.08	9.08
hsa-miR-624-3p	nmiR03620.1:1	Endogenous	1.4	5.97	4.06	1.38	4.05	11.3	1	5.48	1.44	1.64	4.49	1	2.66	3.74	3.85	1.45	1.59	1.56	4.52	7.56	3.17	3.18	1.82
hsa-miR-625-5p	nmiR03621.1:1	Endogenous	26.69	19.9	17.58	31.8	24.27	11.3	22.35	13.69	15.84	21.29	31.43	21.29	21.92	19.93	16.96	18.17	20.64	20.32	40.64	28.08	34.84	16.51	19.97
hsa-miR-626	nmiR03622.1:1	Endogenous	2.11	2.98	1.35	8.29	8.09	2.83	3.58	2.74	5.76	4.91	5.61	1.88	3.99	3.11	4.62	3.63	3.97	9.38	11.29	6.48	7.92	3.18	5.45
hsa-miR-627-3p	nmiR04017.1:1	Endogenous	9.13	18.9	9.47	5.53	14.16	16.95	11.62	16.43	7.2	11.46	14.59	8.14	11.29	15.57	8.48	10.17	11.91	9.38	18.06	15.12	9.5	6.99	17.25
hsa-miR-627-5p	nmiR03623.1:1	Endogenous	1	1	1.35	1.38	2.02	2.83	1	1.37	1.44	1.64	1.12	1	1	1	1	1	1	1.56	2.26	1.08	1.58	1	1
hsa-miR-628-3p	nmiR03624.1:1	Endogenous	8.43	11.94	12.17	16.59	22.25	8.48	11.62	17.79	10.08	16.38	11.22	12.52	9.97	13.08	14.64	10.9	11.91	20.32	15.8	20.52	12.67	15.24	17.25
hsa-miR-628-5p	nmiR03625.1:1	Endogenous	17.56	18.9	17.58	22.12	22.25	31.08	25.03	28.74	14.4	21.29	25.82	18.16	12.62	17.44	22.35	32.7	26.2	35.95	42.9	20.52	12.67	26.04	19.06
hsa-miR-629-5p	nmiR03626.1:1	Endogenous	4.92	5.97	8.11	5.53	8.09	2.83	6.26	9.58	5.76	11.46	7.86	5.64	4.65	3.11	7.71	8.72	5.56	17.19	11.29	10.8	12.67	8.26	9.98
hsa-miR-630	nmiR03627.1:1	Endogenous	2.11	2.98	2.7	4.15	8.09	5.65	4.47	5.48	1.44	8.19	2.24	5.01	4.65	7.47	3.08	2.91	3.97	15.63	13.55	7.56	9.5	3.81	7.26
hsa-miR-631	nmiR03628.1:1	Endogenous	3.51	3.98	2.7	9.68	4.05	5.65	5.36	10.95	7.2	6.55	5.61	1.88	3.99	5.6	6.17	5.81	3.18	6.25	9.03	6.48	14.25	5.72	12.71
hsa-miR-637	nmiR03634.1:1	Endogenous	4.21	6.96	6.76	2.76	4.05	2.83	12.52	6.84	8.64	14.74	12.35	9.39	9.3	6.23	9.25	6.54	5.56	14.07	15.8	9.72	4.75	8.26	9.08
hsa-miR-638	nmiR03635.1:1	Endogenous	3.51	7.96	4.06	2.76	4.05	11.3	6.26	6.84	2.88	9.83	5.61	1.25	4.65	2.49	4.62	2.91	3.18	10.94	9.03	4.32	9.5	1.91	4.54
hsa-miR-639	nmiR03636.1:1	Endogenous	4.92	7.96	4.06	8.29	6.07	5.65	5.36	5.48	4.32	6.55	3.37	3.76	1.99	1.25	3.08	2.91	4.76	7.82	20.32	3.24	9.5	2.54	6.35
hsa-miR-640	nmiR03637.1:1	Endogenous	7.02	9.95	6.76	4.15	14.16	19.78	5.36	5.48	10.08	8.19	12.35	5.01	6.64	10.59	9.25	6.54	8.73	12.51	20.32	9.72	14.25	6.99	8.17
hsa-miR-641	nmiR03638.1:1	Endogenous	3.51	3.98	1.35	4.15	6.07	5.65	3.58	13.69	4.32	3.28	4.49	1.88	1	1.87	2.31	3.63	3.18	1.56	4.52	6.48	4.75	2.54	5.45
hsa-miR-642a-3p	nmiR03951.1:1	Endogenous	4.21	7.96	4.06	9.68	10.11	19.78	1.79	6.84	4.32	13.1	6.73	3.76	4.65	8.1	3.08	6.54	5.56	12.51	15.8	8.64	7.92	3.81	9.98
hsa-miR-642a-5p	nmiR03639.1:1	Endogenous	45.65	69.63	40.57	81.56	28.32	36.73	46.49	26.01	46.08	45.85	52.76	52.6	17.94	32.38	36.22	21.8	36.52	29.7	85.79	31.32	58.6	61.61	49.02
hsa-miR-643	nmiR03641.1:1	Endogenous	1.4	9.95	6.76	2.76	14.16	5.65	7.15	4.11	7.2	4.91	7.86	1.88	4.65	1	4.62	3.63	7.15	4.69	20.32	5.4	7.92	5.72	3.63
hsa-miR-644a	nmiR03643.1:1	Endogenous	6.32	5.97	12.17	1.38	12.14	14.13	7.15	10.95	10.08	11.46	6.73	4.38	1.33	6.23	3.08	7.27	5.56	12.51	15.8	12.96	14.25	2.54	9.08
hsa-miR-648	nmiR03647.1:1	Endogenous	2.81	6.96	8.11	5.53	8.09	2.83	5.36	9.58	7.2	11.46	4.49	2.5	5.32	7.47	1	6.54	3.18	10.94	15.8	11.88	9.5	2.54	8.17
hsa-miR-649	nmiR03648.1:1	Endogenous	5.62	6.96	6.76	4.15	14.16	14.13	6.26	12.32	5.76	6.55	5.61	4.38	7.97	2.49	5.39	2.91	5.56	12.51	9.03	11.88	4.75	4.45	3.63
hsa-miR-650	nmiR03649.1:1	Endogenous	2.11	3.98	4.06	5.53	18.2	11.3	2.68	9.58	2.88	3.28	5.61	2.5	1.33	5.6	3.08	1.45	3.18	9.38	6.77	9.72	3.17	2.54	1.82
hsa-miR-6503-3p	nmiR03995.1:1	Endogenous	4.92	10.94	4.06	4.15	8.09	5.65	16.99	27.38	20.16	3.28	4.49	4.38	9.97	4.36	5.39	21.8	8.73	12.51	20.32	2.16	9.5	4.45	13.62
hsa-miR-6503-5p	nmiR03994.1:1	Endogenous	1	3.98	6.76	2.76	8.09	14.13	3.58	5.48	2.88	6.55	4.49	3.76	1.99	3.11	3.85	4.36	5.56	7.82	9.03	2.16	7.92	1.91	8.17
hsa-miR-651-3p	nmiR04018.1:1	Endogenous	2.81	4.97	4.06	1.38	8.09	2.83	1.79	5.48	5.76	4.91	3.37	1.88	2.66	2.49	2.31	1.45	2.38	3.13	2.26	1.08	4.75	1.91	3.63
hsa-miR-651-5p	nmiR03650.1:1	Endogenous	18.96	15.92	13.52	12.44	36.41	19.78	23.24	17.79	10.08	21.29	26.94	13.78	12.62	20.55	16.96	16.71	16.67	34.39	36.12	21.6	25.34	13.34	14.52
hsa-miR-6511a-3p	nmiR03997.1:1	Endogenous	3.51	1.99	5.41	5.53	10.11	5.65	2.68	6.84	5.76	6.55	10.1	4.38	9.3	6.85	6.94	6.54	7.94	15.63	9.03	8.64	1.58	4.45	6.35
hsa-miR-6511a-5p	nmiR03996.1:1	Endogenous	1	4.97	1.35	1.38	6.07	2.83	1.79	5.48	4.32	3.28	1.12	3.13	1.33	1.87	1	4.36	1.59	4.69	6.77	5.4	1.58	2.54	1.82
hsa-miR-652-3p	nmiR03651.1:1	Endogenous	25.98	13.93	24.34	34.56	22.25	22.6	28.61	19.16	21.6	26.2	12.35	19.41	30.56	19.3	31.6	33.43	26.2	29.7	20.32	19.44	28.51	25.4	26.32
hsa-miR-652-5p	nmiR03974.1:1	Endogenous	1	1	2.7	2.76	10.11	2.83	1	1.37	2.88	1.64	2.24	1	1.99	1.25	1.54	1	3.18	1.56	2.26	2.16	3.17	1.27	1.82
hsa-miR-654-3p	nmiR03653.1:1	Endogenous	2.11	2.98	4.06	4.15	10.11	8.48	3.58	12.32	7.2	6.55	3.37	1.25	3.32	3.11	3.85	4.36	3.18	12.51	13.55	1.08	6.33	2.54	1.82
hsa-miR-654-5p	nmiR03654.1:1	Endogenous	9.83	8.95	1.35	5.53	12.14	11.3	5.36	8.21	8.64	8.19	6.73	5.64	5.98	8.1	6.17	7.99	7.15	10.94	20.32	6.48	6.33	6.35	14.52
hsa-miR-655-3p	nmiR03655.1:1	Endogenous	1	1.99	4.06	5.53	6.07	14.13	2.68	8.21	5.76	4.91	2.24	2.5	1	5.6	3.08	2.18	3.97	3.13	6.77	6.48	7.92	3.81	8.17
hsa-miR-656-3p	nmiR03656.1:1	Endogenous	1	1	1.35	1.38	8.09	11.3	1	6.84	1.44	4.91	1.12	1	1	1	1	1	1	9.38	2.26	1.08	1.58	1	1
hsa-miR-660-3p	nmiR03975.1:1	Endogenous	5.62	1	4.06	2.76	2.02	11.3	7.15	5.48	10.08	3.28	6.73	2.5	4.65	1.87	7.71	3.63	3.97	7.82	11.29	2.16	6.33	3.81	8.17
hsa-miR-660-5p	nmiR03660.1:1	Endogenous	35.81	33.82	70.33	58.06	26.29	53.68	50.96	62.96	33.12	40.94	42.65	36.32	39.2	37.99	54.72	39.97	38.11	42.21	45.16	52.91	63.35	42.55	38.12
hsa-miR-661	nmiR03661.1:1	Endogenous	2.81	13.93	6.76	8.29	20.23	31.08	1	6.84	1.44	9.83	3.37	6.26	3.32	7.47	2.31	1	8.73	26.57	24.84	5.4	28.51	5.72	13.62
hsa-miR-663a	nmiR03664.1:1	Endogenous	4.21	3.98	2.7	1.38	6.07	2.83	6.26	4.11	2.88	3.28	6.73	2.5	2.66	2.49	1.54	1.45	3.97	6.25	6.77	7.56	6.33	1.91	3.63
hsa-miR-664a-3p	nmiR03665.1:1	Endogenous	70.93	91.52	55.45	92.62	48.54	62.15	64.36	41.06	47.52	73.69	80.82	65.75	60.46	64.14	54.72	46.51	67.49	56.27	58.7	43.19	52.26	66.69	58.09
hsa-miR-664b-3p	nmiR03962.1:1	Endogenous	4.21	8.95	8.11	4.15	10.11	11.3	5.36	13.69	4.32	13.1	13.47	5.64	11.29	6.23	9.25	6.54	10.32	6.25	18.06	11.88	7.92	6.35	4.54
hsa-miR-664b-5p	nmiR03961.1:1	Endogenous	1	1	1.35	2.76	2.02	11.3	1	5.48	2.88	1.64	2.24	1	1.99	3.11	1.54	2.91	3.18	3.13	4.52	2.16	9.5	3.18	3.63
hsa-miR-665	nmiR03666.1:1	Endogenous	5.62	7.96	6.76	6.91	8.09	19.78	9.83	6.84	12.96	6.55	8.98	3.76	4.65	6.85	3.85	2.91	3.97	15.63	4.52	5.4	4.75	5.08	7.26
hsa-miR-671-3p	nmiR03670.1:1	Endogenous	1	1.99	5.41	2.76	6.07	5.65	8.94	4.11	5.76	1.64	2.24	1.88	2.66	3.74	5.39	1	1	3.13	11.29	4.32	6.33	1.91	5.45
hsa-miR-671-5p	nmiR03671.1:1	Endogenous	2.81	5.97	4.06	5.53	8.09	28.25	9.83	10.95	5.76	11.46	6.73	3.76	5.32	3.74	1	5.09	5.56	9.38	15.8	9.72	17.42	6.35	7.26
hsa-miR-6720-3p	nmiR03998.1:1	Endogenous	1.4	1.99	1.35	1.38	4.05	2.83	1	4.11	4.32	3.28	3.37	1.25	1.33	1.87	2.31	1.45	1	6.25	6.77	4.32	9.5	1.27	4.54
hsa-miR-6721-5p	nmiR03999.1:1	Endogenous	4.21	6.96	5.41	12.44	20.23	22.6	8.94	13.69	17.28	1.64	6.73	3.76	3.99	6.85	5.39	3.63	3.18	7.82	11.29	7.56	15.84	9.53	3.63
hsa-miR-6724-5p	nmiR04000.1:1	Endogenous	9.13	19.9	12.17	8.29	8.09	19.78	5.36	9.58	8.64	21.29	22.45	13.15	17.27	7.47	10.79	7.27	12.7	20.32	27.09	11.88	20.59	6.35	14.52
hsa-miR-675-5p	nmiR03672.1:1	Endogenous	7.02	1.99	10.82	9.68	10.11	22.6	6.26	4.11	10.08	13.1	6.73	4.38	5.32	4.98	8.48	7.27	5.56	3.13	11.29	11.88	7.92	3.18	2.72
hsa-miR-7-5p	nmiR03123.1:1	Endogenous	49.86	69.63	54.1	47	60.68	101.71	103.7	157.41	82.07	83.51	62.86	72.64	52.49	59.78	86.32	115.54	71.46	50.02	76.76	63.71	80.77	74.94	45.39
hsa-miR-708-5p	nmiR03673.1:1	Endogenous	4.21	1.99	5.41	2.76	2.02	2.83	1.79	2.74	8.64	4.91	3.37	2.5	3.99	2.49	6.94	2.18	3.18	3.13	9.03	4.32	6.33	2.54	4.54
hsa-miR-744-5p	nmiR03677.1:1	Endogenous	2.11	6.96	4.06	2.76	10.11	11.3	6.26	5.48	5.76	8.19	4.49	3.13	3.32	6.85	7.71	2.18	5.56	3.13	9.03	6.48	4.75	8.26	10.89
hsa-miR-758-3p+hsa-miR-411-3p	nmiR03678.1:1	Endogenous	9.83	3.98	10.82	11.06	14.16	16.95	1.79	4.11	14.4	16.38	4.49	5.01	8.64	6.85	9.25	4.36	6.35	21.88	18.06	3.24	19	7.62	9.98
hsa-miR-758-5p	nmiR03993.1:1	Endogenous	4.21	4.97	4.06	6.91	2.02	2.83	6.26	6.84	1.44	1.64	4.49	6.26	9.3	2.49	2.31	7.27	3.97	7.82	9.03	7.56	11.09	2.54	4.54
hsa-miR-760	nmiR03680.1:1	Endogenous	7.02	9.95	9.47	2.76	2.02	19.78	12.52	9.58	14.4	11.46	7.86	6.26	10.63	4.98	6.17	10.17	11.91	4.69	6.77	6.48	12.67	5.72	8.17
hsa-miR-761	nmiR03681.1:1	Endogenous	3.51	1.99	6.76	2.76	6.07	16.95	4.47	13.69	5.76	3.28	4.49	2.5	1.99	5.6	4.62	3.63	7.15	6.25	18.06	4.32	15.84	2.54	7.26
hsa-miR-764	nmiR03683.1:1	Endogenous	3.51	5.97	4.06	4.15	10.11	19.78	4.47	6.84	8.64	3.28	7.86	2.5	2.66	4.98	2.31	6.54	6.35	20.32	20.32	4.32	19	1.91	6.35
hsa-miR-765	nmiR03684.1:1	Endogenous	2.81	6.96	2.7	5.53	22.25	31.08	7.15	12.32	12.96	8.19	12.35	8.14	4.65	4.98	11.56	10.17	10.32	12.51	11.29	7.56	11.09	8.89	17.25
hsa-miR-766-3p	nmiR03685.1:1	Endogenous	7.02	12.93	6.76	8.29	14.16	14.13	4.47	5.48	7.2	11.46	11.22	5.64	4.65	4.98	11.56	4.36	7.94	18.76	4.52	18.36	7.92	10.16	6.35
hsa-miR-766-5p	nmiR03978.1:1	Endogenous	1.4	1	2.7	1.38	2.02	8.48	1	5.48	1.44	4.91	3.37	1	1.99	1.25	2.31	3.63	2.38	1.56	6.77	1.08	1.58	3.18	2.72
hsa-miR-767-3p	nmiR03686.1:1	Endogenous	5.62	4.97	4.06	5.53	4.05	22.6	4.47	5.48	1.44	6.55	4.49	3.13	1.33	1.87	3.85	5.81	3.18	4.69	6.77	4.32	4.75	5.08	7.26
hsa-miR-767-5p	nmiR03687.1:1	Endogenous	3.51	6.96	6.76	1.38	18.2	5.65	5.36	10.95	7.2	18.01	12.35	2.5	1.99	1.87	6.17	5.09	7.94	4.69	15.8	10.8	6.33	7.62	2.72
hsa-miR-769-3p	nmiR03688.1:1	Endogenous	1	1	1.35	1.38	2.02	2.83	1	1.37	1.44	3.28	1.12	1	1	1	1	1	1	1.56	2.26	1.08	1.58	1	1
hsa-miR-769-5p	nmiR03689.1:1	Endogenous	32.3	35.81	25.7	20.74	26.29	28.25	27.71	19.16	30.24	24.56	24.69	26.93	35.88	23.66	23.89	15.26	25.41	23.45	38.38	24.84	25.34	18.42	35.4
hsa-miR-770-5p	nmiR03690.1:1	Endogenous	7.72	13.93	4.06	11.06	14.16	28.25	10.73	12.32	12.96	6.55	15.71	3.76	4.65	4.36	4.62	5.09	8.73	15.63	18.06	9.72	17.42	5.72	9.08
hsa-miR-802	nmiR03691.1:1	Endogenous	2.81	4.97	1.35	2.76	6.07	14.13	3.58	9.58	7.2	8.19	6.73	4.38	4.65	3.74	3.08	1	1.59	14.07	13.55	4.32	9.5	6.99	7.26

hsa-miR-873-3p	nmiR03979.1:1	Endogenous	7.72	12.93	5.41	9.68	6.07	25.43	11.62	5.48	12.96	13.1	11.22	3.13	6.64	4.98	6.94	6.54	5.56	9.38	6.77	17.28	14.25	3.81	10.89
hsa-miR-873-5p	nmiR03692.1:1	Endogenous	6.32	11.94	8.11	4.15	8.09	16.95	16.09	28.74	12.96	8.19	12.35	6.26	6.64	6.85	9.25	14.53	4.76	1.56	22.58	6.48	19	8.89	13.62
hsa-miR-874-3p	nmiR03693.1:1	Endogenous	1	3.98	1.35	6.91	6.07	8.48	5.36	6.84	4.32	1.64	4.49	3.13	3.32	1.25	1	4.36	2.38	4.69	9.03	2.16	6.33	3.81	2.72
hsa-miR-874-5p	nmiR04020.1:1	Endogenous	3.51	5.97	4.06	4.15	4.05	2.83	3.58	5.48	10.08	1.64	5.61	2.5	4.65	7.47	7.71	3.63	5.56	10.94	13.55	7.56	4.75	1.91	4.54
hsa-miR-875-3p	nmiR03694.1:1	Endogenous	1.4	1	1.35	4.15	8.09	8.48	3.58	1.37	4.32	9.83	1.12	1.25	1.33	4.98	1	1	4.76	7.82	11.29	4.32	11.09	4.45	1.82
hsa-miR-876-3p	nmiR03696.1:1	Endogenous	2.81	2.98	5.41	4.15	8.09	19.78	10.73	5.48	18.72	9.83	7.86	5.01	3.99	9.96	3.85	7.27	5.56	10.94	18.06	8.64	12.67	6.99	5.45
hsa-miR-876-5p	nmiR03697.1:1	Endogenous	1	1	1.35	1.38	4.05	8.48	1	8.21	5.76	4.91	1.12	1	1	1	1	2.18	2.38	3.13	4.52	2.16	3.17	1.91	2.72
hsa-miR-877-5p	nmiR03698.1:1	Endogenous	8.43	12.93	5.41	8.29	10.11	14.13	8.94	5.48	8.64	8.19	8.98	4.38	5.32	4.36	6.17	4.36	6.35	7.82	22.58	4.32	11.09	3.18	7.26
hsa-miR-885-3p	nmiR03699.1:1	Endogenous	2.11	4.97	1.35	1.38	6.07	5.65	1.79	9.58	4.32	9.83	4.49	2.5	1.99	2.49	3.85	1.45	3.97	1.56	4.52	6.48	1.58	2.54	6.35
hsa-miR-885-5p	nmiR03700.1:1	Endogenous	1.4	4.97	2.7	2.76	4.05	11.3	1	5.48	4.32	1.64	6.73	3.13	1.33	4.98	3.85	2.91	1.59	1.56	13.55	1.08	3.17	1.91	1.82
hsa-miR-887-3p	nmiR03701.1:1	Endogenous	1.4	4.97	2.7	6.91	10.11	5.65	4.47	1.37	2.88	1.64	4.49	1.88	1.99	1.87	3.08	4.36	5.56	9.38	6.77	4.32	3.17	2.54	6.35
hsa-miR-887-5p	nmiR04021.1:1	Endogenous	4.92	6.96	2.7	6.91	22.25	11.3	7.15	8.21	4.32	4.91	8.98	1.25	5.98	3.74	3.08	7.27	3.97	9.38	20.32	6.48	9.5	3.81	9.98
hsa-miR-888-5p	nmiR03702.1:1	Endogenous	9.13	14.92	8.11	2.76	12.14	28.25	8.05	10.95	12.96	16.38	5.61	7.51	9.3	5.6	6.17	6.54	5.56	15.63	24.84	6.48	17.42	4.45	9.08
hsa-miR-889-3p	nmiR03436.1:1	Endogenous	7.02	13.93	8.11	8.29	10.11	2.83	3.58	9.58	7.2	16.38	7.86	5.01	5.32	5.6	7.71	7.99	12.7	12.51	9.03	11.88	19	6.99	13.62
hsa-miR-890	nmiR03703.1:1	Endogenous	3.51	3.98	2.7	4.15	8.09	11.3	8.05	9.58	5.76	6.55	8.98	1.88	1	5.6	2.31	5.09	1	4.69	2.26	6.48	11.09	2.54	6.35
hsa-miR-891a-5p	nmiR03704.1:1	Endogenous	1.4	2.98	1.35	4.15	2.02	8.48	1.79	1.37	1.44	3.28	4.49	1.25	3.32	1.25	2.31	3.63	1.59	1.56	4.52	6.48	6.33	2.54	2.72
hsa-miR-891b	nmiR03705.1:1	Endogenous	7.72	7.96	9.47	6.91	12.14	22.6	8.94	17.79	8.64	8.19	10.1	2.5	7.31	4.98	12.33	13.08	7.15	9.38	11.29	9.72	6.33	6.99	2.72
hsa-miR-892a	nmiR03706.1:1	Endogenous	7.72	24.87	4.06	6.91	16.18	14.13	19.67	20.53	14.4	11.46	15.71	9.39	19.27	11.21	10.79	15.99	15.88	6.25	22.58	7.56	17.42	8.89	9.08
hsa-miR-892b	nmiR03707.1:1	Endogenous	1	1.99	2.7	1.38	2.02	2.83	2.68	1.37	2.88	3.28	4.49	1	1	1.25	3.08	2.18	3.18	4.69	2.26	3.24	12.67	1.91	1.82
hsa-miR-9-5p	nmiR03124.1:1	Endogenous	16.85	10.94	8.11	5.53	2.02	19.78	14.3	9.58	17.28	14.74	4.49	7.51	7.31	6.23	10.02	5.81	11.12	15.63	18.06	15.12	9.5	13.34	12.71
hsa-miR-922	nmiR03710.1:1	Endogenous	4.21	6.96	5.41	5.53	10.11	14.13	3.58	4.11	5.76	9.83	3.37	8.14	3.99	5.6	7.71	2.18	3.18	10.94	11.29	6.48	11.09	3.18	9.08
hsa-miR-924	nmiR03711.1:1	Endogenous	1	1.99	1.35	1.38	4.05	5.65	1	1.37	1.44	1.64	1.12	2.5	3.99	1	1	1	2.38	6.25	6.77	1.08	6.33	1.27	3.63
hsa-miR-92a-1-5p	nmiR03919.1:1	Endogenous	4.92	5.97	1.35	12.44	8.09	5.65	3.58	9.58	8.64	6.55	3.37	5.64	3.99	7.47	5.39	4.36	7.15	7.82	9.03	7.56	12.67	6.35	13.62
hsa-miR-92a-3p	nmiR03169.1:1	Endogenous	48.45	49.74	56.8	56.68	44.5	62.15	27.71	32.85	44.64	22.93	55	51.35	52.49	52.93	31.6	42.15	27.79	26.57	42.9	33.48	47.51	33.03	59.91
hsa-miR-92b-3p	nmiR03171.1:1	Endogenous	3.51	6.96	8.11	4.15	2.02	11.3	3.58	6.84	8.64	8.19	4.49	2.5	2.66	1.87	4.62	9.45	5.56	10.94	15.8	3.24	9.5	4.45	7.26
hsa-miR-93-5p	nmiR03172.1:1	Endogenous	523.87	483.46	677.57	584.76	669.46	483.11	640.96	735.04	460.77	466.69	392.87	412.04	417.23	441.52	538.71	765.94	627.22	490.83	494.45	619.84	513.13	593.19	436.62
hsa-miR-933	nmiR03712.1:1	Endogenous	4.92	4.97	1.35	1.38	8.09	2.83	4.47	4.11	5.76	6.55	4.49	1.88	3.99	4.36	3.08	2.91	1.59	9.38	9.03	7.56	4.75	1.27	10.89
hsa-miR-934	nmiR03713.1:1	Endogenous	2.11	3.98	2.7	6.91	4.05	14.13	1.79	9.58	2.88	6.55	10.1	4.38	4.65	3.11	6.17	4.36	1	14.07	2.26	10.8	12.67	7.62	8.17
hsa-miR-935	nmiR03714.1:1	Endogenous	5.62	7.96	8.11	4.15	6.07	14.13	8.05	12.32	4.32	9.83	7.86	3.76	5.32	9.34	7.71	10.17	7.94	15.63	4.52	11.88	9.5	5.08	5.45
hsa-miR-936	nmiR03715.1:1	Endogenous	6.32	5.97	4.06	11.06	14.16	14.13	6.26	9.58	7.2	4.91	6.73	3.76	6.64	1.87	5.39	9.45	7.94	14.07	9.03	12.96	11.09	5.08	9.98
hsa-miR-937-3p	nmiR03716.1:1	Endogenous	4.21	7.96	1.35	4.15	10.11	2.83	4.47	6.84	7.2	9.83	3.37	6.89	2.66	4.98	7.71	4.36	1.59	4.69	2.26	3.24	14.25	1.27	7.26
hsa-miR-939-5p	nmiR03718.1:1	Endogenous	3.51	7.96	2.7	1.38	4.05	8.48	2.68	5.48	5.76	3.28	2.24	1.88	1.33	2.49	1.54	1	3.18	4.69	20.32	6.48	4.75	2.54	4.54
hsa-miR-940	nmiR03719.1:1	Endogenous	2.11	1.99	1.35	1.38	2.02	5.65	2.68	2.74	4.32	3.28	4.49	1.25	1.33	1	6.17	6.54	3.97	3.13	11.29	4.32	6.33	1.27	1.82
hsa-miR-941	nmiR03720.1:1	Endogenous	3.51	7.96	6.76	6.91	18.2	14.13	8.94	12.32	12.96	11.46	13.47	4.38	4.65	8.1	6.94	4.36	7.15	6.25	11.29	9.72	3.17	8.26	9.98
hsa-miR-942-3p	nmiR04023.1:1	Endogenous	2.81	6.96	8.11	2.76	2.02	2.83	11.62	17.79	8.64	13.1	4.49	3.76	5.98	3.74	8.48	15.99	7.15	9.38	11.29	5.4	12.67	3.18	10.89
hsa-miR-942-5p	nmiR03721.1:1	Endogenous	2.11	2.98	6.76	1.38	6.07	8.48	8.05	8.21	2.88	4.91	3.37	3.13	1.99	1	1.54	6.54	3.18	1.56	6.77	1.08	4.75	1.27	4.54
hsa-miR-944	nmiR03723.1:1	Endogenous	2.11	2.98	2.7	1.38	6.07	8.48	4.47	2.74	10.08	1.64	5.61	1.88	3.99	1.25	3.08	2.18	1	1.56	6.77	4.32	3.17	3.81	4.54
hsa-miR-95-3p	nmiR03173.1:1	Endogenous	20.36	14.92	12.17	15.21	26.29	33.9	16.99	20.53	18.72	14.74	19.08	18.16	15.28	23.66	10.79	5.81	19.85	23.45	20.32	10.8	17.42	15.24	12.71
hsa-miR-96-5p	nmiR03174.1:1	Endogenous	4.21	13.93	10.82	12.44	22.25	19.78	10.73	16.43	8.64	9.83	7.86	1	5.98	4.98	7.71	7.27	3.97	12.51	22.58	7.56	14.25	4.45	3.63
hsa-miR-98-3p	nmiR03987.1:1	Endogenous	2.81	2.98	2.7	5.53	8.09	5.65	7.15	1.37	2.88	4.91	5.61	1.88	1.99	1.87	4.62	3.63	3.97	7.82	6.77	2.16	3.17	1.27	6.35
hsa-miR-98-5p	nmiR03175.1:1	Endogenous	395.36	329.27	309.71	319.33	410.57	372.93	269.97	275.12	299.5	484.71	380.53	352.55	348.14	406.65	285.15	252.16	330.28	340.77	252.87	304.52	205.89	293.42	249.62
hsa-miR-99a-5p	nmiR03177.1:1	Endogenous	7.72	30.84	16.23	33.18	30.34	33.9	26.82	13.69	14.4	24.56	31.43	14.4	22.59	16.19	15.41	11.63	25.41	20.32	18.06	15.12	28.51	19.05	13.62
hsa-miR-99b-5p	nmiR03178.1:1	Endogenous	20.36	17.91	27.05	27.65	28.32	31.08	8.05	26.01	33.12	39.3	20.2	15.66	15.28	21.8	21.58	35.61	26.2	18.76	13.55	35.64	17.42	27.94	14.52
ACTB	NM_001101.2:1010	Housekeeping	1177.65	1464.3	2016.49	2010.01	2825.48	3362	1290.87	2834.75	1854.59	1917.54	903.61	810.93	1118.16	609.04	1164.5	1410.52	859.06	2476.04	5154.46	2611.1	1987.6	1169.23	1020.28
B2M	NM_004048.2:25	Housekeeping	7969.7	9359.81	10193.4	12690.5	14881.8	17555.9	10836.5	21498.1	11954	15566.3	5340.83	5765.43	6635.22	5779.01	9736.81	10766.7	6943.1	16838.3	28289.7	13348.1	13096	7557.74	5764.97
GAPDH	NM_002046.3:35	Housekeeping	337.78	351.15	692.45	699.5	904.07	1209.19	994.97	1478.28	1128.88	1481.96	440.02	448.36	386.67	455.22	859.31	854.59	432.7	1547.53	1657.19	1182.45	1156.13	725.29	399.4
RPL19	NM_000981.3:315	Housekeeping	933.27	1799.54	1727.07	2446.85	3138.97	3449.58	2896.41	3274.12	2384.47	2303.99	1354.85	1034.48	1035.78	1021.91	1773.34	1512.26	1707.79	3520.23	5405.07	2276.34	3642.61	1918.65	1023.91
RPLP0	NM_001002.3:250	Housekeeping	483.84	885.35	980.52	1345.08	1763.65	1952.22	1669.9	2135.3	1500.38	1290.37	772.28	562.95	595.95	626.47	1045.05	1028.28	1059.92	2213.43	3029.91	1411.38	2120.63	1065.7	580.94
LIG_NEG_A	nmiR00810.1:1	Ligation	33.71	66.65	39.22	37.32	32.36	59.33	40.23	42.43	40.32	26.2	41.53	28.18	39.2	31.14	32.37	26.89	38.11	28.14	38.38	24.84	69.68	36.2	30.86
LIG_NEG_B	nmiR00828.1:1	Ligation	2.11	7.96	2.7	5.53	12.14	25.43	9.83	12.32	7.2	9.83	4.49	3.13	3.32	2.49	7.71	6.54	3.97	10.94	20.32	5.4	12.67	4.45	9.08
LIG_NEG_C	nmiR00803.1:1	Ligation	4.21	5.97	4.06	1.38	10.11	11.3	1.79	5.48	7.2	9.83	4.49	1.25	5.98	3.11	3.85	2.18	3.97	6.25	15.8	4.32	6.33	2.54	6.35
LIG_POS_A	nmiR00813.1:1	Ligation	1955.73	2227.3	2721.11	2267.14	1988.15	2319.5	997.65	670.7	1737.96	1298.56	3112.68	2295.02	1860.28	1930.49	1700.9	906.19	1776.86	1306.8	2576.1	1688.9	2084.21	1602.37	3633.63
LIG_POS_B	nmiR00809.1:1	Ligation	689.6	743.09	838.51	640.05	560.24	683.7	299.47	214.9	601.88	455.23	1265.05	866.04	796.6	731.09	584.95	276.14	492.25	453.32	873.75	557.21	643	506.18	1350.7
LIG_POS_C	nmiR00811.1:1	Ligation	51.97	74.61	83.85	81.56	88.99	56.5	20.56	30.11	61.92	57.31	83.06	50.72	46.51	50.44	55.49	33.43	54.78	39.08	83.54	43.19	71.27	38.74	121.64
NEG_A	ERCC_00096.1:230	Negative	1	6	3	3	2	6	4	6	4	2	7	2	7	7	6	12	1	6	9	5	5	1	10
NEG_B	ERCC_00041.1:440	Negative	4	4	1	3	3	4	6	1	3	1	3	5	7	10	4	7	6	5	7	2	4	5	5
NEG_C	ERCC_00019.1:140	Negative	10	7	3	2	5	5	10	11	9	16	10	3	14	14	8	5	10	11	7	8	10	7	9
NEG_D	ERCC_00076.1:355	Negative	7	11	4	9	3	2	9	6	5	6	6	8	9	7	11	7	7	13	9	6	7	7	11
NEG_E	ERCC_00098.1:785	Negative	3	6	5	4	2	6	6	5	5	7	5	10	10	10	10	6	5	4	11	10	5	9	13
NEG_F	ERCC_00126.1:220	Negative	16	17	7	6	10	6	7	13	14	5	11	8	13	15	16	14	17	11	11	11	14	13	13
NEG_G	ERCC_00144.1:15	Negative	1	1	2	1	3	1	2	5	2	4	1	1	2	2	6	5	2	4	1	2	4	5	1
NEG_H	ERCC_00154.1:115	Negative	3	1	2	3	1	1	3	1	3	5	8	6	3	3	3	4	2	3	1	2	5	4	3
POS_A	ERCC_00117.1:385	Positive	14121	16941	12018	14298	15685	13636	16180	15975	15998	15247	15402	16347	13376	18092	19084	17390	18480	19777	18619	18224	18913	16909	21387
POS_B	ERCC_00112.1:695	Positive	4194	4921	3513	4351	4611	3989	4746	4537	4510	4363	4555	4507	3944	5340	5438	4916	5351	5662	5434	5122	5368	4940	6176
POS_C	ERCC_00002.1:850	Positive	1183	1345	977	1259	1279	1130	1337	1316	1344	1241	1294	1320	1103	1463	1486	1450	1607	1632	1559	1562	1577	1447	1773
POS_D	ERCC_00092.1:540	Positive	237	289	226	275	268	249	264	308	302	258	296	274	289	305	332	325	338	330	336	349	325	352	328
POS_E	ERCC_00035.1:485	Positive	46	40	36	42	58	49	55	47	60	46	46	47	47	47	68	47	53	87	74	68	62	78	80
POS_F	ERCC_00034.1:195	Positive	49	45	18	27	29	34	55	67	35	37	38	44	44	34	58	78	46	50	42	35	57	41	59
ath-miR159a	nmiR01610.1:1	SpikeIn	3.51	3.98	1.35	6.91	14.16	2.83	4.47	9.58	2.88	4.91	5.61	5.64	8.64	4.98	6.94	3.63	3.97	12.51	13.55	7.56	11.09	2.54	7.26
cel-miR-248	nmiR01612.1:1	SpikeIn	4.92	7.96	2.7	2.76	2.02	8.48	4.47	5.48	5.76	9.83	5.61	2.5	7.31	1.87	5.39	2.18	1.59	3.13	15.8	4.32	15.84	1.91	7.26
cel-miR-254	nmiR01613.1:1	SpikeIn	3.51	4.97	2.7	6.91	8.09	2.83	6.26	5.48	8.64	3.28	5.61	3.13	3.32	4.36	3.85	2.18	3.18	7.82	6.77	12.96	9.5	6.99	3.63
osa-miR414	nmiR01622.1:1	SpikeIn	2.11	3.98	5.41	4.15	8.09	8.48	4.47	8.21	7.2	1.64	4.49	5.64	3.99	3.74	2.31	2.18	1.59	4.69	13.55	7.56	9.5	4.45	1.82
osa-miR442	nmiR01625.1:1	SpikeIn	29.49	30.84	36.52	42.85	32.36	36.73	18.77	24.64	46.08	36.03	26.94	26.3	19.27	26.78	35.45	21.8	36.52	31.26	49.67	35.64	28.51	32.39	27.23

Supplementary table 4. A list of pathways enriched with miRNA target genes significantly

miRNA target gene groups	PANTHER Pathways	Number of human reference set (20851)	Number of target genes	Expected number of target genes	Fold enrichment	raw P-value	FDR
hsa-miR-144-3p	Gonadotropin-releasing hormone receptor pathway (P06664)	232	36	11.49	3.13	1.60E-08	1.31E-06
	Wnt signaling pathway (P00057)	317	38	15.7	2.42	2.92E-06	1.60E-04
	Integrin signalling pathway (P00034)	191	25	9.46	2.64	3.35E-05	1.37E-03
	PDGF signaling pathway (P00047)	145	20	7.18	2.78	1.05E-04	3.45E-03
	Angiogenesis (P00005)	172	22	8.52	2.58	2.12E-04	5.80E-03
	FGF signaling pathway (P00021)	121	17	5.99	2.84	2.78E-04	6.51E-03
	Hedgehog signaling pathway (P00025)	22	7	1.09	6.42	3.20E-04	6.56E-03
	EGF receptor signaling pathway (P00018)	136	17	6.74	2.52	9.22E-04	1.51E-02
	CCKR signaling map (P06959)	172	20	8.52	2.35	8.72E-04	1.59E-02
	Insulin/IGF pathway-protein kinase B signaling cascade (P00033)	39	8	1.93	4.14	1.46E-03	2.18E-02
	Interferon-gamma signaling pathway (P00035)	31	7	1.54	4.56	1.79E-03	2.44E-02
hsa-miR-1-3p	Gonadotropin-releasing hormone receptor pathway (P06664)	232	28	9.89	2.83	3.17E-06	5.19E-04
	Adrenaline and noradrenaline biosynthesis (P00001)	29	7	1.24	5.66	5.52E-04	1.51E-02
	EGF receptor signaling pathway (P00018)	136	16	5.8	2.76	5.08E-04	1.66E-02
	Angiogenesis (P00005)	172	19	7.33	2.59	3.28E-04	1.79E-02
	T cell activation (P00053)	88	12	3.75	3.2	7.82E-04	1.83E-02
	FGF signaling pathway (P00021)	121	15	5.16	2.91	4.58E-04	1.88E-02
	VEGF signaling pathway (P00056)	68	10	2.9	3.45	1.26E-03	2.30E-02
	Axon guidance mediated by netrin (P00009)	34	7	1.45	4.83	1.24E-03	2.55E-02
	CCKR signaling map (P06959)	172	17	7.33	2.32	2.37E-03	3.88E-02
hsa-miR-132-3p	Gonadotropin-releasing hormone receptor pathway (P06664)	232	24	5.23	4.59	2.88E-09	2.36E-07
	EGF receptor signaling pathway (P00018)	136	16	3.07	5.22	2.65E-07	1.45E-05
	TGF-beta signaling pathway (P00052)	98	13	2.21	5.88	1.04E-06	4.25E-05
	CCKR signaling map (P06959)	172	16	3.88	4.13	4.52E-06	1.48E-04
	FGF signaling pathway (P00021)	121	13	2.73	4.77	8.63E-06	2.36E-04
	Wnt signaling pathway (P00057)	317	20	7.15	2.8	6.55E-05	1.34E-03
	B cell activation (P00010)	70	9	1.58	5.7	5.97E-05	1.40E-03
	Angiogenesis (P00005)	172	13	3.88	3.35	2.46E-04	4.48E-03
	PI3 kinase pathway (P00048)	52	7	1.17	5.97	3.07E-04	4.57E-03
	Interleukin signaling pathway (P00036)	88	9	1.98	4.54	2.93E-04	4.80E-03
	Integrin signalling pathway (P00034)	191	13	4.31	3.02	6.27E-04	7.91E-03
	PDGF signaling pathway (P00047)	145	11	3.27	3.37	7.04E-04	8.25E-03
	Parkinson disease (P00049)	98	9	2.21	4.07	6.05E-04	8.27E-03
	p53 pathway (P00059)	87	8	1.96	4.08	1.20E-03	1.31E-02
	p53 pathway feedback loops 2 (P04398)	50	6	1.13	5.32	1.42E-03	1.46E-02
	Axon guidance mediated by netrin (P00009)	34	5	0.77	6.52	1.58E-03	1.53E-02
	Ras Pathway (P04393)	74	7	1.67	4.2	2.07E-03	1.88E-02
	Hedgehog signaling pathway (P00025)	22	4	0.5	8.07	2.37E-03	2.05E-02
	Insulin/IGF pathway-protein kinase B signaling cascade (P00033)	39	5	0.88	5.69	2.73E-03	2.24E-02
	Axon guidance mediated by Slit/Robo (P00008)	27	4	0.61	6.57	4.58E-03	3.57E-02
	T cell activation (P00053)	88	7	1.98	3.53	5.05E-03	3.77E-02
	VEGF signaling pathway (P00056)	68	6	1.53	3.91	5.82E-03	4.15E-02
	Insulin/IGF pathway-mitogen activated protein kinase kinase/MAP kinase cascade (P000	31	4	0.7	5.72	7.11E-03	4.86E-02
